# Redox-Neutral Organometallic Elementary Steps at Bismuth: Catalytic Synthesis of Aryl Sulfonyl Fluorides

**DOI:** 10.1021/jacs.1c11463

**Published:** 2021-12-16

**Authors:** Marc Magre, Josep Cornella

**Affiliations:** Max-Planck-Institut für Kohlenforschung, Kaiser-Wilhelm-Platz 1, Mülheim an der Ruhr, 45470, Germany

## Abstract

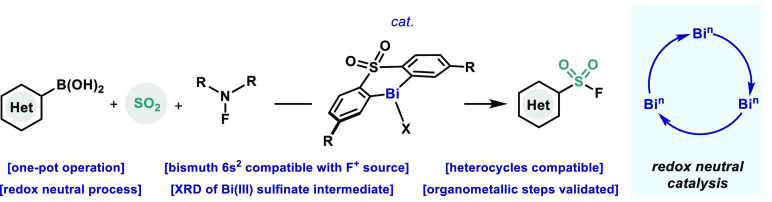

A Bi-catalyzed synthesis of sulfonyl fluorides from the corresponding (hetero)aryl boronic acids is presented. We demonstrate that the organobismuth(III) catalysts bearing a bis-aryl sulfone ligand backbone revolve through different canonical organometallic steps within the catalytic cycle without modifying the oxidation state. All steps have been validated, including the catalytic insertion of SO_2_ into Bi–C bonds, leading to a structurally unique O-bound bismuth sulfinate complex. The catalytic protocol affords excellent yields for a wide range of aryl and heteroaryl boronic acids, displaying a wide functional group tolerance.

Organic molecules bearing a sulfonyl fluoride group (R–SO_2_F) have gained interest in both the fields of chemistry and biology, due to their balanced reactivity and stability under physiological conditions. Among many other applications, these functionalities have found promising applications as covalent protein inhibitors and biological probes.^1,2^ However, the common synthetic methods to obtain such compounds mainly relied on the Cl/F exchange from the parent sulfonyl chloride.^3^ Since 2014, when Sharpless and co-workers introduced the concept of “Sulfur(VI) Fluoride Exchange” (SuFEx) as a powerful reaction for click-chemistry,^4^ intense efforts have been placed in developing alternative routes toward aryl- and alkyl sulfonyl fluorides with broad functional group tolerance and from readily available starting materials.^5^ These efforts have resulted in several transformations that depart from the canonical S(VI) starting material precursor and offer the possibility to engage simple organic halides in cross-coupling-type strategies.^6,7^ In this regard, the pioneering work of Mascitti^8^ and Willis^9^ on Pd-catalyzed sulfur dioxide activation toward the synthesis of sulfones and sulfonamides using SO_2_-surrogates such as K_2_S_2_O_5_ and DABSO (1,4-diazabicyclo[2.2.2]octane bis(sulfur dioxide) adduct) opened a new field on the use of sulfur dioxide for the synthesis of sulfur(VI) containing compounds.^10^ Specifically, Willis^11^ and co-workers demonstrated that this catalytic platform could be applied in the synthesis of (hetero)aryl sulfonyl fluorides from the corresponding aryl bromides and DABSO. Since then, different synthetic methodologies based on Pd- and Cu-catalytic systems allow the conversion of *electrophiles* such as aryl iodides,^12^ alkenyl triflates,^13^ or arenediazonium salts^14^ to the corresponding aryl sulfonyl fluorides (Figure 1A). On the other hand, the use of *aryl nucleophiles* as aryl sources in catalytic protocols has been less studied, with only limited examples reported by Willis and co-workers via Cu(I)^15^ and Ni(II)-catalysis.^16^ These protocols generally occur in two steps, due to incompatibility of the electrophilic fluorinating agent with the catalytic system (Figure 1A). Yet, all these synthetic precedents draw upon the use of transition metals, and synthesis of sulfonyl fluorides via main group catalysis still remains challenging.

**Figure 1:**
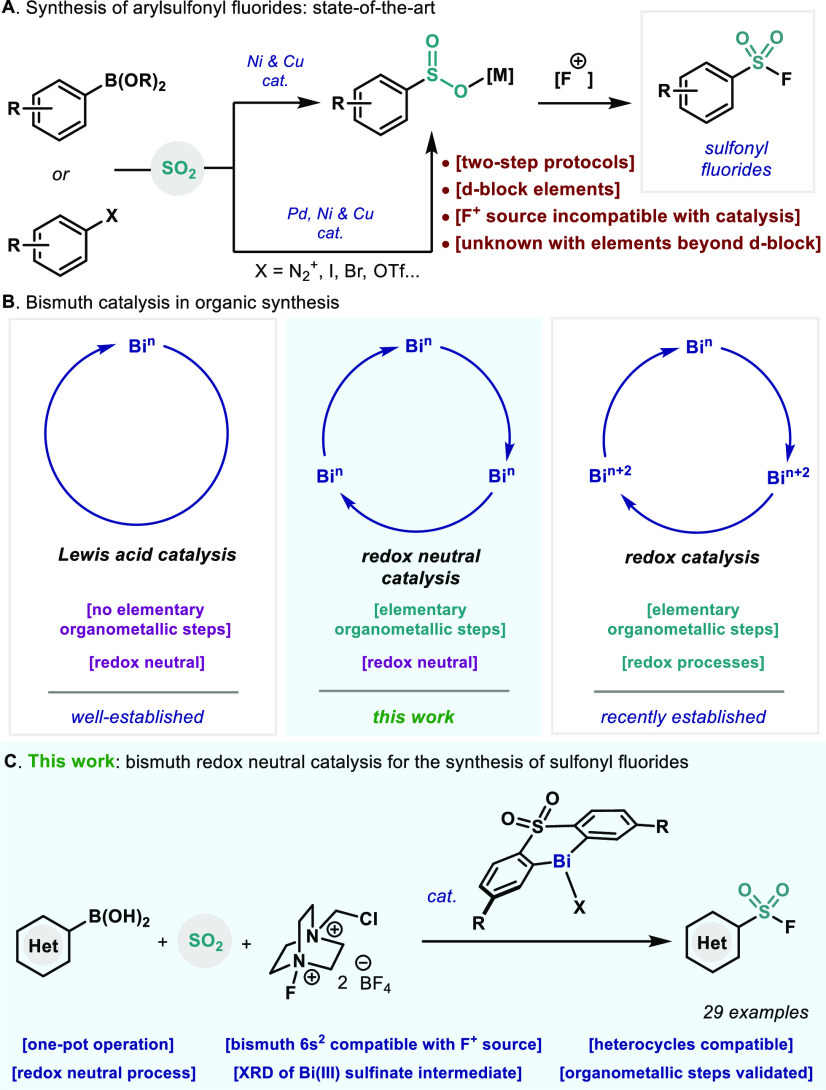
(A) State-of-the art synthesis of aryl sulfonyl fluorides; (B) Bismuth catalysis in organic synthesis; (C) This work: Bismuth(III) redox neutral catalysis for the synthesis of sulfonyl fluorides.

At present, two main catalytic platforms dominate the field of bismuth catalysis in organic synthesis. On one hand, the extensively studied and long-known Lewis acid catalysis, where the bismuth catalyst does not undergo redox processes or participate in catalytic organometallic elementary steps.^17^ On the other hand, bismuth can undergo redox catalysis^18^ through elementary organometallic steps, maneuvering between Bi(I)/(III),^19^ Bi(II)/Bi(III),^20^ or Bi(III)/(V)^21^ (Figure 1B). Herein, we demonstrate that a third catalytic platform for bismuth can also be operative in the conversion of (hetero)aryl boronic acids to the corresponding sulfonyl fluorides. A well-defined organobismuth catalyst revolves through the catalytic cycle maintaining the Bi(III) oxidation state and mimicking elementary organometallic steps. We demonstrate that transmetalation and insertion of sulfur dioxide into the Bi–C(sp^2^) occur effectively delivering a Bi(III)–OS(O)Ar compound. Importantly, the low reactivity of the 6s^2^ lone pair in bismuth permits the presence of electrophilic fluorinating agents and a one-pot synthetic operation (Figure 1C).

We started our investigations by optimizing the reaction between phenyl boronic acid (**1a**) and sulfur dioxide in the presence of Selectfluor as an oxidant (Table 1). Based on our previous work,^21a,21b^ bismuth complexes bearing diarylsulfone ligands (**3a**–**e**) are excellent candidates for mimicking organometallic steps efficiently. Due to the minimal difference in reactivity between Bi complexes bearing different couteranions (BF_4_ in **3a** and OTs in **4**; Table 1, entry 1 vs 2), Bi complexes bearing a BF_4_ were preferentially chosen due to lower MW when compared to OTs-containing catalysts. Sulfone ligand screening showed that catalyst **3c**, with a CF_3_- and a Me group at the *meta*-position in respect to the Bi atom, provided the best conversion of phenyl boronic acid **1a** to **2a** (Table 1, entry 4). Due to solubility issues of the oxidant, a solvent mixture of CDCl_3_/CH_3_CN 5:1 proved to be optimal (Table 1, entry 7). When a stronger base such as Na_2_CO_3_ was tested, formation of benzene was observed, thus decreasing the yield of our desired sulfonyl fluoride (Table 1, entry 8). We were delighted to see that the catalyst loading could be decreased to 5 mol % maintaining the catalytic activity (Table 1, entry 9 vs 7). In agreement with previous results,^21b^ in the absence of base or molecular sieves, undesired benzene forms majorly, which arises from protonation of either B–Ph or Bi–Ph bonds (Table 1, entries 10 and 11). Finally, without the presence of bismuth catalyst **3c**, no phenyl sulfonyl fluoride **2a** was obtained (Table 1, entry 12). It is worth mentioning that the replacement of sulfur dioxide by DABSO was detrimental, decreasing the yield of **2a** (Table 1, entry 13), presumably due to incompatibility of DABCO with the catalytic system.

**Table 1:**
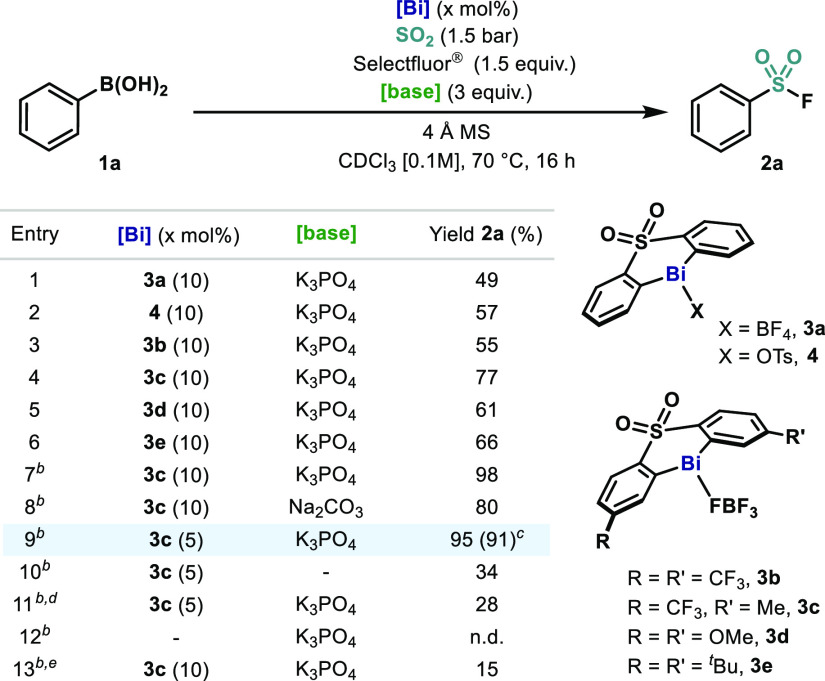
Optimization of the Reaction Conditions^a^

a Reactions performed at 0.05 mmol of **1a**. Yields determined by ^1^H and ^19^F NMR using 1,4-difluorobenzene as internal standard.

b CDCl_3_:CH_3_CN mixture of 5:1 was used as solvent.

c Isolated yield of a reaction performed at 0.2 mmol of **1a**.

d No MS were used.

e DABSO (1.5 equiv) was used instead of SO_2_ (1.5 bar).

With the optimal conditions in hand, the scope was investigated (Table 2). Aryl boronic acids bearing alkyl (**1b**) or halide groups (**1c**–**d**) provided the desired compounds in excellent yields. On the other hand, more electron-rich substrates (**2e**–**f**) performed with lower efficiency. We were pleased to see that electronically and sterically distinct substituents at the *meta*-position were tolerated, attaining the sulfonyl fluoride products (**2g**–**k**) in good yields. Remarkably, sterically hindered aryl boronic acids (**1l**–**1m**) also performed well, showing that the presence of substituents at the *ortho*-position do not affect the catalytic performance of **3c**. Also polyaromatic sulfonyl fluorides (**2n**–**o**) could be isolated in nearly quantitative yields. Contrary to previous Bi-catalyzed redox processes,^21^ this protocol exhibited high functional group compatibility. Indeed, boronic acids containing SiMe_3_ (**1p**), vinyl (**1q**), alkynyl (**1r**), formyl (**1s**), and ester (**1t**) were converted to their corresponding sulfonyl fluorides (**2p**–**t**) in moderate to good yields. Boronic acid containing a benzylic ether position (**1u**) performed well, and no activation of the ether was observed.^22^ More importantly, aryl boronic acids containing *N*-protected anilines in both *para*- and *meta*-positions were also tolerated, achieving good yields of the *N*-Ms (**2v**) and *N*-Boc (**2w**) aryl sulfonyl fluorides, respectively. When heterocyclic boronic acids were tested, a re-evaluation of the catalytic system was required (see Supporting Information). It was found that the combination of **4** and NFSI as a milder oxidant was crucial to convert heteroaryl boronic acids to their corresponding sulfonyl fluorides. Thus, benzofuran (**2x**), furan (**2y**), and benzothiophene (**2z**) were well accommodated. More reactive heteroaryl boronic acids such as unprotected 1*H*-indole (**1aa**), 5-quinoline (**1ab**), and isoxazole (**1ac**) could also be converted to their corresponding sulfonyl fluorides in moderate to good yields, competing favorably with the transition-metal-catalyzed reports.^11,16^ Tolerance of heterocyclic frameworks is a step forward in the field of bismuth catalysis, as coordination to Bi(III) centers and incompatibility with strong oxidants usually precludes reactivity.

**Table 2:**
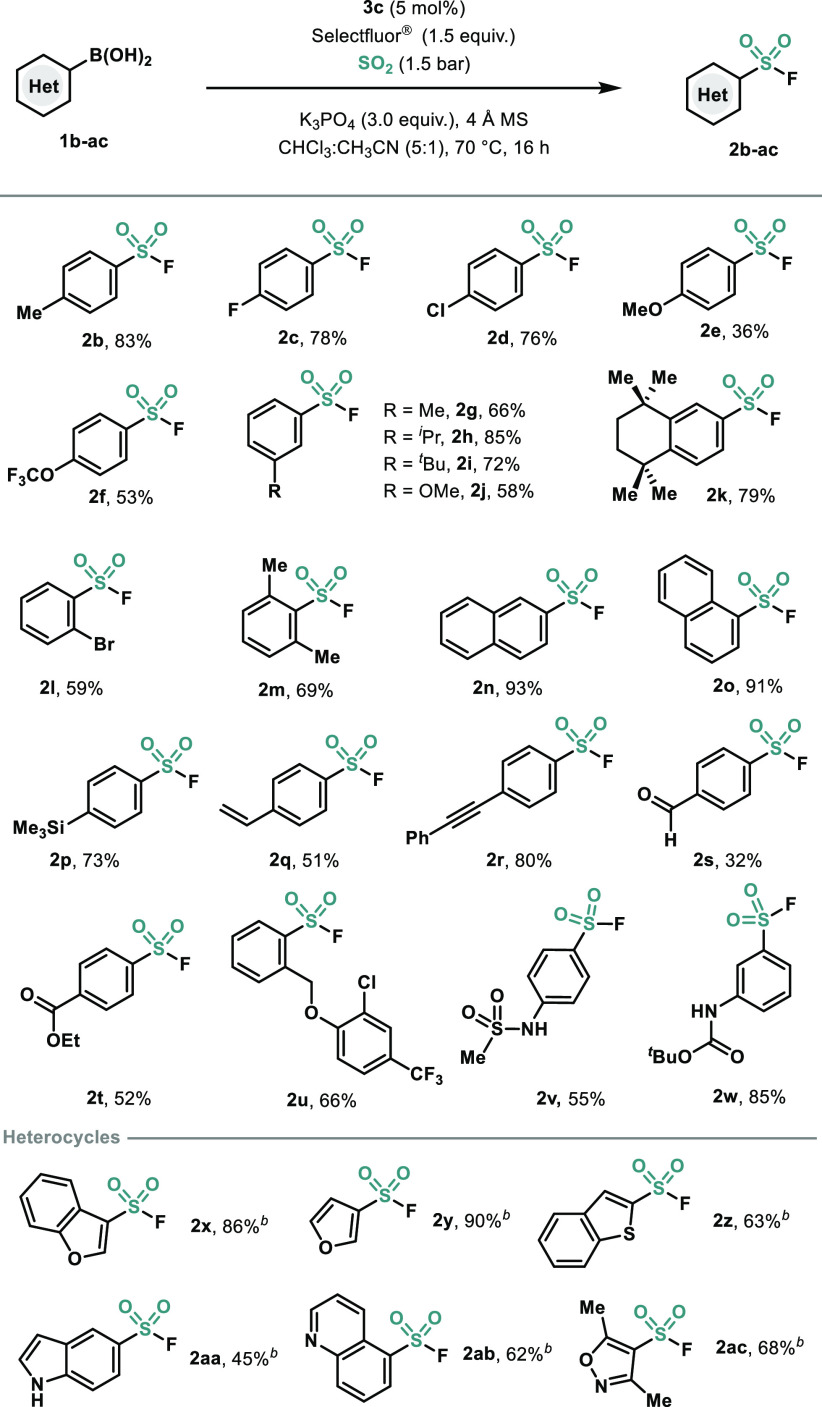
Scope of the Bi-Catalyzed Synthesis of (Hetero)Aryl Sulfonyl Fluorides^a^

a Reaction conditions: **1** (0.2 mmol), **3c** (5 mol %), Selectfluor (0.3 mmol), K_3_PO_4_ (0.6 mmol) and 4 Å MS (40 mg) in CHCl_3_/CH_3_CN (5:1, 2 mL) at 70 °C for 16 h. Yields of isolated pure material after column chromatography.

b Reaction conditions: **1** (0.17 mmol), **4** (10 mol %), NFSI (0.26 mmol), K_2_CO_3_ (0.51 mmol) in CHCl_3_:H_2_O (5%, 2 mL) at 60 °C for 16 h. Yields of isolated pure material after column chromatography.

At this point, the operative mechanism governing this transformation was explored (Scheme 1). In accordance with previous precedents in our group,^21b^ the transmetalation step between Bi catalyst **3c** and **1a** occurred smoothly, affording **5c** in excellent yields (Scheme 1A). Stoichiometric precedents of arylbismuth complexes reacting with SO_2_ propose that insertion can occur at Bi(III)–C^23^ or Bi(V)–C^24^ bonds. In order to investigate whether our catalytic systems proceeds through the former or the latter, we subjected **5c** to a series of oxidation/insertion sequences (Scheme 1B). After exposure of **3c** to Selectfluor and, subsequently, to an atmosphere of SO_2_, only trace amounts of the desired product **2a** were observed (Scheme 1B, *path a*). Similarly, exposure of cationic Bi(V) complex **6** to SO_2_ atmosphere at 70 °C resulted in remarkably low yield of **2a** (Scheme 1B, *path b*). In both cases, formation of fluorobenzene and benzene resulted as the main byproducts (see Supporting Information). This is in agreement with our previous studies, in which cationic pentavalent bismuth species (**6**) are active toward the synthesis of fluorobenzene, via reductive elimination/ligand coupling pathways.^21a^ With a Bi(V) intermediate being highly unlikely, it was envisioned that maybe **5c** could be active toward sulfur dioxide insertion. Treatment of **5c** with SO_2_ resulted in rapid formation of the corresponding diarylbismuth sulfinate **7**, which was fully characterized by NMR and HRMS and single crystal X-ray diffraction (Scheme 1C). This is in stark contrast to previous stoichiometric reports, where SO_2_ reacted with a Bi(V) complex,^24^ and is in agreement with SO_2_ insertion at organobismuth(III) complexes.^23^ It is worth mentioning that diaryl bismuth sulfinate **7** is also obtained when SO_2_ is replaced by DABSO, albeit in lower yields (see Supporting Information). Generally, diarylbismuth benzenesulfinates are prepared via protonolysis of triarylbismuth complexes with arylsulfinic acid.^25^ Although infrared spectra suggested monodentate O-sulfinate coordination to the bismuth center, no structural confirmation via single crystal XRD has been reported. Therefore, this work provides solid evidence that SO_2_ insertion occurs in the Bi(III)–C(sp^2^)^26^ affording the monodentate O-sulfinate diarylbismuth compound **7**. Although the O-bound structure in **7** is not surprising for bismuth(III) due to its high oxo-philicity,^27^ it differs from previous crystal structures reported from the SO_2_ insertion into Pd–Ph^10b^ and Au–Ph^10a^ bonds, where the S-bound coordination to the metal is obtained in all cases. Encouraged by these results, we subjected **7** to oxidation with Selectfluor, and **2a** was obtained at both 25 and 70 °C, along with the regeneration of precatalyst **3c** (Scheme 1C). In order to rule out a possible mechanism which would involve a Bi(III) oxidation after the SO_2_ insertion step, we subjected diarylbismuth tosylate **8**, which contains a Bi(III) and a S(VI) atom to oxidation with Selectfluor (Scheme 1D). Quantitative recovery of **8** and Selectfluor was observed, indicating no oxidation of Bi(III) species. This result, together with the mild conversion of **7** to **2a** under mild conditions, suggests that Selectfluor reacts preferentially with the S(IV), in agreement with previous fluorination of metal aryl sulfinates (see Supporting Information).^11−16^

**Scheme 1:**
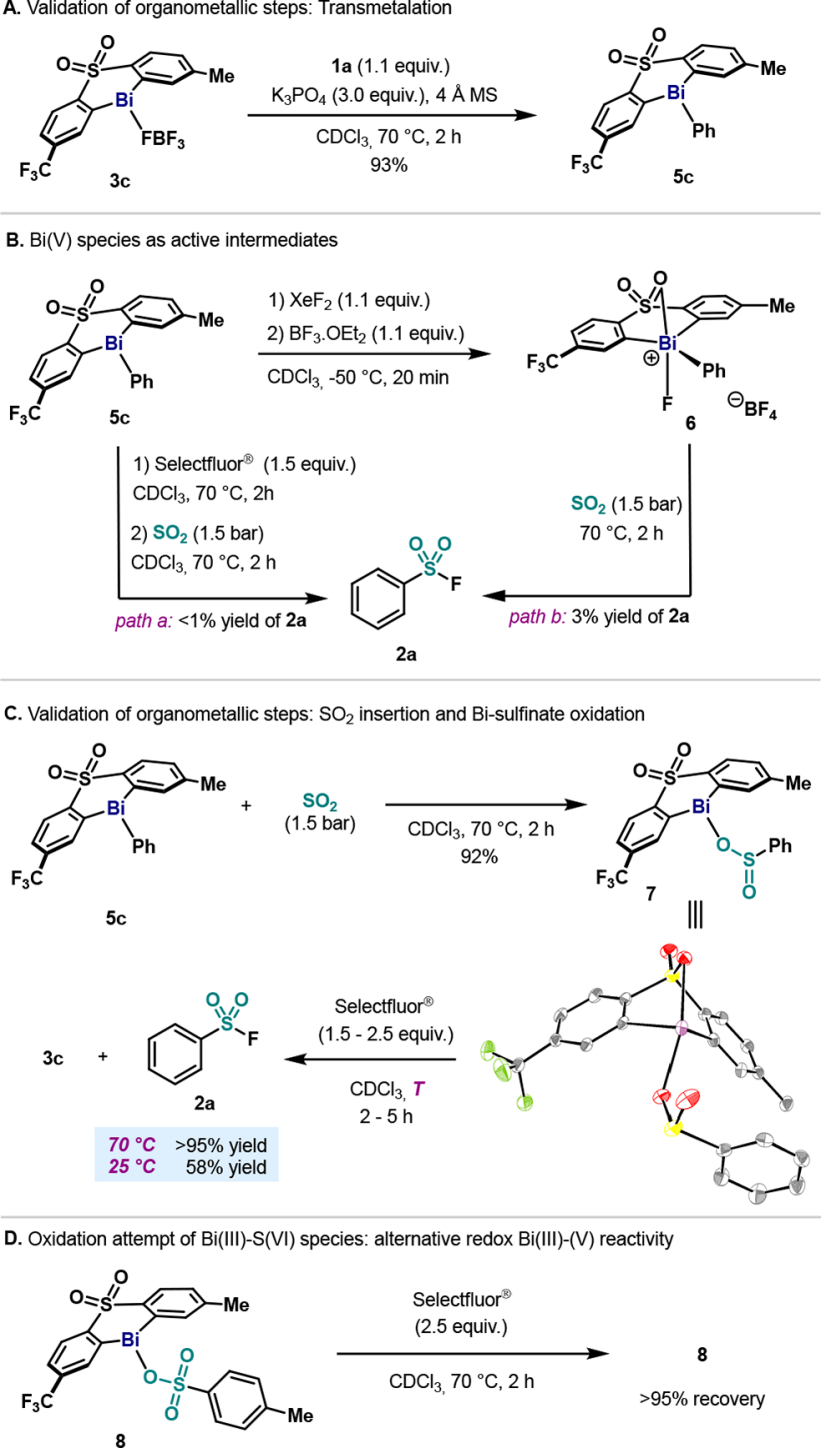
(A) Transmetalation step; (B) Cationic Bi(V) Species As Putative Reactive Intermediate; (C) SO_2_ Insertion and Bi-Sulfinate Oxidation; (D) Oxidation of Bi(III)–S(VI) Species Inset picture: ORTEP of **7**. This compound is a dimer in the unit cell. See SI for details. H atoms are omitted for clarity.

With this mechanistic insight, a plausible mechanism for the conversion of (hetero)aryl boronic acids to their corresponding sulfonyl fluorides is proposed in Scheme 2. Initially, bismuth complex **A** undergoes transmetalation (TM) with the corresponding (hetero)aryl boronic acid **1**, forming triarylbismuth complex **B**. Consequently, sulfur dioxide undergoes Bi–C bond insertion in **B**, leading to bismuth sulfinate intermediate **C**, which upon oxidation of the S(IV) affords the corresponding aryl sulfonyl fluoride **2** with the concomitant regeneration of **A**.

**Scheme 2:**
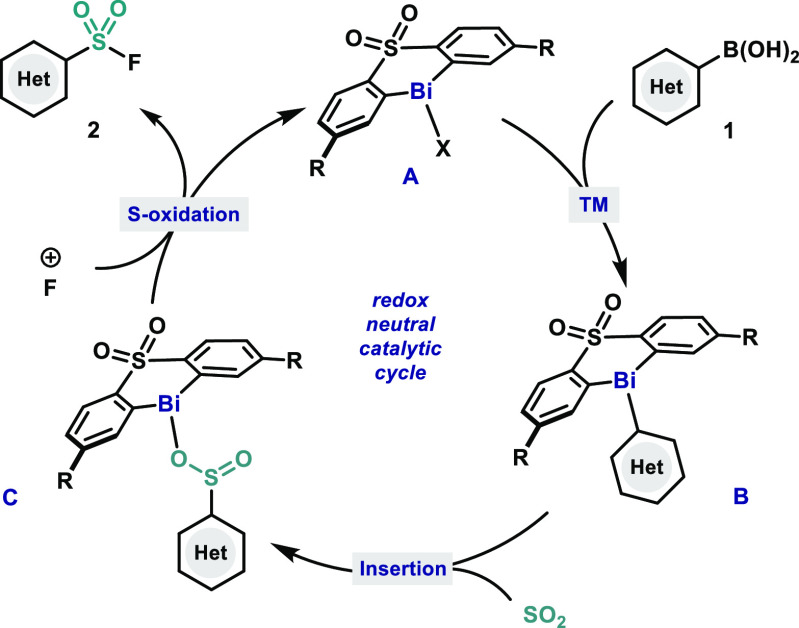
Proposed Mechanism: Redox Neutral Bi(III)-Catalyzed Synthesis of (Hetero)Aryl Sulfonyl Fluorides

In summary, a unique Bi(III)-catalyzed conversion of aryl boronic acids to the corresponding (hetero)arylsulfonyl fluorides has been developed. The canonical organometallic steps by which the Bi complex undergoes catalysis have been elucidated and validated. Transmetalation of boronic acid to the bismuth is followed by a SO_2_ insertion into a Bi–C bond under mild conditions, attaining the corresponding diarylbismuth sulfinate. This novel catalytic cycle results in good to excellent yields and a wide substrate scope, accommodating challenging heteroaryl boronic acids. The results presented in this study reveal bismuth redox neutral catalysis as a promising tool to perform transformations mimicking the fundamental organometallic steps of transition metal catalysts, thus expanding the palette of opportunities for bismuth catalysis in organic synthesis.

## Supporting Information Available

Experimental procedures and analytical data (^1^H, ^13^C, and ^19^F NMR, HRMS) for new compounds; crystallographic data for compound **7** (PDF)

## Accession Codes

CCDC 2118151 contains the supplementary crystallographic data for this paper. These data can be obtained free of charge via www.ccdc.cam.ac.uk/data_request/cif, or by emailing data_request@ccdc.cam.ac.uk, or by contacting The Cambridge Crystallographic Data Centre, 12 Union Road, Cambridge CB2 1EZ, UK; fax: +44 1223 336033.
