# Supplementary material for: Redox-Neutral Organometallic Elementary Steps at Bismuth: Catalytic Synthesis of Aryl Sulfonyl Fluorides
Source: J Am Chem Soc. 2021 Dec 16;143(51):21497–502. doi: 10.1021/jacs.1c11463 (PMC8719321; doi:10.1021/jacs.1c11463)

Supporting Information

Redox-neutral Organometallic Elementary Steps at Bismuth: Catalytic Synthesis of Arylsulfonyl Fluorides

Marc Magre and Josep Cornella*

Max-Planck-Institut für Kohlenforschung, Kaiser-Wilhelm-Platz 1, Mülheim an der Ruhr, 45470, Germany.

cornella@kofo.mpg.de

Table of Contents

1. General methods S3
2. Synthesis of ligands S4
3. Synthesis of Bi(III) compounds S7
   3.1. Synthesis of triarylbismuth compounds **5a-e** S7
   3.2. Synthesis of diarylbismuth tetrafluoroborate compounds **3a-e** S9
   3.3. Synthesis of diarylbismuth tosylate **4** S11
4. Stoichiometric Optimization S12
   4.1. Optimization of ligand backbone S12
   4.2. Transmetalation step S13
      4.2.1. Transmetalation of phenyl boronic acid **1a** S13
      4.2.2. Transmetalation of benzofuran-3-boronic acid **1x** S14
   4.3. SO_2-insertion step S15
   4.4. Bi-sulfinate oxidation S17
5. Catalytic studies S18
   5.1. Optimization of the Bi-catalyzed synthesis of phenyl sulfonyl fluoride **2a** S18
   5.2. Optimization of the Bi-catalyzed synthesis of benzofuran-3-sulfonyl fluoride **2x** S20
   5.3. Scope of Bi-catalyzed synthesis of (hetero)aryl sulfonyl fluorides **2a**-**2ac** S21
6. Mechanistic studies S33
   6.1. Validation of the organometallic steps: Transmetalation S33
   6.2. Bi(V) species as active intermediates S34
      6.2.1. Path a S34
      6.2.2. Path b S36
   6.3. Validation of the organometallic steps: SO_2 insertion into Bi(III)–C bond S38
      6.3.1. SO_2 insertion into Bi–C bond in **5c** with SO_2 gas S38
      6.3.2. SO_2 insertion into Bi–C bond in **5c** using DABSO S39
   6.4. Validation of the organometallic steps: Bi–sulfinate **7** oxidation S40
   6.5. Oxidation of Bi(III)–S(VI) species S41
   6.6. Reactivity of diarylbismuth tetrafluoroborate **3c** S44
      6.6.1. Reactivity with SO_2 S44
      6.6.2. Reactivity with Selectfluor® S45
7. Proposed mechanism S46
8. Single crystal structure analysis of bismuth sulfinate **7** S47
9. References S54
10. Spectral data: ^{1}H, ^{13}C and ^{19}F NMR spectra S55

1. General methods

Unless otherwise stated, all manipulations were performed using standard Schlenk techniques under dry argon in flame-dried glassware. Anhydrous solvents were distilled from appropriate drying agents and were transferred under Ar: tetrahydrofuran (Na/K), chloroform and chloroform-*d* (CaH_2), acetonitrile (SPS) and acetonitrile-d_3 (MS), dichloromethane (CaH_2/P_4O_{10}). Commercially available xenon difluoride, triphenyl bismuth and bismuth tribromide were obtained from STREM, 1-fluoro-2,6-dichloropyridinium tetrafluoroborate, Selectfluor®, NFSI and all the arylboronic acids utilized in this work were obtained from Sigma-Aldrich. Sulfur dioxide was obtained from Air liquid (2.74Kg, 99.98%). 4 Å Molecular sieves were dried at 250 °C under high vacuum for 2 days before use. Flash chromatography: Merck silica gel 60 (40-63 µm). ESI-MS: ESQ 3000 (Bruker). Preparative TLC plates: PLC Silica gel 60 F_{254}, 1 mm, 20x20 cm (Sigma-Aldrich). High-resolution mass determinations: Bruker APEX III FT-MS (7 T magnet) or MAT 95 (Finnigan). NMR spectra were recorded using 300 and 400 MHz Bruker Avance III and 600 MHz Bruker Avance III NMR spectrometers. ^{1}H NMR spectra (300.13 MHz, 400.0 MHz and 600.1 Hz) were referenced to the residual protons of the deuterated solvent,[1] and are reported to tetramethylsilane (δ_{TMS} = 0 ppm), chloroform-*d* (δ_{TMS} = 7.26 ppm) or acetonitrile-d_3 (δ_{TMS} = 1.94 ppm). $^{13}C\{^{1}H\}$ NMR spectra (75 MHz, 101 MHz, 151 MHz) were referenced internally to the D-coupled ^{13}C resonances of the NMR solvent and are reported to tetramethylsilane (δ_{TMS} = 0 ppm), chloroform-*d* (δ_{TMS} = 77.16 ppm) or acetonitrile-d_3 (δ_{TMS} = 1.32 ppm). $^{19}F\{^{1}H\}$ NMR spectra (282 MHz, 471 MHz) are reported relative to the ^{19}F resonances of $CFCl_3$. Chemical shifts (δ) are given in ppm, relative to deuterated solvent residual peak, and coupling constants (*J*) provided in Hz.

IMPORTANT: All the reactions that required gaseous SO_2 were carried out in a J-Young Schlenk and placed in a well-ventilated fume hood.

2. Synthesis of ligands

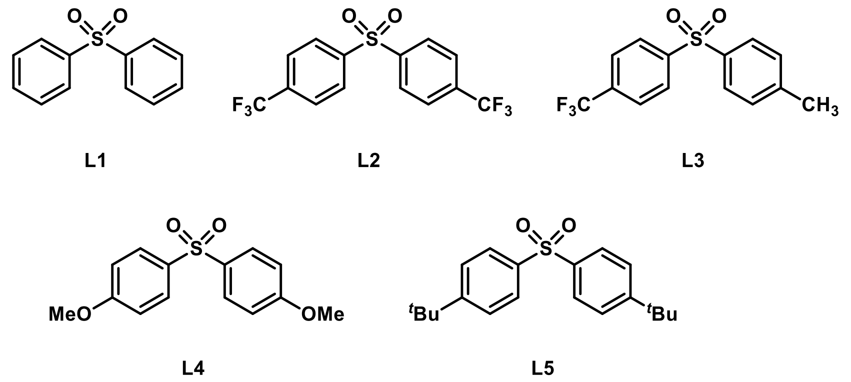

Figure S1. Ligands utilized in this study.

Ligands **L1-L5** were synthesized from the corresponding thioether (obtained either by *Procedure 1*[1] or *Procedure 2*) followed by oxidation with *meta*-chloroperbenzoic acid (*m*CPBA).[2] Characterization and spectroscopic data for diarylsulfones **L1**[3], **L2**[4] and **L3**[5] match with the reported values in the literature.

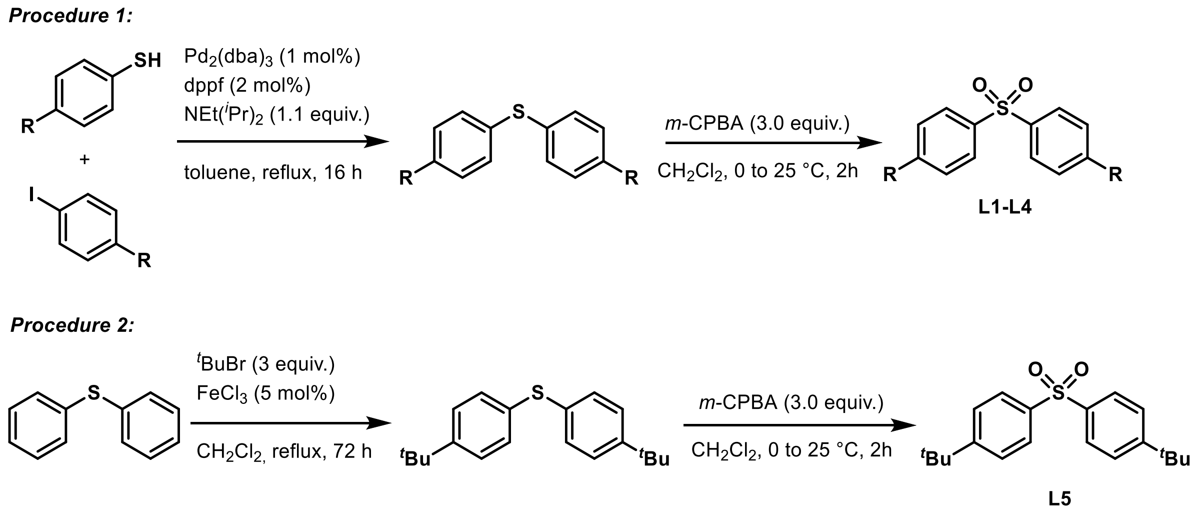

Scheme S1. General synthetic strategies for the synthesis of **L1-L5.**

Ligand **L4** was synthesized using *Procedure 1,* which is described below and the spectroscopic data match with reported values in the literature.[6]

Ligand **L5**, which to the best of our knowledge has not been reported before, was synthesized using *Procedure 2* and its synthesis and spectroscopic characterization is described below.

2.1. Procedure 1 for the synthesis of diarylsulfone ligand ***L4***

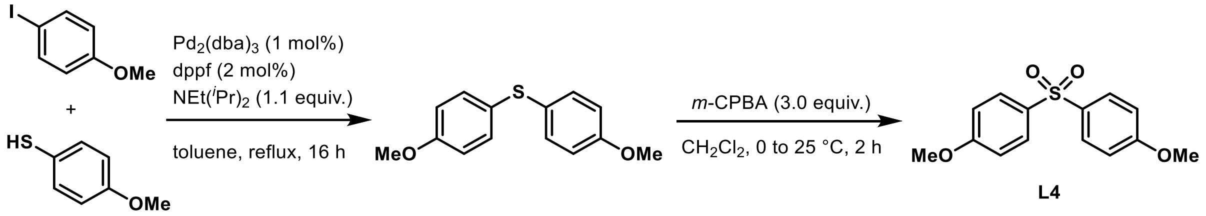

4-iodo anisole (7.13 mmol, 1.7 g), DIPEA (1.1 equiv., 1.4 mL) and 4-methoxythiophenol (1 equiv., 0.9 mL) were added to a solution of $Pd_2(dba)_3$ (1 mol%, 65 mg) and 1,1'-Bis-(diphenylphosphino)ferrocene (dppf) (2 mol%, 79 mg) in dry toluene (90 mL) at 25 °C. After the addition, the mixture was refluxed for 16 h. After cooling down to 25 °C, H_2O (20 mL) was added and the aqueous phase was extracted with EtOAc (3 × 25 mL). The combined organic phases were washed with sat. NaCl (aq.) solution (40 mL) and dried over $MgSO_4$. After evaporation of the volatiles under vacuum, the crude was purified by flash chromatography (SiO_2, hexanes:EtOAc 9:1) as eluent system. The 4,4'-dimethoxy diphenyl sulfide was obtained as colorless oil (1.6 g, 91% yield).[7]

4,4'-Dimethoxy diphenyl sulfide (4.06 mmol, 1.0 g) was dissolved in 100 mL of CH_2Cl_2 at 0 °C. Then, *m*-CPBA (3 equiv., 2.1 g) was added. After 10 min at 0 °C, the reaction is warmed to 25 °C. After 2 h, an aqueous solution of NaOH (1M, 30 mL) is added, extracted with CH_2Cl_2 (2 × 20 mL) and the combined organic phases are dried over $MgSO_4$. Evaporation of the volatiles under vacuum affords **L4** as a white solid (1.06 g, 94% yield). The analytical data match with the reported values in the literature.[6]

2.2. Procedure 2 for synthesis of diarylsulfone ligand ***L5***

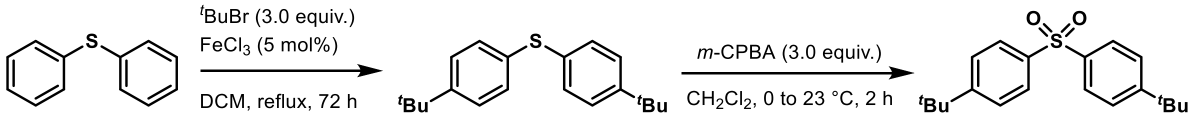

To a CH_2Cl_2 solution (5 mL) of phenyl sulfide (15 mmol, 2.5 mL) and ferric chloride (5 mol%, 122 mg) a CH_2Cl_2 solution (5 mL) of *tert*-butyl bromide (3 equiv., 5.1 mL) was added. The mixture was refluxed for 72 h. After cooling down the reaction mixture, the crude solution was washed with 10% HCl aqueous solution (100 mL), water (100 mL), 5% NaOH aqueous solution (100 mL) and finally with a sat. NaCl (aq.) solution (50 mL). Drying the organic phase over $MgSO_4$ and removal of volatiles under vacuum gave a yellowish solid (4.4 g, 98% yield) that was used without further purification.[8]

4,4’-Di-*tert*-butyl diphenyl sulfide (4.0 mmol, 1.2 g) was dissolved in 100 mL of CH_2Cl_2 at 0 °C. Then, *m*-CPBA (3 equiv., 2.1 g) was added. After 10 min at 0 °C, the reaction is warmed to 25 °C. After 2 h, a, aqueous solution of NaOH (1M, 30 mL) is added, extracted with CH_2Cl_2 (2 × 20 mL) and the combined organic phases dried over $MgSO_4$. Evaporation of the volatiles under vacuum affords **L5** as a white solid (1.3 g, 95% yield).

^{1}H NMR (300 MHz, $CDCl_3$): δ 7.81 – 7.77 (m, 4H), 7.45 – 7.40 (m, 4H), 1.23 (s, 9H).

^{13}C NMR (75 MHz, $CDCl_3$): δ 156.8, 138.9, 127.4, 126.2, 35.1, 31.0.

HRMS (EI, m/z): calc’d for $C_{20}H_{26}O_2S_1$ $[M]^+$ 330.1648; found 330.1651.

3. Synthesis of Bi(III) compounds

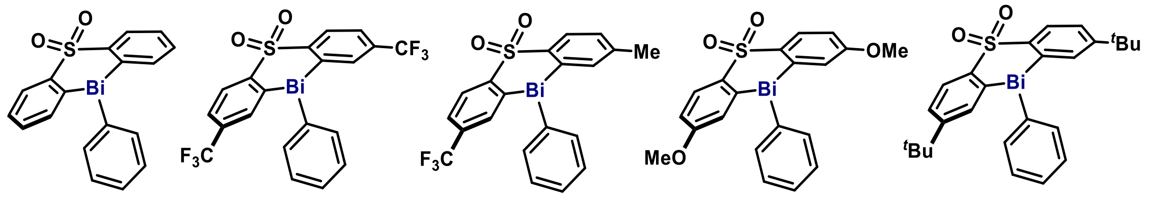

Figure S2. Triarylbismuth compounds **5a-e** synthesized.

Triarylbismuth compounds **5a**[9] and **5b**[4] were synthesized according to a previously reported protocol, and the analytical data match with the values reported in previous literature. Triarylbismuth compounds **5c-e** were synthesized following the procedure described below and fully characterized.

*3.1. Synthesis of triarylbismuth compounds **5a-e***

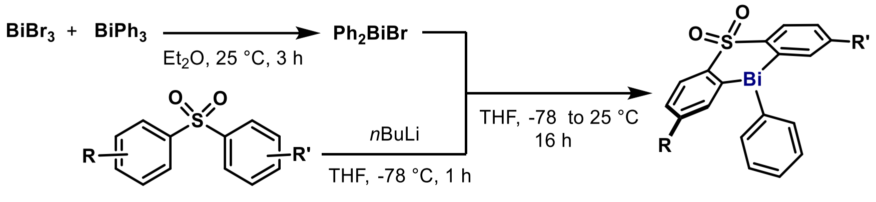

General Procedure: A Schlenk flask equipped with a stir bar was charged with $BiBr_3$ (0.66 equiv.), $BiPh_3$ (0.33 equiv.) and Et_2O (8 mL) under argon atmosphere and stirred at 25 °C for 3 h. THF (10 mL) was added to dissolve the yellow precipitate, and this solution was added dropwise to a solution at –78 °C of the dilithiated ligand. The dilithiated ligand was prepared in a separate Schlenk flask from the corresponding ligand (1 equiv.) dissolved in THF (30 mL) with *n*-butyllithium (2.6 M in hexanes, 2.1 equiv.) at –78 °C for 1 h. Following the addition of the bismuth compound to the dilithiated ligand, the mixture was stirred overnight, slowly warming to 25 °C. The reaction was quenched by the addition of sat. NaCl (aq.) solution (20 mL) and extracted with EtOAc (3 × 40 mL). The combined organics were dried over Na_2SO_4, filtered and concentrated under vacuum. The crude material was purified by flash chromatography (SiO_2, hexanes:EtOAc) to give the corresponding triarylbismuth compounds **5a-e** as solids.

2-Methyl-10-phenyl-8-(trifluoromethyl)-10*H*-dibenzo[*b,e*][1,4]thiabismine-5,5-dioxide (5c): Following the General Procedure, using 1.0 g of **L3** as ligand, **5c** (1.09 g, 56% yield) was obtained as a white solid (Eluent: hexanes:EtOAc 8:2).

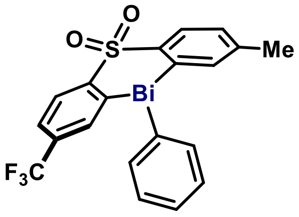

5c

^{1}H NMR (600 MHz, $CDCl_3$): δ 8.44 (dt, *J* = 8.1, 0.7 Hz, 1H), 8.28 (d, *J* = 8.0 Hz, 1H), 8.04 (dt, *J* = 1.9, 0.7 Hz, 1H), 7.78 – 7.75 (m, 2H), 7.71 (dd, *J* = 1.2, 0.6 Hz, 1H), 7.66 (ddd, *J* = 8.1, 1.8, 0.7 Hz, 1H), 7.50 – 7.45 (m, 2H), 7.44 – 7.39 (m, 1H), 7.23 (ddd, *J* = 8.0, 1.7, 0.7 Hz, 1H), 2.28 (s, 3H).

^{13}C NMR (151 MHz, $CDCl_3$): δ 166.3, 159.6, 159.1, 145.7, 144.8, 138.7, 138.3, 138.1, 134.6 (q, *J* = 32.4 Hz), 134.5 (q, *J* = 3.6 Hz), 131.3, 130.3, 129.3, 129.1, 128.2, 127.8, 126.9, 125.4 (q, *J*= 3.7 Hz), 123.4 (q, *J*= 273.4 Hz), 21.6.

^{19}F NMR (565 MHz, $CDCl_3$): δ –62.95

HRMS (ESI, m/z): calc'd for $C_{20}H_{14}BiF_3SO_2Na^+$ $[M+Na]^+$ 607.0363; found 607.0366

2,8-Dimethoxy-10-phenyl-10*H*-dibenzo[*b,e*][1,4]thiabismine-5,5-dioxide (5d): Following the General Procedure, using 500 mg of **L4** as ligand, **5d** (650 mg, 64% yield) was obtained as a white solid (Eluent: hexanes:EtOAc 7:3).

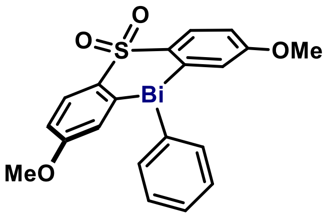

5d

^{1}H NMR (600 MHz, $CDCl_3$): δ 8.28 (d, *J* = 8.6 Hz, 2H), 7.81 (dd, *J* = 7.9, 1.4 Hz, 2H), 7.46 – 7.42 (m, 2H), 7.39 – 7.35 (m, 3H), 6.84 (dd, *J* = 8.6, 2.5 Hz, 2H), 3.69 (s, 6H).

^{13}C NMR (151 MHz, $CDCl_3$): δ 166.5, 163.5, 160.0, 138.8, 134.2, 131.0, 128.7, 128.6, 123.5, 112.7, 55.7.

HRMS (ESI, m/z): calc'd for $C_{20}H_{17}BiSO_4Na^+$ $[M+Na]^+$ 585.0544; found 585.0545

2,8-Di-*tert*-butyl-10-phenyl-10*H*-dibenzo[*b*,*e*][1,4]thiabismine 5,5-dioxide (5e): Following the General Procedure, using 1.1 g of **L5** as ligand, **5e** (1.1 g, 54% yield) was obtained as an off-white solid (Eluent: hexanes:EtOAc 8:2).

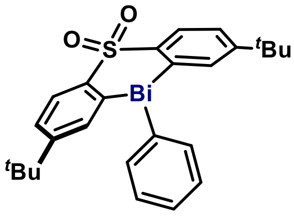

5e

^{1}H NMR (600 MHz, $CDCl_3$): δ 8.26 (dd, *J* = 8.2, 0.4 Hz, 2H), 7.87 (dd, *J* = 1.9, 0.4 Hz, 2H), 7.78 (dd, *J* = 7.9, 1.3 Hz, 2H), 7.44 – 7.39 (m, 2H), 7.38 – 7.34 (m, 3H), 1.15 (s, 18H).

^{13}C NMR (151 MHz, $CDCl_3$): δ 166.0, 158.4, 156.5, 139.2, 138.7, 135.3, 130.7, 128.6, 126.9, 124.9, 35.2, 31.1.

HRMS (ESI, m/z): calc'd for $C_{26}H_{29}BiSO_2Na^+$ $[M+Na]^+$ 637.1585; found 637.1584.

*3.2. Synthesis of diarylbismuth tetrafluoroborate compounds **3a-e***

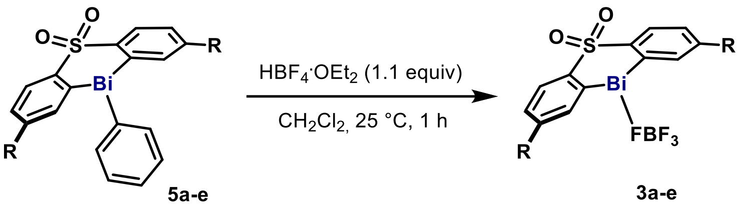

General Procedure: Triarylbismuth compounds **5a-e** (1.0 equiv.) were dissolved in dry CH_2Cl_2 (2 mL) and acid tetrafluoroborate diethylether complex (1.1 equiv.) was added. The reaction mixture was stirred for 1 h at 25 °C and then the solvent was removed under vacuum. The remaining solid was washed with MTBE (2 × 3 mL) to afford complexes **3a-e** as white solids.

Diarylbismuth tetrafluoroborate (3a): Following the protocol described above at 0.64 mmol scale of **5a**, complex **3a** was obtained as a white solid (300 mg, 91% yield).

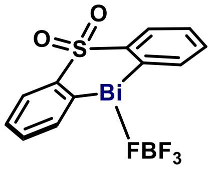

3a

^{1}H NMR (400 MHz, CD_3CN): δ 9.13 (d, *J* = 7.3 Hz, 2H), 8.49 (dd, *J* = 7.7, 1.2 Hz, 2H), 7.96 (td, *J* = 7.5, 1.2 Hz, 2H), 7.63 (td, *J* = 7.6, 1.1 Hz, 2H). Peaks at 4.64 and 1.52 ppm correspond to residual Et_2O coordinated to **3a**.

^{13}C NMR (101 MHz, CD_3CN): δ 196.7, 140.3, 137.5, 136.3, 130.2, 129.9.

^{19}F NMR (282 MHz, CD_3CN): δ –151.23.

HRMS (ESI, m/z): calc'd for $C_{12}H_8BiSO_2^+$ $[M-BF_4]^+$ 425.0043; found 425.0044.

Diarylbismuth tetrafluoroborate (3b): Following the protocol described above at 0.29 mmol scale of **5b**, complex **3b** was obtained as a brownish solid (150 mg, 82% yield).

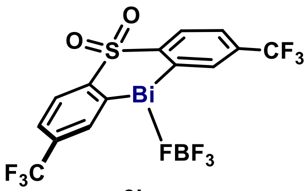

3b

^{1}H NMR (400 MHz, CD_3CN): δ 9.39 (bs, 2H), 8.64 (d, *J* = 8.0 Hz, 2H), 7.92 (dd, *J* = 8.1, 0.9 Hz, 2H). Peaks at 4.67 and 1.52 ppm correspond to residual Et_2O coordinated to **3b**.

^{13}C NMR (101 MHz, CD_3CN): δ 144.1, 137.5 (q, *J* = 32.5 Hz), 133.1 (q, *J* = 3.8 Hz), 130.7, 127.1 (d, *J* = 3.9 Hz), 124.6 (q, *J* = 273.0 Hz).

^{19}F NMR (282 MHz, CD_3CN): δ –63.45 (6F), –151.52 (4F).

HRMS (ESI, m/z): calc'd for $C_{14}H_6BiF_6SO_2^+$ $[M-BF_4]^+$ 560.9791; found 560.9798.

Diarylbismuth tetrafluoroborate (3c): Following the protocol described above at 0.92 mmol scale of **5c**, complex **3c** was obtained as an off-white solid (500 mg, 92% yield).

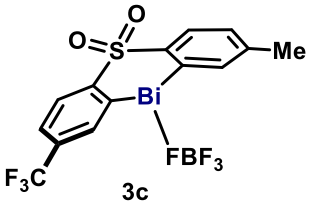

3c

^{1}H NMR (400 MHz, CD_3CN): δ 9.01 (bs, 1H), 8.69 (bs, 1H), 8.53 (d, *J* = 8.0 Hz, 1H), 8.40 (d, *J* = 7.9 Hz, 1H), 7.82 (dd, *J* = 8.0, 1.7 Hz, 1H), 7.39 (dd, *J* = 7.9, 1.6 Hz, 1H), 2.34 (s, 3H). Peaks at 4.64 and 1.52 ppm correspond to residual Et_2O coordinated to **3c**.

^{13}C NMR (101 MHz, CD_3CN): δ 149.0, 145.1, 136.8, 136.7, 132.9 (q, *J* = 3.7 Hz), 130.5, 130.4, 129.9, 126.9 (q, *J* = 3.8 Hz), 124.6 (q, *J* = 273.1 Hz), 21.7.

^{19}F NMR (282 MHz, CD_3CN): δ –63.43 (3F), –151.63 (4F).

HRMS (ESI, m/z): calc'd for $C_{14}H_9BiF_3SO_2^+$ $[M-BF_4]^+$ 507.0074; found 507.0081.

Diarylbismuth tetrafluoroborate (3d): Following the protocol described above at 0.20 mmol scale of **5d**, complex **3d** was obtained as a white solid (100 mg, 88% yield).

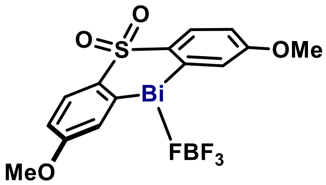

3d

^{1}H NMR (400 MHz, CD_3CN): δ 8.62 (bs, 2H), 8.41 (d, *J* = 8.6 Hz, 2H), 7.01 (dd, *J* = 8.6, 2.5 Hz, 2H), 3.86 (s, 3H). Peaks at 4.67 and 1.52 ppm correspond to residual Et_2O coordinated to **3d**.

^{13}C NMR (101 MHz, CD_3CN): δ 167.7, 131.9, 122.3, 114.3, 56.5.

^{19}F NMR (282 MHz, CD_3CN): δ –151.45.

HRMS (ESI, m/z): calc'd for $C_{14}H_{12}BiSO_4^+$ $[M-BF_4]^+$ 485.0255; found 485.0259.

Diarylbismuth tetrafluoroborate (3e): Following the protocol described above at 0.26 mmol scale of **5e**, complex **3e** was obtained as beige solid (120 mg, 73% yield).

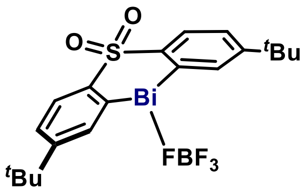

3e

^{1}H NMR (400 MHz, CD_3CN): δ 9.04 (bs, 2H), 8.41 (d, *J* = 8.2 Hz, 2H), 7.59 (dd, *J* = 8.2, 1.8 Hz, 2H), 1.22 (s, 18H). Peaks at 4.65 and 1.51 ppm correspond to residual Et_2O coordinated to **3e**.

^{13}C NMR (101 MHz, CD_3CN): δ 197.8, 161.3, 138.0, 133.6, 130.3, 127.3, 36.6, 31.2.

^{19}F NMR (282 MHz, CD_3CN): δ–151.59.

HRMS (ESI, m/z): calc'd for $C_{20}H_{24}BiSO_2^+$ $[M\text{-}BF_4]^+$ 537.1296; found 537.1303.

*3.3. Synthesis of diarylbismuth tosylate **4***

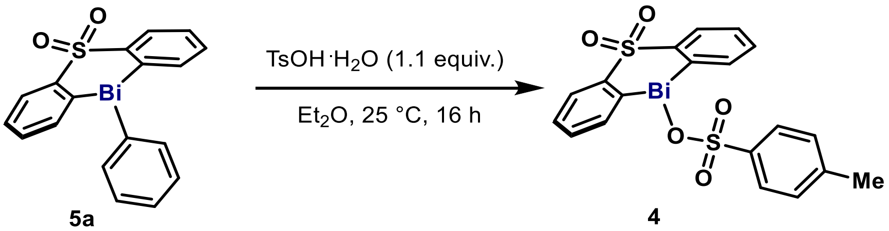

5a **4**

Procedure: Triarylbismuth **5a** (0.56 mmol) and $TsOH{\cdot}H_2O$ (0.61 mmol, 1.1 equiv.) were dissolved in Et_2O (10 mL). The reaction mixture was stirred for 16 h at 25 °C and then, the white precipitate was filtered, washed with Et_2O (2 × 10 mL) and dried under vacuum, obtaining **4** (300 mg, 90% yield) as a white solid. Analytical data for **4** are in agreement with the reported values in the literature.[9]

4. Stoichiometric Optimization

4.1. Optimization of ligand backbone

General Procedure: Triarylbismuth compounds **5a-e** (0.02 mmol) and oxidant (0.03 mmol, 1.5 equiv.) were mixed with $CDCl_3$ (0.7 mL) under argon atmosphere. Then, the Schlenk was pressurized with SO_2 (1.5 bar) and the reaction mixture was stirred at 70 °C for 16 h. After cooling down the reaction to 25 °C, 1,4-difluorobenzene was added as internal standard (0.02 mmol, addition by weight) and the crude reaction was analyzed by ^{1}H and ^{19}F NMR to determine the yield of phenyl sulfonyl fluoride **2a** (see Table S1).

Table S1. Ligand and oxidant screening.

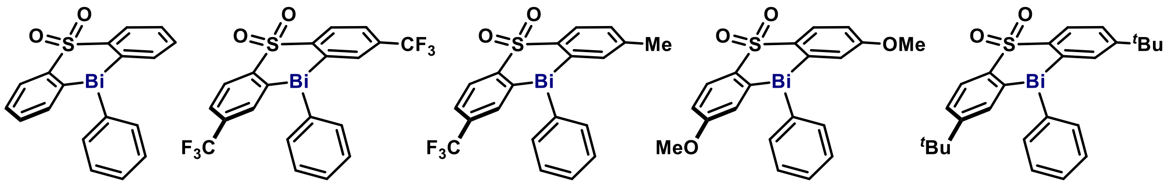

5a **5b** **5c** **5d** **5e**

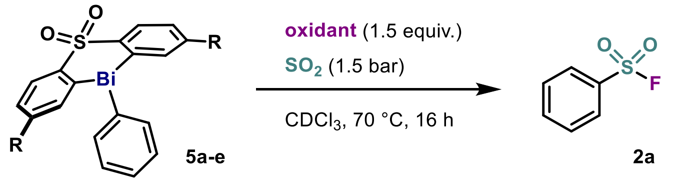

entry	[Bi]	oxidant	yield **2a** (%)[a]
1	**5a**	$[Cl_2PyF]BF_4$	6[b]
2	**5b**	$[Cl_2PyF]BF_4$	10[b]
3	**5c**	$[Cl_2PyF]BF_4$	15[b]
4	**5d**	$[Cl_2PyF]BF_4$	16[b]
5	**5e**	$[Cl_2PyF]BF_4$	12[b]
6	**5a**	NFSI	40
7	**5b**	NFSI	42
8	**5c**	NFSI	55
9	**5d**	NFSI	48
7	**5e**	NFSI	41
6	**5c**	Selectfluor®	70
7	**5d**	Selectfluor®	60

[a]Yields determined by ^{1}H and ^{19}F NMR using 1,4-difluorobenzene as internal standard
[b]Side reactivity of $[Cl_2PyF]BF_4$ was observed (ca. 40-60%)

4.2. Transmetalation step

*4.2.1. Transmetalation of phenyl boronic acid **1a***

Procedure: In a culture tube, diarylbismuth tetrafluoroborate **3c** (0.02 mmol), phenyl boronic acid **1a** (0.02 mmol, 1.0 equiv.), base (0.06 mmol, 3.0 equiv.) and 10 mg of MS were mixed with anhydrous $CDCl_3$ (0.7 mL) under an argon atmosphere and the reaction mixture was stirred for 2 h at 70 °C. After cooling down to 25 °C, 1,3,5-trimethoxybenzene was added as internal standard and the crude reactions were analyzed by ^{1}H NMR to determine the yield of **5c** (see Table S2).

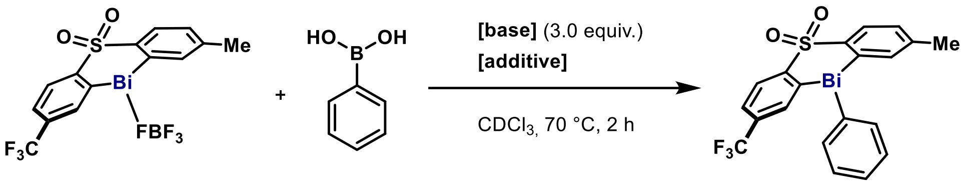

3c **1a** (1.0 eq.) **5c**

Table S2. Transmetalation of phenyl boronic acid **1a**. Yield of **5c** calculated by ^{1}H NMR using 1,3,5-trimethoxybenzene as internal standard.

entry	**base**	**additive**	yield **5c** (%)
1	K_3PO_4	-	41
2	K_2CO_3	-	28
3	Na_3PO_4	-	35
4	K_3PO_4	4 Å MS	97
5	Na_3PO_4	4 Å MS	90
6[a]	K_3PO_4	4 Å MS	76
7[a]	Na_3PO_4	4 Å MS	70

[a]CD_3CN was used as solvent instead of $CDCl_3$

*4.2.2. Transmetalation of benzofuran-3-boronic acid **1x***

Procedure: In a culture tube, diarylbismuth complex (0.02 mmol), benzofuran-3-boronic acid **1x** (0.02 mmol, 1.0 equiv.), base (0.06 mmol, 3 equiv.) and additive were mixed with anhydrous $CDCl_3$ (0.7 mL) under an Ar atmosphere and the reaction was stirred for 2 h at 70 °C. Then, 1,3,5-trimethoxybenzene was added as internal standard and the crude reaction was analyzed by ^{1}H NMR to determine the yield of diaryl(heteroaryl)bismuth (see Table S3).

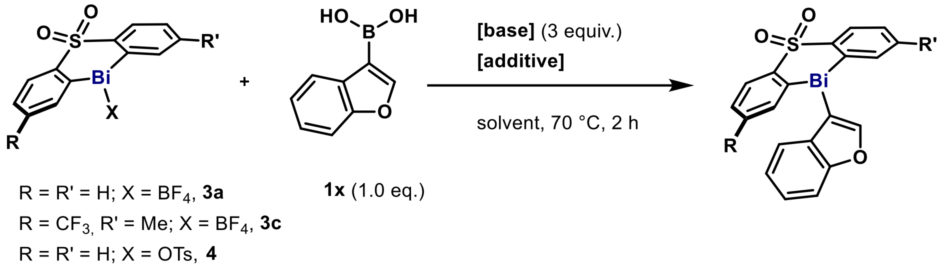

Table S3. Transmetalation of benzofuran-3-boronic acid **1x**. Yield calculated by ^{1}H NMR using 1,3,5-trimethoxybenzene as internal standard.

entry	**Bi**	**base**	**additive**	yield (%)
1	**3c**	K_3PO_4	4 Å MS	<5
2	**3c**	K_2CO_3	4 Å MS	<5
3	**3a**	K_3PO_4	4 Å MS	<5
4	**3a**	K_2CO_3	4 Å MS	<5
5	**4**	K_3PO_4	4 Å MS	10
6	**4**	K_2CO_3	4 Å MS	8
7	**3c**	K_2CO_3	H_2O (5%)	25
8	**3a**	K_2CO_3	H_2O (5%)	31
9	**4**	K_2CO_3	H_2O (5%)	95

10-(Benzofuran-3-yl)-10*H*-dibenzo[*b*,*e*][1,4]thiabismine 5,5-dioxide (9): Following the protocol described above (conditions Table S3, entry 9) at 0.3 mmol scale of **4**, bismuth compound **9** (150 mg, 93% yield) was obtained as white solid after the removal of solvent under vacuum.

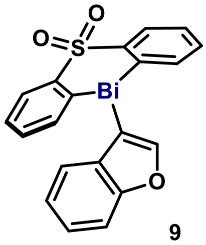
9

^{1}H NMR (600 MHz, $CDCl_3$): δ 8.42 (ddd, *J* = 7.8, 1.3, 0.5 Hz, 2H), 7.95 (ddd, *J* = 7.3, 1.2, 0.5 Hz, 2H), 7.71 (s, 1H), 7.58 (dt, *J* = 8.2, 0.8 Hz, 1H), 7.40 (ddd, *J* = 7.4, 1.2 Hz, 2H), 7.27 – 7.23 (m, 2H), 7.19 – 7.16 (m, 1H), 6.79 (ddd, *J* = 8.1, 7.2, 1.0 Hz, 1H), 6.18 (d, *J* = 7.8 Hz, 1H).

^{13}C NMR (151 MHz, $CDCl_3$): δ 157.0, 154.2, 152.6, 141.9, 138.2, 133.3, 133.0, 128.6, 127.3, 124.5, 123.2, 122.5, 111.5.

HRMS (ESI, m/z): calc'd for $C_{20}H_{13}BiSO_3Na^+$ $[M+Na]^+$ 565.0282; found 565.0282.

4.3. SO_2-insertion step

Procedure: In a pressure Schlenk, bismuth compounds **5c** or **9** (0.1 mmol), were dissolved in anhydrous $CDCl_3$ (1.5 mL) or in $CDCl_3$:H_2O (5%). Then, the Schlenk was pressurized with SO_2 (1.5 bar) and the reaction mixture was stirred for 2 h at 70 °C. Finally, the solvent was evaporated under vacuum (first dried over $MgSO_4$ in case of b) and the solids were washed with dry pentane to yield the corresponding bismuth-sulfinate **7** and **10** in quantitative yield as off-white solids.

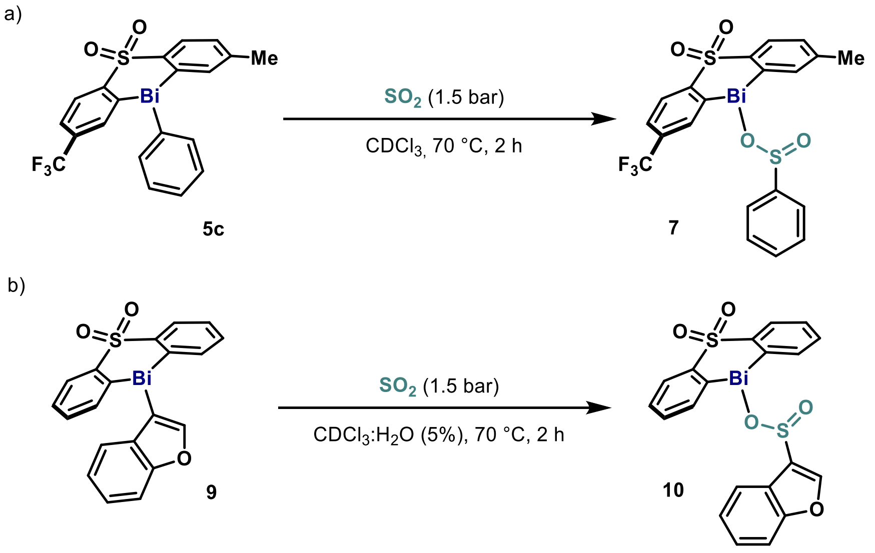

2-Methyl-5,5-dioxido-8-(trifluoromethyl)-10H-dibenzo[b,e][1,4]thiabismin-10-yl benzenesulfinate (7): Following the protocol described above (a), bismuth sulfinate **7** was obtained as off-white solid (93% yield, 60 mg).

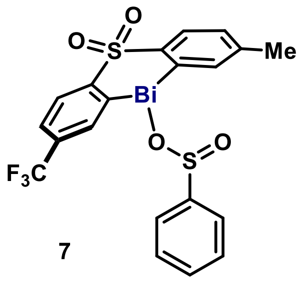

^{1}H NMR (400 MHz, $CDCl_3$): δ 8.84 (bs, 1H), 8.54 (bs, 1H), 8.38 (d, J = 7.9 Hz, 1H), 8.26 (d, J = 7.9 Hz, 1H), 7.88 – 7.86 (m, 2H), 7.69 – 7.66 (m, 1H), 7.61 – 7.58 (m, 3H), 7.27 – 7.24 (m, 1H), 2.40 (s, 3H).

^{13}C NMR (101 MHz, $CDCl_3$): δ 185.1, 153.0, 147.3, 144.6, 137.0, 136.7, 136.5, 136.4, 133.3 (d, J = 3.9 Hz), 131.6, 129.6, 129.4 (d, J = 2.4 Hz), 128.9, 128.5, 126.3, 125.9 (q, J = 3.8 Hz), 124.5, 123.5 (q, J = 273.9 Hz), 22.0.

^{19}F NMR (282 MHz, $CDCl_3$) δ –62.73.

HRMS (APCI, m/z): calc'd for $C_{20}H_{15}BiF_3S_2O_4$ $[M+H]^+$ 649.0162; found 649.0166.

Single crystal X-ray: Quality crystals for X-ray diffraction of Bi-sulfinate **7** were obtained by vapor transfer of pentane into a solution of **7** in $CDCl_3$.

5,5-Dioxido-10H-dibenzo[b,e][1,4]thiabismin-10-yl benzofuran-3-sulfinate (10): Following the protocol described above (b), bismuth sulfinate **10** was obtained as an off-white solid (91% yield, 55 mg)

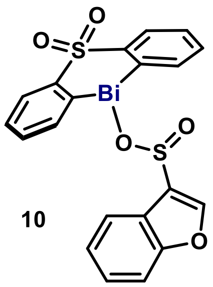

10

^{1}H NMR (400 MHz, $CDCl_3$): δ 8.76 (d, *J* = 7.1 Hz, 2H), 8.33 (dd, *J* = 7.7, 1.3 Hz, 2H), 8.03 (s, 1H), 7.97 – 7.94 (m, 1H), 7.68 (td, *J* = 7.4, 1.2 Hz, 2H), 7.62 – 7.59 (m, 1H), 7.46 – 7.40 (m, 4H).

^{13}C NMR (101 MHz, $CDCl_3$): δ 183.7, 156.0, 145.8, 144.9, 140.6, 136.4, 135.4, 128.8, 128.7, 125.8, 124.0, 123.6, 122.7, 121.2, 120.5, 112.4, 111.4, 106.6.

HRMS (APCI, m/z): calc'd for $C_{20}H_{14}BiS_2O_5$ $[M+H]^+$ 607.0081; found 607.0077.

4.4. Bi-sulfinate oxidation

Procedure: Bismuth sulfinate **7** or **10** (0.02 mmol) and oxidant (0.03 mmol, 1.5 equiv.) were dissolved in anhydrous $CDCl_3$ (0.7 mL) or in $CDCl_3$:H_2O (5%) and the reaction was stirred for 2 h at 70 °C. Then, 1,4-difluorobenzene was added as internal standard and the crude reactions were analyzed by ^1H and ^{19}F NMR to determine the yield of the corresponding aryl sulfonyl fluorides (see Table S4).

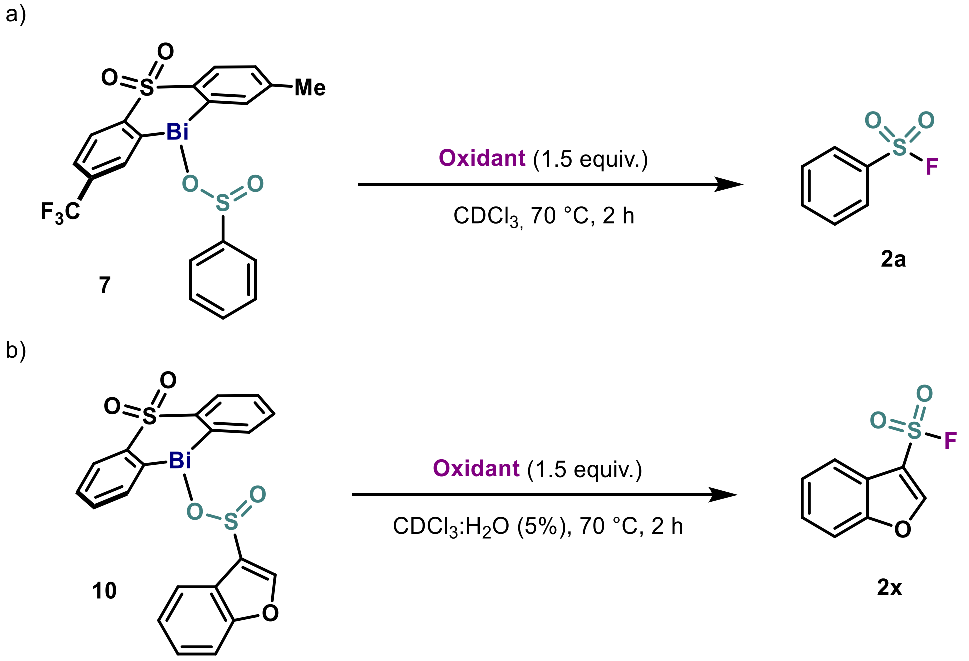

Table S4. Bi-sulfinate oxidation. Yield of **2a** and **2x** calculated by ^1H and ^{19}F NMR using 1,4-difluorobenzene as internal standard.

entry	[Bi]	oxidant	yield of **2** (%)
1	**7**	NFSI	75
2	**7**	Selectfluor®	92
3	**10**	NFSI	48
4	**10**	Selectfluor®	<5

5. Catalytic studies

*5.1. Optimization for the Bi-catalyzed synthesis of phenyl sulfonyl fluoride **2a**.*

Procedure: In a glovebox under nitrogen, a pressure Schlenk equipped with a stirring bar was charged with phenyl boronic acid **1a** (0.05 mmol), bismuth catalyst (10-5 mol%), base (3.0-2.0 equiv.), oxidant (1.5 equiv.) and 4 Å molecular sieves (10 mg) were dissolved in dry solvent (0.5 mL). Then, the Schlenk was pressurized with SO_2 (1.5 bar) and left stirring for 16 h at 70 °C. After the reaction time, the reaction was cooled to 25 °C, 1,4-difluorobenzene was added as internal standard and the crude was filtered through Celite®. The crude mixture was analyzed by ^{1}H and ^{19}F NMR to determine the yield of **2a** (see Table 5).

Table S5. Optimization of reaction conditions for the Bi-catalyzed formation of phenyl sulfonyl fluoride **2a**.

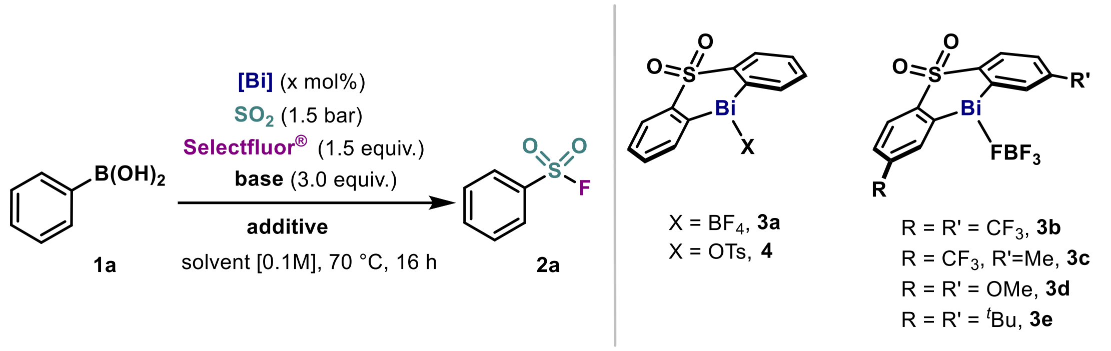

entry	base (equiv.)	catalyst (x mol%)	additive	solvent	F^+ source	**2a,** yield (%)[a]
1	K_3PO_4 (3)	**3a** (10)	4 Å MS	$CDCl_3$	Selectfluor®	49
2	K_3PO_4 (3)	**3b** (10)	4 Å MS	$CDCl_3$	Selectfluor®	55
3	K_3PO_4 (3)	**3c** (10)	4 Å MS	$CDCl_3$	Selectfluor®	77
4	K_3PO_4 (3)	**3d** (10)	4 Å MS	$CDCl_3$	Selectfluor®	61
5	K_3PO_4 (3)	**3e** (10)	4 Å MS	$CDCl_3$	Selectfluor®	66
6	K_3PO_4 (3)	**4** (10)	4 Å MS	$CDCl_3$	Selectfluor®	62
7	K_3PO_4 (3)	**3c** (10)	4 Å MS	$CDCl_3$	Selectfluor®	77
8	K_3PO_4 (3)	**3c** (10)	4 Å MS	$CDCl_3$	NFSI	61
9[b]	K_3PO_4 (3)	**3c** (10)	4 Å MS	$CDCl_3$	Selectfluor®	41
10[c]	K_3PO_4 (3)	**3c** (10)	4 Å MS	$CDCl_3$	Selectfluor®	88
11[d]	K_3PO_4 (3)	**3c** (10)	4 Å MS	$CDCl_3$	Selectfluor®	98
12[d]	Na_3PO_4 (3)	**3c** (10)	4 Å MS	$CDCl_3$	Selectfluor®	85
13[d]	Na_2CO_3 (3)	**3c** (10)	4 Å MS	$CDCl_3$	Selectfluor®	80
14[d]	K_3PO_4 (2)	**3c** (10)	4 Å MS	$CDCl_3$	Selectfluor®	72
15[d,e]	K_3PO_4 (3)	**3c** (10)	4 Å MS	$CDCl_3$	Selectfluor®	15
16[d,f]	K_3PO_4 (3)	**3c** (10)	4 Å MS	$CDCl_3$	Selectfluor®	n.d.
17[d]	-	**3c** (10)	4 Å MS	$CDCl_3$	Selectfluor®	34
18[d]	K_3PO_4 (3)	**3c** (10)	-	$CDCl_3$	Selectfluor®	28
19[d]	K_3PO_4 (3)	**3c** (5)	4 Å MS	$CDCl_3$	Selectfluor®	95 (91)
20[d]	K_3PO_4 (3)	**3c** (2.5)	4 Å MS	$CDCl_3$	Selectfluor®	44
21[d]	K_3PO_4 (3)	-	4 Å MS	$CDCl_3$	Selectfluor®	n.d.

[a]Yields determined by ^1H and ^{19}F NMR yield using 1,4-difluorobenzene as internal standard. In parenthesis, yield of product **2a** at 0.2 mmol scale.
[b]$CDCl_3$:CD_3CN 1:1 mixture.
[c]$CDCl_3$:CD_3CN 3:1 mixture.
[d]$CDCl_3$:CD_3CN 5:1 mixture.
[e]DABSO (1.5 equiv.) was used as SO_2 source.
[f]$Na_2S_2O_5$ (3.0 equiv.) was used as SO_2 source.

5.2. Optimization of the Bi-catalyzed synthesis of benzofuran-3-sulfonyl fluoride ***2x****.*

Procedure: A pressure Schlenk equipped with a stirring bar was charged with heteroaryl boronic acid **1x** (0.02 mmol), diarylbismuth tosylate **4** (10–5 mol%), K_2CO_3 (2–3 equiv.) and NFSI (1.5 equiv.). Then, dry $CHCl_3$ (0.5 mL) was added followed by H_2O (10–1%). The Schlenk was pressurized with SO_2 (1.5 bar) and left stirring for 16 h at 60 °C. After the reaction time, the crude reaction was cooled to 25 °C, 1,4-difluorobenzene was added as internal standard and the crude mixture was filtered through $MgSO_4$. ^1H and ^{19}F NMR were used to determine the yield of **2x** (see Table 6).

Table S6. Optimization of reaction conditions for the Bi-catalyzed synthesis of benzofuran-3-sulfonyl fluoride **2x.**

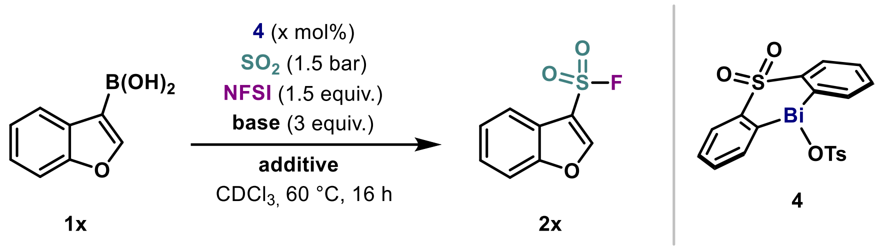

entry	base	**4** (x mol%)	additive (%)	**2x**, yield (%)[a]
1	K_2CO_3 (3)	**4** (10)	H_2O (5)	88
2	K_2CO_3 (3)	**4** (10)	H_2O (1)	55
3	K_2CO_3 (3)	**4** (10)	H_2O (10)	81
4	K_2CO_3 (2)	**4** (10)	H_2O (5)	60
5	-	**4** (10)	H_2O (5)	22
6	K_2CO_3 (3)	**4** (10)	-	15
7	K_2CO_3 (3)	**4** (5)	H_2O (5)	61
8	K_2CO_3 (3)	-	H_2O (5)	n.d.

[a]Yields determined by ^1H and ^{19}F NMR using 1,4-difluorobenzene as internal standard.

5.3. Scope of Bi-catalyzed synthesis of sulfonyl fluorides

5.3.1. General procedure for aryl sulfonyl fluorides

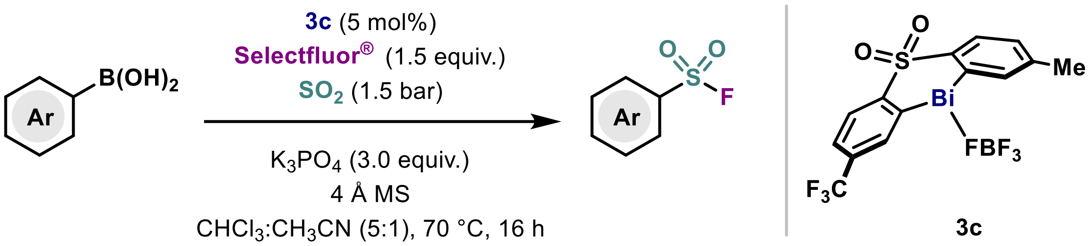

General procedure A: A pressure Schlenk equipped with a stirring bar was charged with aryl boronic acid (0.2 mmol), diarylbismuth tetrafluoroborate **3c** (5 mol%, 5.9 mg), K_3PO_4 (3.0 equiv., 0.6 mmol, 128 mg) and Selectfluor® (1.5 equiv., 106 mg), 4Å molecular sieves (50 mg) and dry $CHCl_3$:CH_3CN (5:1, 3 mL). The Schlenk was pressurized with SO_2 (1.5 bar) and left stirring for 16 h at 70 °C. After the reaction time, the reaction was cooled to 25 °C and filtered, the solid was washed with CH_2Cl_2 (2 × 3 mL) and the volatiles evaporated under vacuum. The crude material was purified by flash chromatography (SiO_2, pentane:CH_2Cl_2 as eluent system).

5.3.1 General procedure for heteroaryl sulfonyl fluorides

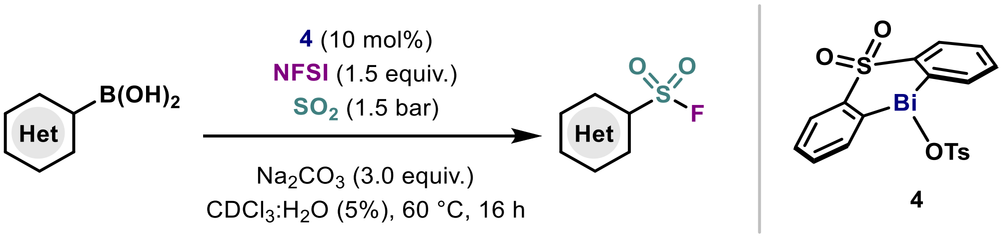

General procedure B: A pressure Schlenk equipped with a stirring bar was charged with heteroaryl boronic acid (0.17 mmol), diarylbismuth tosylate **4** (10 mol%, 10 mg), Na_2CO_3 (3.0 equiv., 0.5 mmol, 69 mg) and NFSI (1.5 equiv., 79 mg). Then, dry $CHCl_3$ (2 mL) was added followed by 100 µL of H_2O (5%). The Schlenk was pressurized with SO_2 (1.5 bar) and left stirring for 16 h at 60 °C. After the reaction time, the reaction was cooled to 25 °C and dried over $MgSO_4$, washed with CH_2Cl_2 (2 × 3 mL) and the volatiles evaporated under vacuum. The crude material was purified by flash chromatography (SiO_2, pentane:CH_2Cl_2 as eluent system).

Table S7. Scope of the Bi-catalyzed synthesis or (hetero)aryl-sulfonyl fluorides **2a-2ac.**

Electronics and sterics

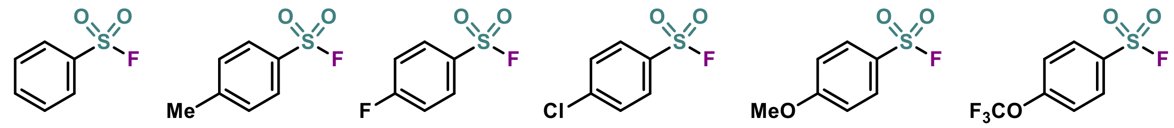

2a, 91% **2b**, 83% **2c**, 78% **2d**, 76% **2e**, 36% **2f**, 53%

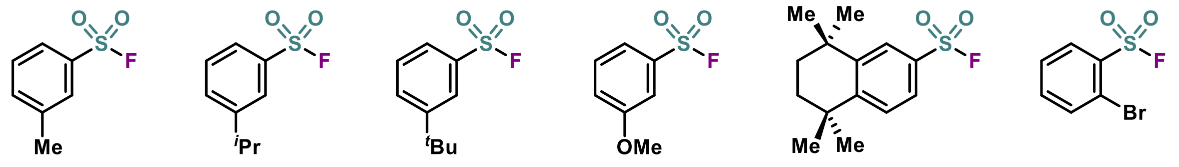

2g, 66% **2h**, 85% **2i**, 72% **2j**, 58% **2k**, 79% **2l**, 59%

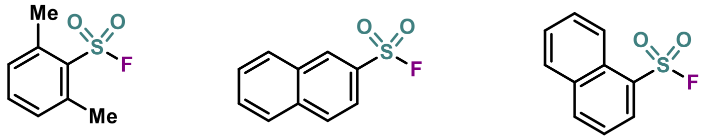

2m, 69% **2n**, 93% **2o**, 91%

Functional group tolerance

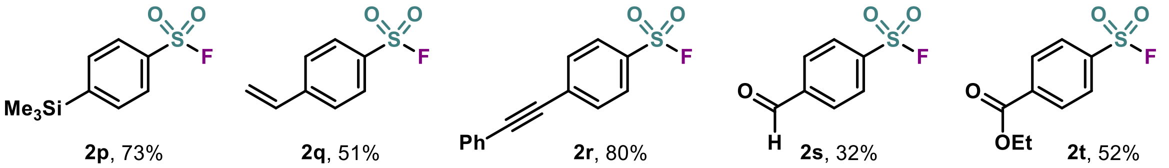

2p, 73% **2q**, 51% **2r**, 80% **2s**, 32% **2t**, 52%

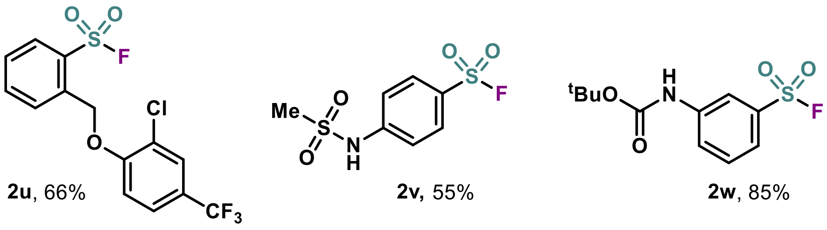

2u, 66% **2v,** 55% **2w**, 85%

Heterocycles

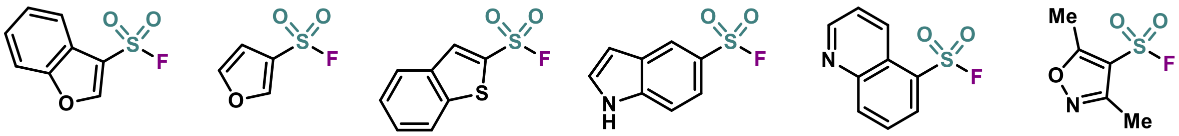

2x, 86% **2y**, 90% **2z**, 63% **2aa**, 45% **2ab**, 62% **2ac**, 68%

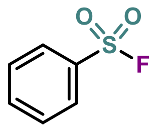

2a

Benzenesulfonyl fluoride (2a):[10] Following the general procedure A, **2a** was obtained in 91% yield as colorless oil after a flash column chromatography (Eluent: pentane:CH_2Cl_2 8:2).

^{1}H NMR (600 MHz, $CDCl_3$): δ 8.04 – 8.02 (m, 2H), 7.80 – 7.77 (m, 1H), 7.66 – 7.63 (m, 2H).

^{13}C NMR (151 MHz, $CDCl_3$): δ 135.5, 133.2 (d, J = 24.3 Hz), 129.7, 128.4.

^{19}F NMR (565 MHz, $CDCl_3$): δ 65.84.

HRMS (EI, m/z): calc'd for $C_6H_5FO_2S$ $[M]^+$ 159.9988; found 159.9990.

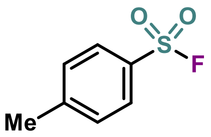

2b

4-Methylbenzenesulfonyl fluoride (2b):[10] Following the general procedure A, **2b** was obtained in 83% yield as colorless oil after a flash column chromatography (Eluent: pentane:CH_2Cl_2 8:2).

^{1}H NMR (600 MHz, $CDCl_3$): δ 7.90 (d, J = 8.4 Hz, 2H), 7.42 (dt, J = 8.7, 0.8 Hz, 2H), 2.49 (s, 3H).

^{13}C NMR (151 MHz, $CDCl_3$): δ 147.0, 130.2, 130.1 (d, J = 24.1 Hz), 128.5, 21.8.

^{19}F NMR (565 MHz, $CDCl_3$): δ 68.14.

HRMS (EI, m/z): calc'd for $C_7H_7FO_2S$ $[M]^+$ 174.0145; found 174.0145.

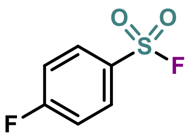

2c

4-Fluorobenzenesulfonyl fluoride (2c):[10] Following the general procedure A, **2c** was obtained in 78% yield as colorless oil after a flash column chromatography (Eluent: pentane:CH_2Cl_2 8:2).

^{1}H NMR (600 MHz, $CDCl_3$): δ 8.08 – 8.04 (m, 2H), 7.34 – 7.30 (m, 2H).

^{13}C NMR (151 MHz, $CDCl_3$): δ 166.8 (d, J = 260.0 Hz), 131.5 (d, J = 10.1 Hz), 129.0 (dd, J = 25.8, 3.3 Hz), 117.2 (d, J = 23.1 Hz).

^{19}F NMR (565 MHz, $CDCl_3$): δ 66.74 (1F), –99.36 (1F).

HRMS (EI, m/z): calc'd for $C_6H_4F_2O_2S$ $[M]^+$ 177.9895; found 177.9896.

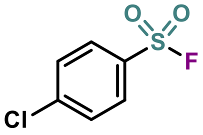

2d

4-Chlorobenzenesulfonyl fluoride (2d):[10] Following the general procedure A, **2d** was obtained in 76% yield as colorless oil after a flash column chromatography (Eluent: pentane:CH_2Cl_2 8:2).

^{1}H NMR (600 MHz, $CDCl_3$): δ 7.97 – 7.95 (m, 2H), 7.63 – 7.61 (m, 2H).

^{13}C NMR (151 MHz, $CDCl_3$): δ 142.7, 131.4 (d, J = 25.8 Hz), 130.1, 129.9.

^{19}F NMR (565 MHz, $CDCl_3$): δ 66.45.

HRMS (EI, m/z): calc'd for $C_6H_4FO_2SCl$ $[M]^+$ 193.9599; found 193.9601.

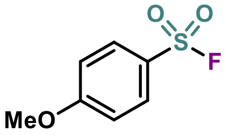

2e

4-Methoxybenzenesulfonyl fluoride (2e):[10] Following the general procedure A, **2e** was obtained in 36% yield as colorless oil after a flash column chromatography (Eluent: pentane:CH_2Cl_2 7:3).

^{1}H NMR (600 MHz, $CDCl_3$): δ 7.96 – 7.93 (m, 2H), 7.08 – 7.05 (m, 2H), 3.92 (s, 3H).

^{13}C NMR (151 MHz, $CDCl_3$): δ 165.2, 130.9, 124.2 (d, J = 24.8 Hz), 114.9, 55.9.

^{19}F NMR (565 MHz, $CDCl_3$): δ 67.24.

HRMS (EI, m/z): calc'd for $C_7H_7FO_3S$ $[M]^+$ 190.0094; found 190.0097.

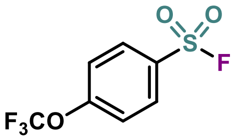

2f

4-(Trifluoromethoxy)benzenesulfonyl fluoride (2f):[11] Following the general procedure A, **2f** was obtained in 53% yield as colorless oil after a flash column chromatography (Eluent: pentane:CH_2Cl_2 8:2).

^{1}H NMR (600 MHz, $CDCl_3$): δ 8.11 – 8.08 (m, 2H), 7.47 – 7.44 (m, 2H).

^{13}C NMR (151 MHz, $CDCl_3$): δ 154.3 (q, J = 1.9 Hz), 130.9, 121.2 (d, J = 1.5 Hz), 120.1 (q, J = 260.8 Hz).

^{19}F NMR (565 MHz, $CDCl_3$): δ 66.54 (1F), –57.72 (3F).

HRMS (EI, m/z): calc'd for $C_7H_4F_4O_3S$ $[M]^+$ 243.9812; found 243.9815.

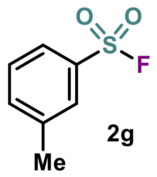

2g

3-Methylbenzenesulfonyl fluoride (2g):[10] Following the general procedure A, **2g** was obtained in 66% yield as colorless oil after a flash column chromatography (Eluent: pentane:CH_2Cl_2 8:2).

^{1}H NMR (600 MHz, $CDCl_3$): δ 7.85 – 7.83 (m, 2H), 7.61 – 7.59 (m, 1H), 7.55 – 7.52 (m, 1H), 2.51 (s, 3H).

^{13}C NMR (151 MHz, $CDCl_3$): δ 140.2, 136.32, 132.9 (d, J = 23.7 Hz), 129.5, 128.6, 125.6, 21.3.

^{19}F NMR (565 MHz, $CDCl_3$): δ 67.67.

HRMS (EI, m/z): calc'd for $C_7H_7FO_2S$ $[M]^+$ 174.0145; found 174.0149.

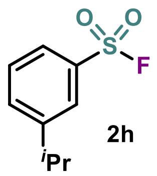

2h

3-Isopropylbenzenesulfonyl fluoride (2h): Following the general procedure A, **2h** was obtained in 85% yield as colorless oil after a flash column chromatography (Eluent: pentane:CH_2Cl_2 8:2).

^{1}H NMR (600 MHz, $CDCl_3$): δ 7.85 (t, J = 1.9 Hz, 1H), 7.84 – 7.83 (m, 1H), 7.63 (d, J = 7.8 Hz, 1H), 7.55 (t, J = 7.7, 1H), 3.04 (p, J = 6.9 Hz, 2H), 1.30 (d, J = 7.0 Hz, 6H).

^{13}C NMR (151 MHz, $CDCl_3$): δ 151.1, 133.9, 133.0 (d, J = 23.6 Hz), 129.6, 126.2, 125.9, 34.1, 23.6.

^{19}F NMR (565 MHz, $CDCl_3$): δ 65.80.

HRMS (EI, m/z): calc'd for $C_9H_{11}FO_2S$ $[M]^+$ 202.0458; found 202.0461.

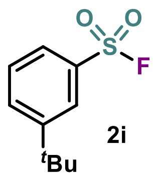

2i

3-(*tert*-Butyl)benzenesulfonyl fluoride (2i): Following the general procedure A, **2i** was obtained in 72% yield as colorless oil after a flash column chromatography (Eluent: pentane:CH_2Cl_2 8:2).

^{1}H NMR (600 MHz, $CDCl_3$): δ 8.00 (t, J = 7.8 Hz, 1H), 7.83 (dd, J = 7.8, 1.8, 0.5 Hz, 1H), 7.80 (ddd, J = 8.0, 1.8, 0.6 Hz, 1H), 7.56 (tdd, 7.9, 1.1, 0.5 Hz, 1H), 1.37 (s, 9H).

^{13}C NMR (151 MHz, $CDCl_3$): δ 153.5, 132.9 (d, J = 24.0 Hz), 132.8, 129.4, 125.6, 125.1, 35.2, 31.0

^{19}F NMR (565 MHz, $CDCl_3$): δ 65.83.

HRMS (EI, m/z): calc'd for $C_{10}H_{13}FO_2S$ $[M]^+$ 216.0615; found 216.0616.

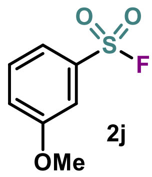

2j

3-Methoxybenzenesulfonyl fluoride (2j):[10] Following the general procedure A, **2j** was obtained in 58% yield as yellowish oil after a flash column chromatography (Eluent: pentane:CH_2Cl_2 8:2).

^{1}H NMR (600 MHz, $CDCl_3$): δ 7.60 (ddd, J = 7.8, 1.7, 0.6 Hz, 1H), 7.53 (tdd, J = 7.8, 1.2, 0.4 Hz, 1H), 7.47 (dd, J = 2.6, 1.8 Hz, 1H), 7.28 (ddt, J = 8.4, 2.6, 0.9 Hz, 1H), 3.89 (s, 3H).

^{13}C NMR (151 MHz, $CDCl_3$): δ 160.2, 134.0 (d, J = 24.2 Hz), 130.7, 122.2, 120.6, 112.6, 55.9.

^{19}F NMR (565 MHz, $CDCl_3$): δ 65.56.

HRMS (EI, m/z): calc'd for $C_7H_7FO_3S$ $[M]^+$ 190.0094; found 190.0094.

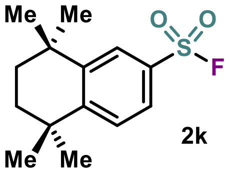

2k

5,5,8,8-tetramethyl-5,6,7,8-tetrahydronaphthalene-2-sulfonyl fluoride (2k): Following the general procedure A, **2k** was obtained in 79% yield as an off-white solid after a flash column chromatography (Eluent: pentane:CH_2Cl_2 8:2).

^{1}H NMR (600 MHz, $CDCl_3$): δ 7.91 (d, J = 2.1 Hz, 1H), 7.72 (dd, J = 8.8, 2.1 Hz, 1H), 7.53 (dd, J = 8.4, 0.8 Hz, 1H), 1.73 (s, 4H), 1.32 (s, 6H), 1.31 (s, 6H).

^{13}C NMR (151 MHz, $CDCl_3$): δ 153.8, 147.2, 123.0 (d, J = 23.4 Hz), 128.2, 127.0, 125.1, 35.0, 34.8, 34.4, 34.3, 31.6, 31.5.

^{19}F NMR (565 MHz, $CDCl_3$): δ 66.01.

HRMS (EI, m/z): calc'd for $C_{14}H_{19}FO_2S$ $[M]^+$ 270.1084; found 270.1084.

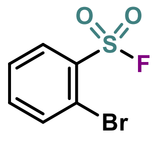

2l

2-Bromobenzenesulfonyl fluoride (2l):[12] Following the general procedure A, **2l** was obtained in 59% yield as a white solid after a flash column chromatography (Eluent: pentane:CH_2Cl_2 8:2).

^{1}H NMR (600 MHz, $CDCl_3$): δ 8.15 – 8.13 (m, 1H), 7.86 – 7.84 (m, 1H), 7.60 – 7.54 (m, 2H).

^{13}C NMR (151 MHz, $CDCl_3$): δ 136.1, 135.9, 134.0 (d, J = 24.3 Hz), 132.1 (d, J = 1.7 Hz), 127.9, 121.1 (d, J = 1.3 Hz).

^{19}F NMR (565 MHz, $CDCl_3$): δ 57.9.

HRMS (EI, m/z): calc'd for $C_6H_4FO_2SBr$ $[M]^+$ 237.9094; found 237.9098.

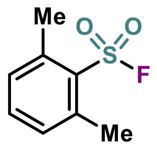

2m

2,6-Dimethylbenzenesulfonyl fluoride (2m): Following the general procedure A, **2m** was obtained in 69% yield as a white crystalline solid after a flash column chromatography (Eluent: pentane:CH_2Cl_2 8:2).

^{1}H NMR (600 MHz, $CDCl_3$): δ 7.43 (t, *J* = 7.7 Hz, 1H), 7.23 (dd, *J* = 7.7, 0.8 Hz, 2H), 2.69 (s, 6H).

^{13}C NMR (151 MHz, $CDCl_3$): δ 140.1, 134.0, 132.1 (d, *J* = 20.1 Hz), 131.1, 131.0, 22.5.

^{19}F NMR (565 MHz, $CDCl_3$): δ 67.83.

HRMS (EI, m/z): calc'd for $C_8H_9FO_2S$ $[M]^+$ 188.0301; found 188.0305.

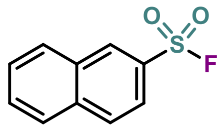

2n

Naphthalene-2-sulfonyl fluoride (2n):[12] Following the general procedure A, **2n** was obtained in 93% yield as a beige solid after a flash column chromatography (Eluent: pentane:CH_2Cl_2 8:2).

^{1}H NMR (600 MHz, $CDCl_3$): δ 8.62 (dd, *J* = 1.4, 0.6 Hz, 1H), 8.07 (dt, *J* = 8.7, 0.7 Hz, 1H), 8.04 (ddd, *J* = 8.2, 1.3, 0.7 Hz, 1H), 7.98 (dq, *J* = 8.3, 0.6 Hz, 1H), 7.94 (dd, *J* = 8.7, 1.9 Hz, 1H), 7.76 (ddd, *J* = 8.2, 6.9, 1.3 Hz, 1H), 7.70 (ddd, *J* = 8.2, 6.9, 1.2 Hz, 1H).

^{13}C NMR (151 MHz, $CDCl_3$): δ 136.0, 131.8, 130.9 (d, *J* = 1.2 Hz), 130.3, 130.1, 129.8 (d, *J* = 24.4 Hz), 129.6, 128.3, 128.1, 122.2.

^{19}F NMR (565 MHz, $CDCl_3$): δ 66.27.

HRMS (EI, m/z): calc'd for $C_{10}H_7FO_2S$ $[M]^+$ 210.0145; found 210.0147

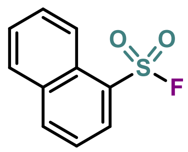

2o

Naphthalene-1-sulfonyl fluoride (2o):[10] Following the general procedure A, **2o** was obtained in 91% yield as a beige solid after a flash column chromatography (Eluent: pentane:CH_2Cl_2 8:2).

^{1}H NMR (600 MHz, $CDCl_3$): δ 8.56 – 8.54 (m, 1H), 8.37 (dt, *J* = 7.4, 1.0 Hz, 1H), 8.24 (dt, *J* = 8.4, 1.1 Hz, 1H), 8.01 – 7.99 (m, 1H), 7.78 (ddd, *J* = 8.5, 6.9, 1.4 Hz, 1H), 7.69 (ddd, *J* = 8.1, 6.9, 1.1 Hz, 1H), 7.62 (ddd, *J* = 8.2, 7.4, 1.5 Hz, 1H).

^{13}C NMR (151 MHz, $CDCl_3$): δ 136.9, 134.0, 131.0, 129.5, 129.2 (d, *J* = 23.3 Hz), 129.0, 128.3, 127.7, 124.1, 124.0.

^{19}F NMR (565 MHz, $CDCl_3$): δ 62.60.

HRMS (EI, m/z): calc'd for $C_{10}H_7FO_2S$ $[M]^+$ 210.0145; found 210.0148.

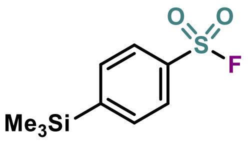

2p

4-(Trimethylsilyl)benzenesulfonyl fluoride (2p):[10] Following the general procedure A, **2p** was obtained in 73% yield as colorless oil after a flash column chromatography (Eluent: pentane:CH_2Cl_2 8:2).

^{1}H NMR (600 MHz, $CDCl_3$): δ 7.96 – 7.95 (m, 2H), 7.77 – 7.75 (m, 2H), 0.33 (s, 9H).

^{13}C NMR (151 MHz, $CDCl_3$): δ 151.2, 134.3, 133.1 (d, *J* = 24.0 Hz), 127.1, -1.5.

^{19}F NMR (565 MHz, $CDCl_3$): δ 65.79.

HRMS (ESI, m/z): calc'd for $C_9H_{13}FO_2SSiNa$ $[M+Na]^+$ 255.0282; found 255.0282.

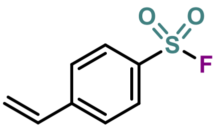

2q

4-Vinylbenzenesulfonyl fluoride (2q): Following the general procedure A, **2q** was obtained in 51% yield as yellowish oil after a flash column chromatography (Eluent: pentane:CH_2Cl_2 8:2).

^{1}H NMR (600 MHz, $CDCl_3$): δ 7.98 – 7.95 (m, 2H), 7.63 – 7.61 (m, 2H), 6.81 – 6.76 (m, 1H), 5.97 (d, *J* = 17.6 Hz, 1H), 5.54 (d, *J* = 10.9 Hz, 1H).

^{13}C NMR (151 MHz, $CDCl_3$): δ 144.9, 134.9, 131.7 (d, *J* = 24.6 Hz), 128.9, 127.3, 119.5.

^{19}F NMR (565 MHz, $CDCl_3$): δ 66.24.

HRMS (EI, m/z): calc'd for $C_8H_7FO_2S$ $[M]^+$ 186.0145; found 186.0149.

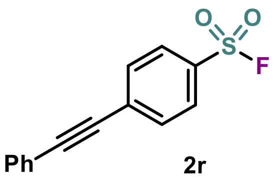

2r

4-(Phenylethynyl)benzenesulfonyl fluoride (2r): Following the general procedure A, **2r** was obtained in 80% yield as an off-white solid after a flash column chromatography (Eluent: pentane:CH_2Cl_2 8:2).

^{1}H NMR (600 MHz, $CDCl_3$): δ 8.00 – 7.98 (m, 2H), 7.75 – 7.73 (m, 2H), 7.57 – 7.56 (m, 2H), 7.42 – 7.38 (m, 3H).

^{13}C NMR (151 MHz, $CDCl_3$): δ 132.4, 131.9, 131.7 (d, *J* = 25.0 Hz), 131.3, 129.4, 128.6, 128.4, 121.9, 95.0, 87.2.

^{19}F NMR (565 MHz, $CDCl_3$): δ 66.27.

HRMS (EI, m/z): calc'd for $C_{14}H_9FO_2S$ $[M]^+$ 260.0302; found 260.0306.

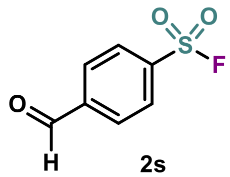

2s

4-Formylbenzenesulfonyl fluoride (2s):[13] Following the general procedure A, **2s** was obtained in 32% yield as yellowish oil after a flash column chromatography (Eluent: pentane:CH_2Cl_2 8:2).

^{1}H NMR (600 MHz, $CDCl_3$): δ 10.17 (s, 1H), 8.22 – 8.20 (m, 2H), 8.15 – 8.13 (m, 2H).

^{13}C NMR (151 MHz, $CDCl_3$): δ 190.2, 140.9, 137.9 (d, *J* = 25.7 Hz), 130.5, 129.4.

^{19}F NMR (565 MHz, $CDCl_3$): δ 65.83.

HRMS (EI, m/z): calc'd for $C_7H_5FO_3S$ $[M]^+$ 187.9937; found 187.9937.

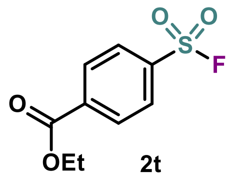

2t

Ethyl 4-(fluorosulfonyl)benzoate (2t):[11] Following the general procedure A, **2t** was obtained in 52% yield as a white solid after a flash column chromatography (Eluent: pentane:CH_2Cl_2 8:2).

^{1}H NMR (600 MHz, $CDCl_3$): δ 8.29 – 8.27 (m, 2H), 8.10 – 8.08 (m, 2H), 4.45 (q, *J* = 7.1 Hz, 2H), 1.43 (t, *J* = 7.1 Hz, 3H).

^{13}C NMR (151 MHz, $CDCl_3$): δ 164.4, 136.9, 136.6 (d, *J* = 25.3 Hz), 130.7, 128.5, 62.1, 14.2.

^{19}F NMR (565 MHz, $CDCl_3$): δ 65.77.

HRMS (EI, m/z): calc'd for $C_9H_9FO_4S$ $[M]^+$ 232.0200; found 232.0201.

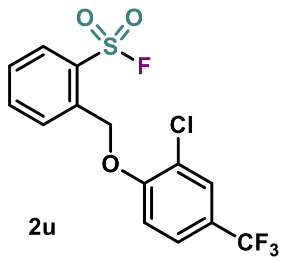

2u

2-((2-Chloro-4-(trifluoromethyl)phenoxy)methyl)benzenesulfonyl fluoride (2u): Following the general procedure A, **2u** was obtained in 66% yield as a white crystalline solid after a flash column chromatography (Eluent: pentane:CH_2Cl_2 8:2).

^{1}H NMR (600 MHz, $CDCl_3$): δ 8.15 (dd, *J* = 8.0, 1.3 Hz, 1H), 8.08 (dd, *J* = 7.6, 1.0 Hz, 1H), 7.85 (td, *J* = 7.7, 1.3 Hz, 1H), 7.63 – 7.61 (m, 1H), 7.54 (dd, *J* = 8.2, 0.8 Hz, 1H), 7.26 – 7.24 (m, 1H), 7.22 (d, *J* = 1.9 Hz, 1H), 5.56 (s, 2H).

^{13}C NMR (151 MHz, $CDCl_3$): δ 153.8, 136.7, 136.2, 131.1, 130.6 (d, *J* = 1.3 Hz), 130.4 (q, *J* = 24.1 Hz), 129.2, 128.9, 127.4 (q, *J* = 1.7 Hz), 123.5 (q, *J* = 272.5 Hz), 119.3 (q, *J* = 3.9 Hz), 110.6 (q, *J* = 3.7 Hz), 67.1.

^{19}F NMR (565 MHz, $CDCl_3$): δ 64.36 (1F), –62.59 (3F).

HRMS (EI, m/z): calc'd for $C_{14}H_9F_4O_2SCl$ $[M]^+$ 367.9891; found 367.9892.

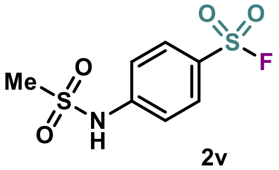

2v

4-(Methylsulfonamido)benzenesulfonyl fluoride (2v): Following the general procedure A, **2v** was obtained in 55% yield as a white solid after a flash column chromatography (Eluent: pentane:CH_2Cl_2 1:1).

^{1}H NMR (600 MHz, $CDCl_3$): δ 8.00 (d, *J* = 8.8 Hz, 2H), 7.39 (d, *J* = 8.6 Hz, 2H), 7.03 (bs, 1H), 3.17 (s, 3H).

^{13}C NMR (151 MHz, $CDCl_3$): δ 143.8, 130.9, 128.1 (d, *J* = 25.6 Hz), 118.2, 40.8.

^{19}F NMR (565 MHz, $CDCl_3$): δ 66.81

HRMS (EI, m/z): calc'd for $C_7H_8FNO_4S$ $[M]^+$ 252.9873; found 252.9878.

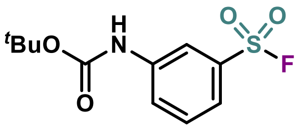

2w

***tert*-Butyl (3-(fluorosulfonyl)phenyl)carbamate (2w)**: Following the general procedure A, **2w** was obtained in 85% yield as a white solid after a flash column chromatography (Eluent: pentane:CH_2Cl_2 6:4).

^{1}H NMR (600 MHz, $CDCl_3$): δ 8.11 (t, *J* = 2.0 Hz, 1H), 7.72 (d, *J* = 8.3 Hz, 1H), 7.65 (ddd, *J* = 7.8, 1.5, 0.7 Hz, 1H), 7.52 (td, *J* = 8.0, 1.0 Hz, 1H), 6.79 (bs, 1H), 1.53 (s, 9H).

^{13}C NMR (151 MHz, $CDCl_3$): δ 152.3, 140.1, 133.9 (d, *J* = 24.5 Hz), 130.4, 124.9, 122.4, 117.7, 82.0, 28.3.

^{19}F NMR (565 MHz, $CDCl_3$): δ 65.43.

HRMS (EI, m/z): calc'd for $C_{11}H_{14}FNO_4S$ $[M]^+$ 275.0622; found 275.0625.

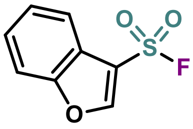

2x

Benzofuran-3-sulfonyl fluoride (2x): Following the general procedure B, **2x** was obtained in 86% yield as a white solid after a flash column chromatography (Eluent: pentane:CH_2Cl_2 8:2).

^{1}H NMR (600 MHz, $CDCl_3$): δ 8.39 (d, *J* = 2.2 Hz, 1H), 7.90 – 7.88 (m, 1H), 7.65 (ddd, *J* = 8.3, 1.2, 0.7 Hz, 1H), 7.54 – 7.48 (m, 2H).

^{13}C NMR (151 MHz, $CDCl_3$): δ 155.5 (d, *J* = 1.2 Hz), 151.5 (d, *J* = 3.7 Hz), 127.4, 125.8, 121.6, 120.5, 116.6 (d, *J* = 33.2 Hz), 112.7.

^{19}F NMR (565 MHz, $CDCl_3$): δ 67.58.

HRMS (ESI, m/z): calc'd for $C_8H_5FO_3SNa$ $[M+Na]^+$ 222.9835; found 229.9835.

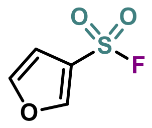

2y

Furan-3-sulfonyl fluoride (2y): Following the general procedure B, **2y** was obtained in 90% yield as yellowish oil after a flash column chromatography (Eluent: pentane:CH_2Cl_2 8:2).

^{1}H NMR (600 MHz, CDCl$_3$): δ 8.19 (ddd, *J* = 2.3, 1.6, 0.8 Hz, 1H), 7.61 (dd, *J* = 1.6 Hz, 1H), 6.82 (dd, *J* = 2.0, 0.9 Hz, 1H).

^{13}C NMR (151 MHz, CDCl$_3$): δ 148.7 (d, *J* = 3.4 Hz), 145.7, 120.9 (d, *J* = 33.0 Hz), 108.7.

^{19}F NMR (565 MHz, CDCl$_3$): δ 67.98.

HRMS (GC-EI, m/z): calc'd for $C_4H_3FO_3S$ $[M]^+$ 149.9781; found 149.9782.

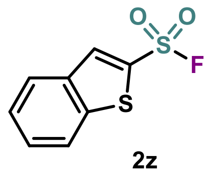

2z

Benzo[*b*]thiophene-2-sulfonyl fluoride (2z): Following the general procedure B, **2z** was obtained in 63% yield as a white solid after a flash column chromatography (Eluent: pentane:CH_2Cl_2 8:2).

^{1}H NMR (600 MHz, CDCl$_3$): δ 8.21 – 8.20 (m, 1H), 8.00 – 7.98 (m, 1H), 7.94 (dq, *J* = 8.3, 0.9 Hz, 1H), 7.61 (ddd, *J* = 8.4, 7.1, 1.2 Hz, 1H), 7.54 (ddd, *J* = 8.1, 7.1, 1.0 Hz, 1H).

^{13}C NMR (151 MHz, CDCl$_3$): δ 143.2 (d, *J* = 1.4 Hz), 136.9, 134.5 (d, *J* = 1.7 Hz), 131.6 (d, *J* = 31.0 Hz), 129.1, 126.7, 126.3, 123.0.

^{19}F NMR (565 MHz, CDCl$_3$): δ 70.95.

HRMS (ESI, m/z): calc'd for $C_8H_5FO_2S_2Na$ $[M+Na]^+$ 238.9607; found 238.9609.

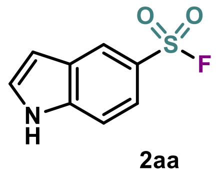

2aa

1*H*-Indole-5-sulfonyl fluoride (2aa):[10] Following the general procedure B, **2aa** was obtained in 45% yield as a yellowish solid after a flash column chromatography (Eluent: pentane:CH_2Cl_2 3:7).

^{1}H NMR (600 MHz, CDCl$_3$): δ 8.65 (bs, 1H), 8.38 (dd, *J* = 1.8, 0.7 Hz, 1H), 7.80 (dd, *J* = 8.7, 1.9 Hz, 1H), 7.57 (dd, *J* = 8.7, 0.8 Hz, 1H), 7.43 (dd, *J* = 3.3, 2.3 Hz, 1H), 6.76 (ddd, *J* = 3.1, 2.0, 1.0 Hz, 1H).

^{13}C NMR (151 MHz, CDCl$_3$): δ 139.1, 127.6, 127.5, 123.9 (d, *J* = 23.2 Hz), 123.5, 121.4, 112.2, 104.8.

^{19}F NMR (565 MHz, CDCl$_3$): δ 68.32.

HRMS (ESI, m/z): calc'd for $C_8H_5FO_2S$ $[M-H]^-$ 198.0030; found 198.0031.

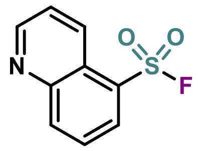

2ab

Quinoline-5-sulfonyl fluoride (2ab): Following the general procedure B, **2ab** was obtained in 62% yield as a white solid after a flash column chromatography (Eluent: pentane:CH_2Cl_2 6:4).

^{1}H NMR (600 MHz, $CDCl_3$): δ 9.19 (dd, *J* = 4.2, 1.8 Hz, 1H), 8.52 (dd, *J* = 7.4, 1.4 Hz, 1H), 8.32 (dd, *J* = 8.4, 1.7 Hz, 1H), 8.23 (dd, *J* = 8.2, 1.4 Hz, 1H), 7.73 – 7.70 (m, 1H), 7.63 (dd, *J* = 8.3, 4.2 Hz, 1H).

^{13}C NMR (151 MHz, $CDCl_3$): δ 152.8, 143.9, 136.7, 136.2, 133.2, 131.6 (d, *J* = 21.2 Hz), 129.2, 125.4, 123.1.

^{19}F NMR (565 MHz, $CDCl_3$): δ 60.19.

HRMS (ESI, m/z): calc'd for $C_9H_6FNO_2SNa$ $[M+Na]^+$ 233.9995; found 233.9994.

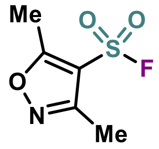

2ac

3,5-Dimethylisoxazole-4-sulfonyl fluoride (2ac): Following the general procedure B, **2ac** was obtained in 68% yield as thick colorless oil after a flash column chromatography (Eluent: pentane:CH_2Cl_2 6:4).

^{1}H NMR (600 MHz, $CDCl_3$): δ 2.72 (s, 3H), 2.47 (s, 3H).

^{13}C NMR (151 MHz, $CDCl_3$): δ 157.7, 135.8, 129.6 (d, *J* = 48.5 Hz), 12.9, 10.6.

^{19}F NMR (565 MHz, $CDCl_3$): δ 71.02.

HRMS (GC-EI, m/z): calc'd for $C_5H_6FNO_2S$ $[M]^+$ 179.0047; found 179.0050.

6. Mechanistic studies

6.1. Validation of the organometallic steps: Transmetallation

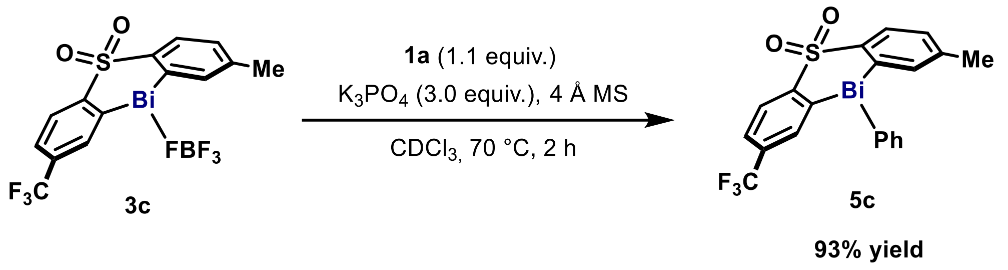

Procedure: In a culture tube, diarylbismuth tetrafluoroborate **3c** (0.02 mmol), phenyl boronic acid **1a** (0.022 mmol, 1.1 equiv.), K_3PO_4 (0.06 mmol, 3.0 equiv.) and 10 mg of 4 Å MS were mixed with anhydrous $CDCl_3$ (0.7 mL) under an Ar atmosphere and the reaction was stirred for 2 h at 70 °C. Then, 1,3,5-trimethoxybenzene was added as internal standard and the crude reactions were analyzed by ^1H NMR to determine the yield of **5c** (Figure S3).

Figure S3: Crude ^1H NMR of transmetalation of **1a** and **3c.** (Peak at 6.09 ppm: 1.28 equiv. of 1,3,5-trimethoxybenzene as internal standard).

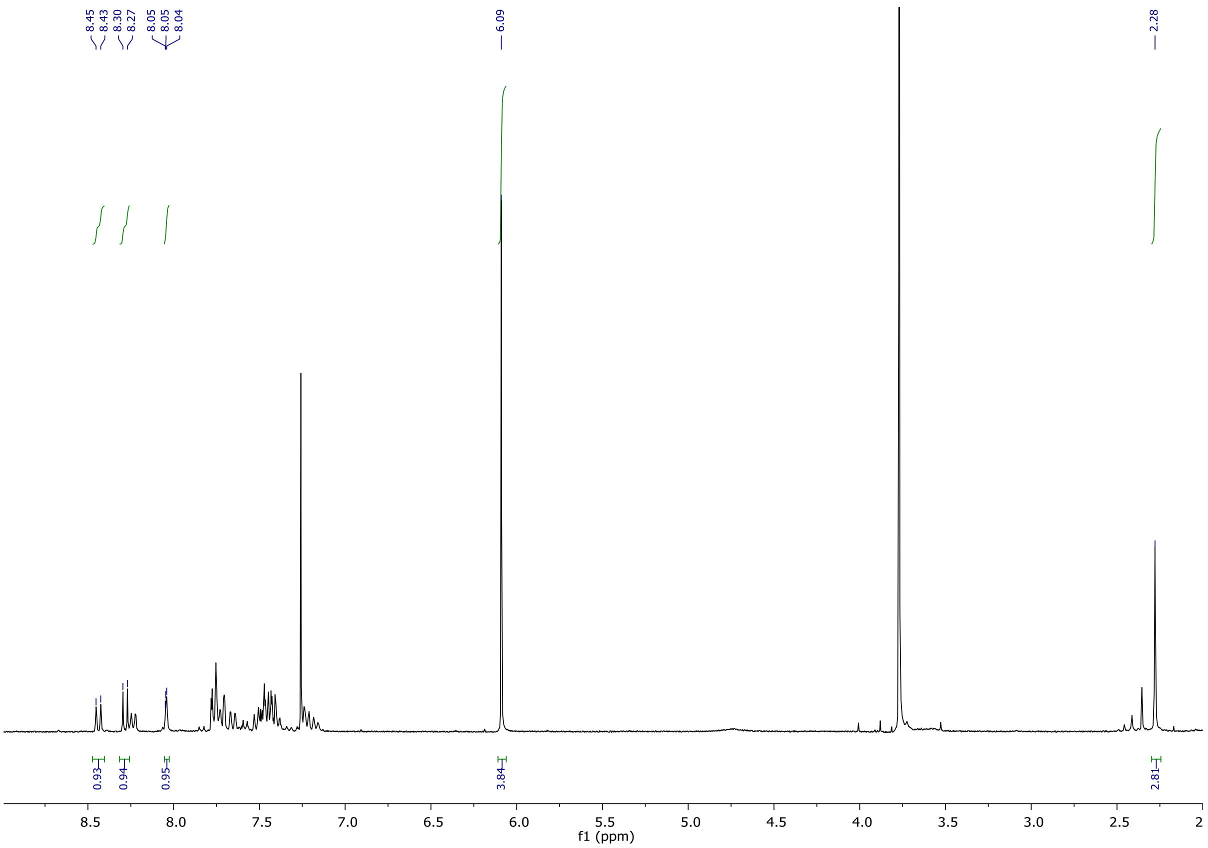

6.2. Bi(V) species as active intermediates

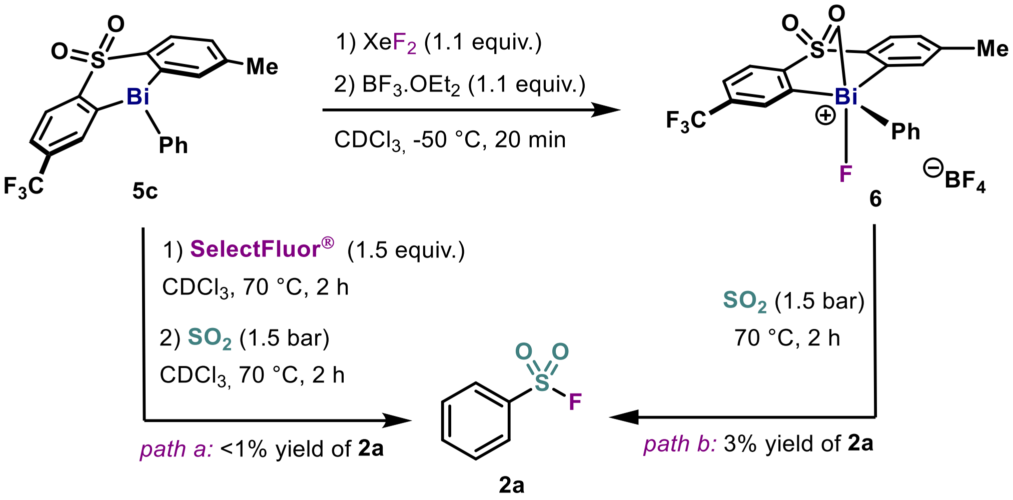

6.2.1. Path a

Procedure: In a pressure Schlenk under argon, triarylbismuth compound **5c** (0.024 mmol) and Selectfluor® (1.5 equiv.) were dissolved in 0.7 mL of $CDCl_3$. The reaction was stirred at 70 °C for 2 h. Then, the reaction crude was cooled to 23 °C and it was pressurized with SO_2 (1.5 bar) and the reaction was left stirring at 70 °C for an additional 2 h. Then, internal standard (1,4-difluorobenzene) was added by weight and the crude was analyzed by ^{19}F NMR (Figure S4).

In ^{19}F NMR we can observe only trace amounts (<1%) of our desired product **2a**. In ^{1}H NMR we can observe that new Bi-species are formed together with the formation of benzene (25%). This finding suggests that the oxidation of **5c** with Selectfluor® furnishes bismuth intermediates that, in the presence of SO_2, provide not the desired product **2a**, but benzene in 25% yield.

Figure S4. ^{19}F NMR of crude mixture after following **path a** (1.22 equiv. of 1,4-difluorobenzene as internal standard).

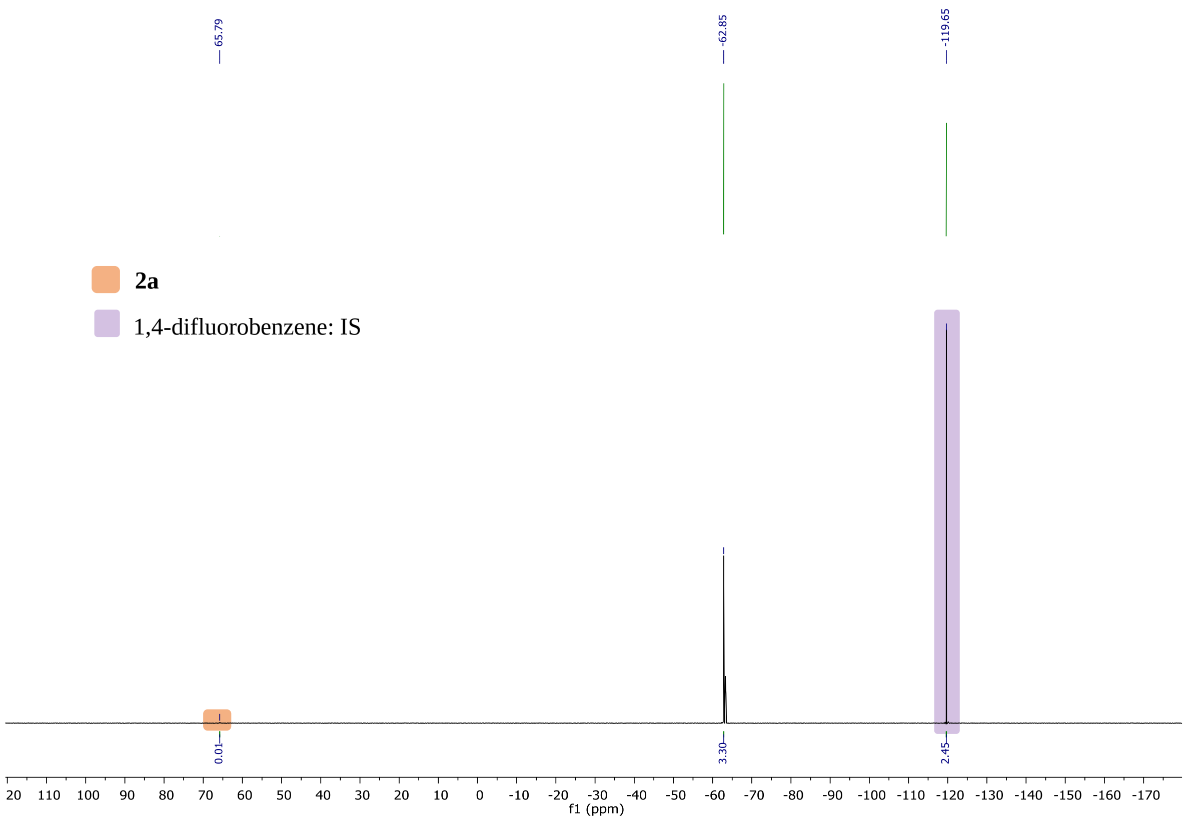

6.2.2. Path b

Procedure step 1: In a culture tube under argon, triarylbismuth compound **5c** (0.07 mmol) was dissolved in dry $CHCl_3$ (2 mL) and at 0 °C, XeF_2 (1.1 equiv.) was added in one portion. The reaction was stirred at this temperature for 1 h. Then, the volatiles were evaporated under vacuum and the desired pentavalent bismuth difluoride **11** was obtained as a beige solid in quantitative yield (42 mg).

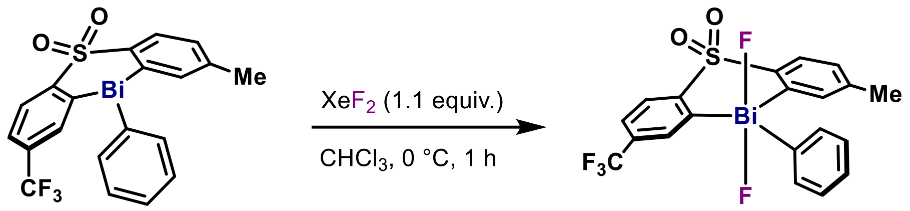

5c **11**

^{1}H NMR (300 MHz, CDCl$_3$): δ 8.59 (bs, 1H), 8.40 – 8.38 (m, 2H), 8.22 – 8.14 (m, 3H), 7.82 – 7.79 (m, 1H), 7.70 (t, *J* = 7.6 Hz, 2H), 7.60 (d, *J* = 7.1 Hz, 1H), 7.39 (d, *J* = 7.9 Hz, 1H), 2.48 (s, 3H).

^{13}C NMR - The high insolubility of **11** prevented its characterization by ^{13}C NMR due to the appearance of broad signals.

^{19}F NMR (282 MHz, CDCl$_3$): δ –62.90. Fluorine atoms attached to Bi atom are not visible.

HRMS (ESI, m/z): calc'd for $C_{20}H_{14}BiF_4SO_2$ $[M-F]^+$ 603.0449; found 603.0450.

Procedure step 2: In a NMR tube under argon, Bi(V) difluoride **11** (0.024 mmol) was dissolved in 0.6 mL of $CDCl_3$. The reaction was cooled to –50 °C and then $BF_3{\cdot}OEt_2$ (1.1 equiv.) was added. After 20 min, bismuth complex **6** was characterized by NMR at –50 °C.

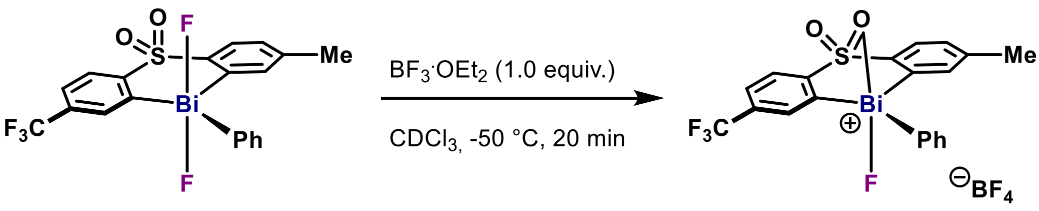

11 **6**

^{1}H NMR (500 MHz, CDCl$_3$): δ 8.60 (d, *J* = 9.1 Hz, 2H), 8.46 (d, *J* = 8.0 Hz, 1H), 8.27 (d, *J* = 7.8 Hz, 1H), 8.11 (d, *J* = 8.3 Hz, 2H), 7.96 (d, *J* = 8.2 Hz, 1H), 7.88 (t, *J* = 7.9 Hz, 2H), 7.72 (t, *J* = 7.3 Hz, 1H), 7.56 (d, *J* = 7.9 Hz, 1H), 2.57 (s, 3H).

^{13}C NMR (126 MHz, $CDCl_3$): δ 168.1, 167.3, 158.4, 151.6, 147.8, 138.4, 137.8 (q, *J* = 33.9 Hz), 134.9, 134.4, 133.4, 132.6, 132.0 (d, *J* = 4.0 Hz), 129.8, 129.4 (d, *J* = 3.7 Hz), 128.7, 122.4 (q, *J* = 274.6 Hz), 22.7.

^{19}F NMR (470 MHz, $CDCl_3$): δ –62.61 (3F), –153.24 (4F). Fluorine atom attached to bismuth is not visible.

Procedure path b: The reaction was carried out in a pressure Schlenk, where Bi(V) difluoride **11** (0.024 mmol) was dissolved under argon in 0.6 mL of $CDCl_3$. The reaction was cooled to –50 °C and then $BF_3.OEt_2$ (1.1 equiv.) was added. After 10 min at –50 °C, the Schlenk was pressurized with SO_2 (1.5 bar) and the reaction was heated at 70 °C for 2 h. Then, internal standard (1,4-difluorobenzene) was added by weight and the crude was analyzed by ^{19}F NMR (Figure S5).

We can observe that only trace amounts (<5%) of our desired product **2a** are obtained. On the other hand, fluorobenzene (49%) is the major product in the crude mixture. This finding is in agreement with previously published literature it is shown that cationic bismuth(V) **6**-type compounds are active towards the synthesis of fluoroarenes via a reductive elimination in **6**.[14]

Figure S5. ^{19}F Crude NMR of **path b** (0.73 equiv. of 1,4-difluorobenzene as internal standard).

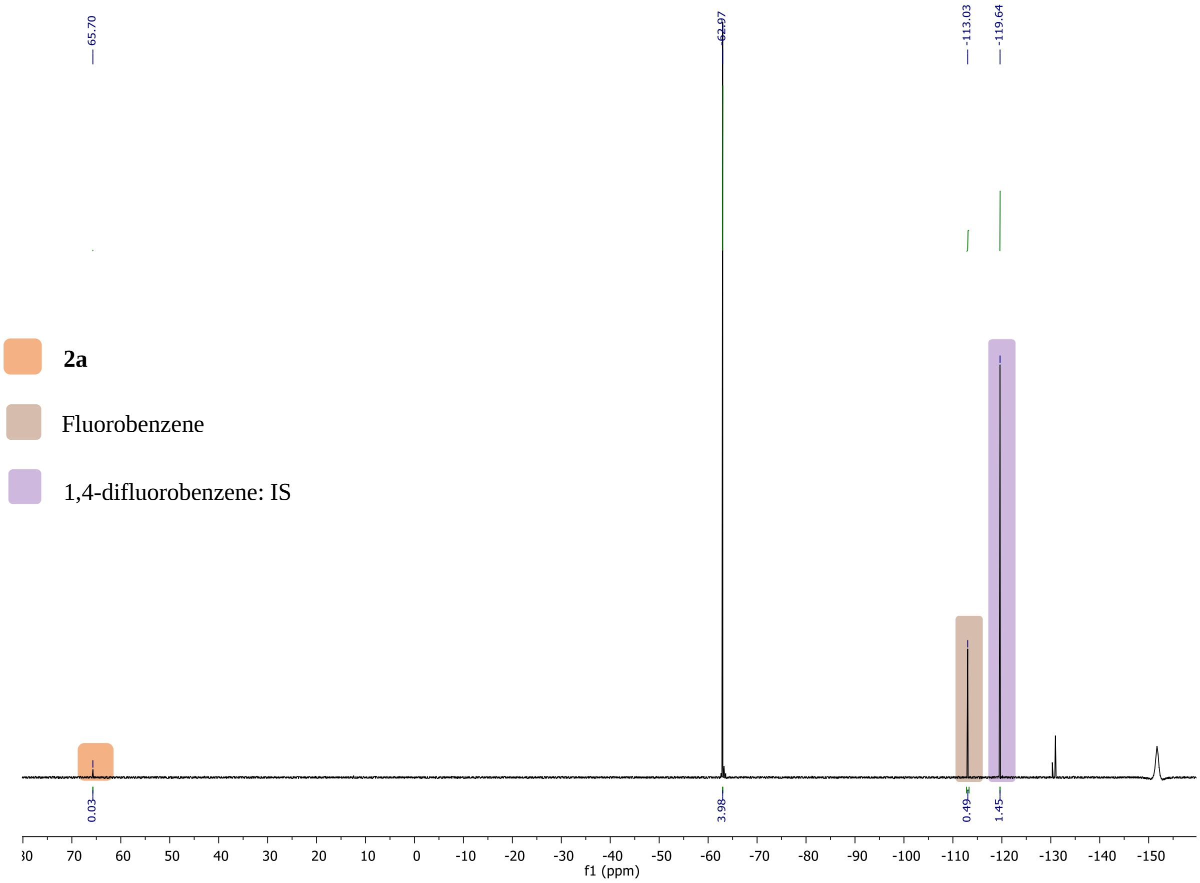

6.3. Validation of the organometallic steps: SO_2 insertion into Bi(III)–C bond

6.3.1. SO_2 insertion into Bi–C bond in **5c** with SO_2 gas:

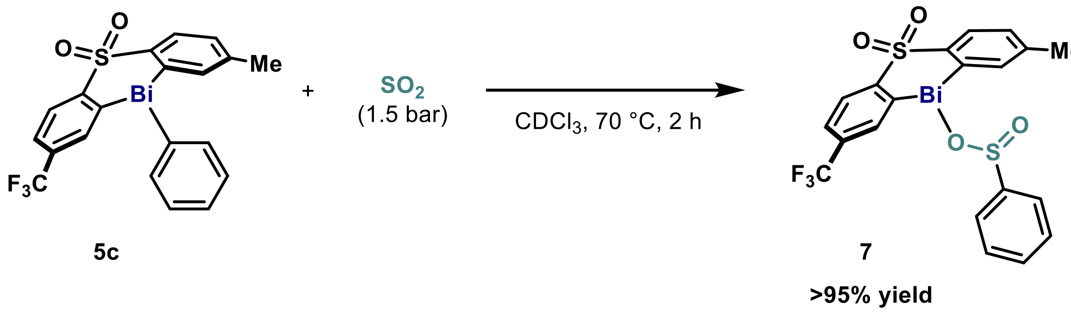

Procedure: In a pressure Schlenk under argon, triarylbismuth **5c** (0.025 mmol) was dissolved in anhydrous $CDCl_3$ (0.6 mL). Then, the Schlenk was pressurized with SO_2 (1.5 bar) and the reaction was stirred for 2 h at 70 °C. Then, internal standard (1,3,5-trimethoxybenzene) was added and the crude was analyzed by ^1H NMR (Figure S6).

Figure S6. ^1H NMR of the bismuth sulfinate **7**. (Peak at 6.09 ppm: 0.95 equiv. of 1,3,5-trimethoxybenzene as internal standard).

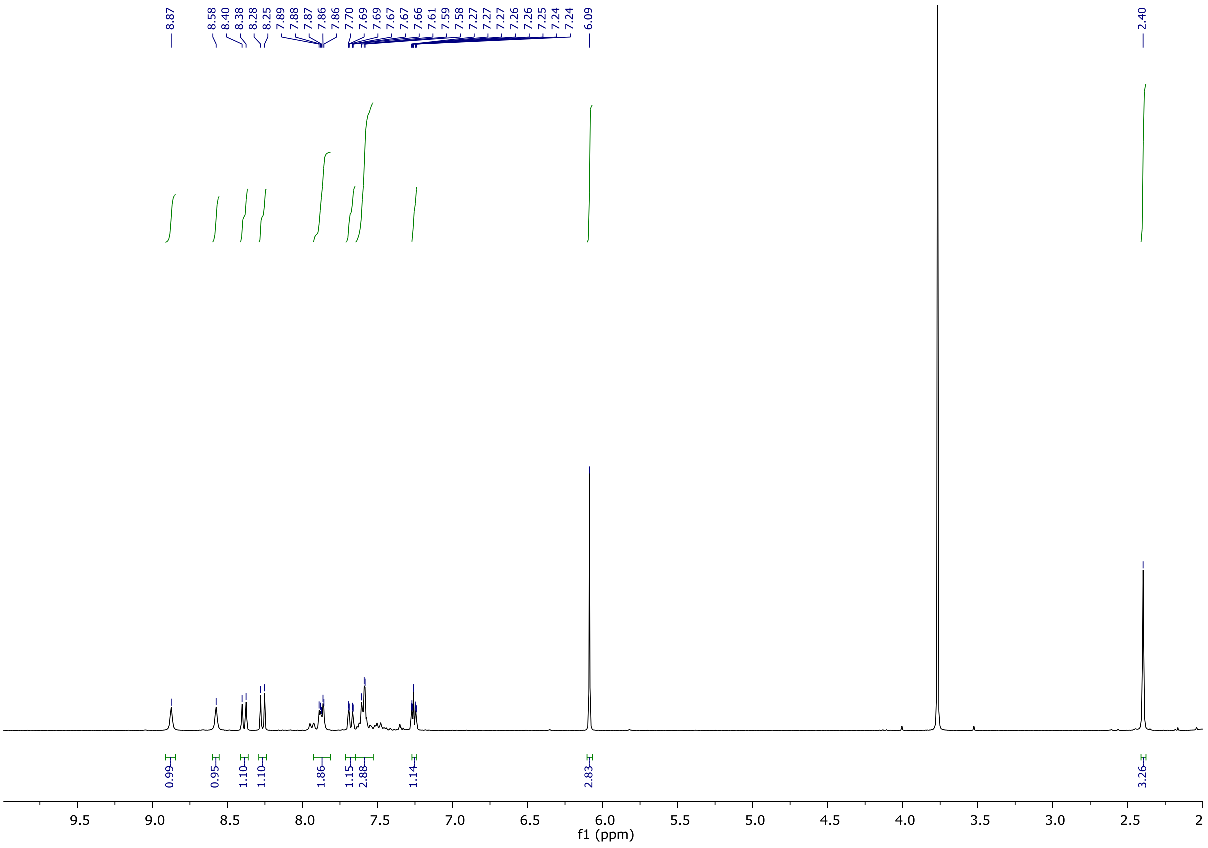

6.3.2. SO_2 insertion into Bi–C bond in **5c** using DABSO:

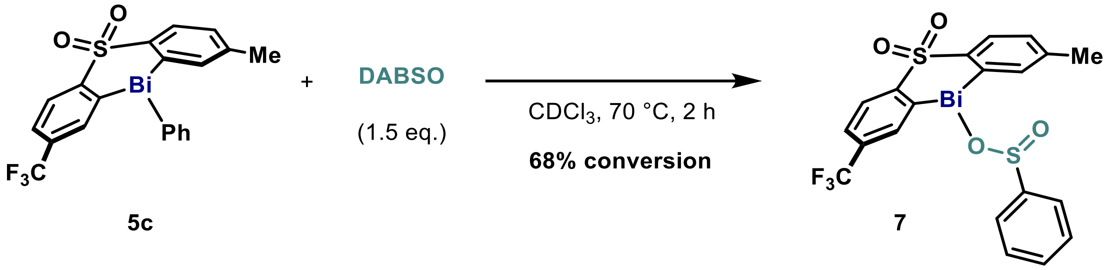

Procedure: In a culture tube under argon, triarylbismuth **5c** (0.025 mmol) and DABSO (1.5 equiv.) were dissolved in anhydrous $CDCl_3$ (0.6 mL) and the reaction was stirred for 2 h at 70 °C and the crude mixture was analyzed by ^{19}F NMR to check the conversion (Figure S7).

Figure S7. ^{19}F NMR of the crude reaction of the triarylbismuth **5c** and DABSO.

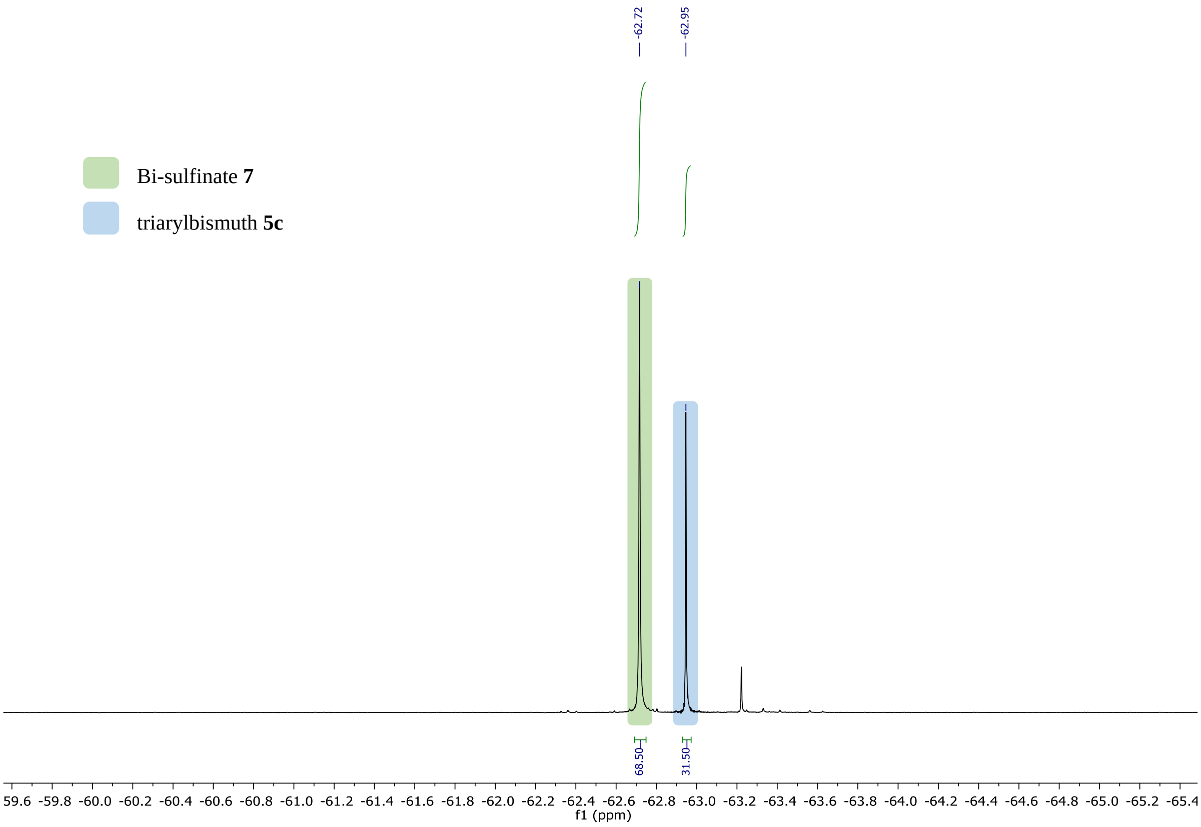

*6.4. Validation of the organometallic steps: Bi-sulfinate **7** oxidation*

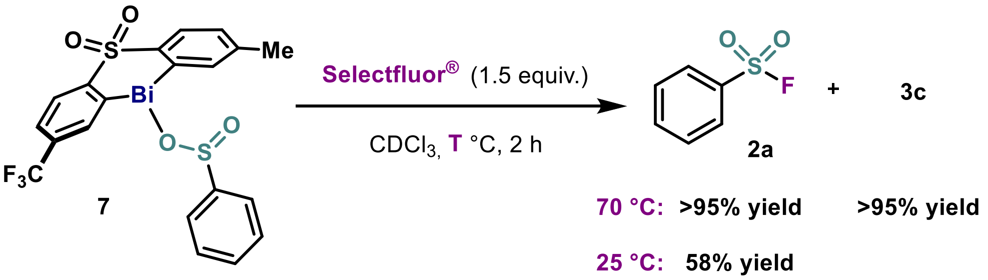

Procedure: In a culture tube under argon, bismuth sulfinate **7** (0.025 mmol) and Selectfluor® (1.5 equiv.) were dissolved in anhydrous $CDCl_3$ (0.7 mL) and the reaction was stirred for 2 h at 70 and the crude mixture was analyzed by ^1H NMR (Figure S8). In order to dissolve all the solids in the crude mixture, $CDCl_3$ was evaporated under vacuum and the crude was dissolved in CD_3CN. Moreover, in order to evaluate the reactivity of the bismuth sulfinate **7**, the same reaction was carried out at 25 °C and the crude was analyzed by ^{19}F NMR, indicating a 58% yield of **2a** at 25 °C (Figure S9).

Figure S8. ^1H NMR of crude mixture of oxidation of bismuth sulfinate **7** at 70 °C.

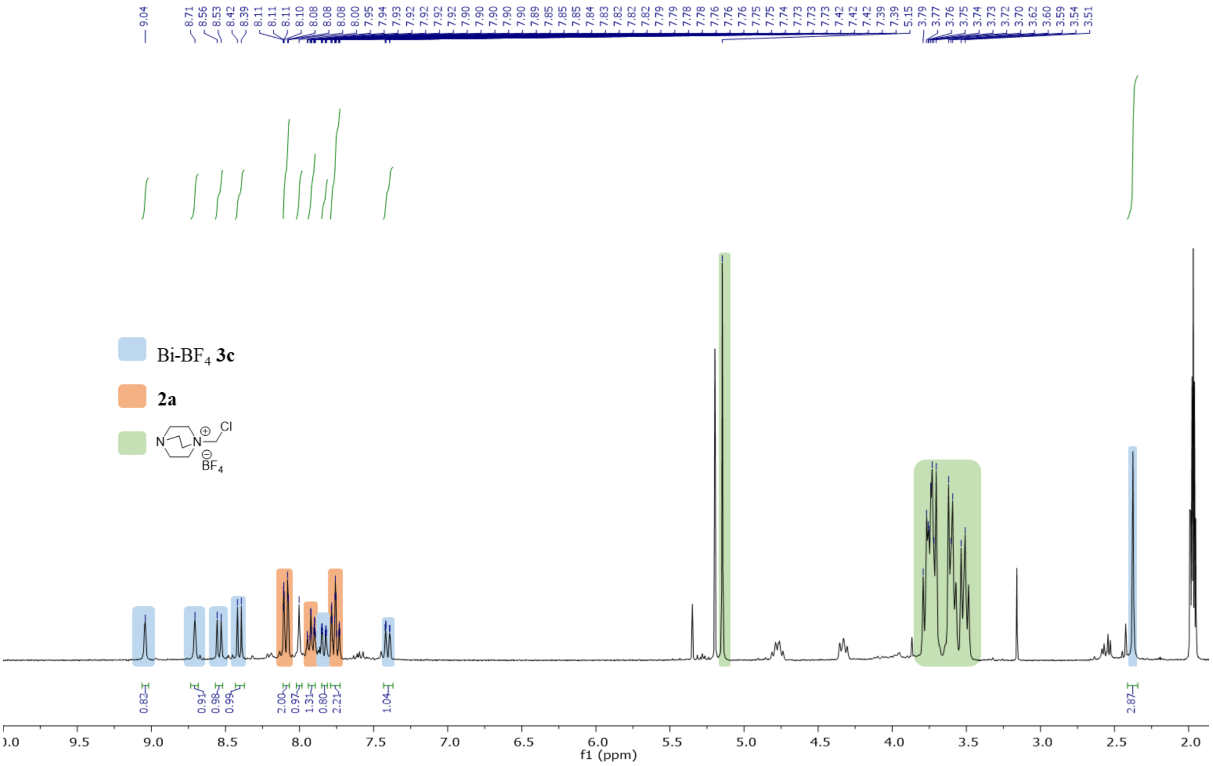

Figure S9. ^{1}H NMR of crude mixture of oxidation of bismuth sulfinate **7** at 25 °C in CDCl3. (1.5 equiv. of 1,4-difluorobenzene as internal standard).

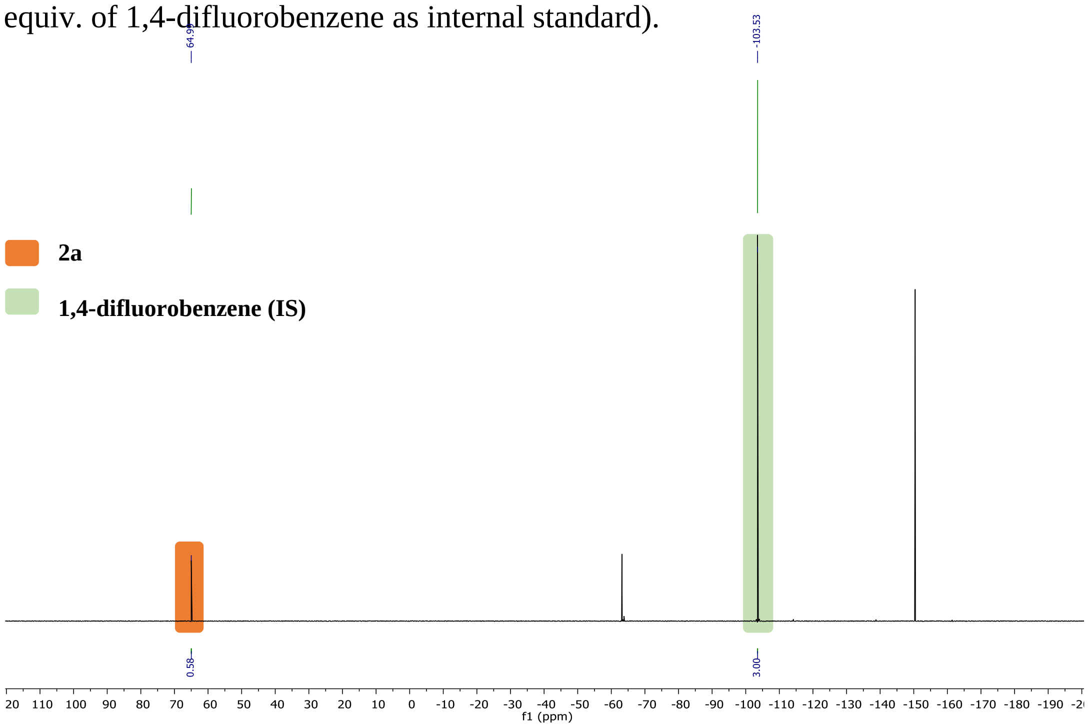

6.5. Oxidation of Bi(III)-S(VI) species

Synthesis of diarylbismuth tosylate (8): In a round bottom flask, triarylbismuth **5c** (0.54 mmol) and TsOH·H_2O (0.06 mmol, 1.1 equiv.) were dissolved in Et_2O (10 mL). The reaction was stirred for 16 h at 25 °C and then, the white precipitate was filtered, washed with Et_2O (2 × 10 mL) and dried under vacuum, obtaining **8** (340 mg, 93% yield) as a white solid.

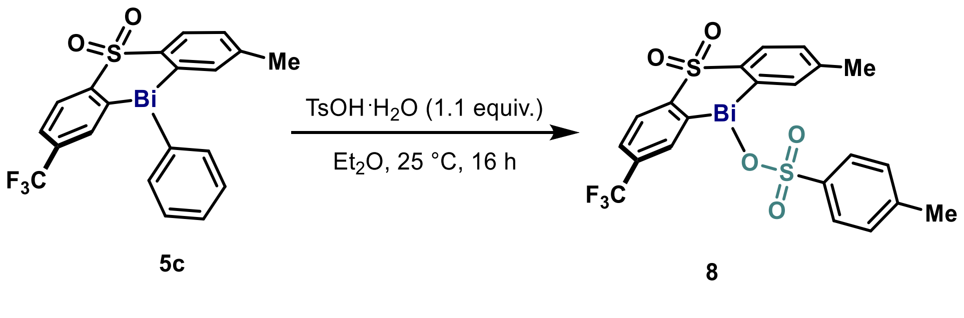

2-Methyl-5,5-dioxido-8-(trifluoromethyl)-10H-dibenzo[*b*,*e*]-[1,4]thiabismin-10-yl 4-methylbenzenesulfonate (8):

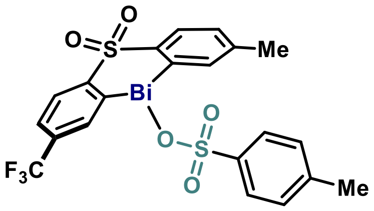

8

^{1}H NMR (400 MHz, CD$_3$CN): δ 9.15 (bs, 1H), 8.68 (bs, 1H), 8.51 (d, *J* = 8.0 Hz, 1H), 8.36 (d, *J* = 7.9 Hz, 1H), 7.84 (dd, *J* = 8.1, 1.0 Hz, 1H), 7.78 (d, *J* = 8.0 Hz, 2H), 7.39 (dd, *J* = 7.9, 1.6 Hz, 1H), 7.35 (d, *J* = 8.0 Hz, 2H), 2.42 (s, 3H), 2.40 (s, 3H).

^{13}C NMR (101 MHz, CD$_3$CN): δ 148.6, 145.3, 143.0, 139.9, 136.8, 136.6, 132.8 (q, *J* = 3.7 Hz), 130.3, 130.2, 129.9, 129.7, 126.8, 126.7, 21.6, 21.0.

^{19}F NMR (282 MHz, CD$_3$CN): δ –63.39.

HRMS (ESI, m/z): calc'd for $C_{21}H_{17}BiF_2S_2O_5$ $[M+H]^+$ 679.0268; found 679.0272.

Procedure for oxidation of 8: In a culture tube under argon, diarylbismuth tosylate **8** (0.02 mmol) and Selectfluor® (2.5 equiv.) were dissolved in 0.7 mL of $CDCl_3$ and heated at 70 °C for 2 h. The solvent was then evaporated under vacuum and crude was dissolved in CD_3CN in order to solubilize all the crude material. Then, internal standard (1,4-difluorobenzene) was added by weight and the crude was analyzed by ^{1}H and ^{19}F NMR (Figure S10).

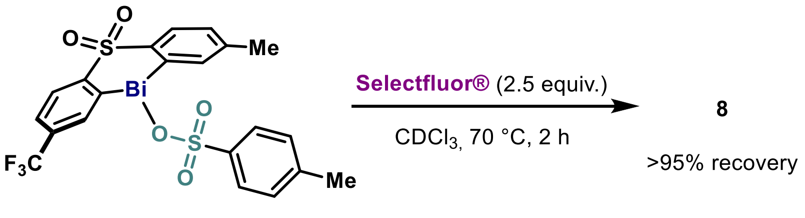

8

Figure S10. ^{1}H and ^{19}F NMR of the reaction crude (1.0 equiv. of 1,4-difluorobenzene as internal standard).

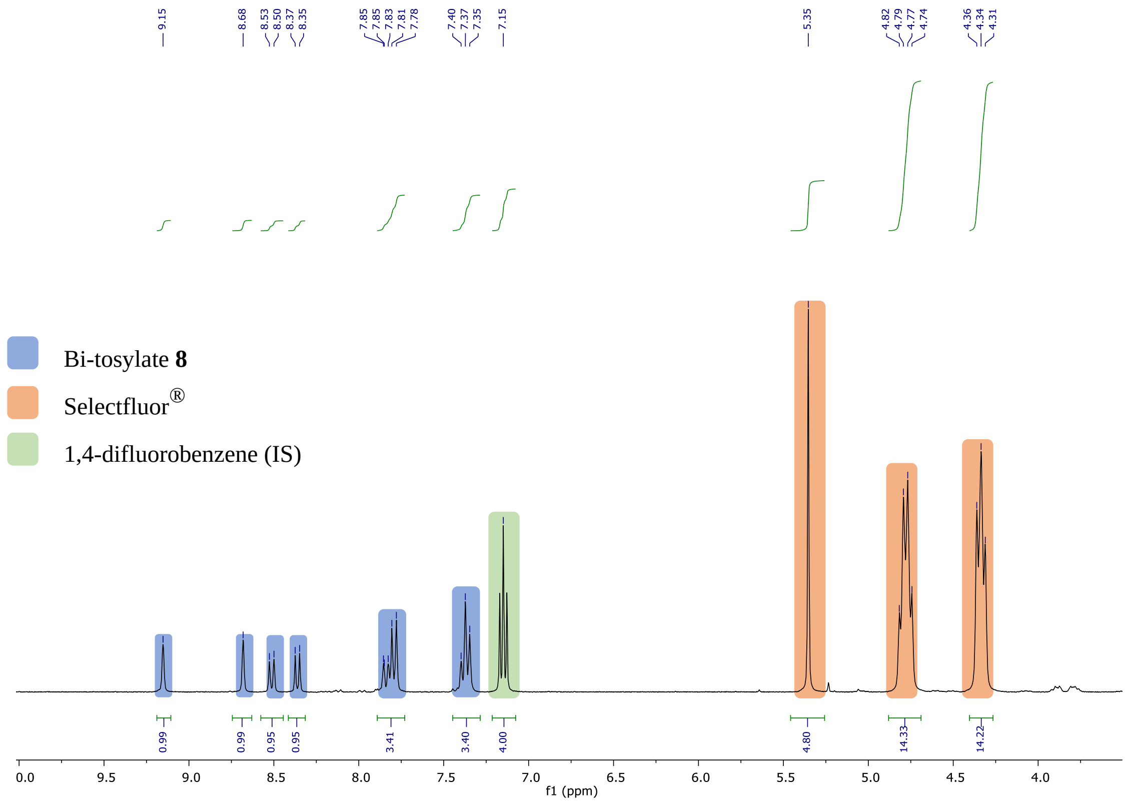

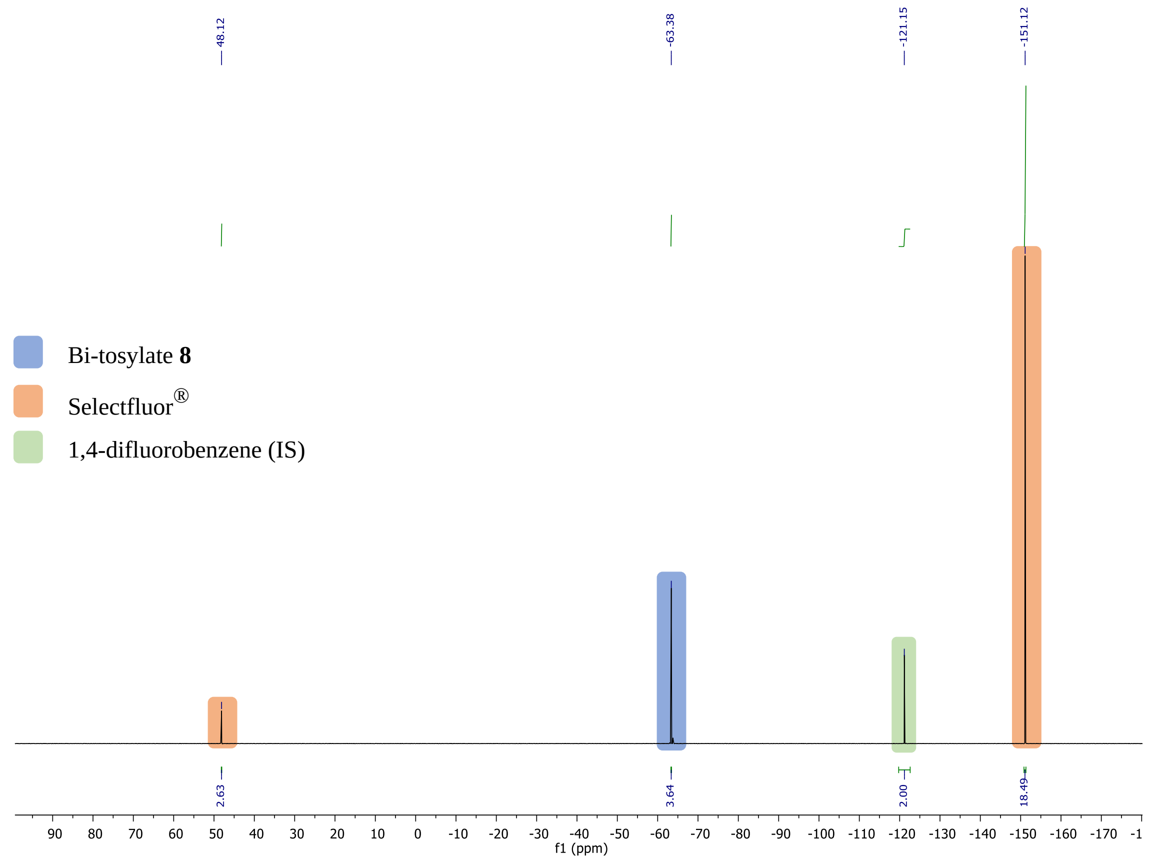

As we observe in the reaction crude (Figure S10), diarylbismuth tosylate **8**, which contains a Bi(III) and a S(VI) atoms does not undergo oxidation, under the described conditions. This result points toward a low-reactivity of a Bi(III)-X species in oxidation, and would suggest that in the catalytic conditions the S(IV) gets oxidized to S(VI) (validating section 6.4).

*6.6. Reactivity of diarylbismuth tetrafluoroborate **3c***

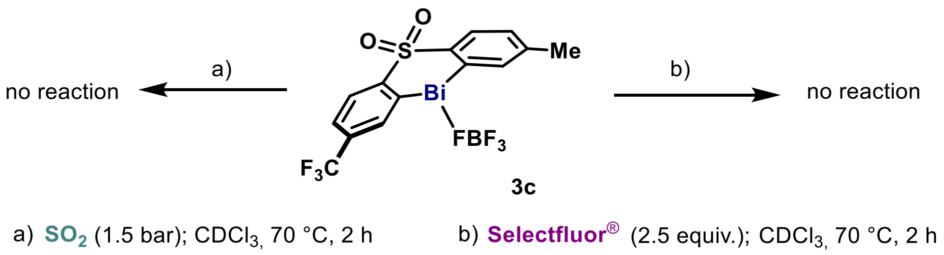

6.6.1. Reactivity with SO_2 (a):

Procedure: In a pressure Schlenk under argon, diarylbismuth tetrafluoroborate **3c** (0.024 mmol) was dissolved in 1 mL of $CDCl_3$ and the Schlenk was pressurized with SO_2 (1.5 bar). After heating at 70 °C for 2 h, the volatiles were evaporated under vacuum and the crude was re-dissolved in CD_3CN for solubility reasons. Then, internal standard (1,4-difluorobenzene) was added by weight and the crude was analyzed by ^{1}H NMR (Figure S11).

Figure S11. ^{1}H NMR of the reaction crude (0.97 equiv. of 1,4-difluorobenzene as internal standard).

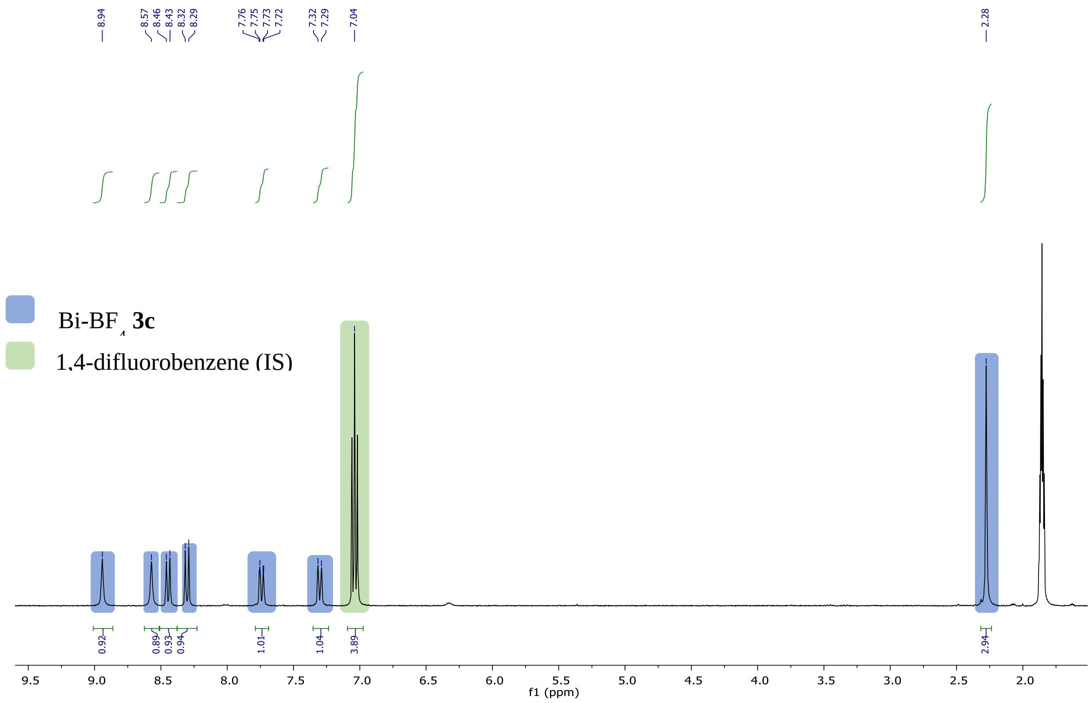

6.6.2. Reactivity with Selectfluor® (b):

Procedure: In a culture tube under argon, diarylbismuth tetrafluoroborate **3c** (0.024 mmol) and Selectfluor® (2.5 equiv.) were dissolved in 1 mL of $CDCl_3$ and heated at 70 °C for 2 h. After the reaction time, the volatiles were evaporated under vacuum and the crude was re-dissolved in CD_3CN for solubility reasons. Then, internal standard (1,4-difluorobenzene) was added by weight and the crude was analyzed by ^1H NMR (Figure S12).

Figure S12. ^1H NMR of the reaction crude (1.39 equiv. of 1,4-difluorobenzene as internal standard).

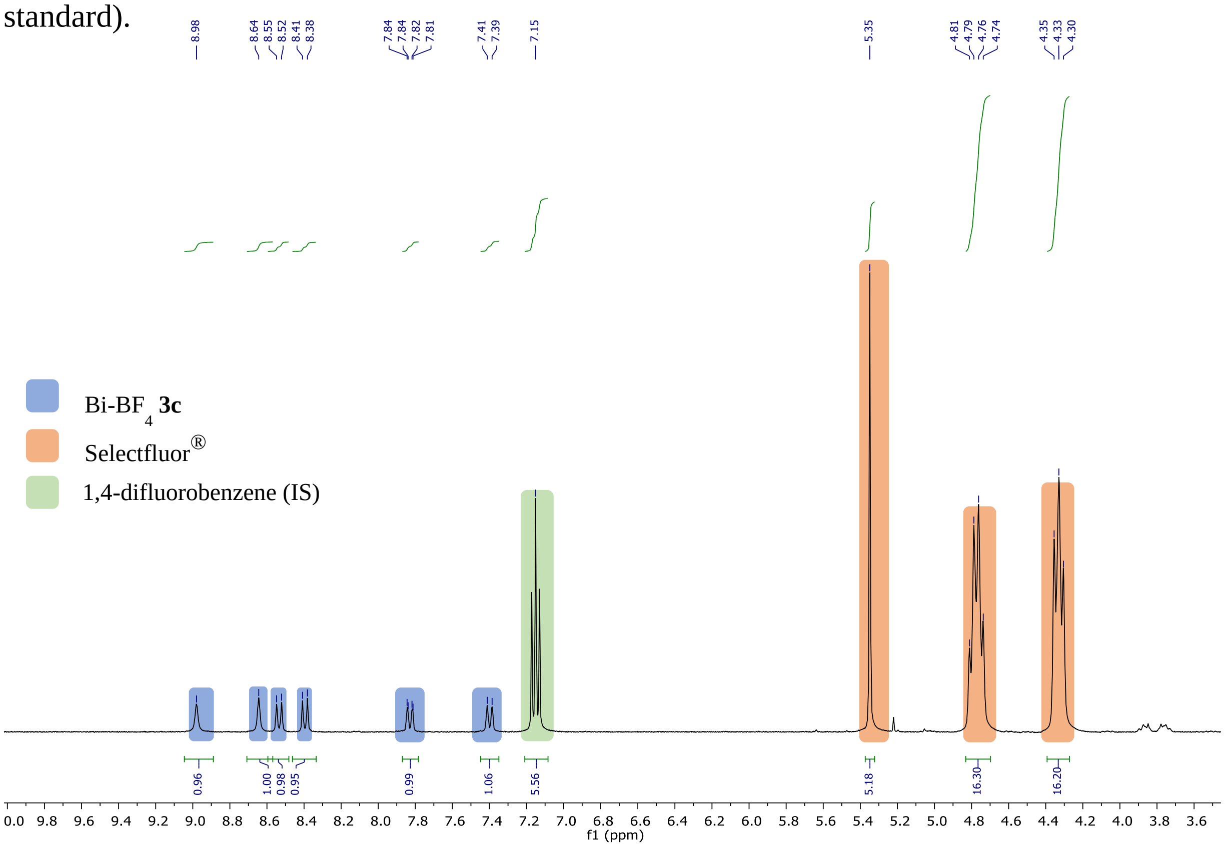

These control experiments in Section 6.6 validate our proposed mechanism, in which catalyst **3c** only undergoes transmetalation, to afford the corresponding triarylbismuth **5c**. In this case, diarylbismuth tetrafluoroborate **3c** does not react neither with SO_2 nor with electrophilic fluorinating agent Selectfluor®.

7. Proposed mechanism

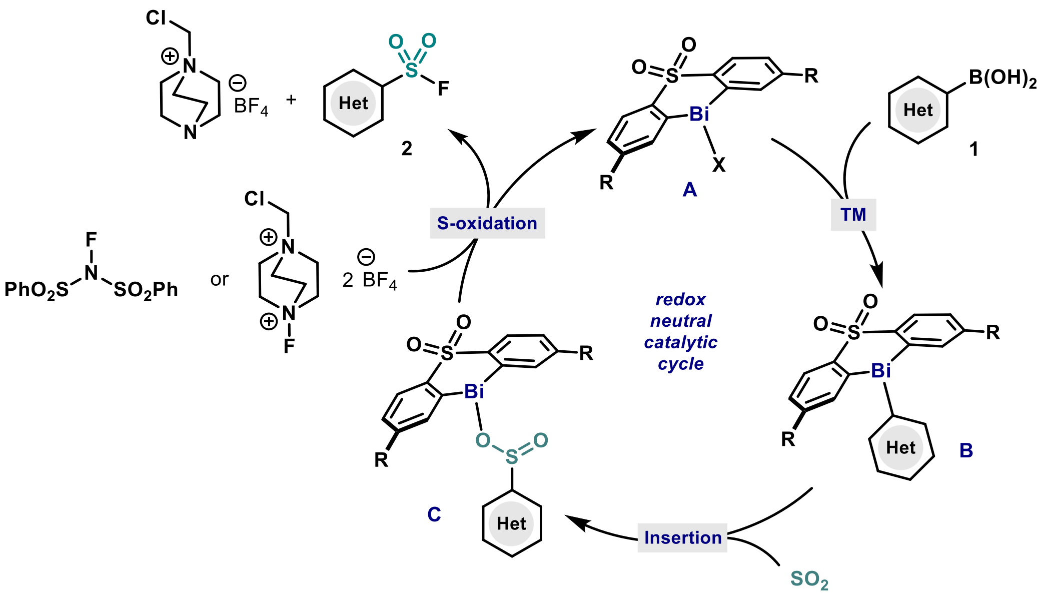

Scheme S2. Proposed mechanism. Redox neutral Bi(III)-catalyzed synthesis of (hetero)aryl sulfonyl fluorides *via* Bi(III)-sulfinate intermediate **C**.

8. Single crystal structure analysis of bismuth sulfinate 7

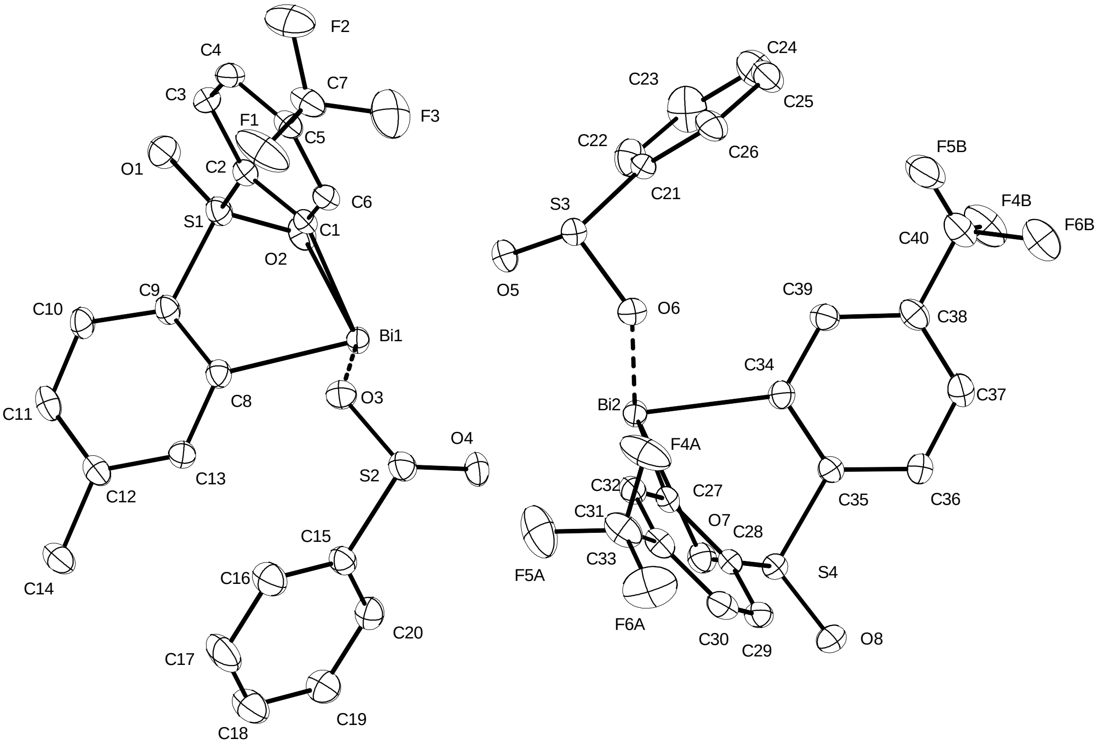

Figure S13. The crystal structure has two molecules of **7** in the asymmetric unit, which are linked asymmetrically by two long additional Bi…O contacts (not shown) of 2.85 and 2.67 Å. One of the two bismuth moieties is disordered in a 4:1 fashion, forcing the trifluoromethylgroup to overlap with the terminal methyl group and vice versa. H atoms have been removed for clarity.

X-ray Crystal Structure Analysis of 7: $C_{40}H_{28}Bi_2F_6O_8S_4$, M = 1296.82 g·mol^{-1}, colourless, crystal size 0.10 x 0.10 x 0.06 mm, monoclinic, *P2₁/n* (no. 14), a = 14.3018(9), b = 18.3091(10), c = 16.0212(11) Å , β = 90.399(6), V = 4195.1(5) Å^3, Z = 4, T = 100(2) K, ρ = 2.053 g·m^{-3}, $\mu(Mo\text{-}K_\alpha)$ = 8.655 mm^{-1}, λ = 0.71073 Å. X-ray diffraction data were collected using a Bruker AXS Enraf-Nonius KappaCCD diffractometer with a FR591 rotating Mo-anode X-ray source, 2.852 < θ < 27.498°, 91024 measured reflections, 15278 independent reflections, 12773 reflections with $I > 2\sigma(I)$, R_{int} = 0.0510. The structure was solved by *SHELXT* and refined by full-matrix least-squares (*SHELXL*) against F^2 to R_1 = 0.0296 [$I > 2\sigma(I)$], wR_2 = 0.0633, 571 parameters.

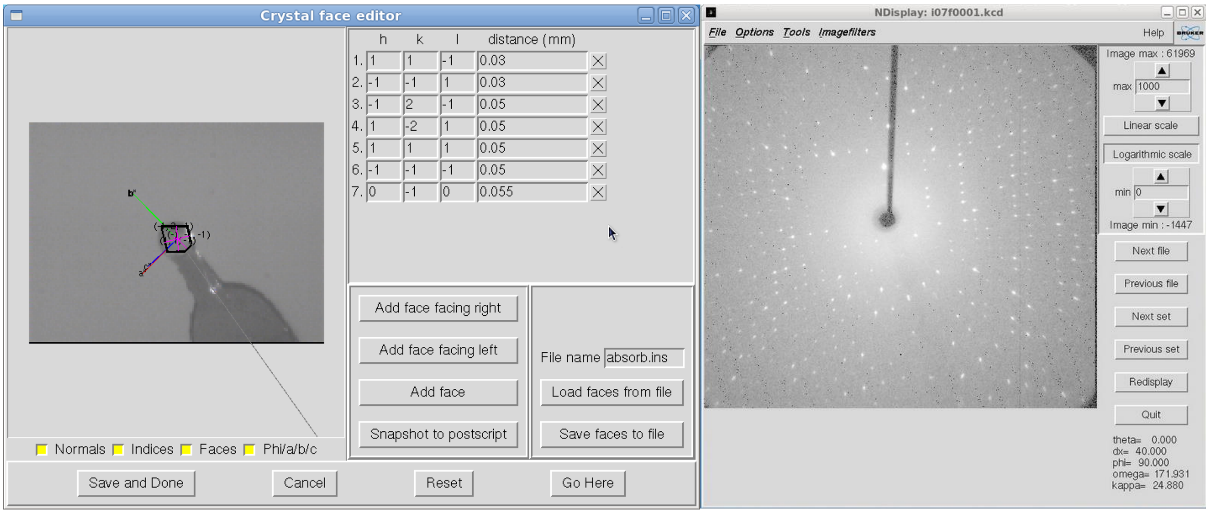

INTENSITY STATISTICS FOR DATASET # 1 14049sad.raw

Resolution	#Data	#Theory	%Complete	Redundancy	Mean I	Mean I/s	Rmerge	Rsigma
Inf - 2.65	252	264	95.5	11.83	73.82	63.91	0.0439	0.0132
2.65 - 1.77	585	585	100.0	8.90	53.39	50.20	0.0375	0.0147
1.77 - 1.40	857	857	100.0	8.10	37.01	42.64	0.0368	0.0164
1.40 - 1.22	833	833	100.0	7.67	28.66	37.74	0.0360	0.0186
1.22 - 1.11	831	831	100.0	7.22	21.47	31.89	0.0368	0.0217
1.11 - 1.03	824	824	100.0	6.98	20.28	29.26	0.0408	0.0234
1.03 - 0.97	804	804	100.0	6.65	16.61	26.02	0.0445	0.0268
0.97 - 0.92	875	875	100.0	6.37	14.55	23.30	0.0489	0.0302
0.92 - 0.88	816	816	100.0	6.07	12.26	20.51	0.0549	0.0349
0.88 - 0.84	987	987	100.0	5.79	10.41	17.99	0.0625	0.0403
0.84 - 0.82	578	578	100.0	5.60	9.79	16.64	0.0669	0.0443
0.82 - 0.79	958	958	100.0	5.31	8.57	14.76	0.0738	0.0507
0.79 - 0.77	732	732	100.0	5.20	7.37	13.09	0.0836	0.0581
0.77 - 0.75	806	806	100.0	4.83	7.50	12.47	0.0888	0.0627
0.75 - 0.73	895	895	100.0	4.76	6.39	10.76	0.1008	0.0720
0.73 - 0.71	1004	1004	100.0	4.52	6.07	9.90	0.1093	0.0800
0.71 - 0.70	556	556	100.0	4.40	5.27	9.10	0.1228	0.0929
0.70 - 0.68	1175	1176	99.9	4.22	4.39	7.30	0.1397	0.1127
0.68 - 0.67	633	633	100.0	4.04	3.86	6.33	0.1604	0.1348
0.67 - 0.66	703	703	100.0	3.98	3.91	6.28	0.1658	0.1396
0.66 - 0.65	768	790	97.2	3.71	3.54	5.45	0.1784	0.1643
0.75 - 0.65	5734	5757	99.6	4.26	4.85	7.99	0.1294	0.1047
Inf - 0.65	16472	16507	99.8	5.79	14.54	19.98	0.0505	0.0343

The high residual electron density (highest peak: 1.82 at 0.77 Å from Bi1 and deepest hole: -1.88 at 0.64 Å from Bi1) could possibly be caused by anharmonic displacement of the Bi atom.

Complete .cif-data of the compound are avaliable under the CCDC number **CCDC- 2118151**.

Table S8. Crystal data and structure refinement.

Identification code	14049
Empirical formula	$C_{40} H_{28} Bi_2 F_6 O_8 S_4$
Color	colourless
Formula weight	1296.82 g·mol^{-1}
Temperature	100(2) K
Wavelength	0.71073 Å
Crystal system	Monoclinic
Space group	$P2_1$/n, (No. 14)
Unit cell dimensions	a = 14.3018(9) Å α= 90°.
	b = 18.3091(10) Å β= 90.399(6)°.
	c = 16.0212(11) Å γ = 90°.
Volume	4195.1(5) Å^3
Z	4
Density (calculated)	2.053 Mg·m^{-3}
Absorption coefficient	8.655 mm^{-1}
F(000)	2464 e
Crystal size	0.10 x 0.10 x 0.06 mm^3
θ range for data collection	2.642 to 32.577°.
Index ranges	-21 ≤ h ≤ 21, -27≤ k ≤ 27, -24 ≤ l ≤ 24
Reflections collected	91024
Independent reflections	15278 [R_{int} = 0.0510]
Reflections with I>2σ(I)	12773
Completeness to θ = 25.242°	99.9 %
Absorption correction	Gaussian
Max. and min. transmission	0.64782 and 0.40067
Refinement method	Full-matrix least-squares on F^2
Data / restraints / parameters	15278 / 0 / 571
Goodness-of-fit on F^2	1.069
Final R indices [I>2σ(I)]	R_1 = 0.0296 wR^2 = 0.0590
R indices (all data)	R_1 = 0.0428 wR^2 = 0.0633
Extinction coefficient	n/a
Largest diff. peak and hole	1.821 and -1.877 e·Å^{-3}

Table S9. Bond lengths [Å] and angles [°].

Bi(2)-O(6)	2.227(2)	Bi(2)-O(7)	2.669(2)
Bi(2)-C(27)	2.260(3)	Bi(2)-C(34)	2.309(3)
Bi(1)-O(3)	2.242(2)	Bi(1)-O(2)	2.651(2)
Bi(1)-C(8)	2.283(3)	Bi(1)-C(1)	2.270(3)
S(1)-O(2)	1.461(2)	S(1)-C(2)	1.776(3)
S(1)-O(1)	1.434(2)	S(1)-C(9)	1.761(3)
S(3)-O(6)	1.557(2)	S(3)-C(21)	1.800(3)
S(3)-O(5)	1.487(3)	S(4)-O(8)	1.436(2)
S(4)-O(7)	1.463(2)	S(4)-C(28)	1.769(3)
S(4)-C(35)	1.755(3)	S(2)-O(4)	1.489(3)
S(2)-O(3)	1.547(2)	S(2)-C(15)	1.795(3)
F(2)-C(7)	1.337(4)	C(8)-C(9)	1.394(4)
C(8)-C(13)	1.395(4)	C(27)-C(28)	1.394(4)
C(27)-C(32)	1.390(4)	C(28)-C(29)	1.394(4)
C(1)-C(2)	1.388(4)	C(1)-C(6)	1.390(4)
C(39)-H(39)	0.9500	C(39)-C(34)	1.398(4)
C(39)-C(38)	1.405(4)	C(20)-H(20)	0.9500
C(20)-C(15)	1.382(5)	C(20)-C(19)	1.381(5)
F(3)-C(7)	1.333(4)	C(34)-C(35)	1.397(4)
C(2)-C(3)	1.390(4)	C(21)-C(22)	1.379(5)
C(21)-C(26)	1.390(4)	C(12)-C(11)	1.402(5)
C(12)-C(13)	1.404(4)	C(12)-C(14)	1.507(4)
C(15)-C(16)	1.385(4)	F(1)-C(7)	1.346(4)
C(36)-H(36)	0.9500	C(36)-C(37)	1.391(4)
C(36)-C(35)	1.395(4)	C(37)-H(37)	0.9500
C(37)-C(38)	1.390(5)	C(9)-C(10)	1.392(4)
C(32)-H(32)	0.9500	C(32)-C(31)	1.395(4)
C(5)-C(4)	1.395(5)	C(5)-C(6)	1.395(4)
C(5)-C(7)	1.499(5)	C(22)-H(22)	0.9500
C(22)-C(23)	1.387(5)	C(19)-H(19)	0.9500
C(19)-C(18)	1.380(5)	C(4)-H(4)	0.9500
C(4)-C(3)	1.386(5)	C(25)-H(25)	0.9500
C(25)-C(26)	1.386(5)	C(25)-C(24)	1.380(5)
C(6)-H(6)	0.9500	C(26)-H(26)	0.9500
C(18)-H(18)	0.9500	C(18)-C(17)	1.379(5)
C(11)-H(11)	0.9500	C(11)-C(10)	1.378(4)
C(16)-H(16)	0.9500	C(16)-C(17)	1.391(5)

C(13)-H(13)	0.9500	C(10)-H(10)	0.9500
C(3)-H(3)	0.9500	C(38)-C(40)	1.506(4)
C(17)-H(17)	0.9500	C(23)-H(23)	0.9500
C(23)-C(24)	1.380(6)	F(4A)-C(33)	1.350(4)
C(31)-C(30)	1.393(5)	C(31)-C(33)	1.506(5)
F(5A)-C(33)	1.315(5)	C(29)-H(29)	0.9500
C(29)-C(30)	1.387(5)	C(14)-H(14A)	0.9800
C(14)-H(14B)	0.9800	C(14)-H(14C)	0.9800
C(30)-H(30)	0.9500	C(33)-H(33A)	0.9800
C(33)-H(33B)	0.9800	C(33)-H(33C)	0.9800
C(33)-F(6A)	1.314(5)	C(24)-H(24)	0.9500
C(40)-H(40A)	0.9800	C(40)-H(40B)	0.9800
C(40)-H(40C)	0.9800	C(40)-F(5B)	1.126(12)
C(40)-F(4B)	1.410(13)	C(40)-F(6B)	1.393(13)

O(6)-Bi(2)-O(7)	150.23(7)	O(6)-Bi(2)-C(27)	83.60(9)
O(6)-Bi(2)-C(34)	89.43(9)	C(27)-Bi(2)-O(7)	72.20(9)
C(27)-Bi(2)-C(34)	85.25(10)	C(34)-Bi(2)-O(7)	71.88(9)
O(3)-Bi(1)-O(2)	148.00(8)	O(3)-Bi(1)-C(8)	88.95(10)
O(3)-Bi(1)-C(1)	81.75(10)	C(8)-Bi(1)-O(2)	72.05(9)
C(1)-Bi(1)-O(2)	71.73(9)	C(1)-Bi(1)-C(8)	86.14(10)
O(2)-S(1)-C(2)	104.06(13)	O(2)-S(1)-C(9)	
105.00(14)	O(1)-S(1)-O(2)	119.94(15)	O(1)-
S(1)-C(2)	110.57(15)	O(1)-S(1)-C(9)	
111.47(14)	C(9)-S(1)-C(2)	104.49(14)	O(6)-
S(3)-C(21)	100.56(13)	O(5)-S(3)-O(6)	
108.20(14)	O(5)-S(3)-C(21)	102.91(15)	O(8)-
S(4)-O(7)	119.18(14)	O(8)-S(4)-C(28)	
111.28(14)	O(8)-S(4)-C(35)	111.49(14)	O(7)-
S(4)-C(28)	105.09(13)	O(7)-S(4)-C(35)	
105.15(14)	C(35)-S(4)-C(28)	103.25(14)	O(4)-
S(2)-O(3)	108.30(14)	O(4)-S(2)-C(15)	
103.22(15)	O(3)-S(2)-C(15)	101.36(14)	S(3)-
O(6)-Bi(2)	119.07(13)	S(2)-O(3)-Bi(1)	
118.95(13)	S(1)-O(2)-Bi(1)	99.25(11)	C(9)-
C(8)-Bi(1)	117.1(2)	C(9)-C(8)-C(13)	118.0(3)
C(13)-C(8)-Bi(1)	124.9(2)	S(4)-O(7)-Bi(2)	98.90(11)
C(28)-C(27)-Bi(2)	117.9(2)	C(32)-C(27)-Bi(2)	123.9(2)
C(32)-C(27)-C(28)	118.1(3)	C(27)-C(28)-S(4)	115.4(2)

C(29)-C(28)-S(4)	121.8(2)	C(29)-C(28)-C(27)	122.7(3)
C(2)-C(1)-Bi(1)	118.0(2)	C(2)-C(1)-C(6)	118.0(3)
C(6)-C(1)-Bi(1)	123.9(2)	C(34)-C(39)-H(39)	119.5
C(34)-C(39)-C(38)	121.0(3)	C(38)-C(39)-H(39)	119.5
C(15)-C(20)-H(20)	120.4	C(19)-C(20)-H(20)	120.4
C(19)-C(20)-C(15)	119.1(3)	C(39)-C(34)-Bi(2)	126.4(2)
C(35)-C(34)-Bi(2)	116.3(2)	C(35)-C(34)-C(39)	117.3(3)
C(1)-C(2)-S(1)	114.9(2)	C(1)-C(2)-C(3)	123.2(3)
C(3)-C(2)-S(1)	121.8(2)	C(22)-C(21)-S(3)	121.2(2)
C(22)-C(21)-C(26)	121.1(3)	C(26)-C(21)-S(3)	117.7(2)
C(11)-C(12)-C(13)	118.6(3)	C(11)-C(12)-C(14)	121.3(3)
C(13)-C(12)-C(14)	120.0(3)	C(20)-C(15)-S(2)	120.8(2)
C(20)-C(15)-C(16)	121.0(3)	C(16)-C(15)-S(2)	118.2(3)
C(37)-C(36)-H(36)	120.9	C(37)-C(36)-C(35)	118.1(3)
C(35)-C(36)-H(36)	120.9	C(36)-C(37)-H(37)	119.6
C(38)-C(37)-C(36)	120.8(3)	C(38)-C(37)-H(37)	119.6
C(8)-C(9)-S(1)	115.7(2)	C(10)-C(9)-S(1)	121.7(2)
C(10)-C(9)-C(8)	122.6(3)	C(27)-C(32)-H(32)	120.0
C(27)-C(32)-C(31)	119.9(3)	C(31)-C(32)-H(32)	120.0
C(4)-C(5)-C(6)	121.2(3)	C(4)-C(5)-C(7)	120.1(3)
C(6)-C(5)-C(7)	118.7(3)	C(21)-C(22)-H(22)	120.4
C(21)-C(22)-C(23)	119.2(3)	C(23)-C(22)-H(22)	120.4
C(20)-C(19)-H(19)	119.7	C(18)-C(19)-C(20)	120.5(3)
C(18)-C(19)-H(19)	119.7	C(5)-C(4)-H(4)	120.2
C(3)-C(4)-C(5)	119.7(3)	C(3)-C(4)-H(4)	120.2
C(26)-C(25)-H(25)	120.1	C(24)-C(25)-H(25)	120.1
C(24)-C(25)-C(26)	119.7(3)	C(34)-C(35)-S(4)	116.4(2)
C(36)-C(35)-S(4)	120.6(2)	C(36)-C(35)-C(34)	123.1(3)
C(1)-C(6)-C(5)	119.7(3)	C(1)-C(6)-H(6)	120.2
C(5)-C(6)-H(6)	120.2	C(21)-C(26)-H(26)	120.4
C(25)-C(26)-C(21)	119.2(3)	C(25)-C(26)-H(26)	120.4
C(19)-C(18)-H(18)	119.9	C(17)-C(18)-C(19)	120.3(3)
C(17)-C(18)-H(18)	119.9	C(12)-C(11)-H(11)	119.2
C(10)-C(11)-C(12)	121.6(3)	C(10)-C(11)-H(11)	119.2
C(15)-C(16)-H(16)	120.4	C(15)-C(16)-C(17)	119.3(3)
C(17)-C(16)-H(16)	120.4	F(2)-C(7)-F(1)	106.1(3)
F(2)-C(7)-C(5)	113.3(3)	F(3)-C(7)-F(2)	106.1(3)
F(3)-C(7)-F(1)	106.2(3)	F(3)-C(7)-C(5)	113.1(3)
F(1)-C(7)-C(5)	111.4(3)	C(8)-C(13)-C(12)	121.0(3)

C(8)-C(13)-H(13)	119.5	C(12)-C(13)-H(13)	119.5
C(9)-C(10)-H(10)	120.9	C(11)-C(10)-C(9)	118.2(3)
C(11)-C(10)-H(10)	120.9	C(2)-C(3)-H(3)	120.9
C(4)-C(3)-C(2)	118.2(3)	C(4)-C(3)-H(3)	120.9
C(39)-C(38)-C(40)	120.4(3)	C(37)-C(38)-C(39)	119.7(3)
C(37)-C(38)-C(40)	119.8(3)	C(18)-C(17)-C(16)	119.8(3)
C(18)-C(17)-H(17)	120.1	C(16)-C(17)-H(17)	120.1
C(22)-C(23)-H(23)	120.0	C(24)-C(23)-C(22)	119.9(4)
C(24)-C(23)-H(23)	120.0	C(32)-C(31)-C(33)	118.8(3)
C(30)-C(31)-C(32)	121.0(3)	C(30)-C(31)-C(33)	120.1(3)
C(28)-C(29)-H(29)	120.8	C(30)-C(29)-C(28)	118.3(3)
C(30)-C(29)-H(29)	120.8	C(12)-C(14)-H(14A)	109.5
C(12)-C(14)-H(14B)	109.5	C(12)-C(14)-H(14C)	109.5
H(14A)-C(14)-H(14B)	109.5	H(14A)-C(14)-H(14C)	109.5
H(14B)-C(14)-H(14C)	109.5	C(31)-C(30)-H(30)	120.0
C(29)-C(30)-C(31)	119.9(3)	C(29)-C(30)-H(30)	120.0
F(4A)-C(33)-C(31)	111.1(3)	C(31)-C(33)-H(33A)	109.5
C(31)-C(33)-H(33B)	109.5	C(31)-C(33)-H(33C)	109.5
F(5A)-C(33)-F(4A)	106.4(3)	F(5A)-C(33)-C(31)	111.8(3)
H(33A)-C(33)-H(33B)	109.5	H(33A)-C(33)-H(33C)	109.5
H(33B)-C(33)-H(33C)	109.5	F(6A)-C(33)-F(4A)	106.5(3)
F(6A)-C(33)-C(31)	112.9(3)	F(6A)-C(33)-F(5A)	107.8(3)
C(25)-C(24)-C(23)	120.8(3)	C(25)-C(24)-H(24)	119.6
C(23)-C(24)-H(24)	119.6	C(38)-C(40)-H(40A)	109.5
C(38)-C(40)-H(40B)	109.5	C(38)-C(40)-H(40C)	109.5
H(40A)-C(40)-H(40B)	109.5	H(40A)-C(40)-H(40C)	109.5
H(40B)-C(40)-H(40C)	109.5	F(5B)-C(40)-C(38)	116.8(7)
F(5B)-C(40)-F(4B)	115.0(9)	F(5B)-C(40)-F(6B)	113.5(9)
F(4B)-C(40)-C(38)	104.1(5)	F(6B)-C(40)-C(38)	107.7(6)
F(6B)-C(40)-F(4B)	97.6(8)		

9. References

[1] Okauchi, T.; Kuramoto, K.; Kitamura, M., Facile Preparation of Aryl Sulfides Using Palladium Catalysis under Mild Conditions. *Synlett* **2010,** *2010*, 2891–2894

[2] Dyer, J. C.; Harris, D. L.; Evans, S. A. Oxygen-17 nuclear magnetic resonance spectroscopy of sulfoxides and sulfones. Alkyl substituent-induced chemical-shift effects. *J. Org. Chem.* **1982,** *47*, 3660–3664.

[3] Gopalsamy, A.; Shi, M.; Stauffer, B.; Bahat, R.; Billiard, J.; Ponce-de-Leon, H.; Seestaller-Wehr, L.; Fukayama, S.; Mangine, A.; Moran, R.; Krishnamurthy, G.; Bodine, P. Identification of Diarylsulfone Sulfonamides as Secreted Frizzled Related Protein-1 (sFRP-1) Inhibitors. *J. Med. Chem.* **2008,** *51*, 7670–7672.

[4] Planas, O.; Peciukenas, V.; Cornella, J. Bismuth-Catalyzed Oxidative Coupling of Arylboronic Acids with Triflate and Nonaflate Salts. *J. Am. Chem. Soc.* **2020**, *142*, 11382–11387.

[5] Chen, L.; Liang, J.; Chen, Z.-Y.; Chen, J.; Yan, M.; Zhang, X.-J. A Convenient Synthesis of Sulfones via Light Promoted Coupling of Sodium Sulfinates and Aryl Halides. *Adv. Synth. Catal.* **2019**, *361*, 956–960.

[6] Slagman, S.; Jonkers, W. A.; Zuilhof, H.; Franssen, M. C. R. Elucidating the mechanism behind the laccase-mediated modification of poly(ethersulfone). *RSC. Adv.* **2018**, *8*, 27101–27110.

[7] Ke, F.; Qu, Y.; Jiang, Z.; Li, Z.; Wu, D.; Zhou, X. An Efficient Copper-Catalyzed Carbon−Sulfur Bond Formation Protocol in Water. *Org. Lett.* **2011**, *13*, 454–457.

[8] Carpino, L. A.; Gao, H. S.; Ti, G. S.; Segev, D. Thioxanthene dioxide based amino-protecting groups sensitive to pyridine bases and dipolar aprotic solvents. *J. Org. Chem.* **1989**, *54*, 5887–5897.

[9] Jurrat, M.; Maggi, L.; Lewis, W.; Ball, L. T. Modular bismacycles for the selective C–H arylation of phenols and naphthols. *Nat. Chem.* **2020**, *12*, 260–269.

[10] Davies, A. T.; Curto, J. M.; Bagley, S. W.; Willis, M. C. One-pot palladium-catalyzed synthesis of sulfonyl fluorides from aryl bromides. *Chem. Sci.*, **2017**, *8*, 1233–1237.

[11] Liu, S.; Huang. Y.; Xu, X.-H.; Qing, F.-L. Fluorosulfonylation of arenediazonium tetrafluoroborates with Na2S2O5 and N-fluorobenzenesulfonimide. *J. Fluor. Chem.* **2020**, DOI: 10.1016/j.jfluchem.2020.109653.

[12] Zhong, T.; Pang, M.-K.; Chen, Z.-D.; Zhang, B.; Wenig, J.; Lu, G. Copper-free Sandmeyer-type Reaction for the Synthesis of Sulfonyl Fluorides. *Org. Lett.* **2020**, *22*, 3072–3078.

[13] van der Zouwen, A. J.; Lohse, J.; Wieske, L. H. E.; Hohmann, K. F.; van der Vlag, R.; Witte. M. D. An in situ combinatorial methodology to synthesize and screen chemical probes. *Chem. Commun.*, **2019**, *55*, 2050–2053.

[14] Planas, O.; Wang, F.; Leutzsch, M.; Cornella, J. Fluorination of Arylboronic esters Enabled by Bismuth Redox Catalysis. *Science* **2020**, *367*, 313–317.

10. Spectral data: ^{1}H, ^{13}C and ^{19}F NMR spectra

^{1}H NMR (300 MHz, $CDCl_3$)

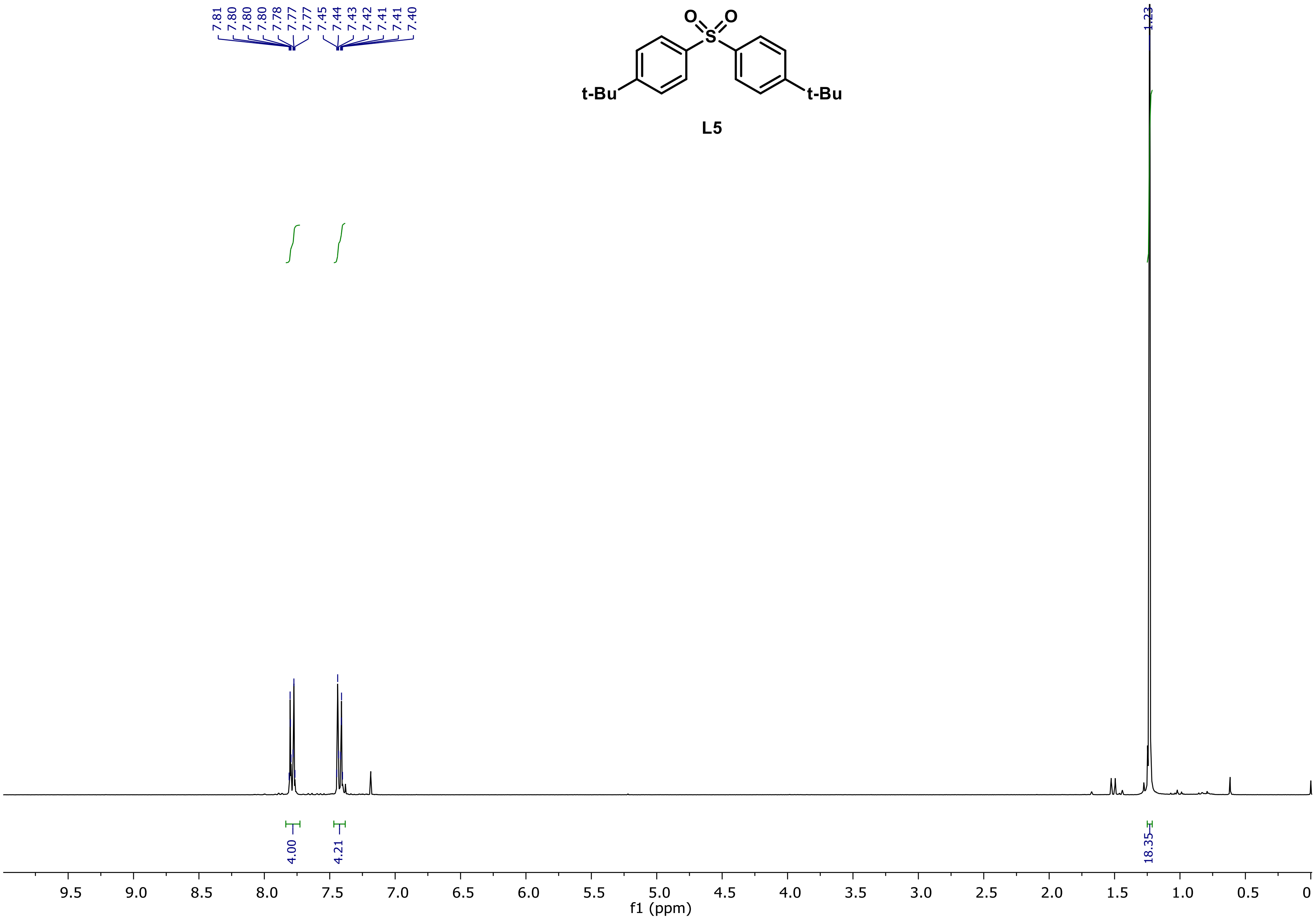

^{13}C NMR (75 MHz, $CDCl_3$)

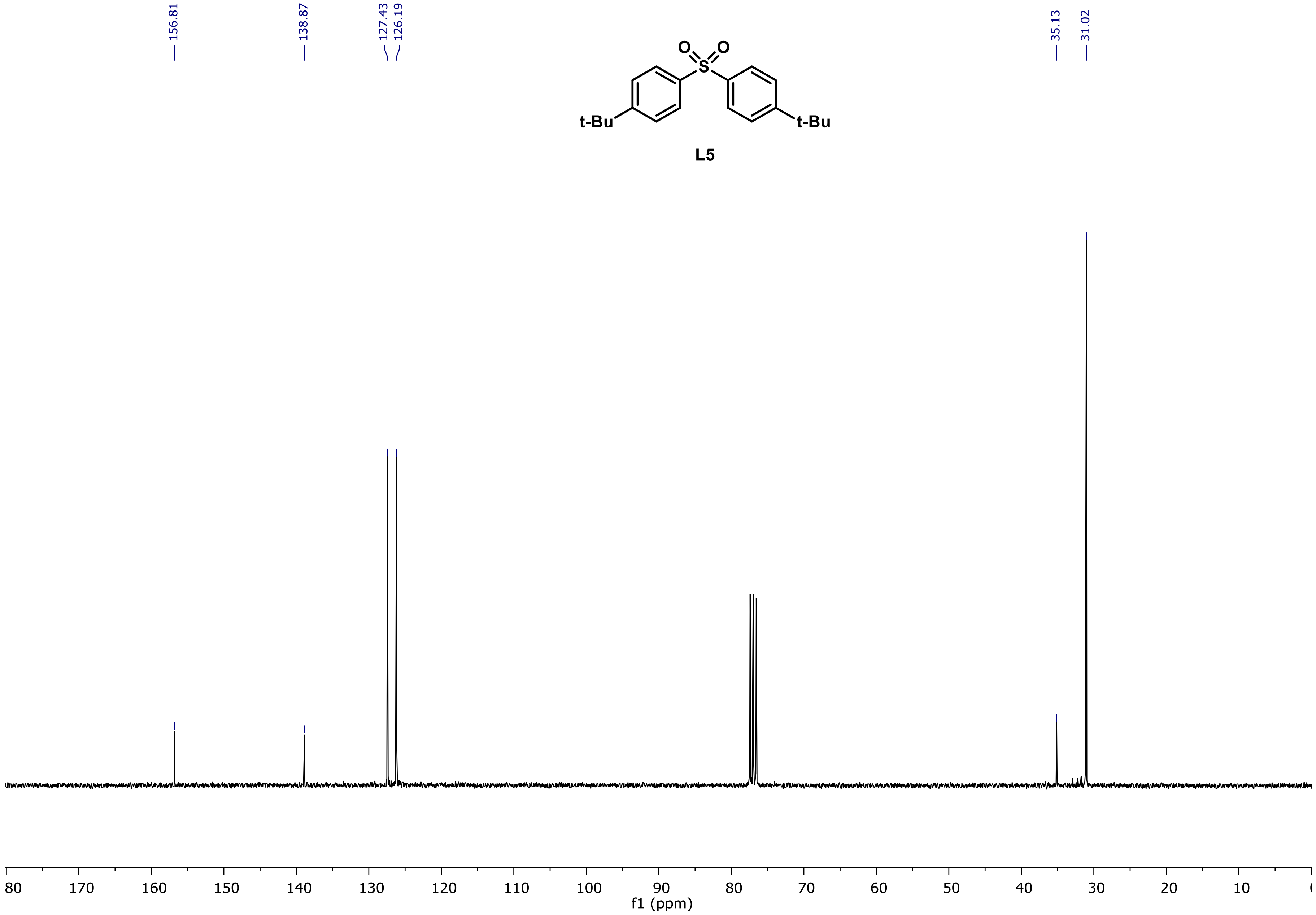

^{1}H NMR (600 MHz, $CDCl_3$)

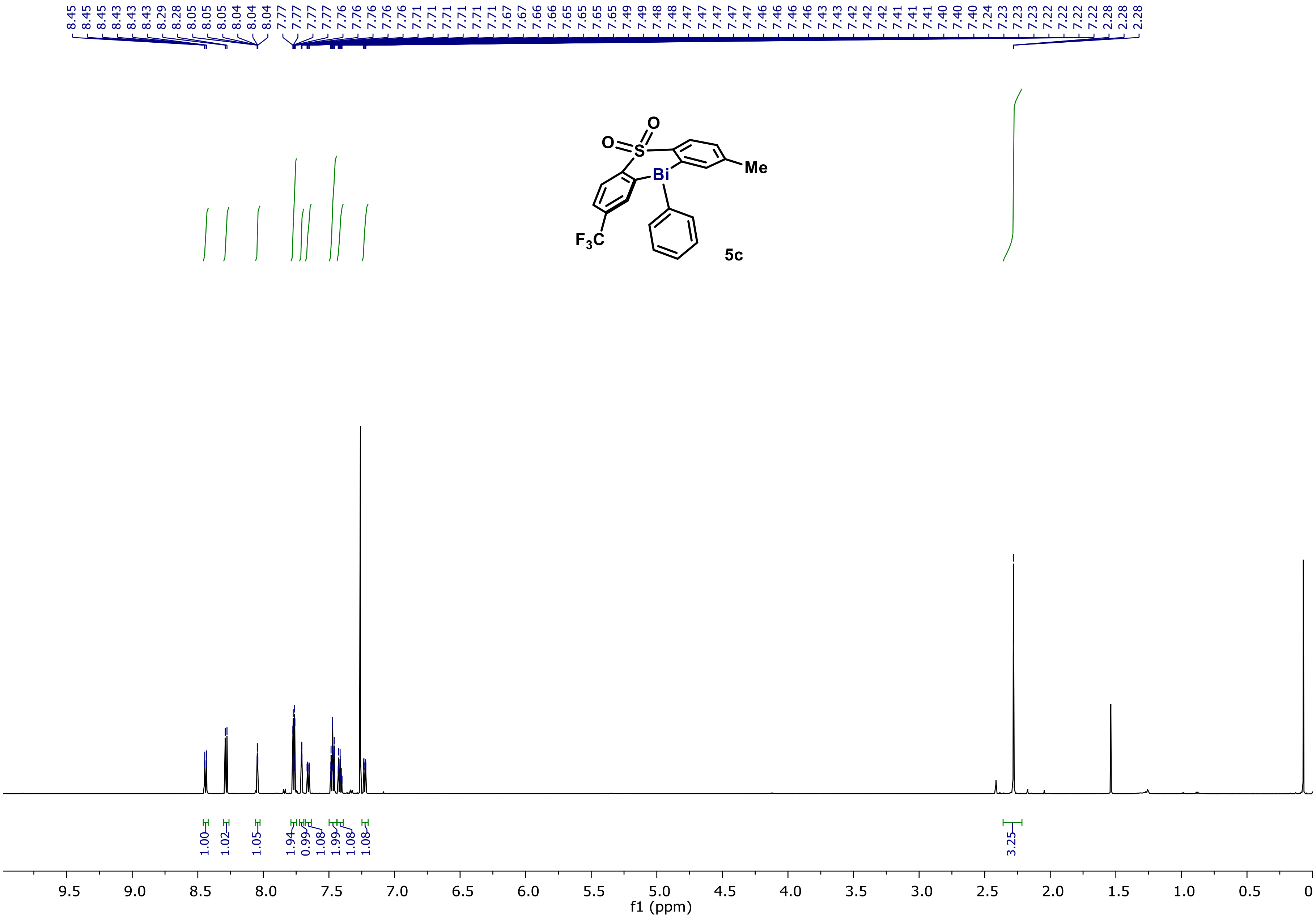

^{13}C NMR (151 MHz, $CDCl_3$)

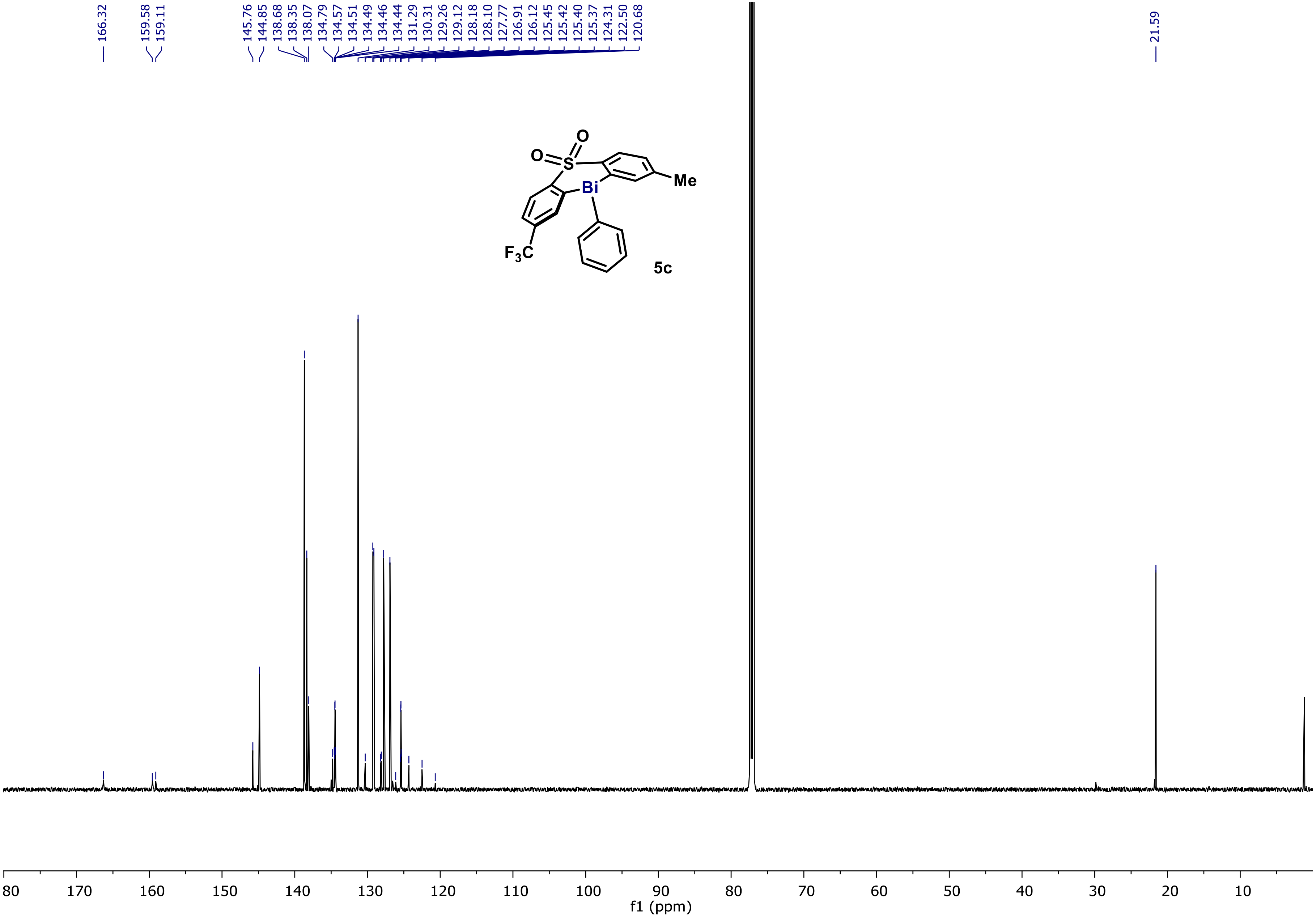

^{19}F NMR (565 MHz, $CDCl_3$)

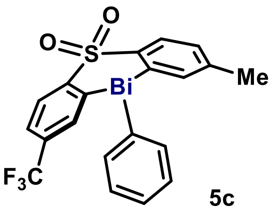

5c

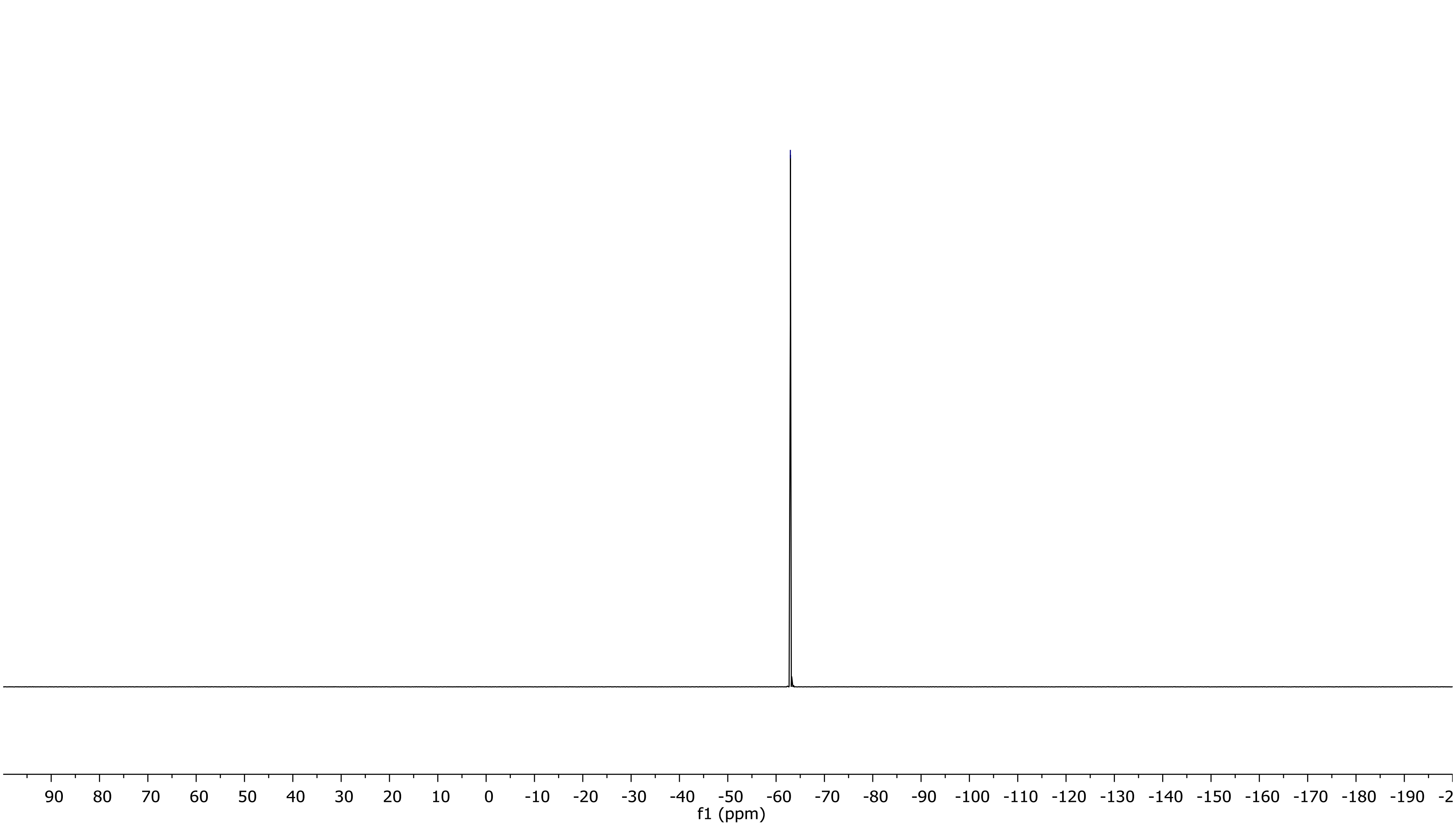

^{1}H NMR (600 MHz, CDCl$_3$)

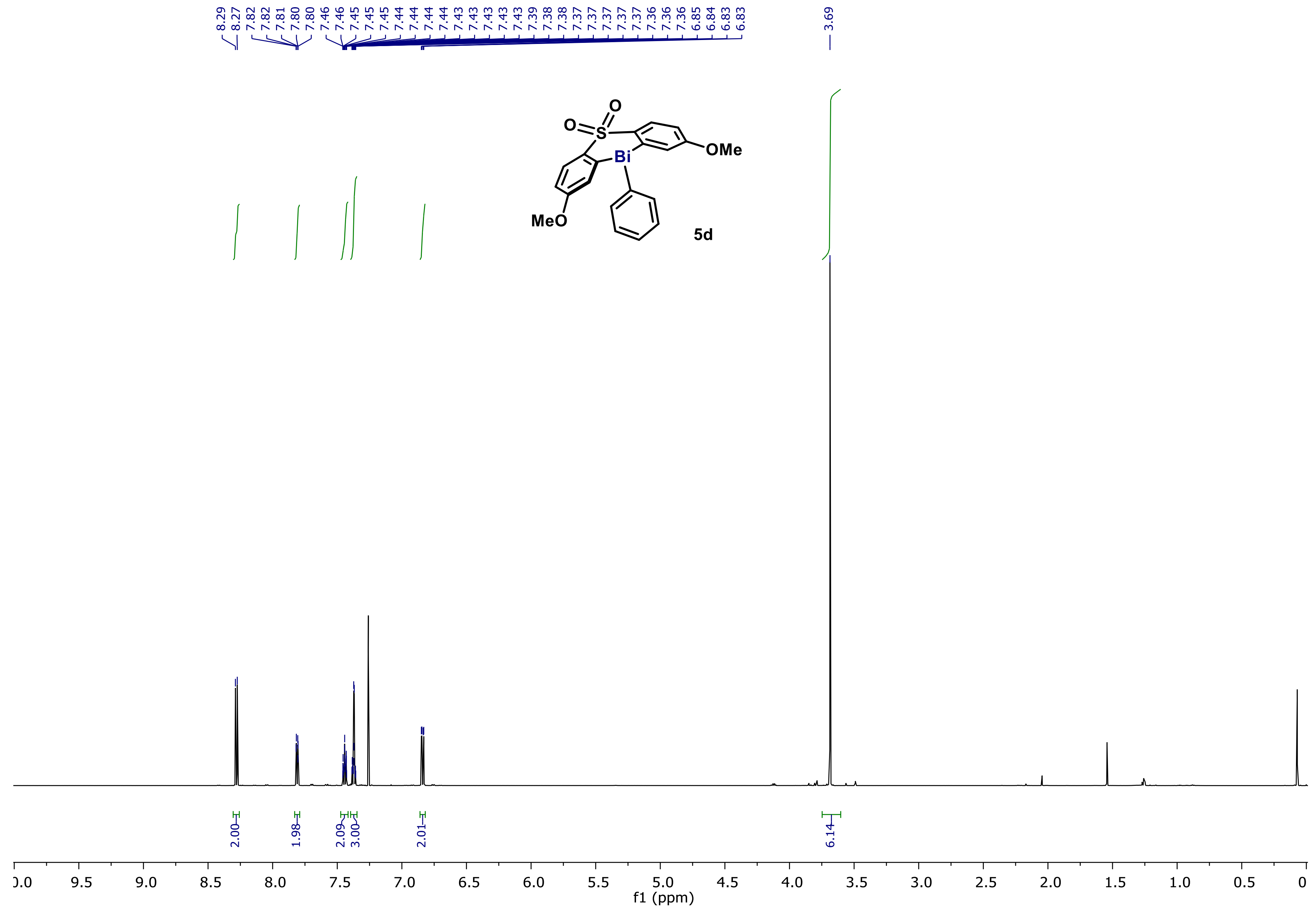

^{13}C NMR (151 MHz, $CDCl_3$)

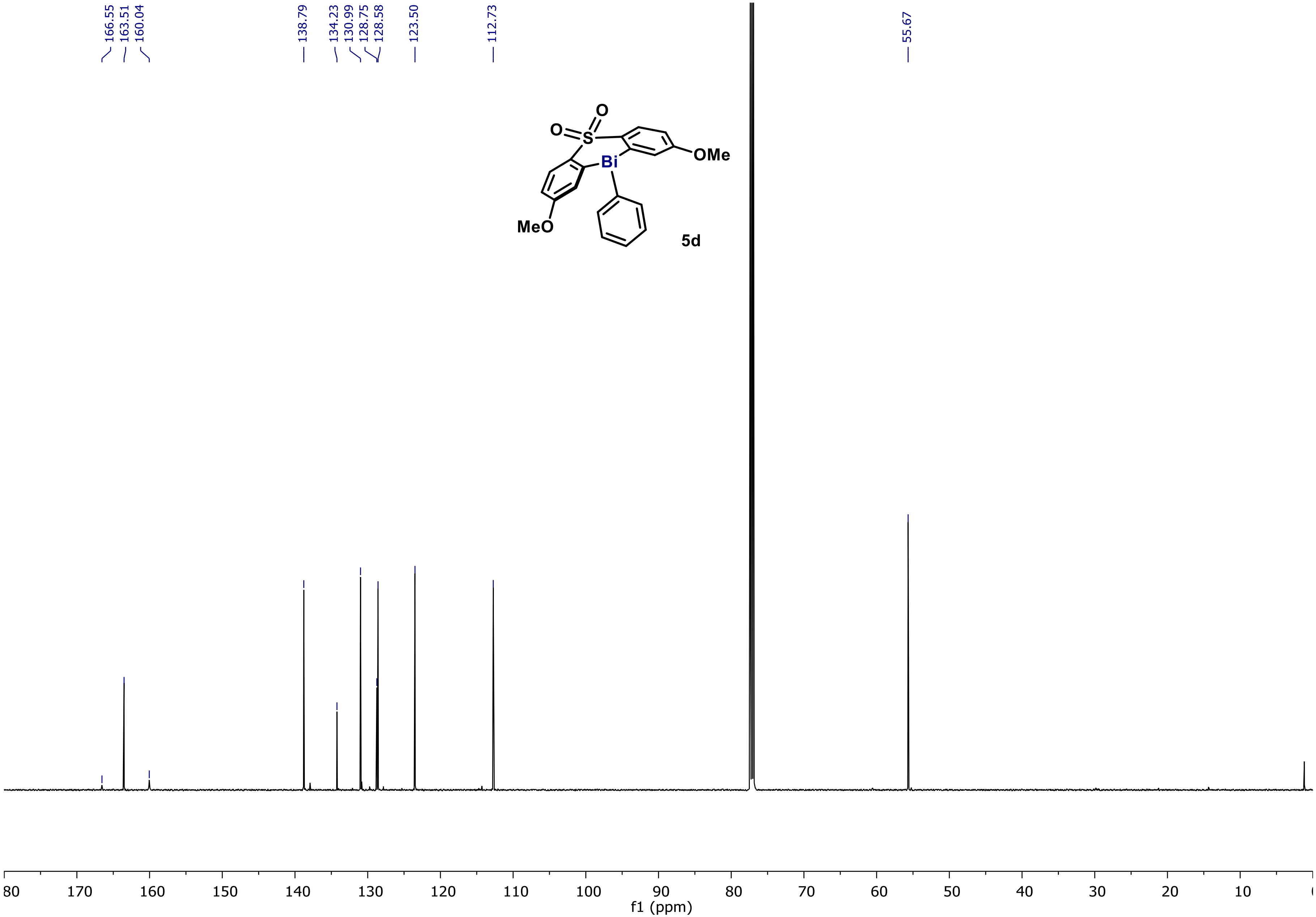

^{1}H NMR (600 MHz, $CDCl_3$)

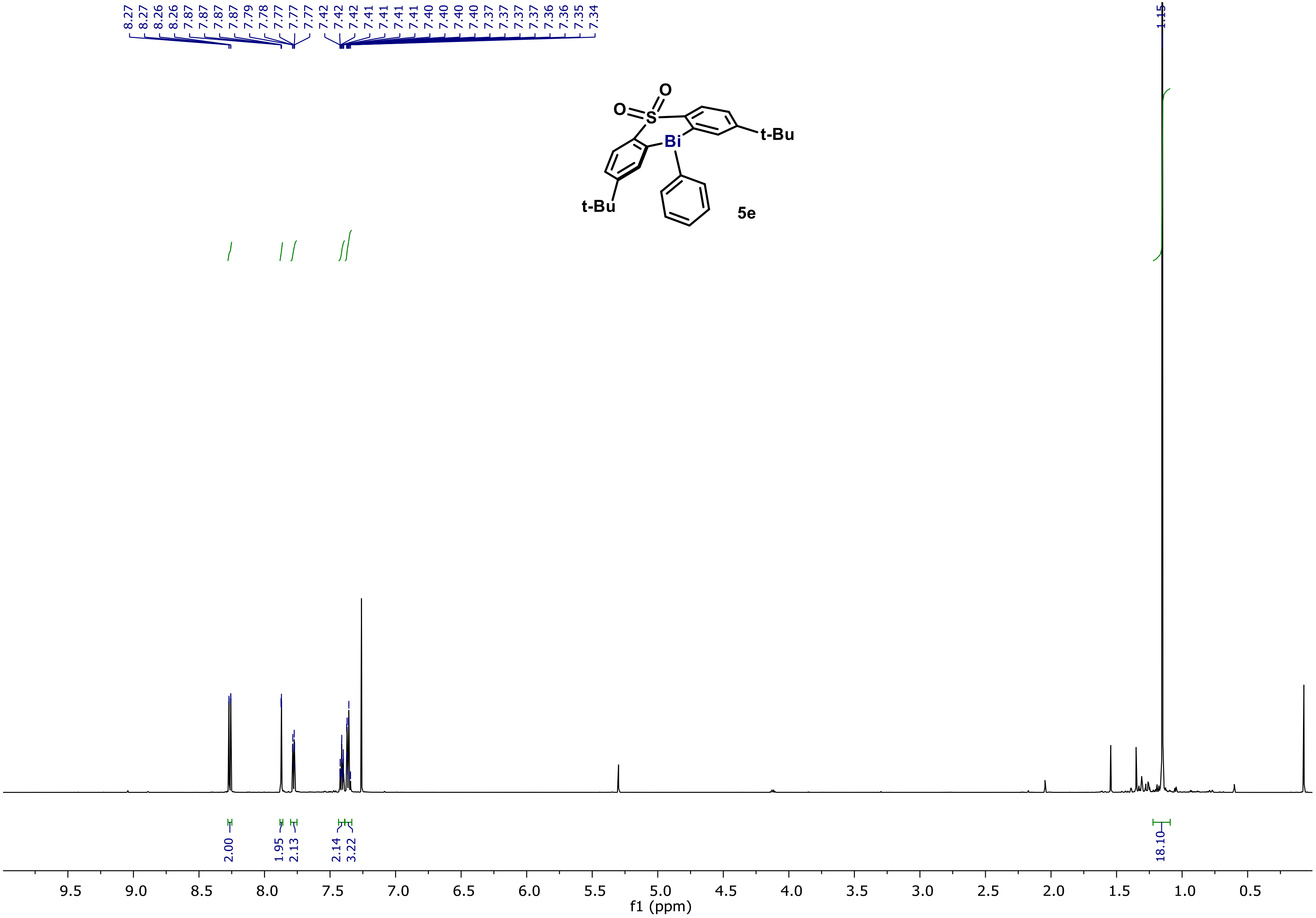

^{13}C NMR (151 MHz, CDCl$_3$)

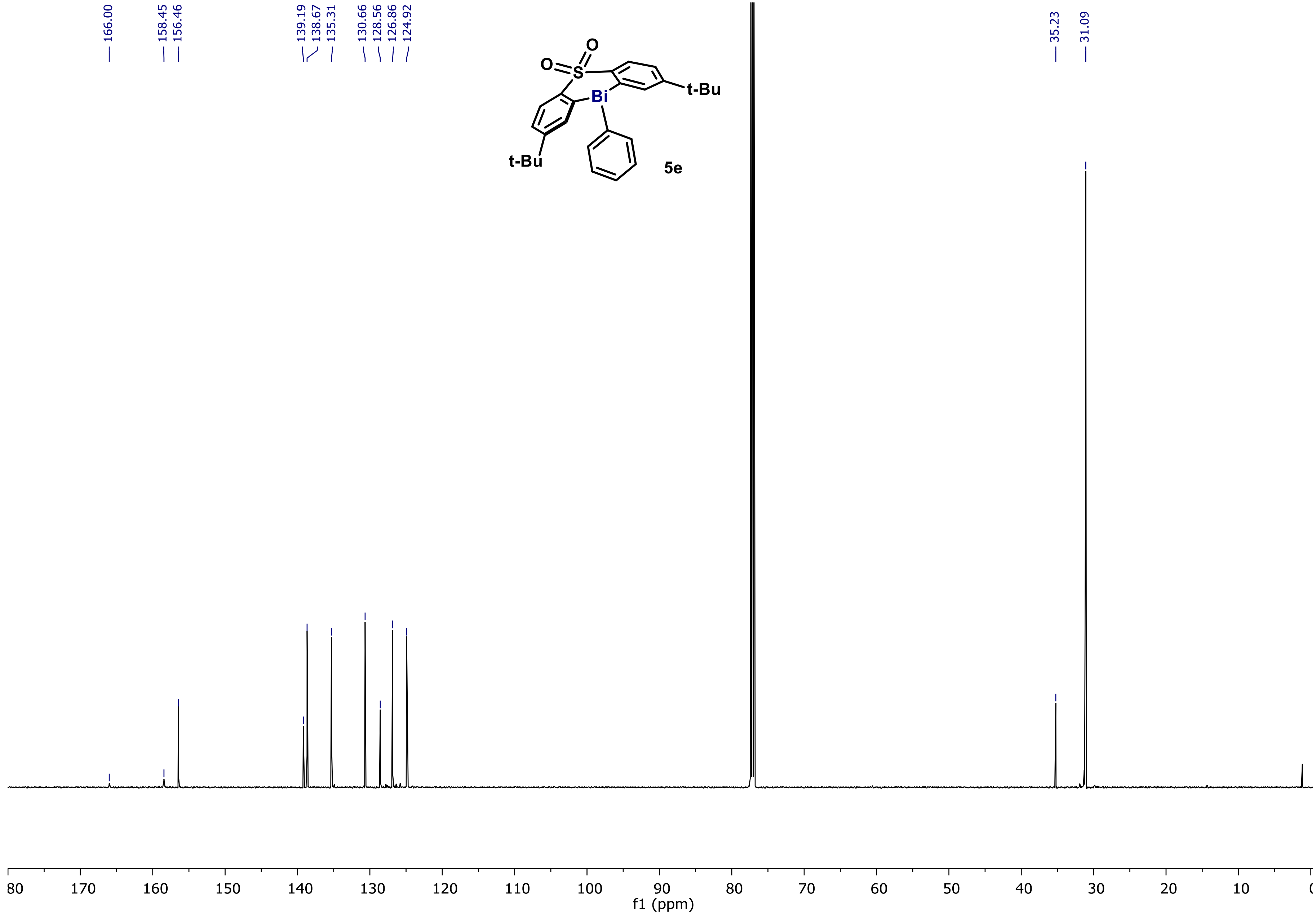

^{1}H NMR (400 MHz, CD_3CN)

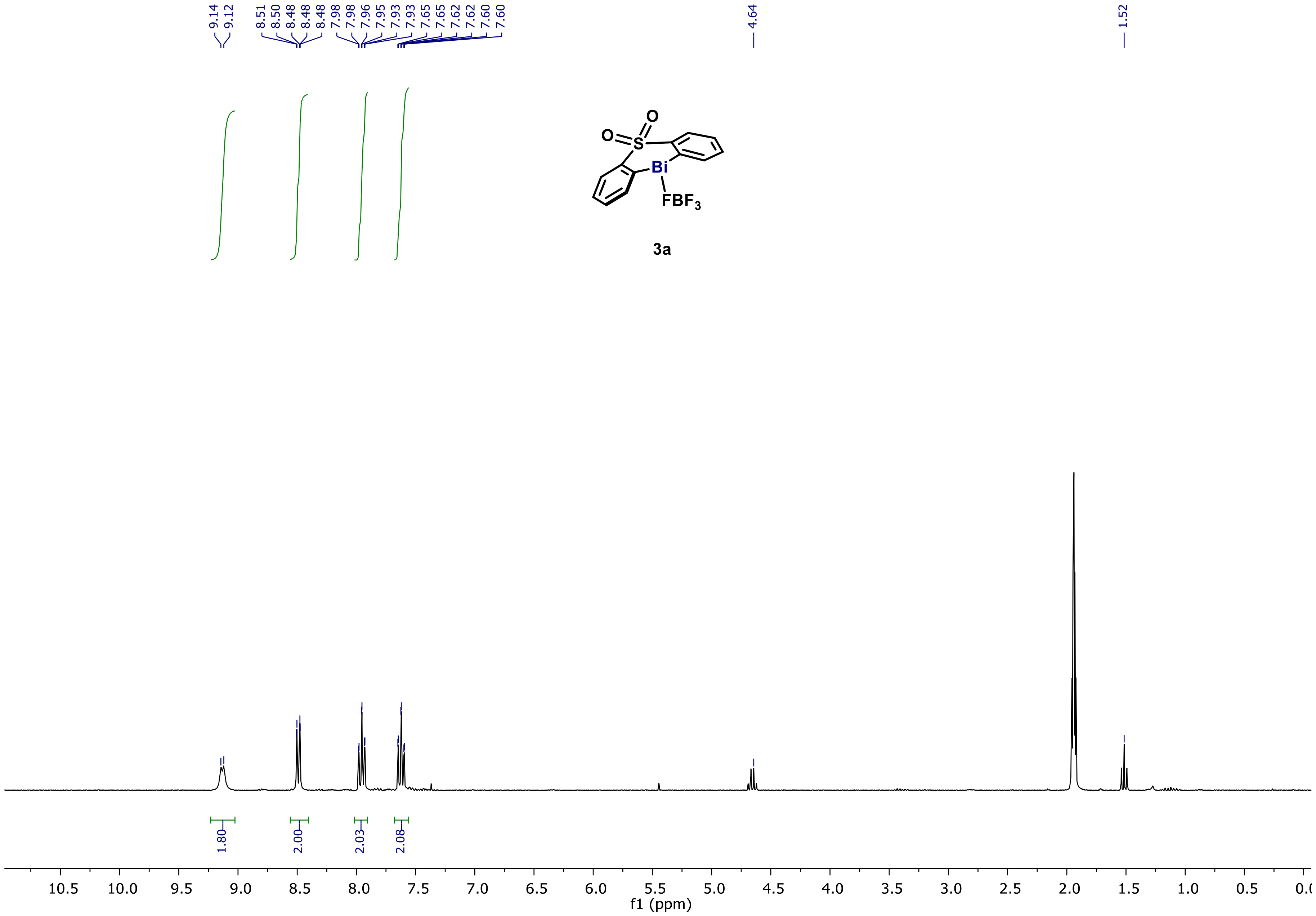

^{13}C NMR (101 MHz, CD_3CN)

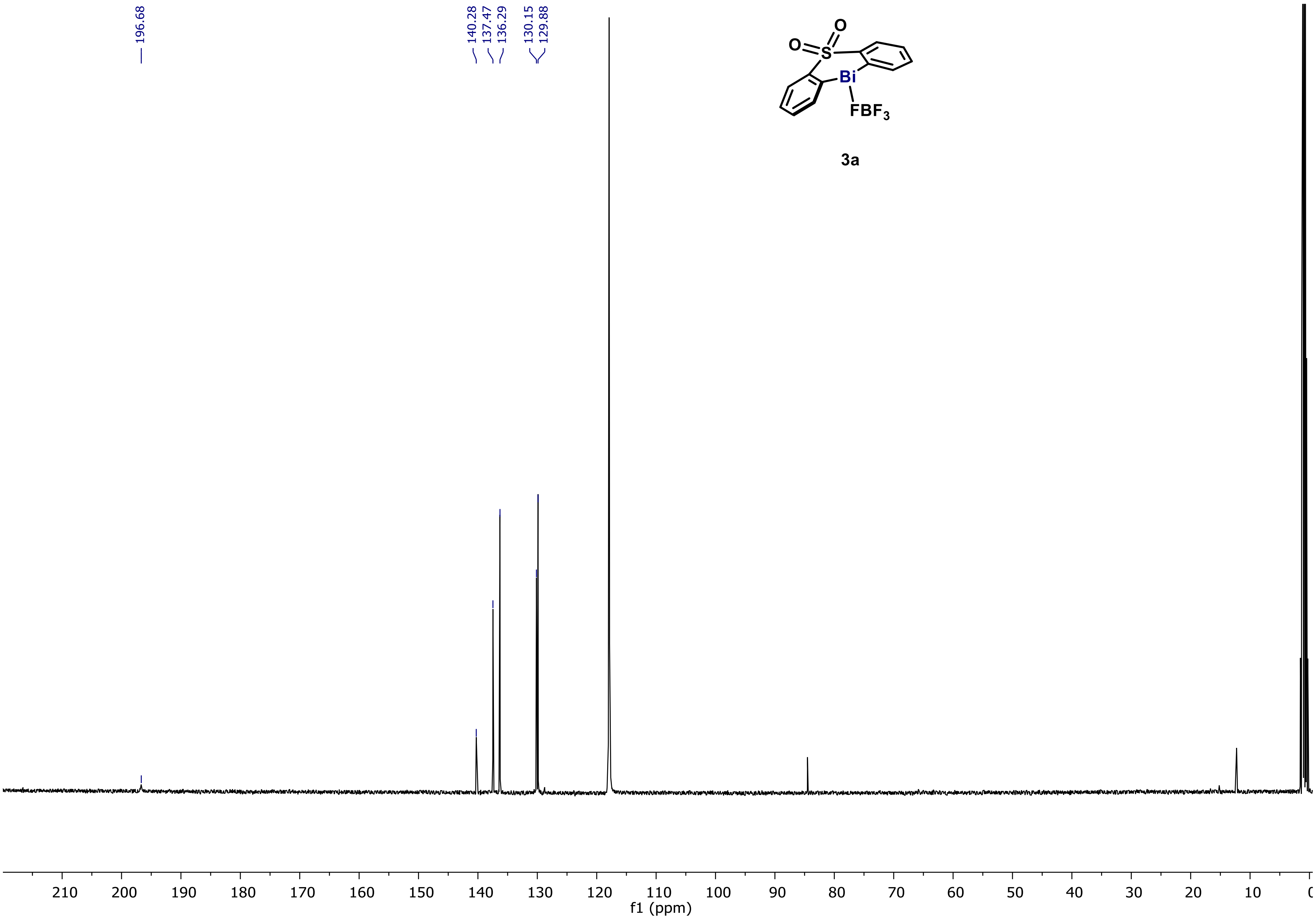

^{19}F NMR (282 MHz, CD_3CN)

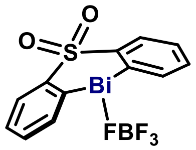

3a

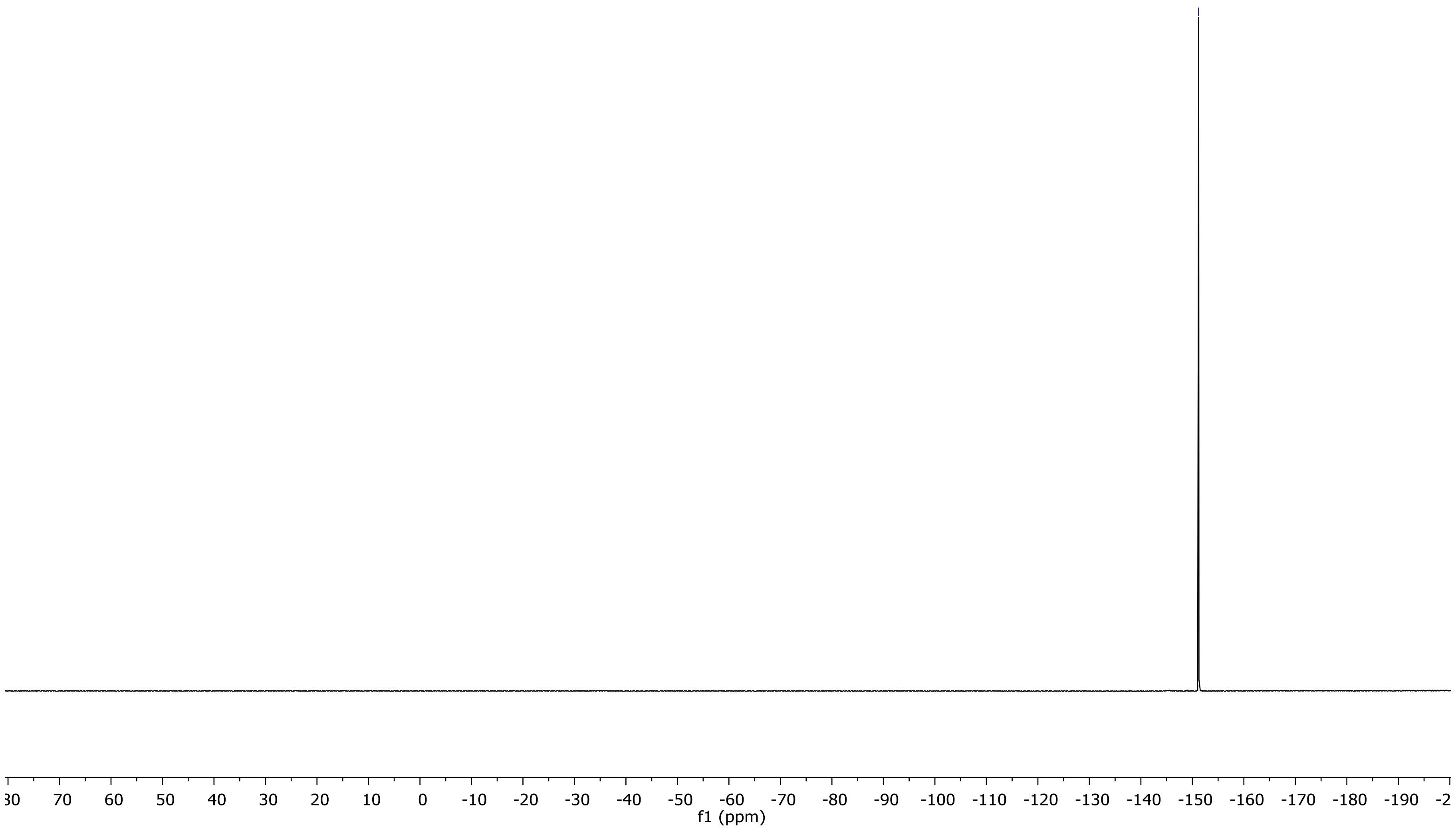

^{1}H NMR (400 MHz, CD_3CN)

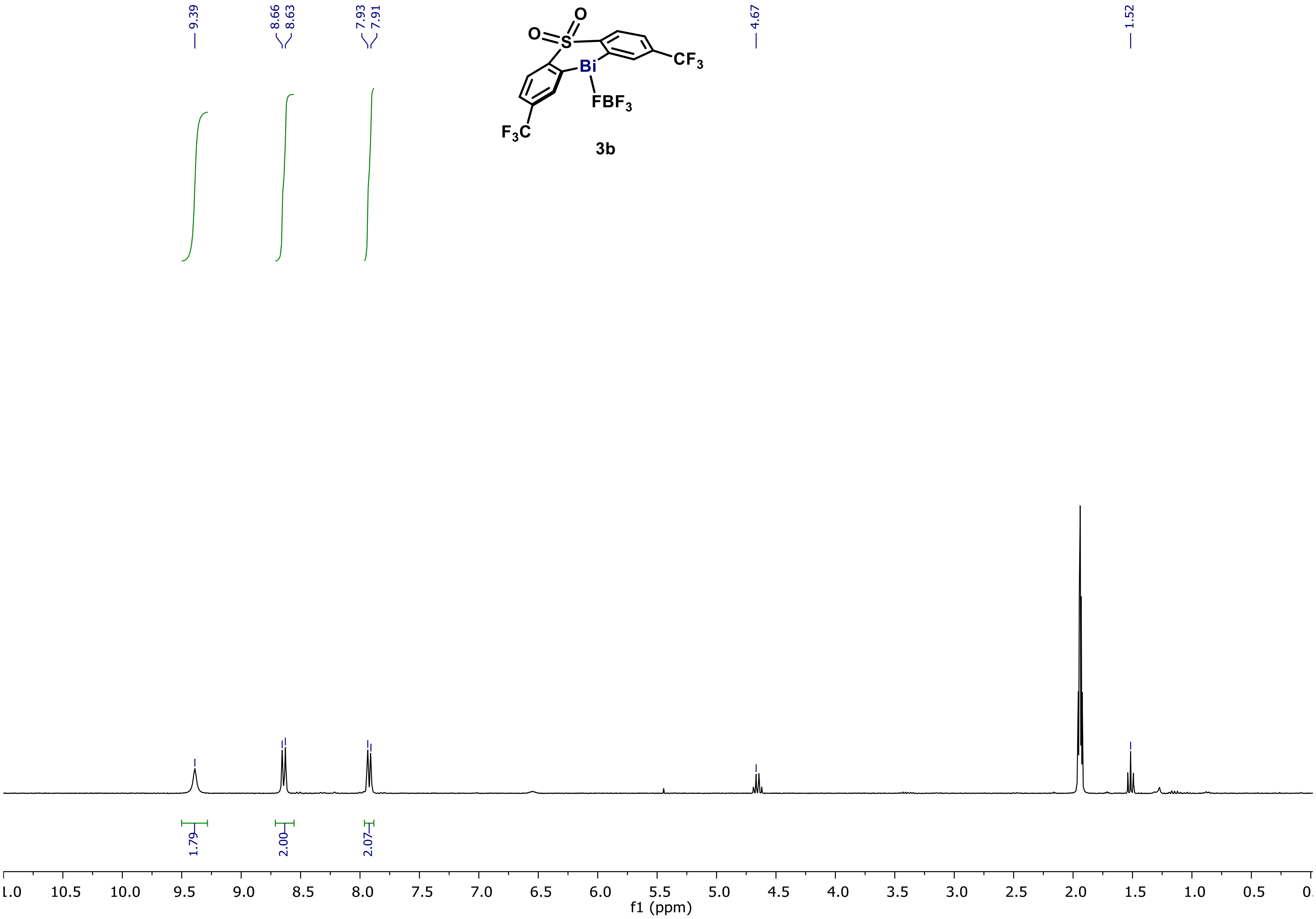

3b

^{13}C NMR (101 MHz, CD_3CN)

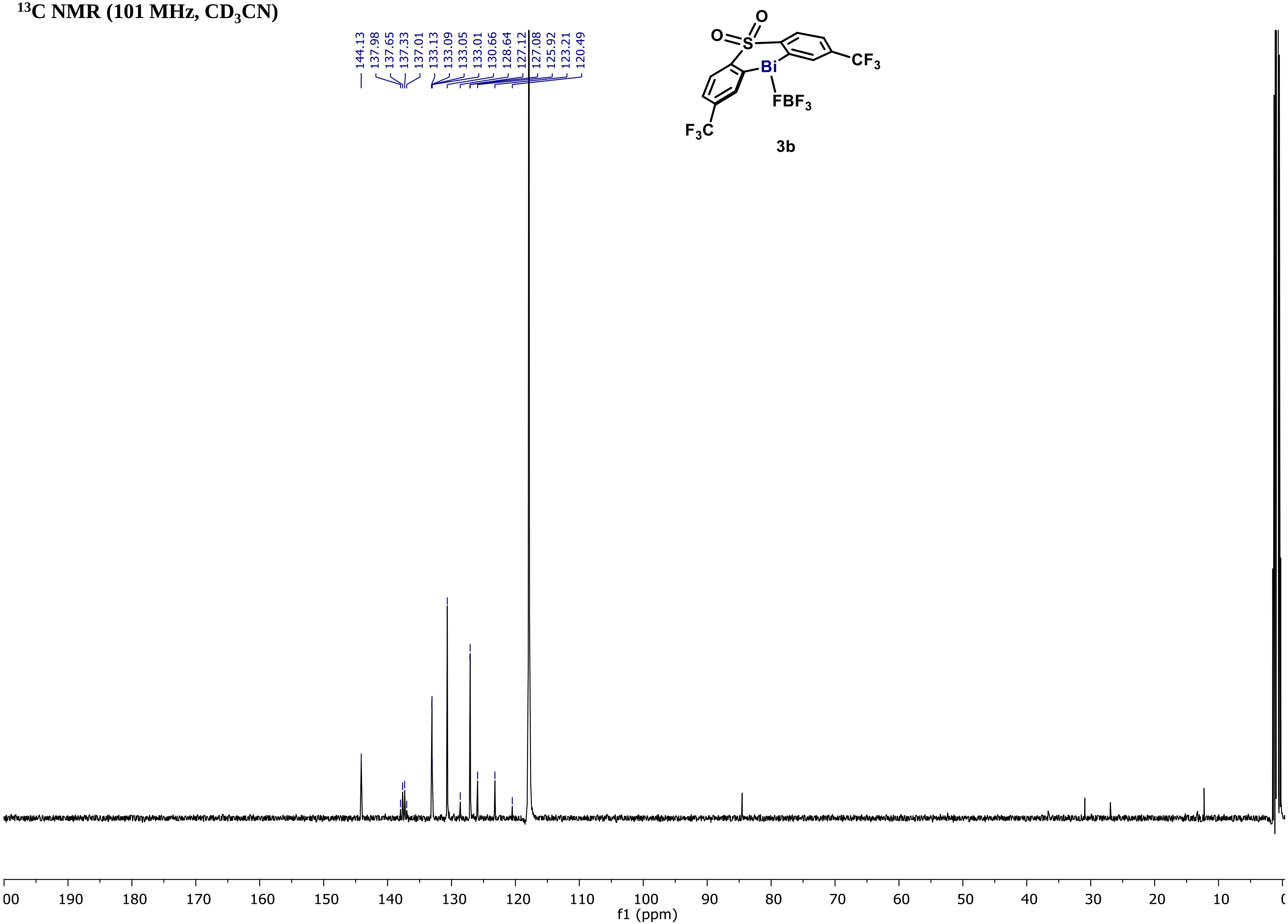

^{19}F NMR (282 MHz, CD_3CN)

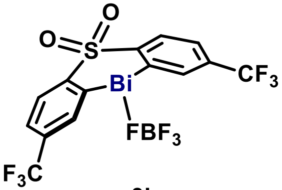

3b

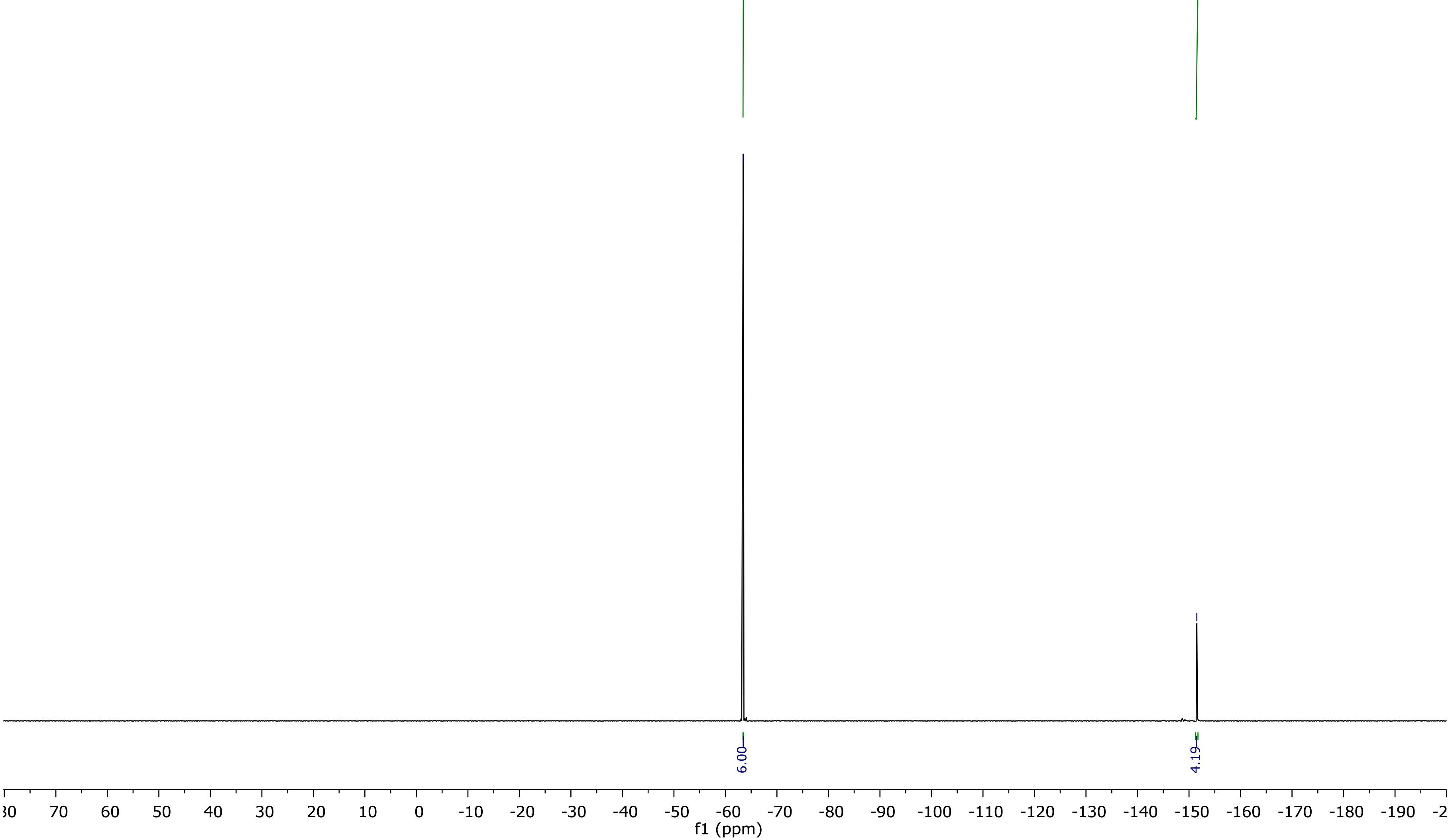

^{1}H NMR (400 MHz, CD_3CN)

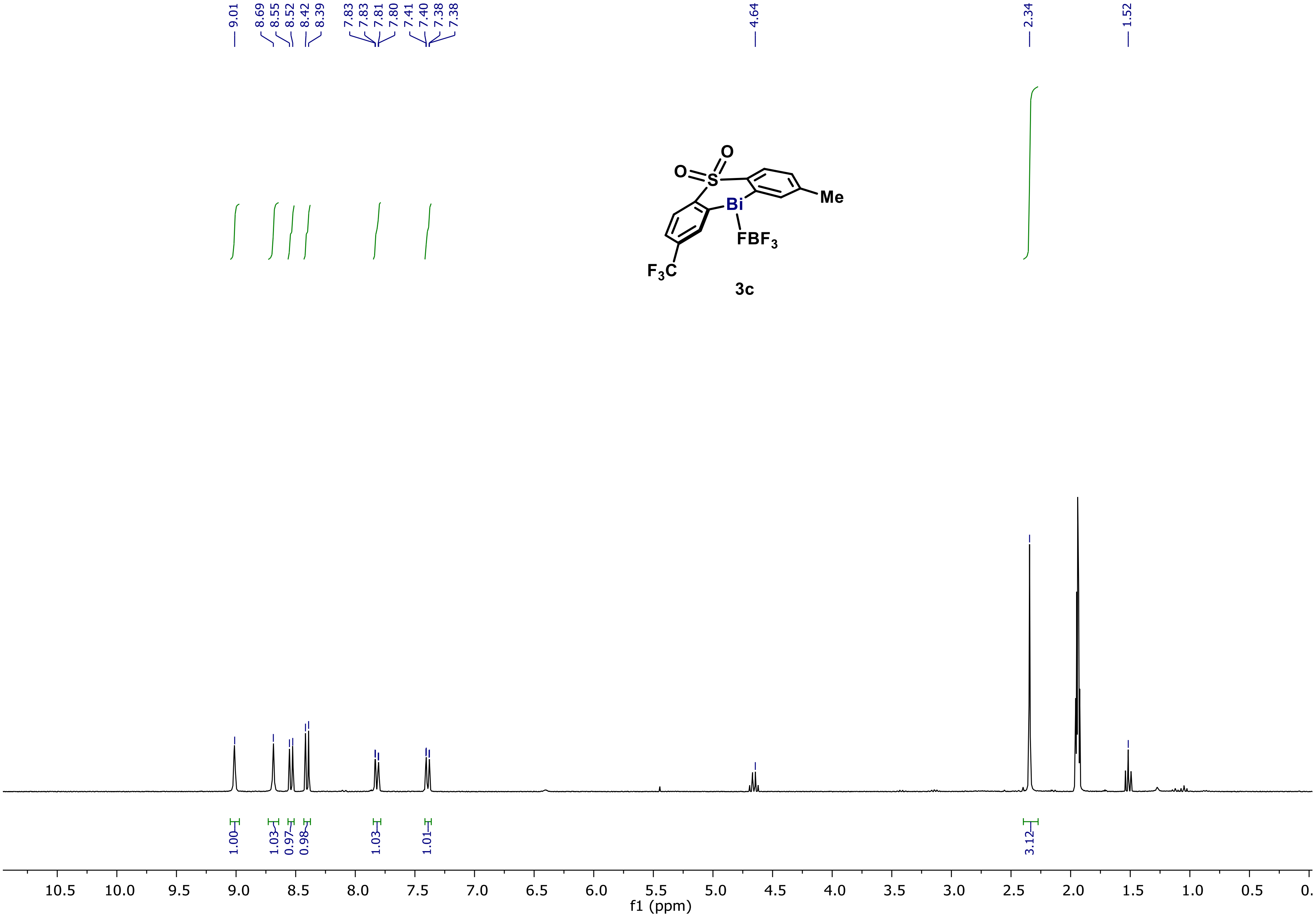

3c

^{13}C NMR (101 MHz, CD_3CN)

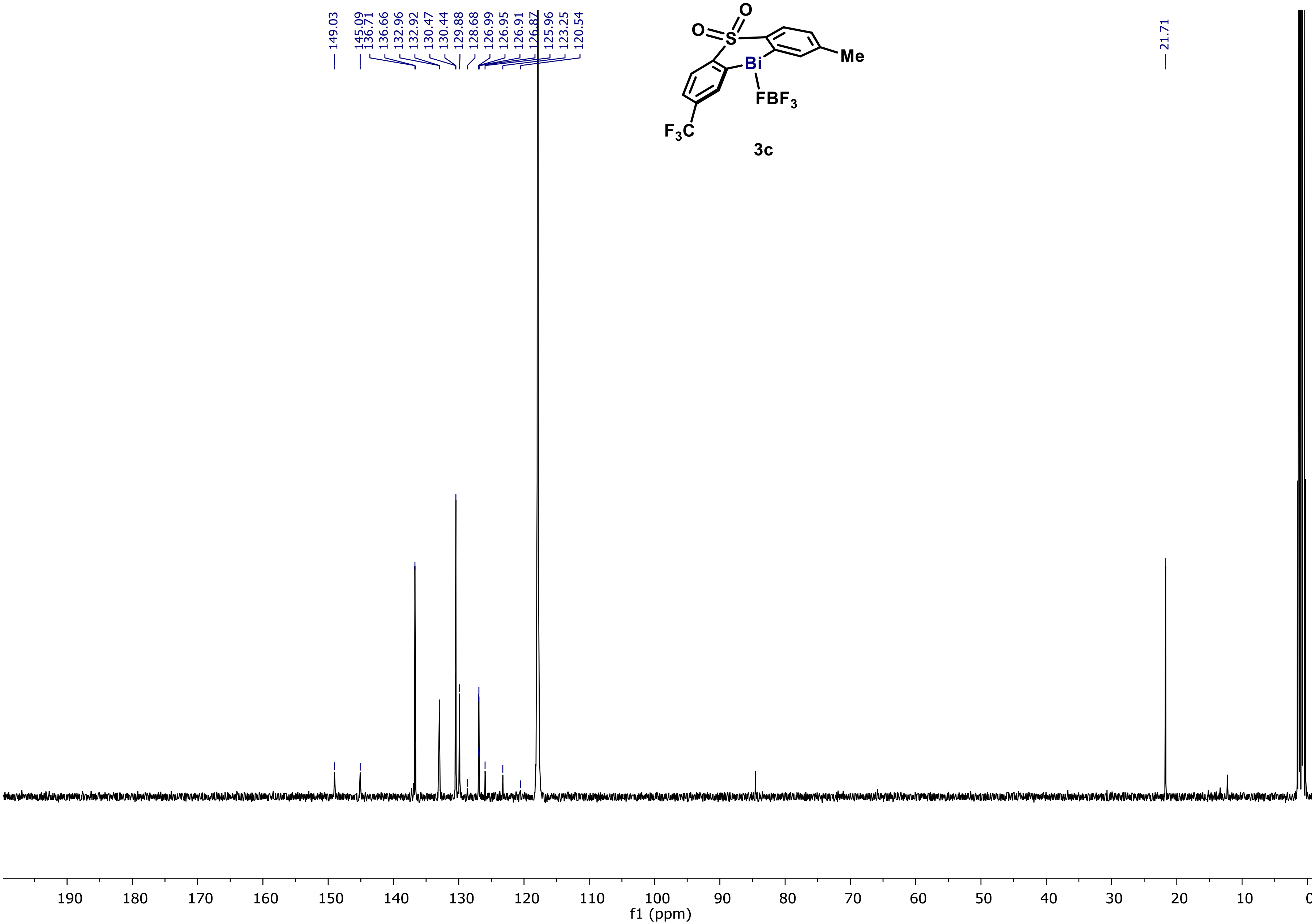

^{19}F NMR (282 MHz, CD_3CN)

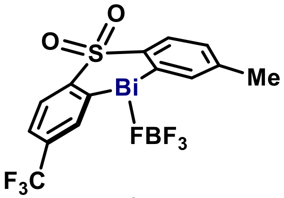

3c

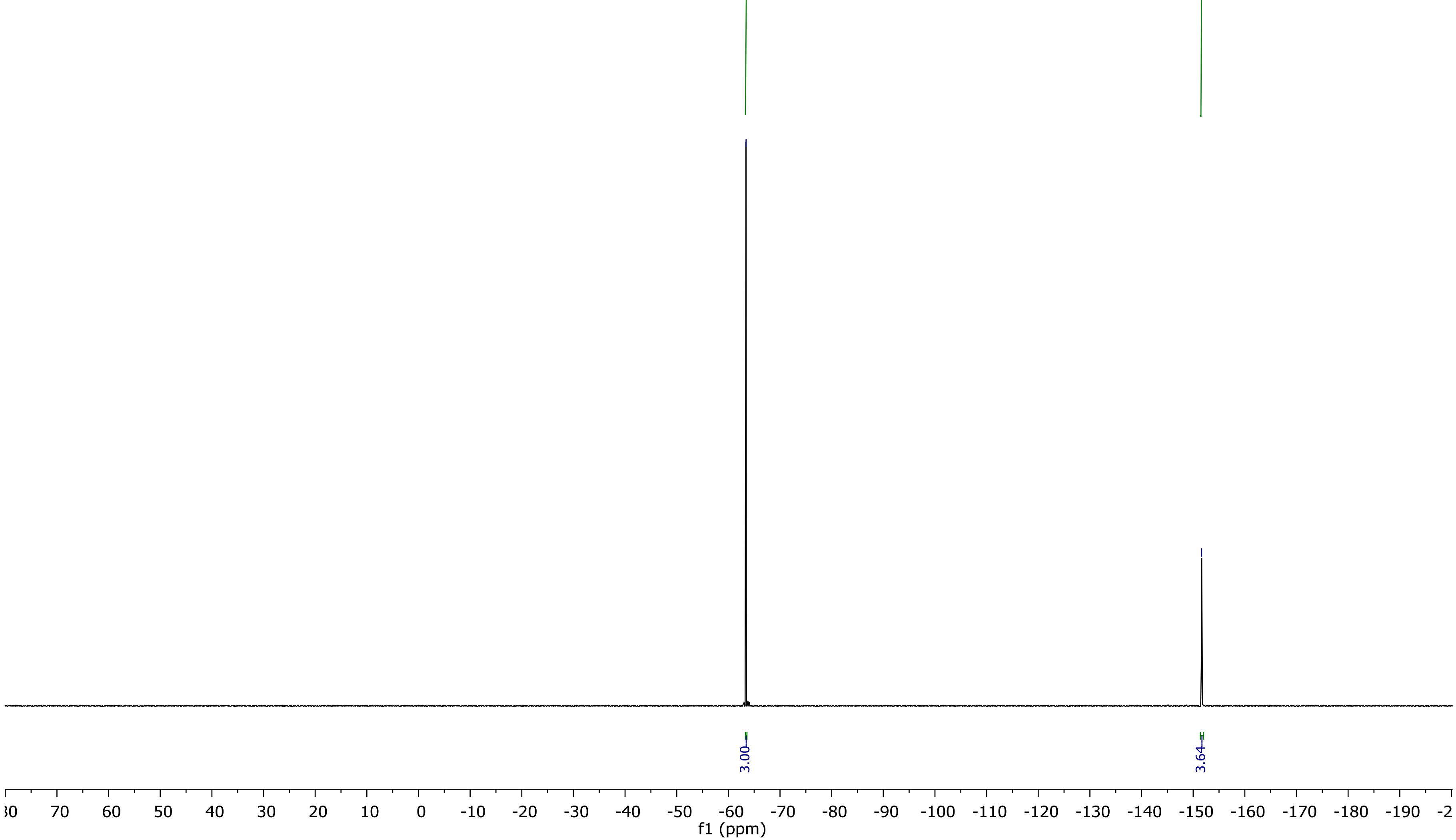

^{1}H NMR (400 MHz, CD_3CN)

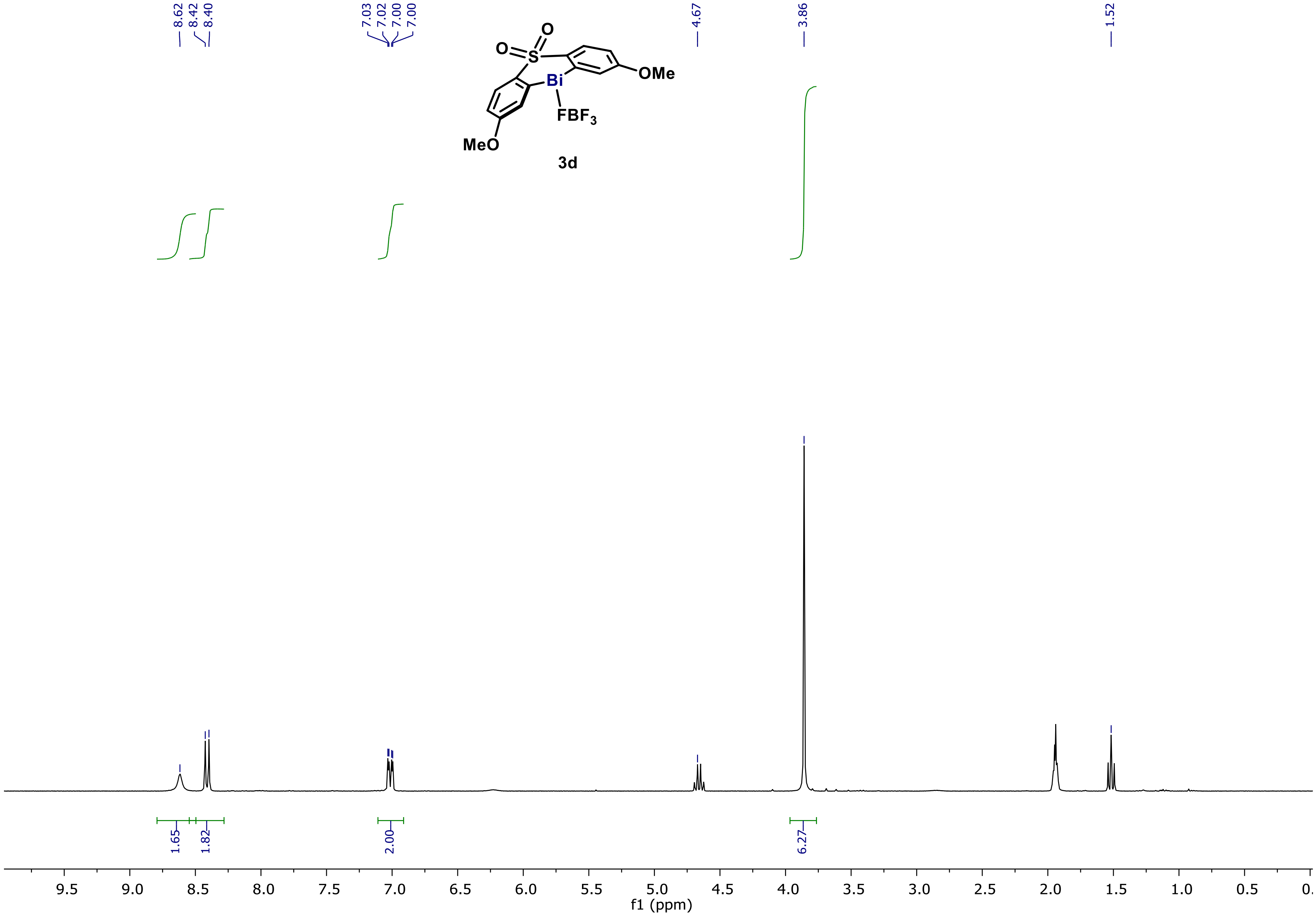

^{13}C NMR (101 MHz, CD_3CN)

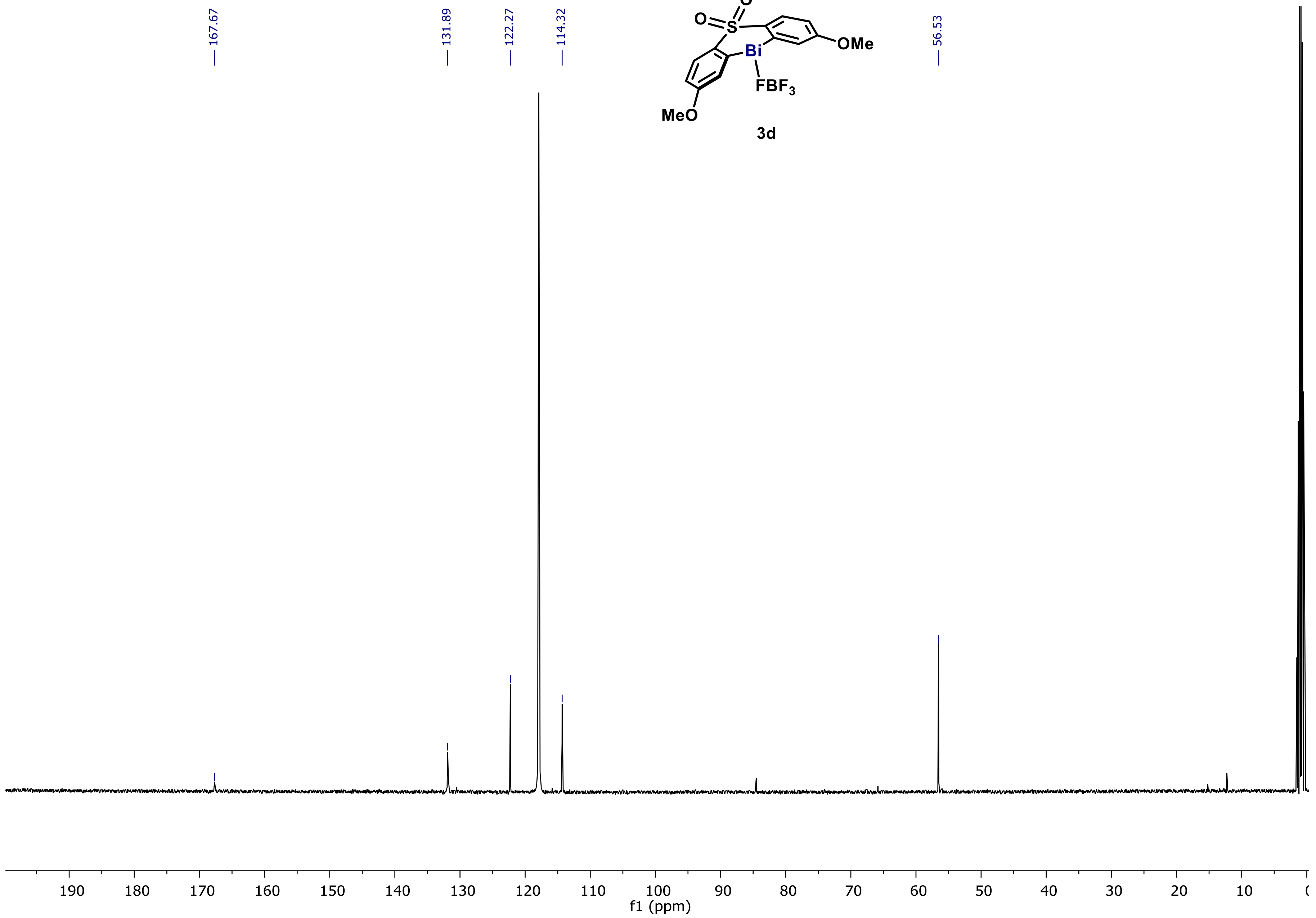

^{19}F NMR (282 MHz, CD_3CN)

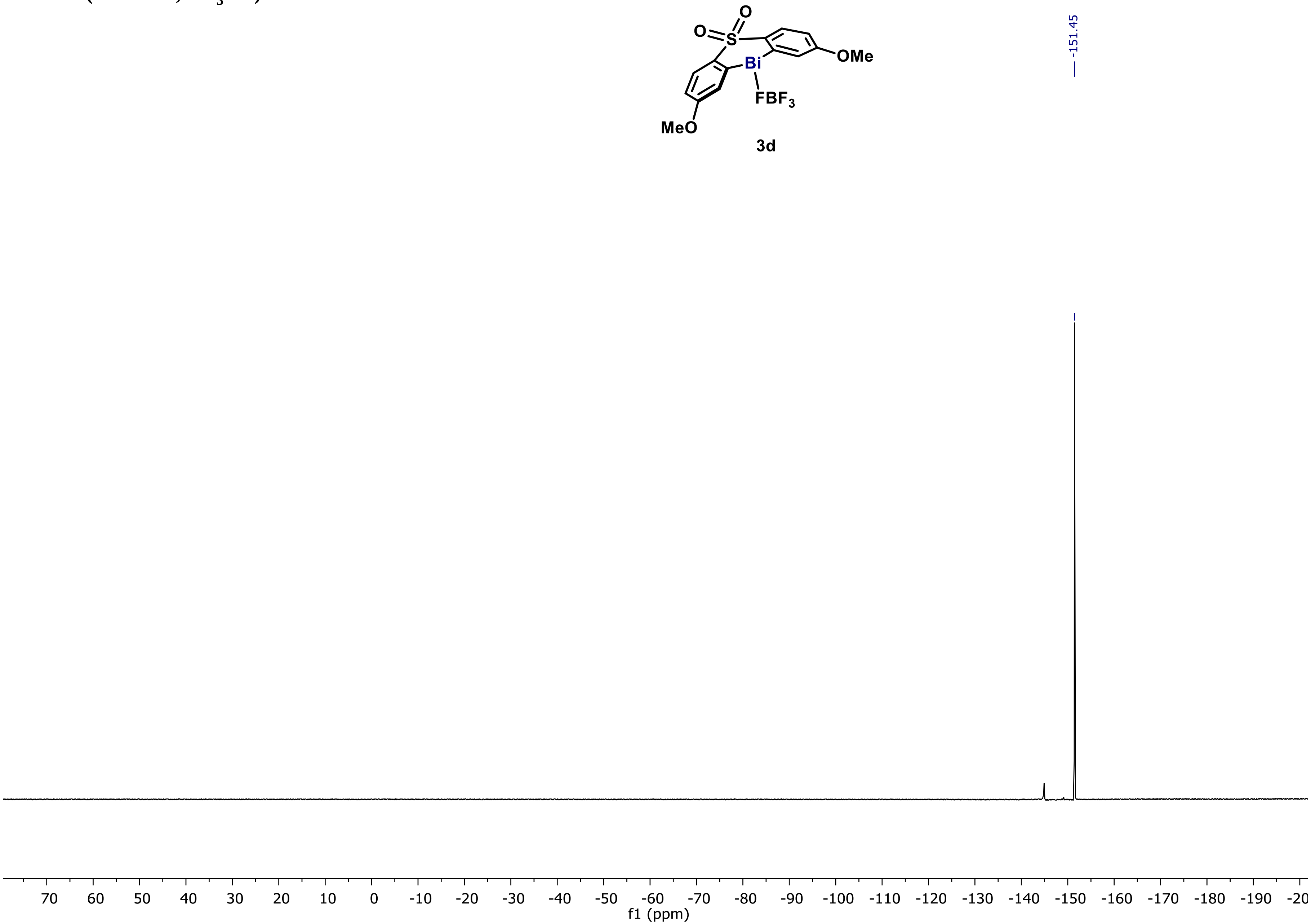

^{1}H NMR (400 MHz, CD_3CN)

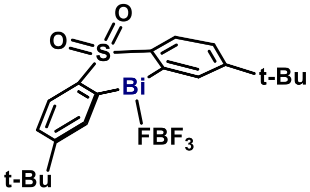

3e

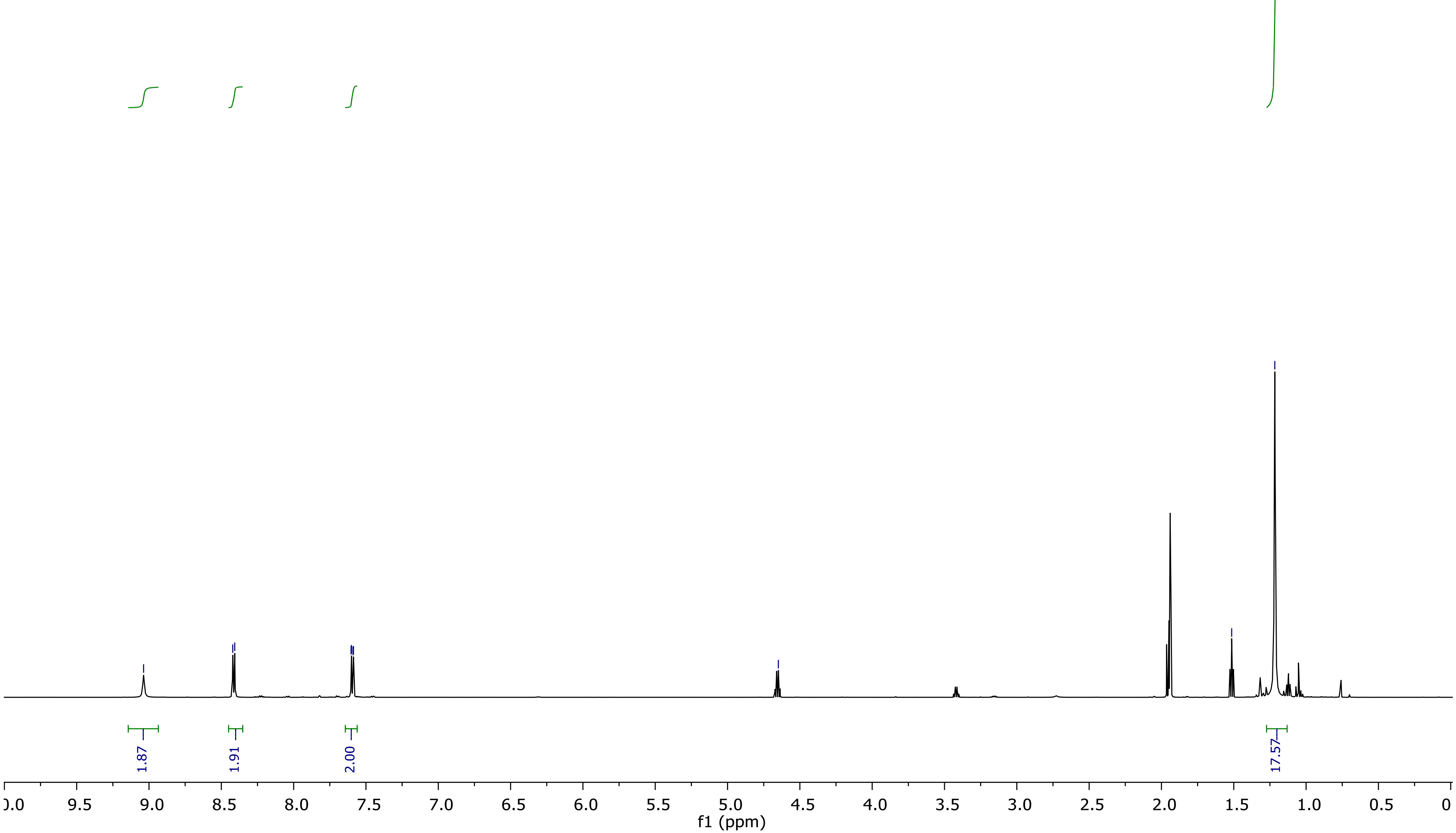

^{13}C NMR (101 MHz, CD_3CN)

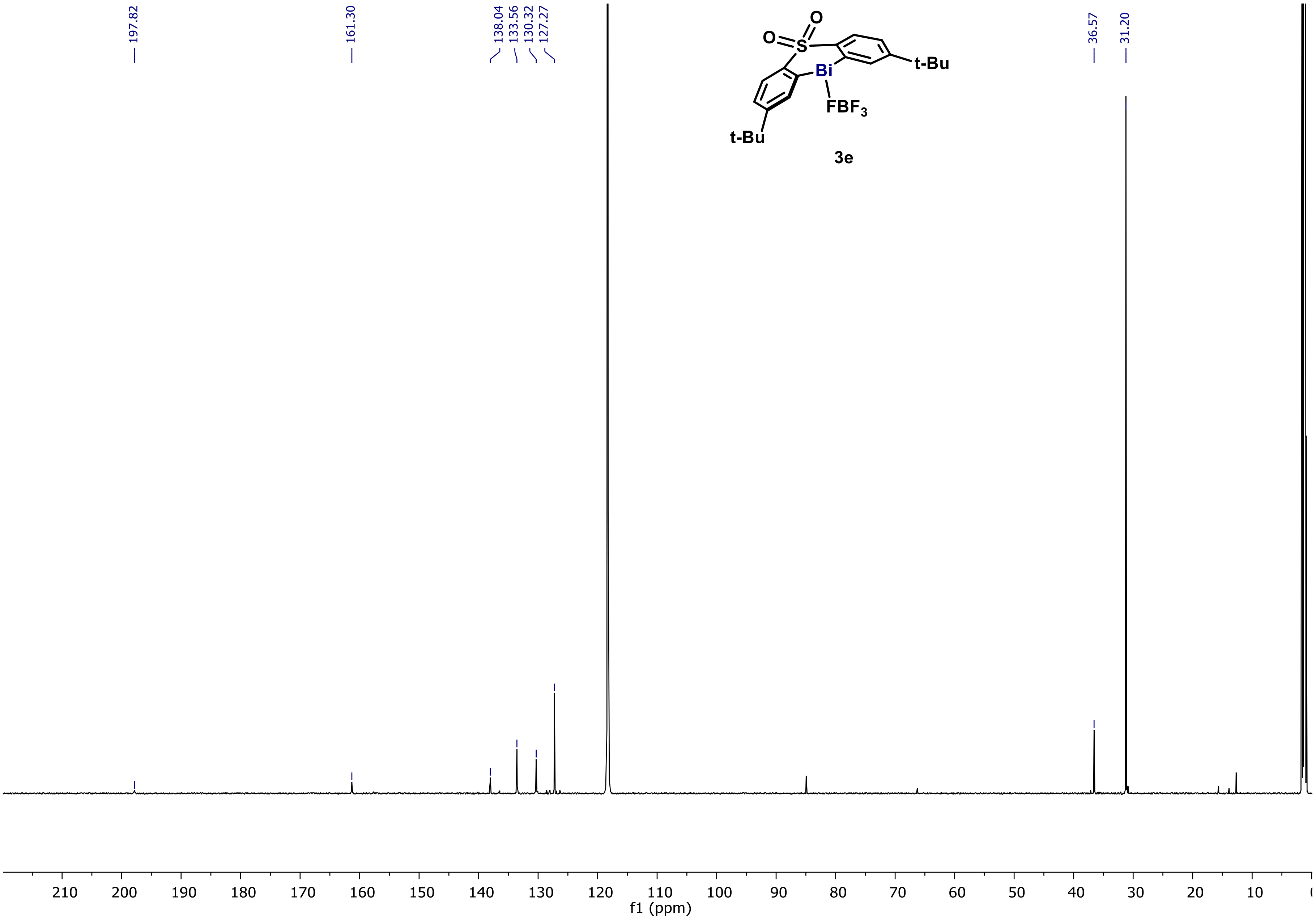

^{19}F NMR (282 MHz, CD_3CN)

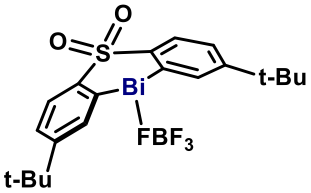

3e

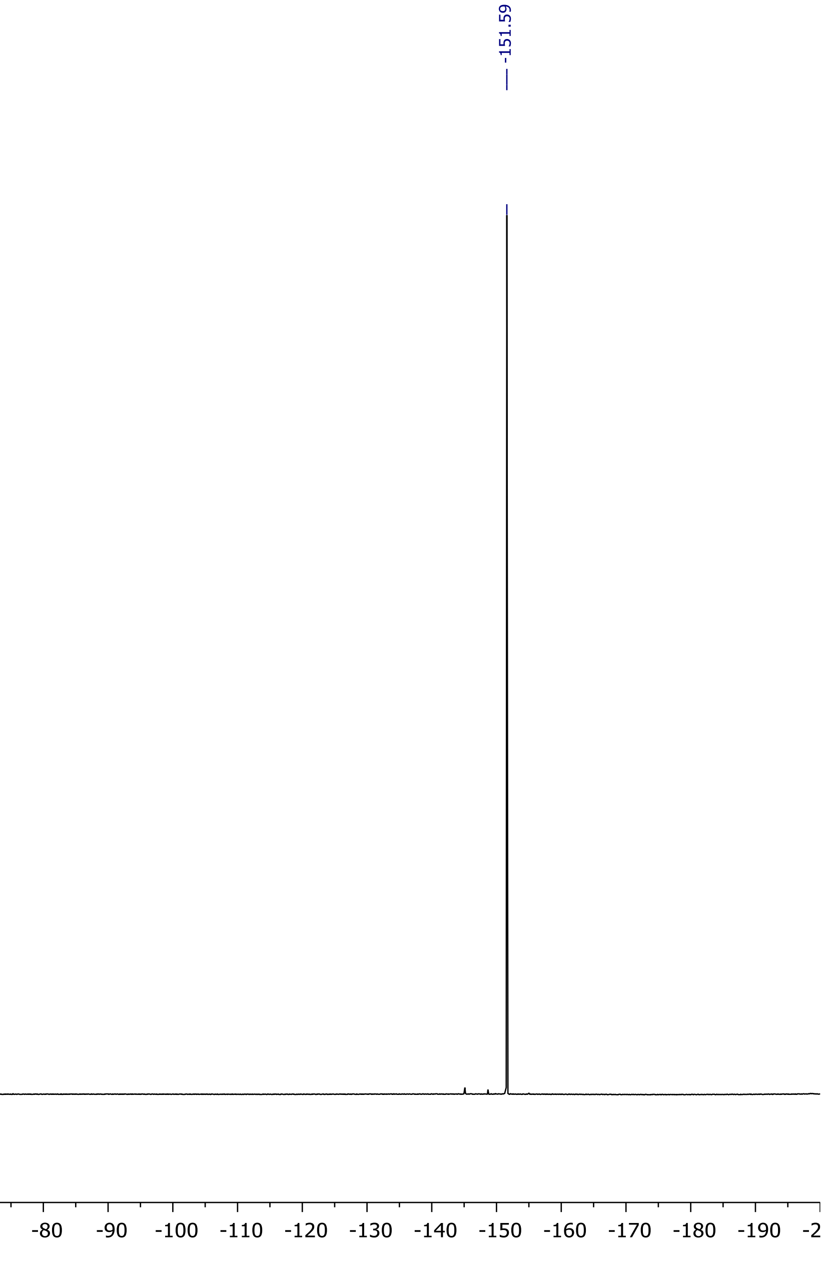

^{1}H NMR (600 MHz, $CDCl_3$, at -50 °C)

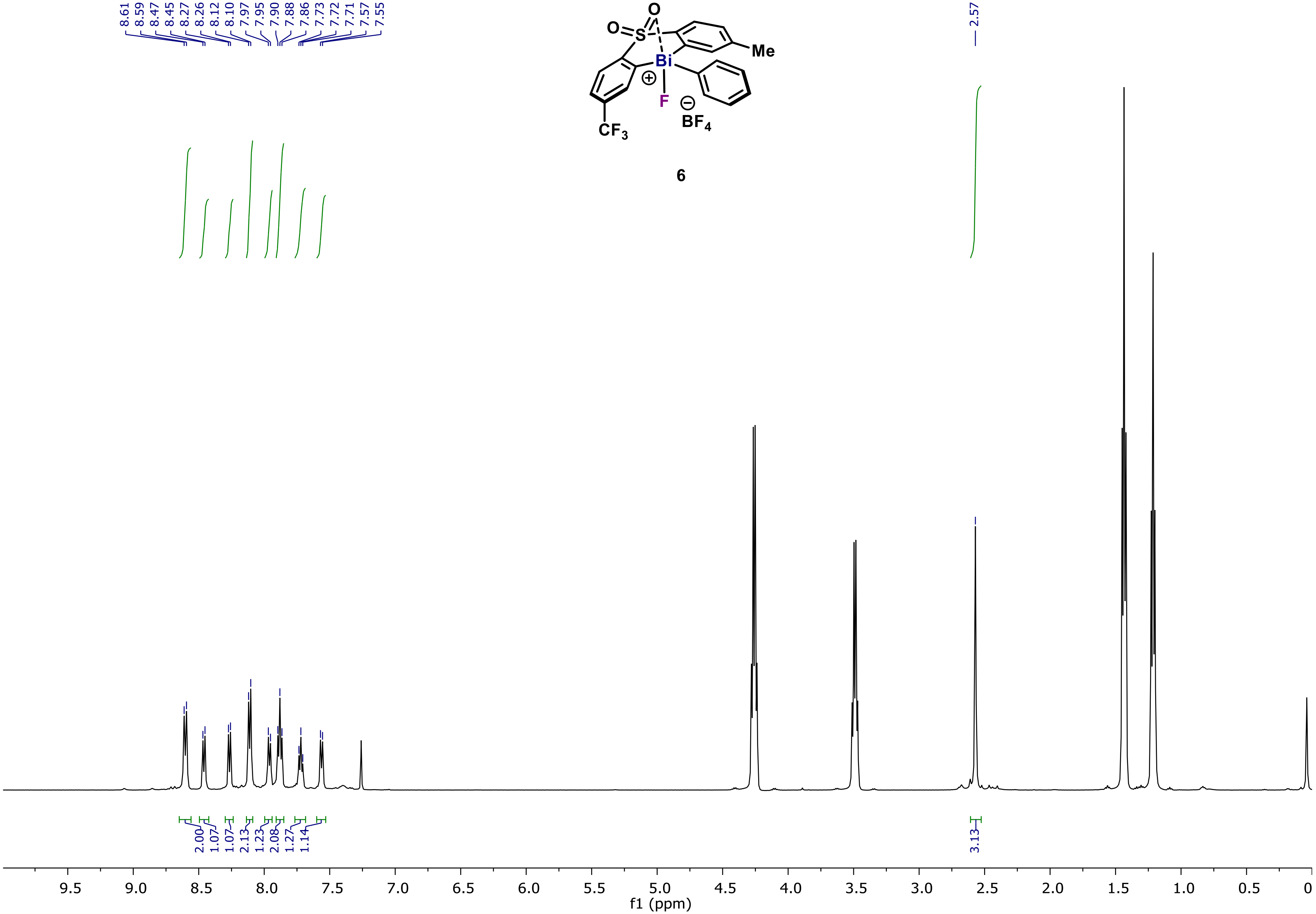

^{13}C NMR (151 MHz, $CDCl_3$, at -50 °C)

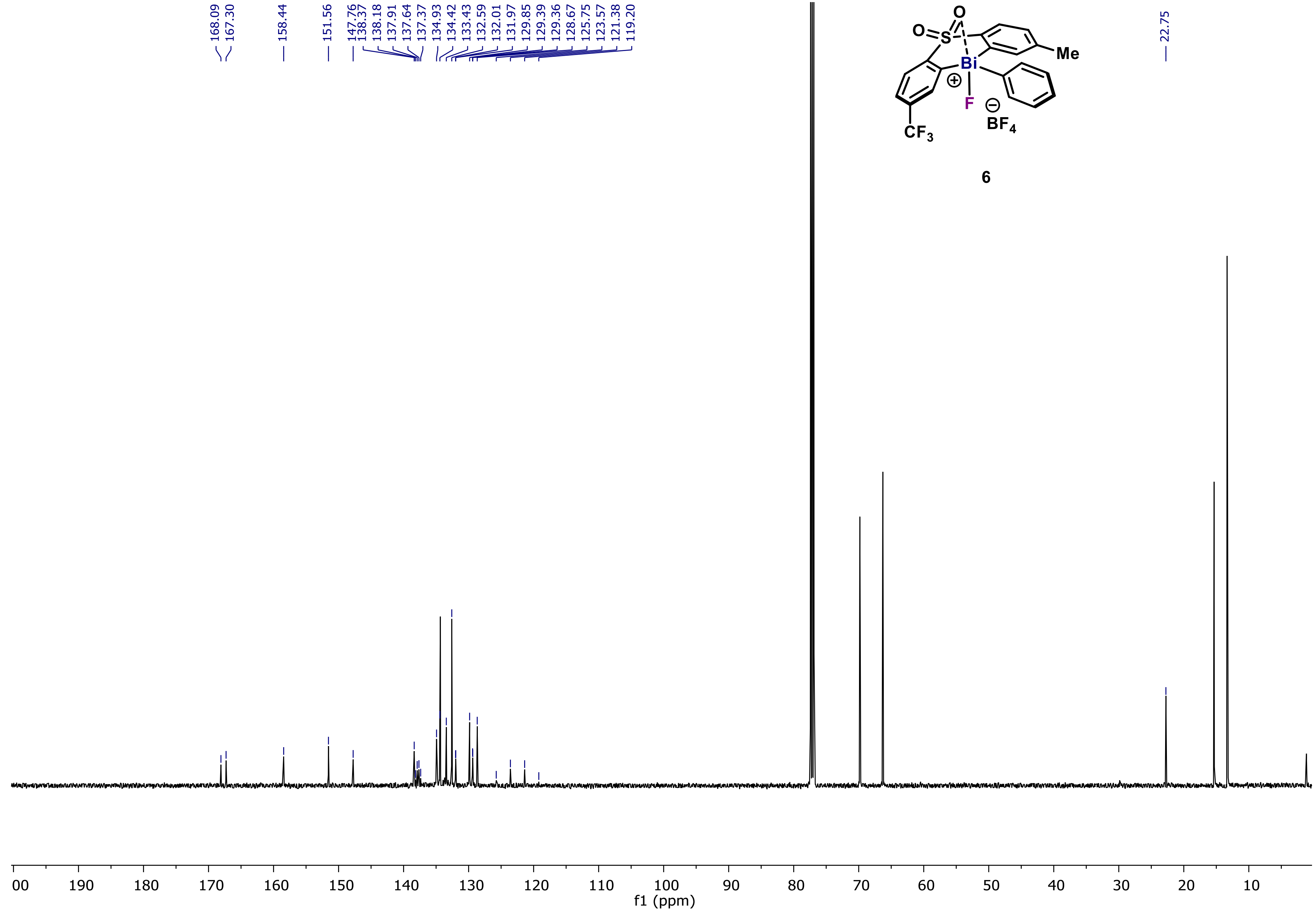

^{19}F NMR (470 MHz, $CDCl_3$, at -50 °C)

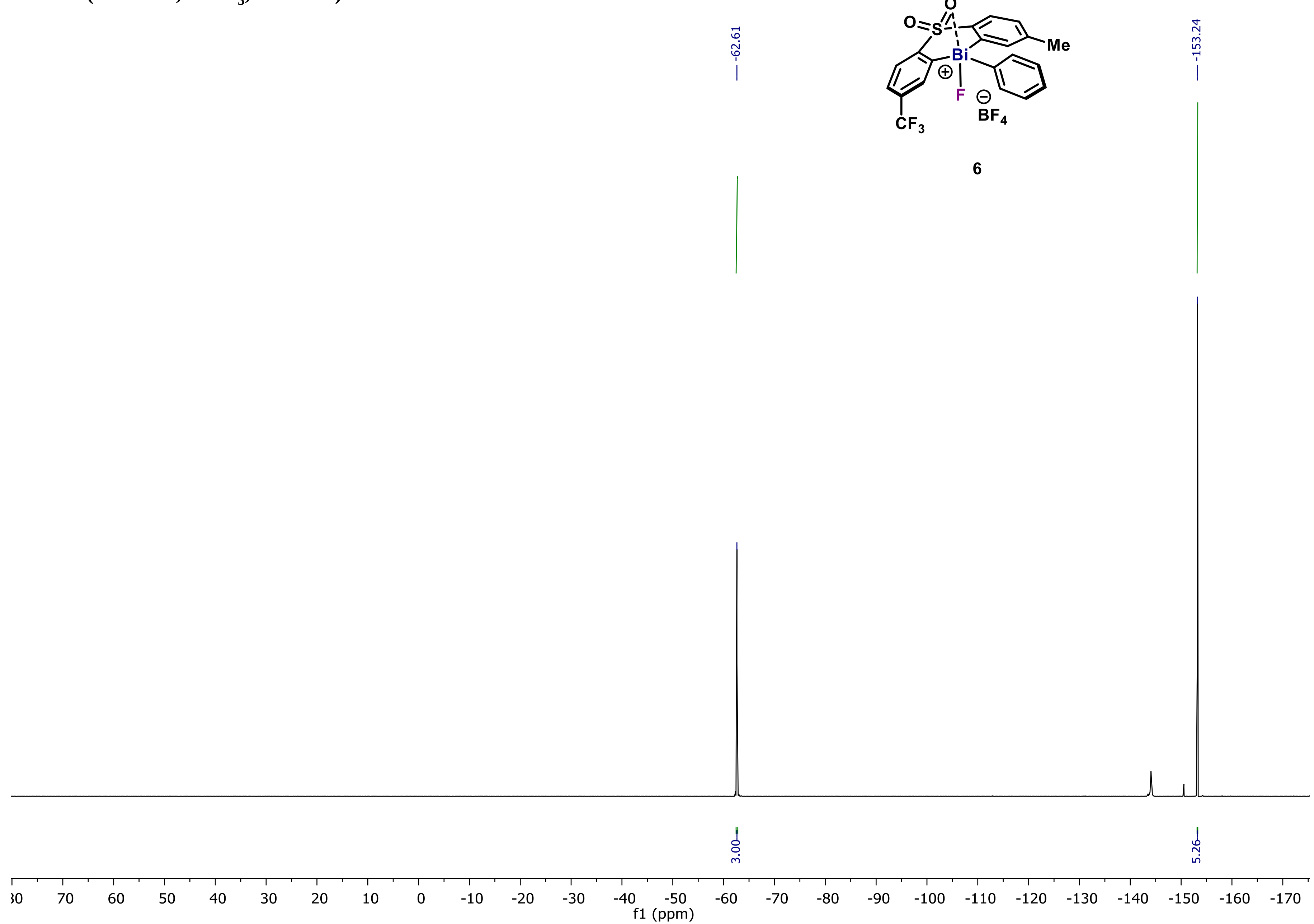

6

^{1}H NMR (400 MHz, $CDCl_3$)

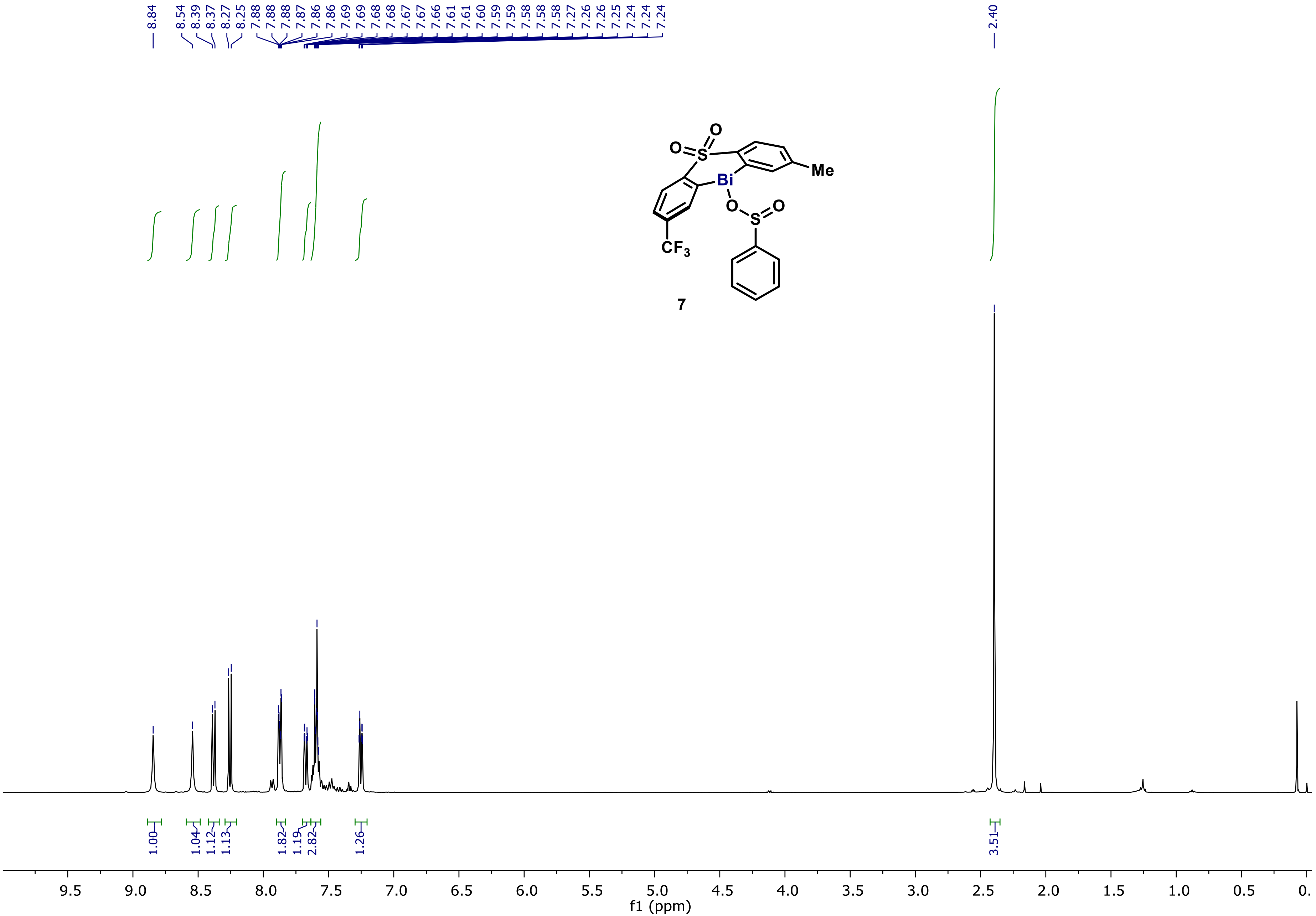

^{13}C NMR (101 MHz, $CDCl_3$)

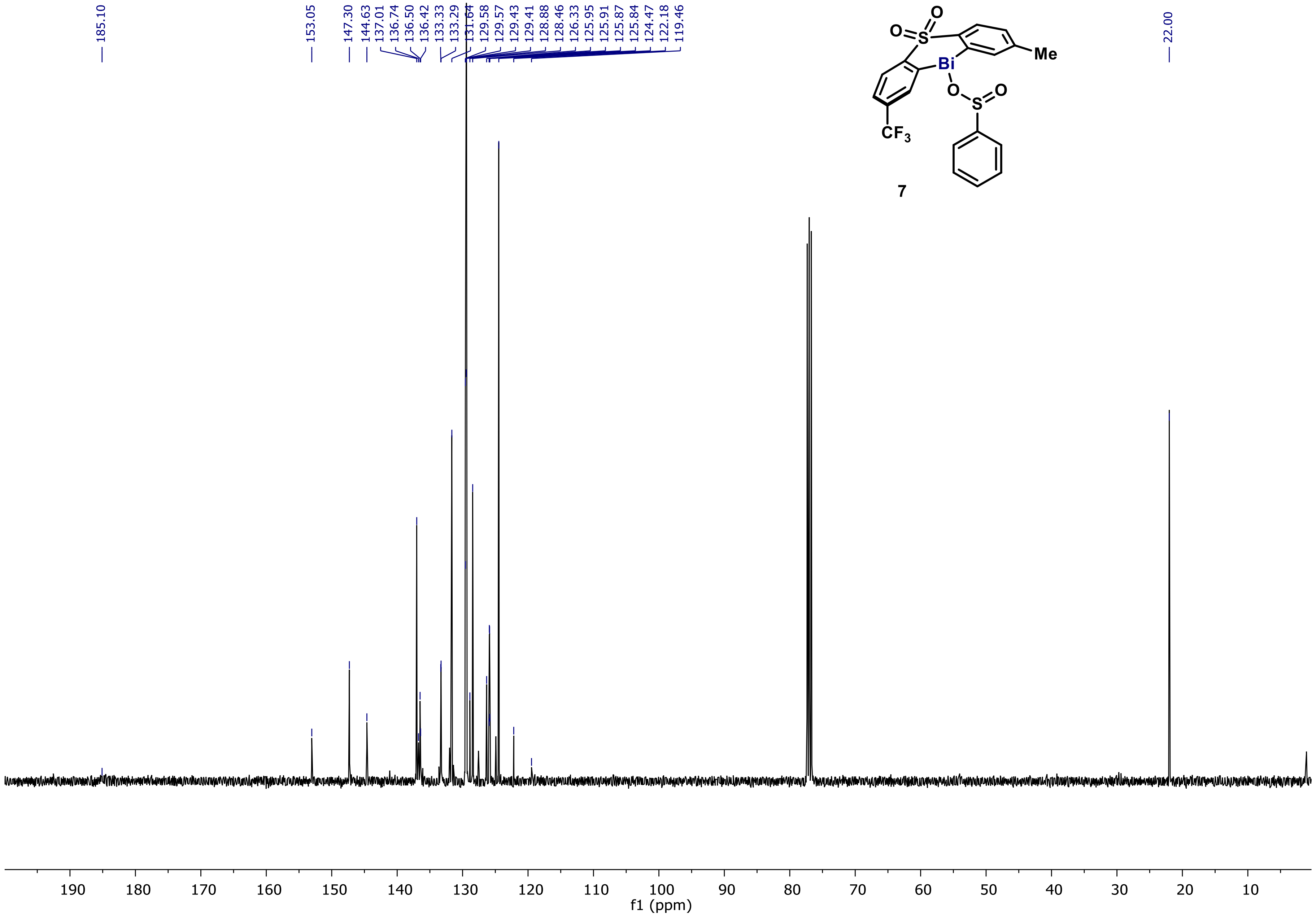

^{19}F NMR (282 MHz, CDCl$_3$)

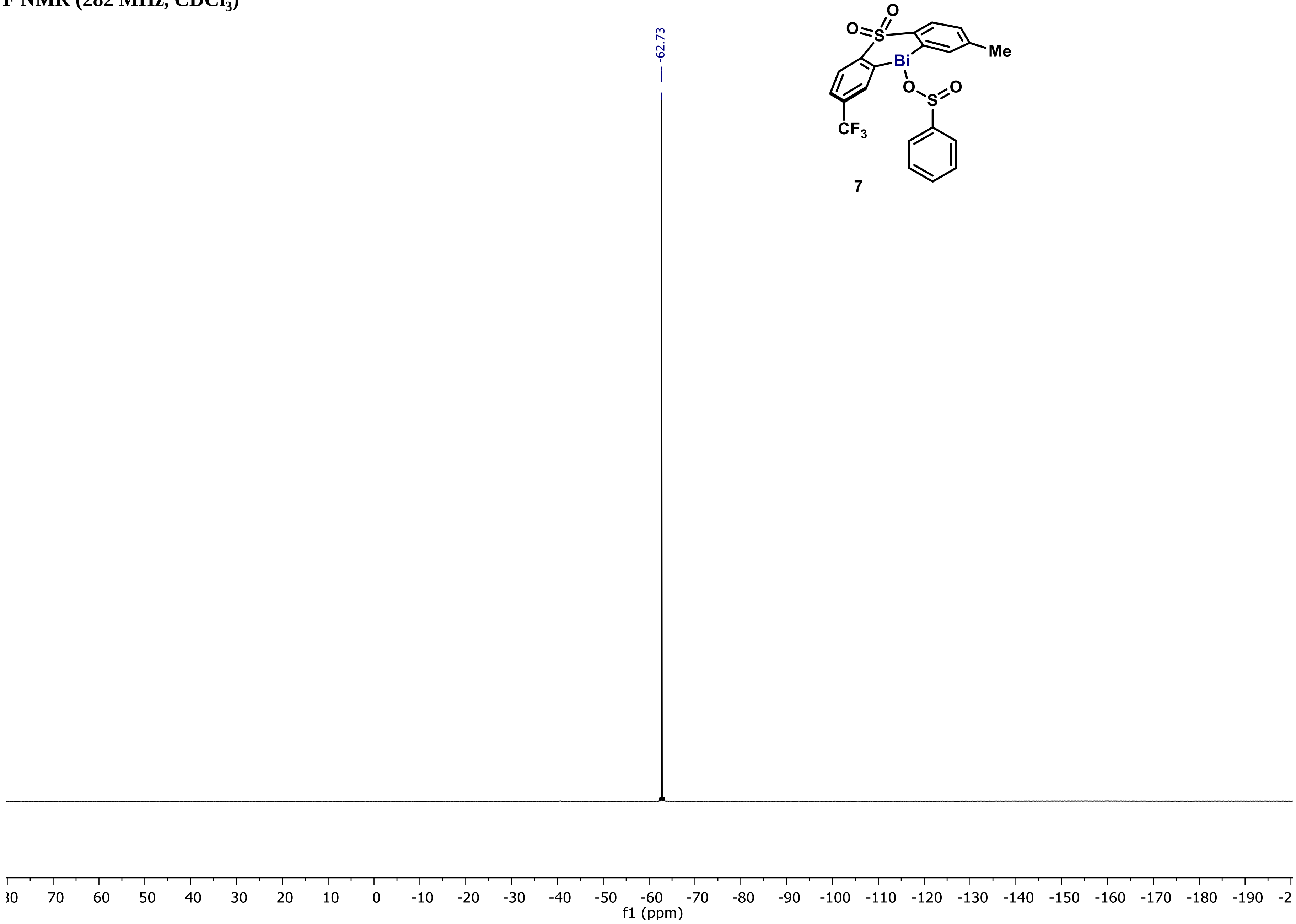

7

^{1}H NMR (400 MHz, CD_3CN)

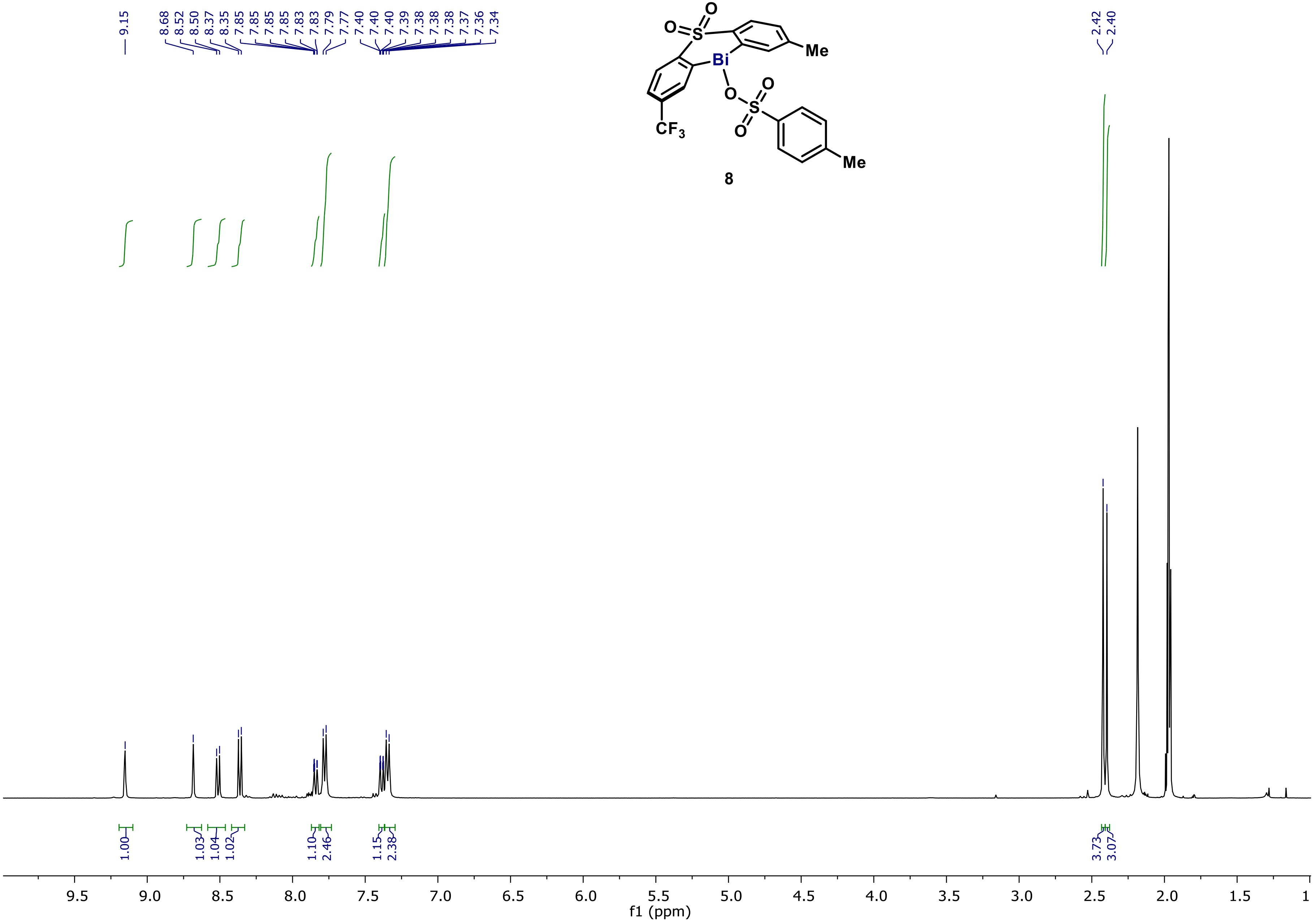

^{13}C NMR (101 MHz, CD_3CN)

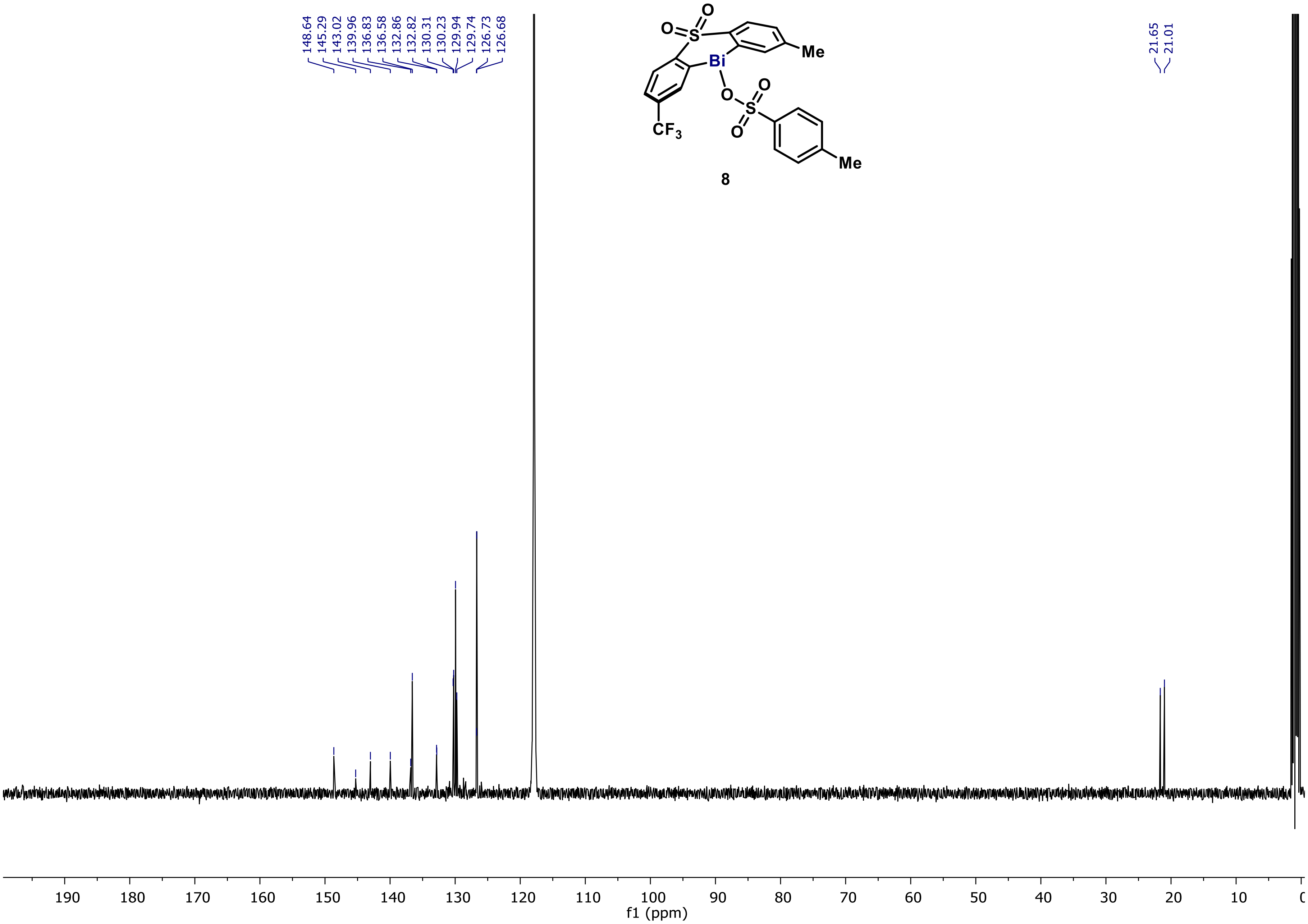

^{19}F NMR (282 MHz, CD_3CN)

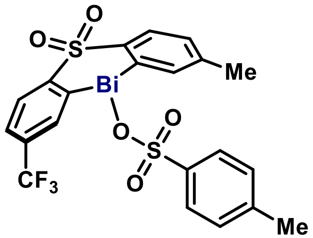

8

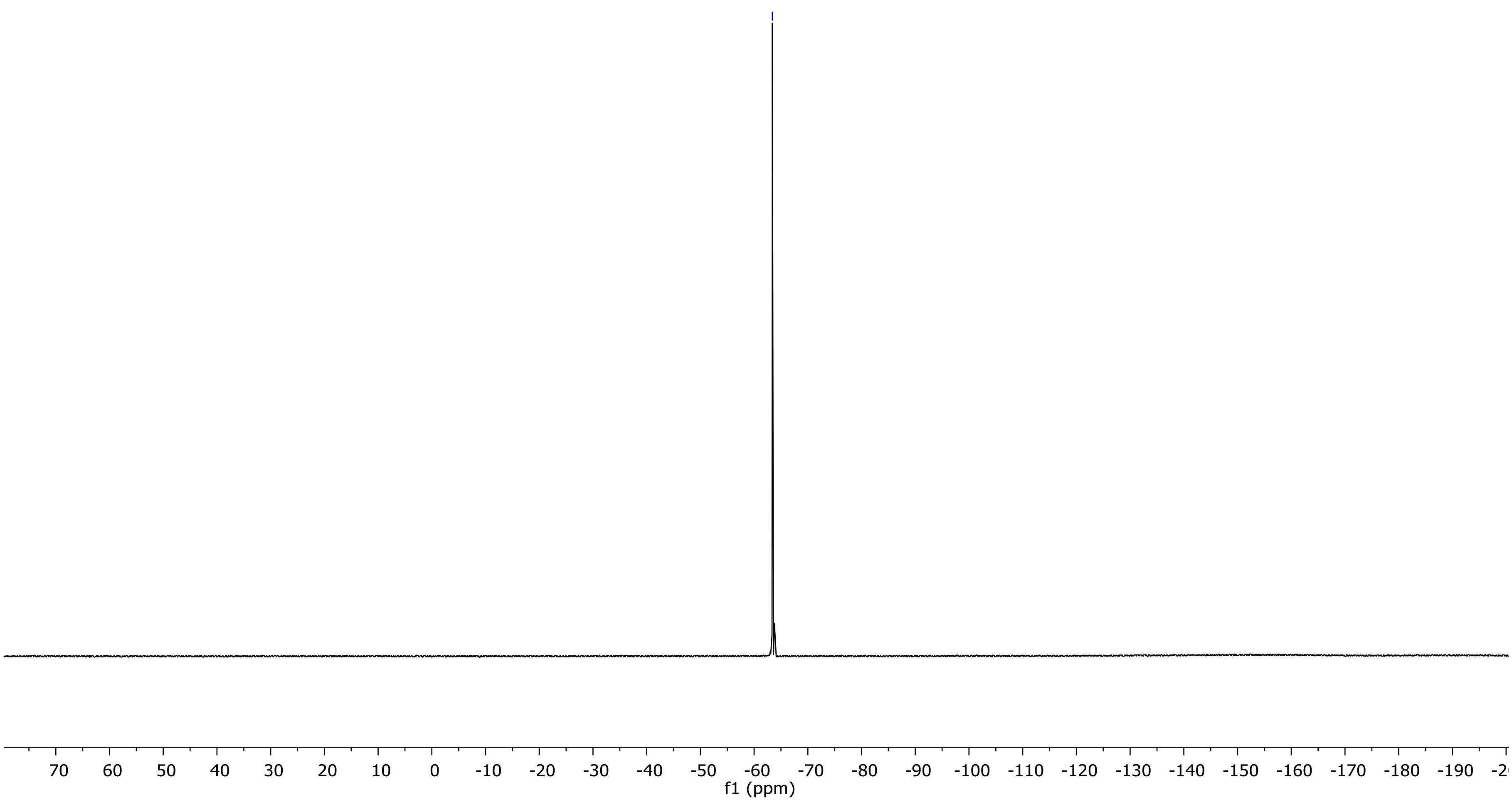

^{1}H NMR (600 MHz, $CDCl_3$)

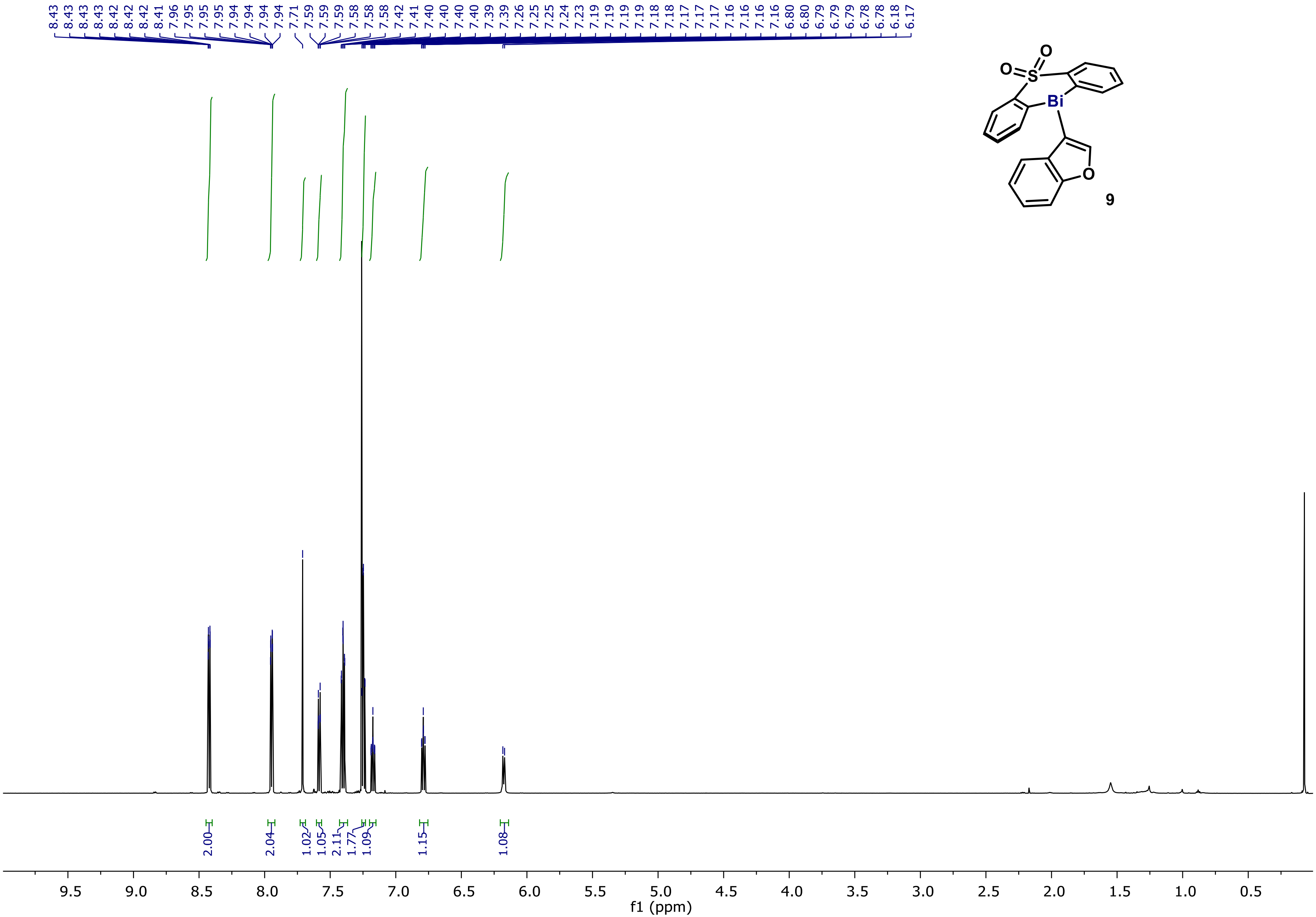

9

^{13}C NMR (151 MHz, CDCl$_3$)

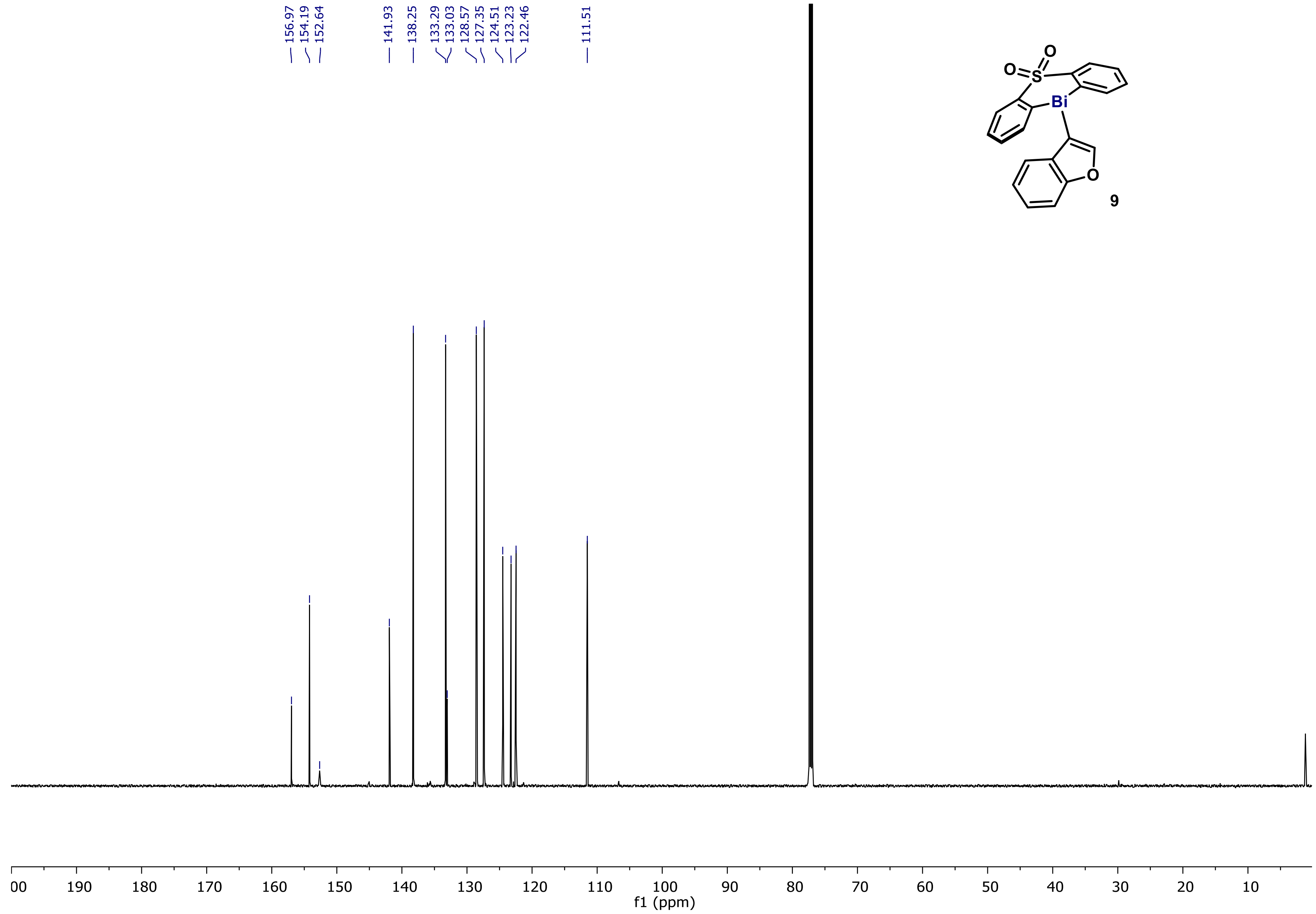

^{1}H NMR (400 MHz, $CDCl_3$)

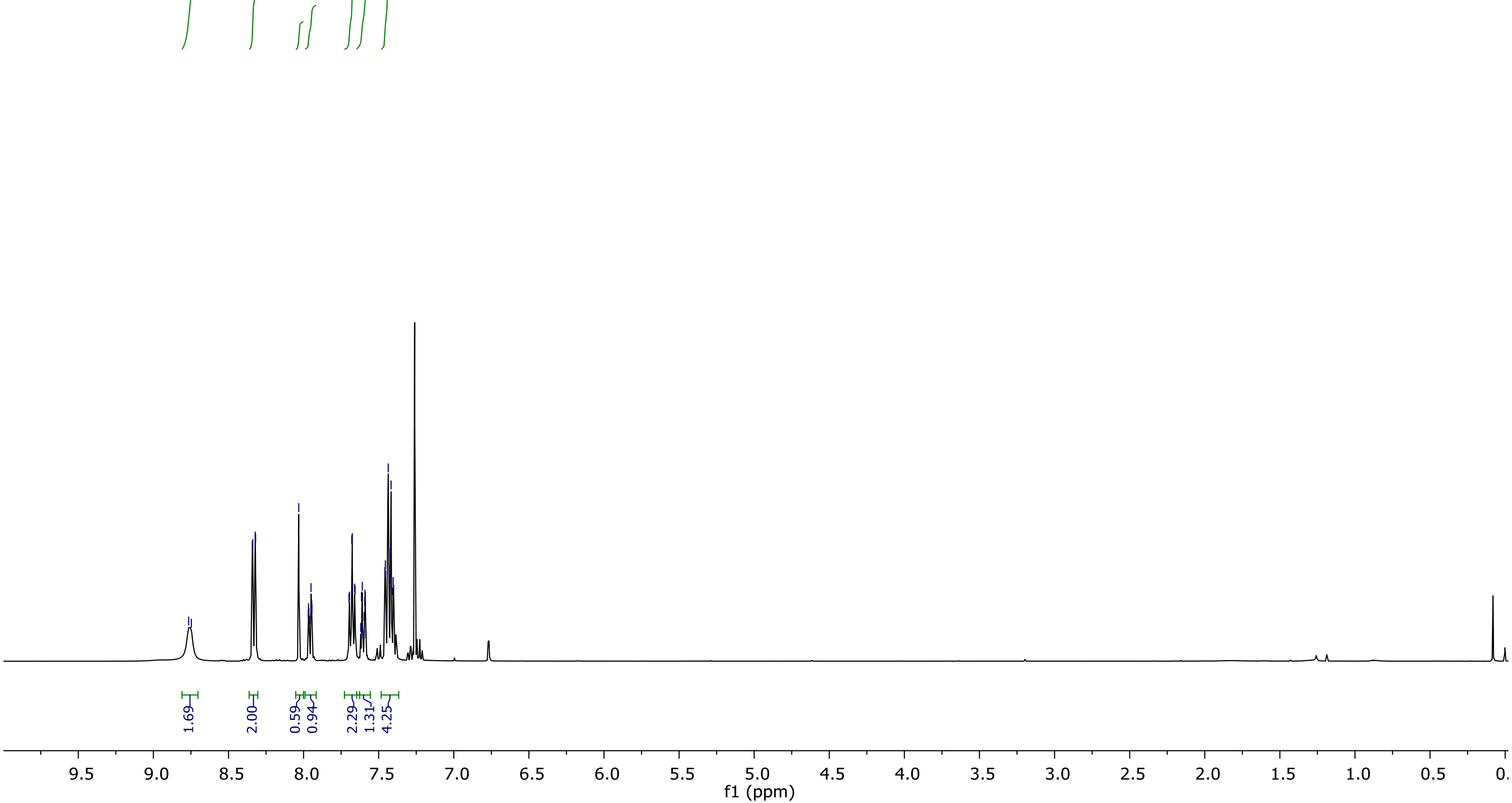

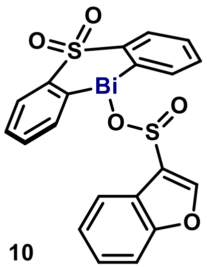

10

^{13}C NMR (101 MHz, $CDCl_3$)

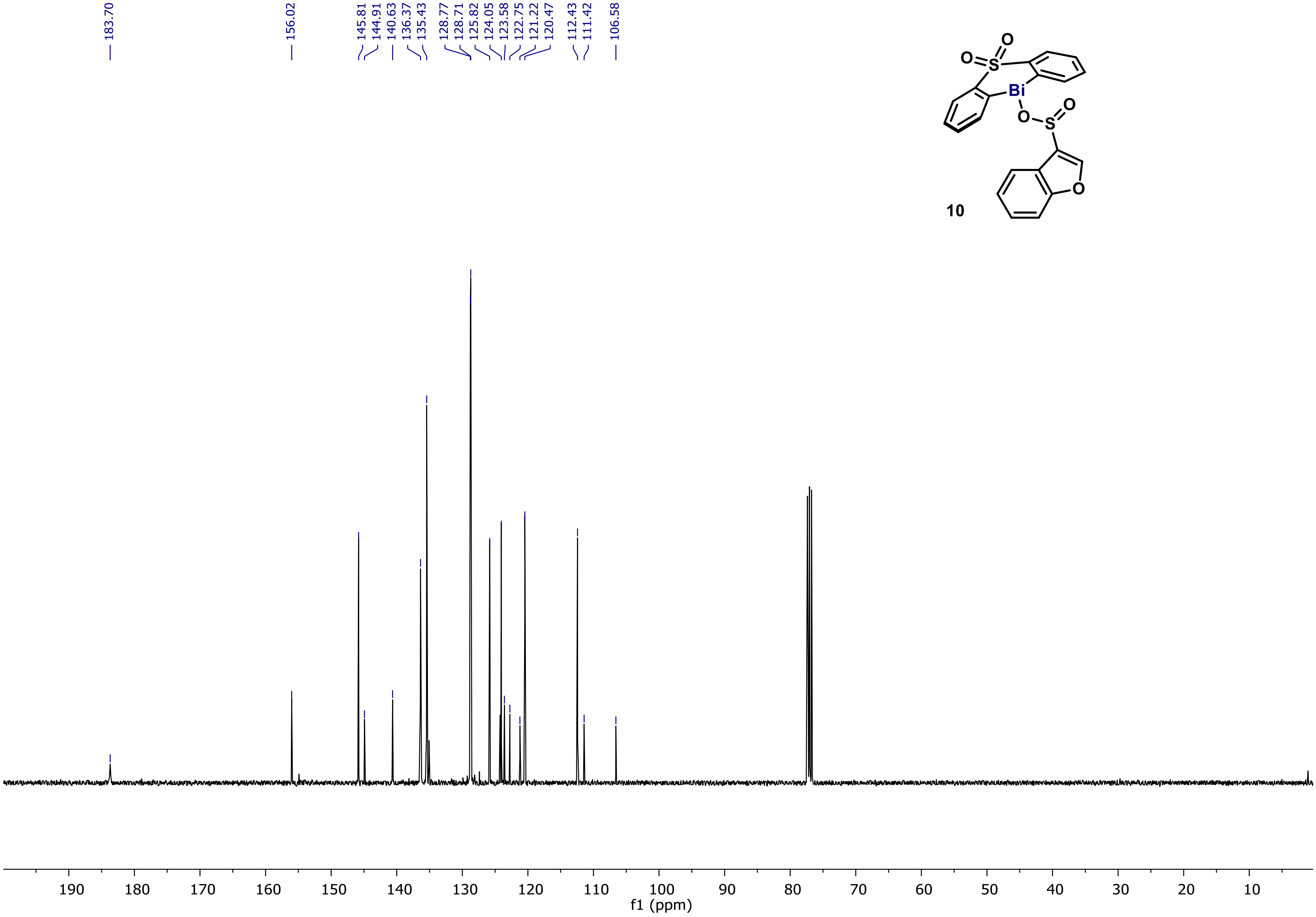

^{1}H NMR (300 MHz, $CDCl_3$)

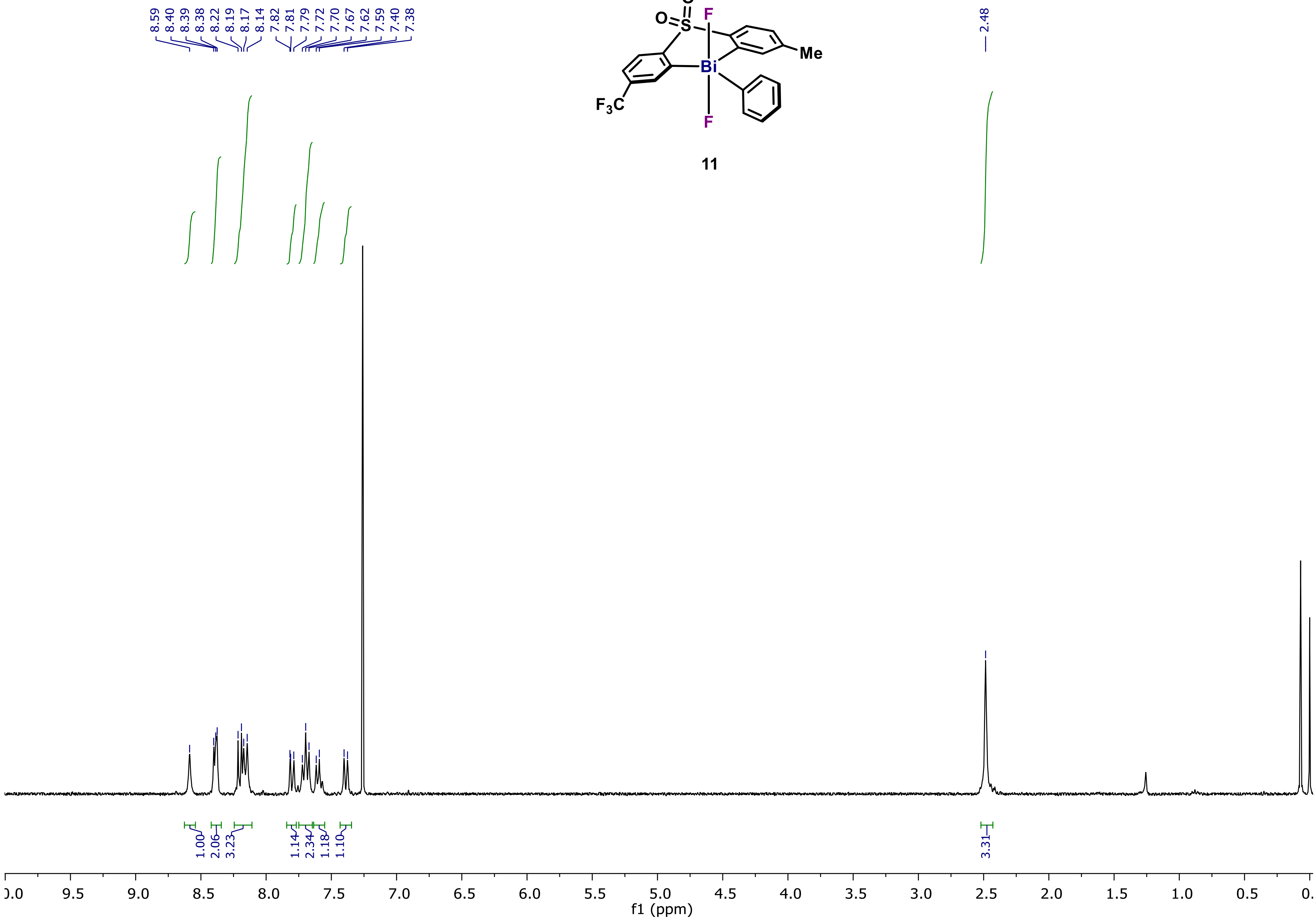

^{19}F NMR (282 MHz, $CDCl_3$)

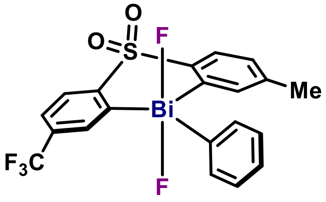

11

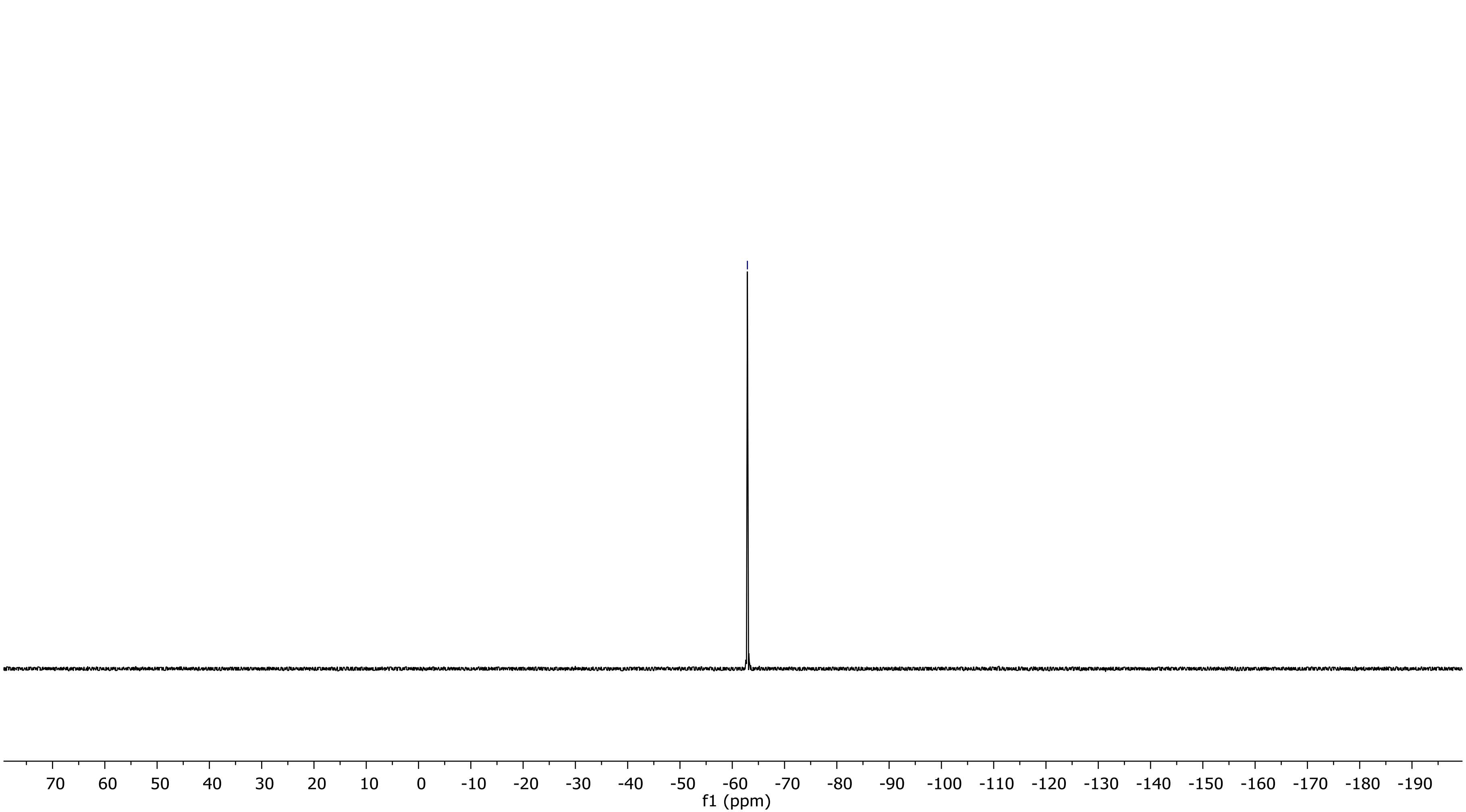

^{1}H NMR (600 MHz, $CDCl_3$)

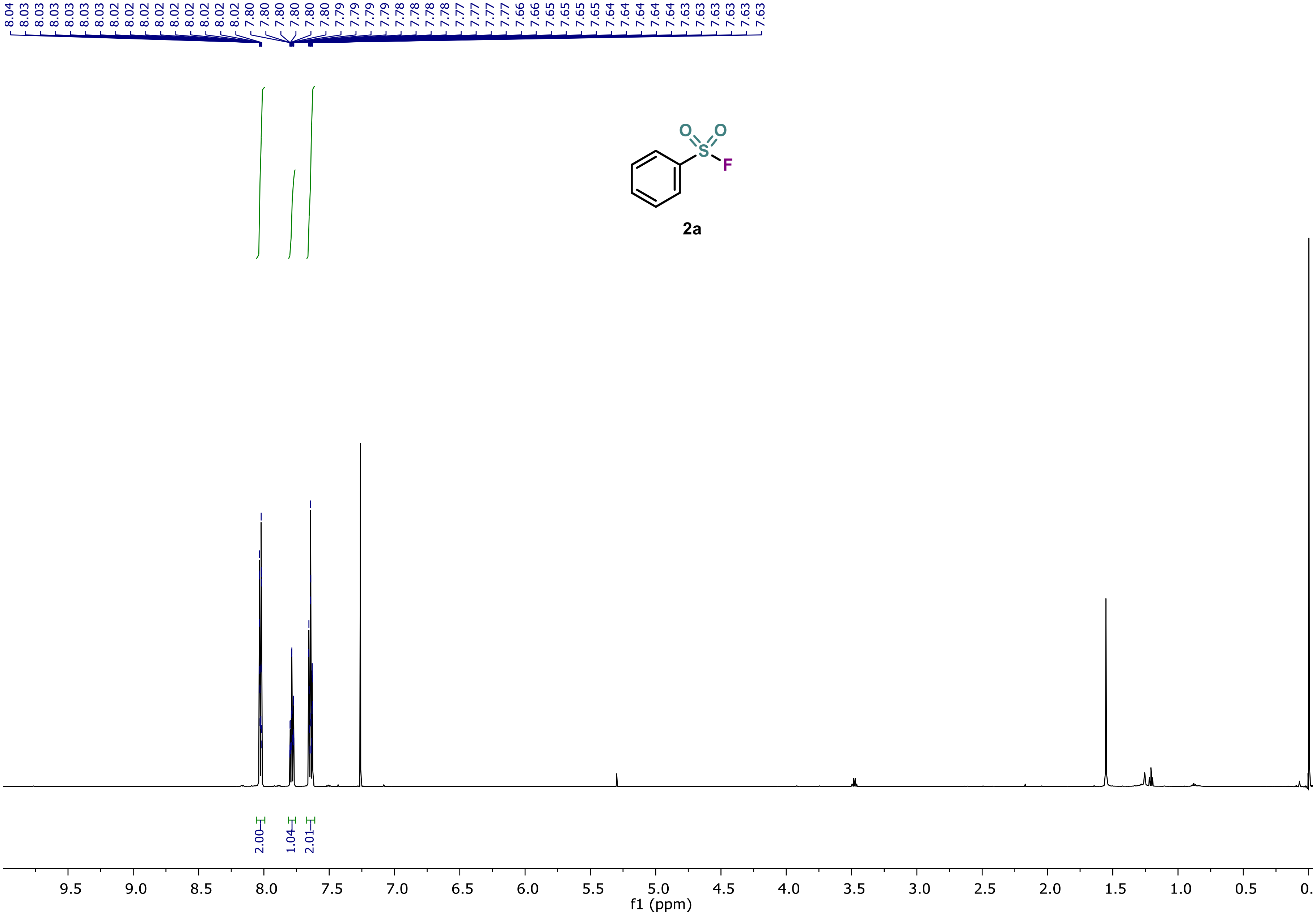

2a

^{13}C NMR (151 MHz, $CDCl_3$)

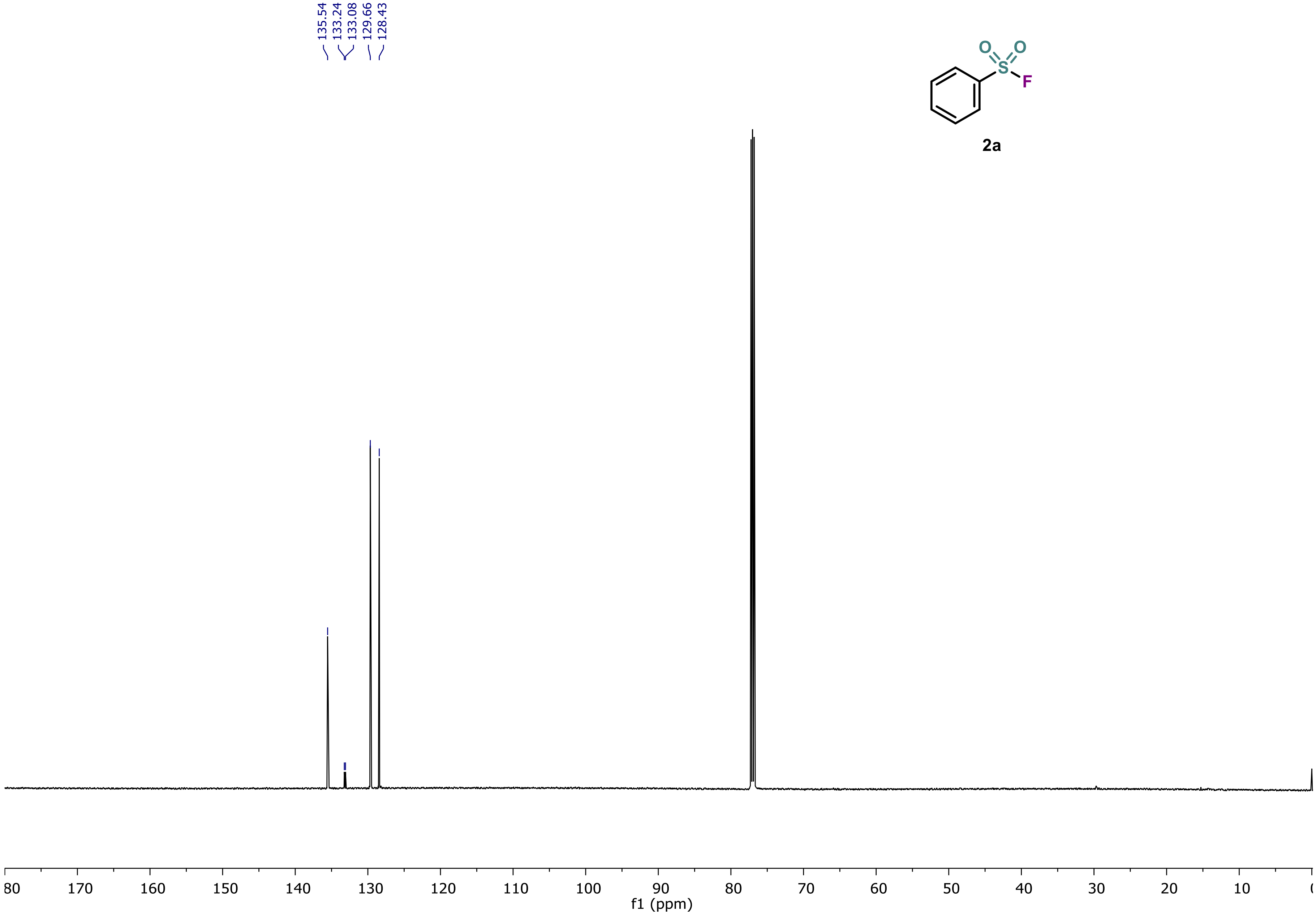

2a

^{19}F NMR (565 MHz, $CDCl_3$)

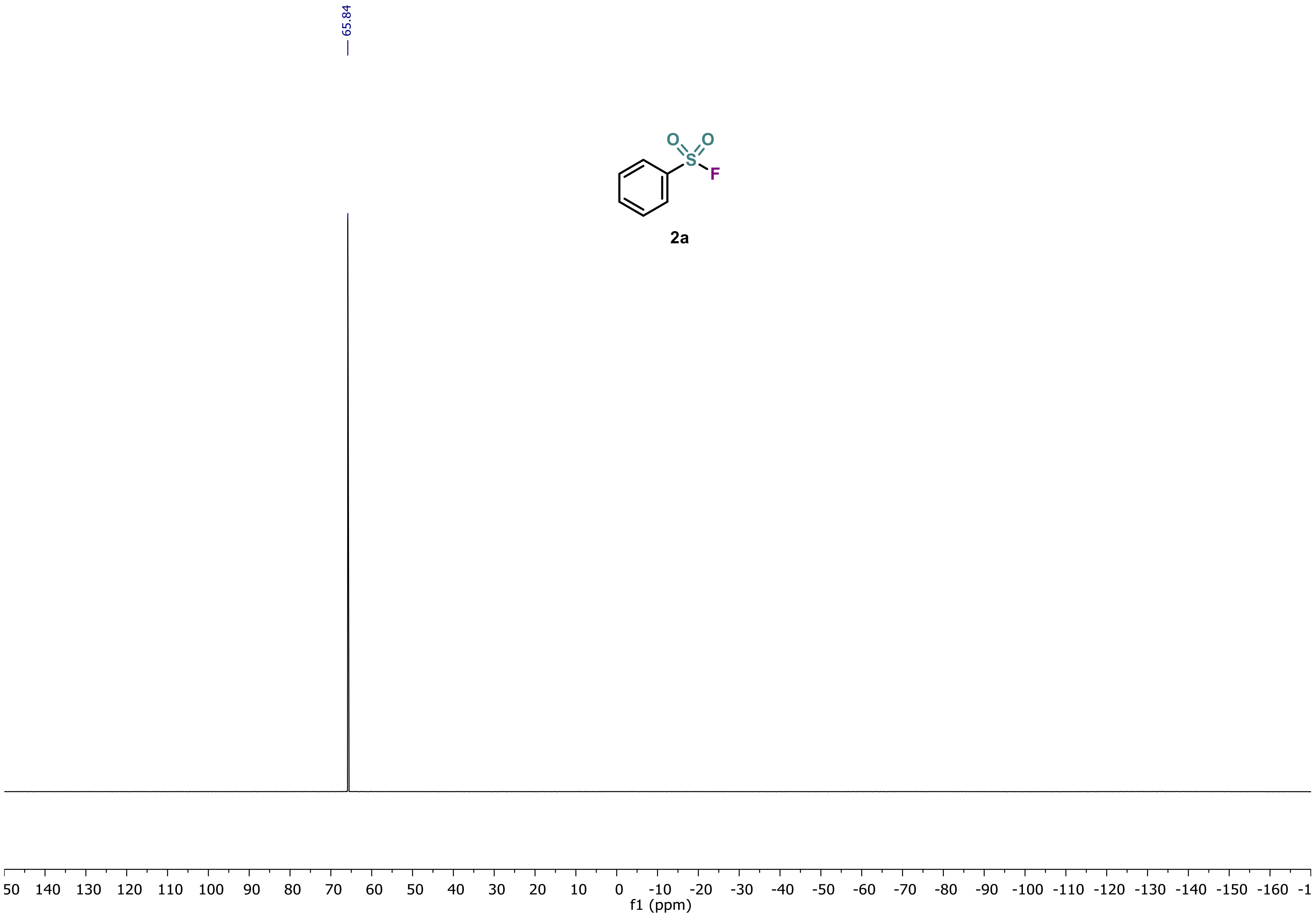

^{1}H NMR (600 MHz, $CDCl_3$)

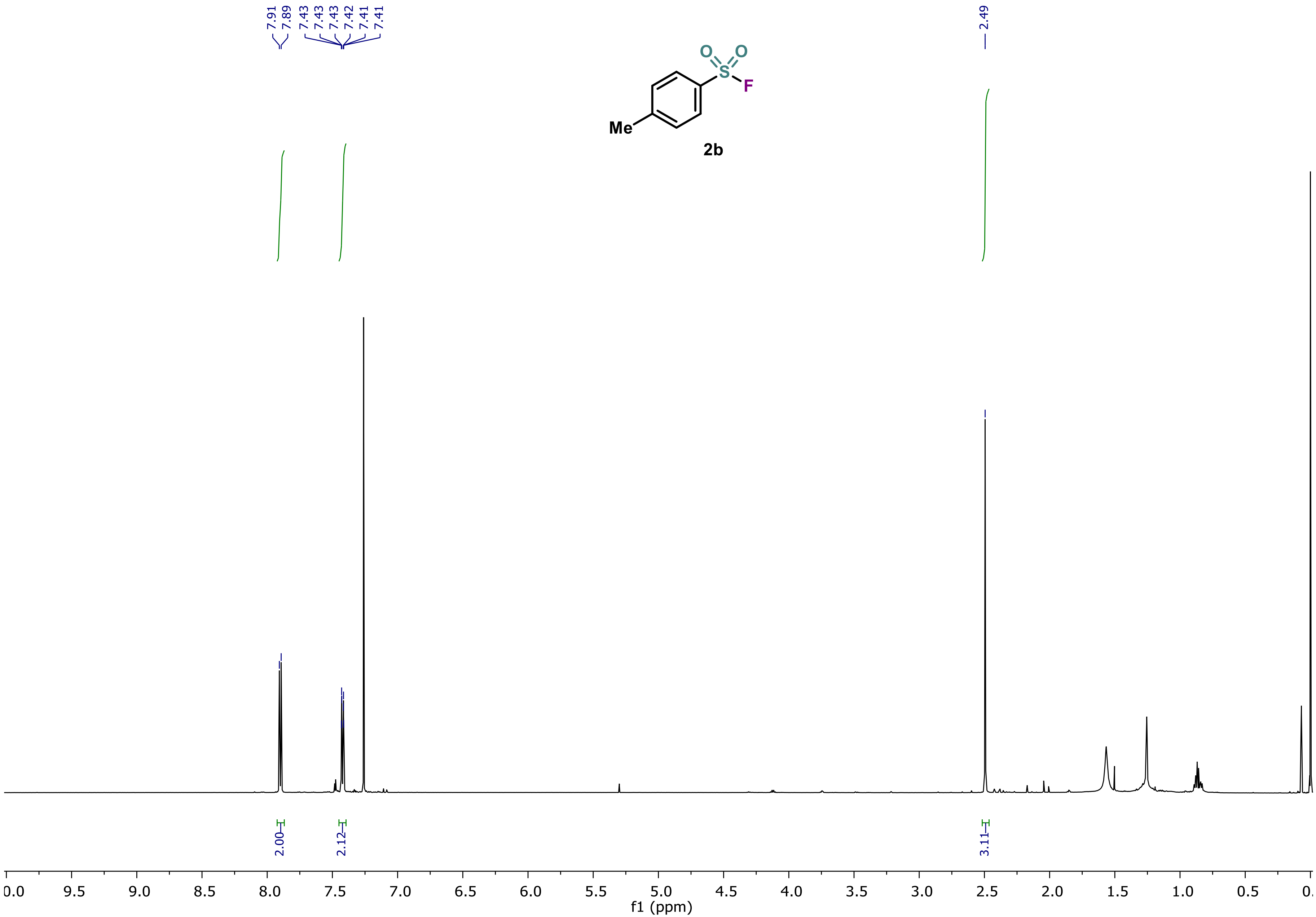

^{13}C NMR (151 MHz, $CDCl_3$)

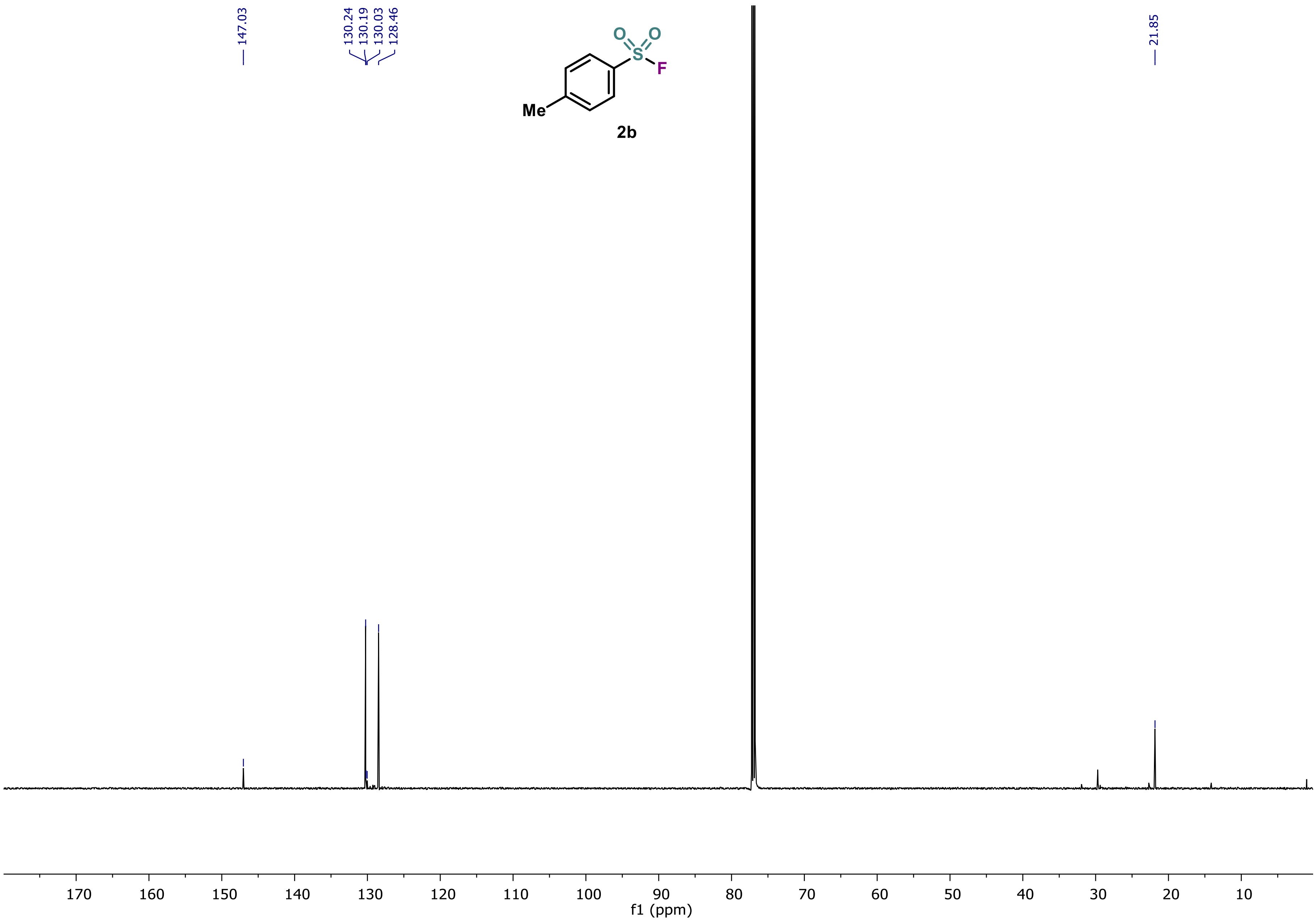

^{19}F NMR (565 MHz, $CDCl_3$)

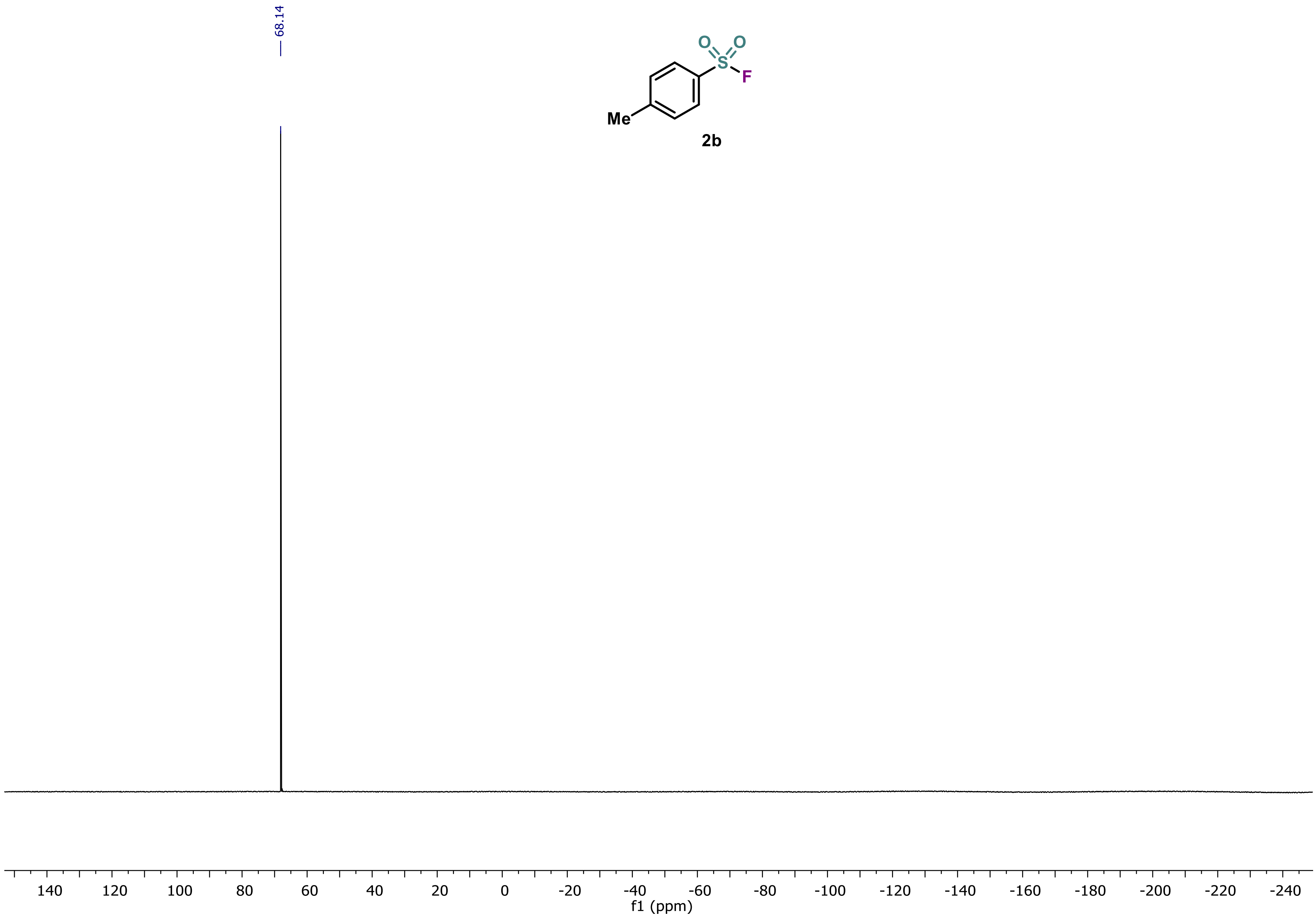

^{1}H NMR (600 MHz, $CDCl_3$)

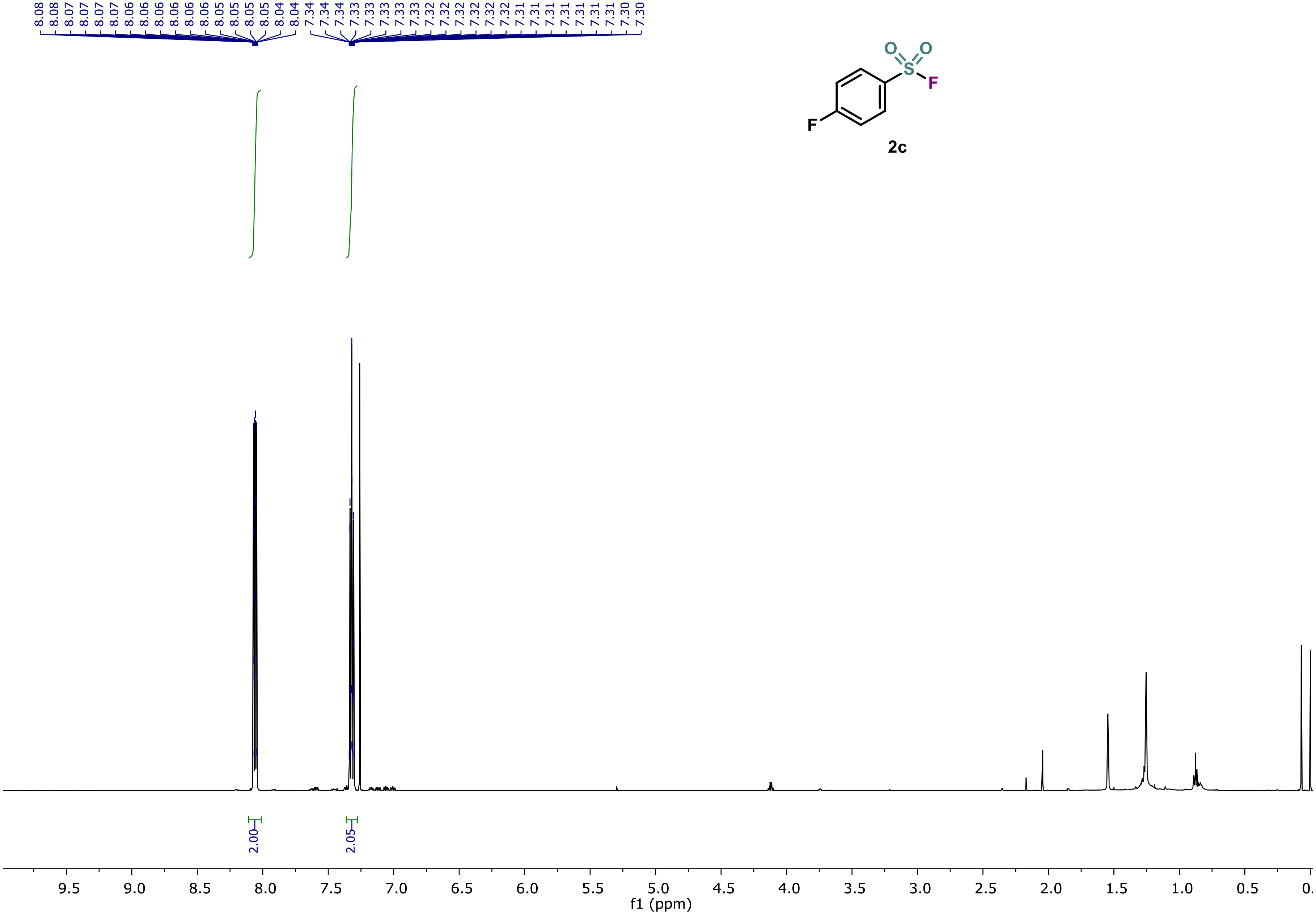

^{13}C NMR (151 MHz, $CDCl_3$)

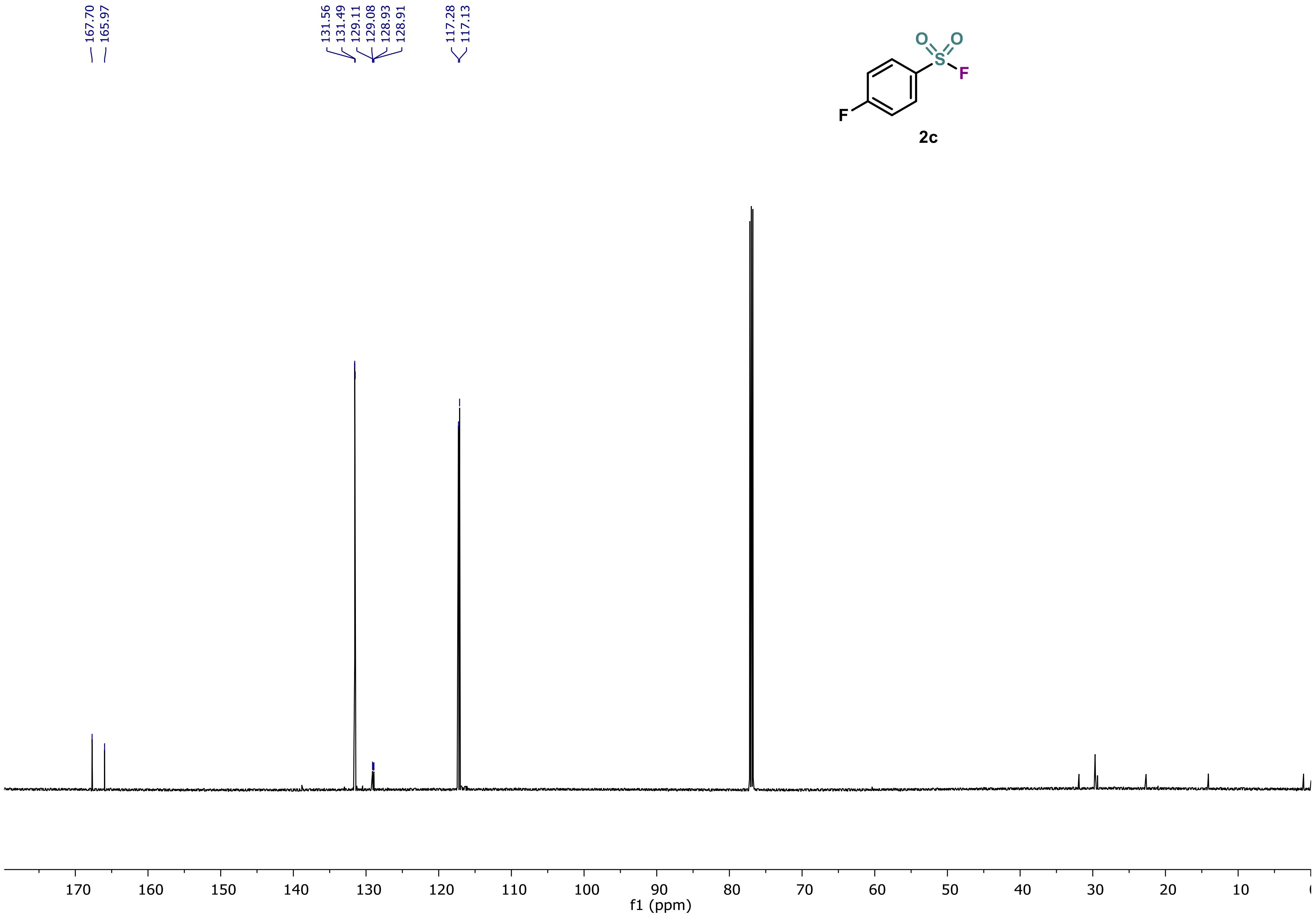

2c

^{19}F NMR (565 MHz, $CDCl_3$)

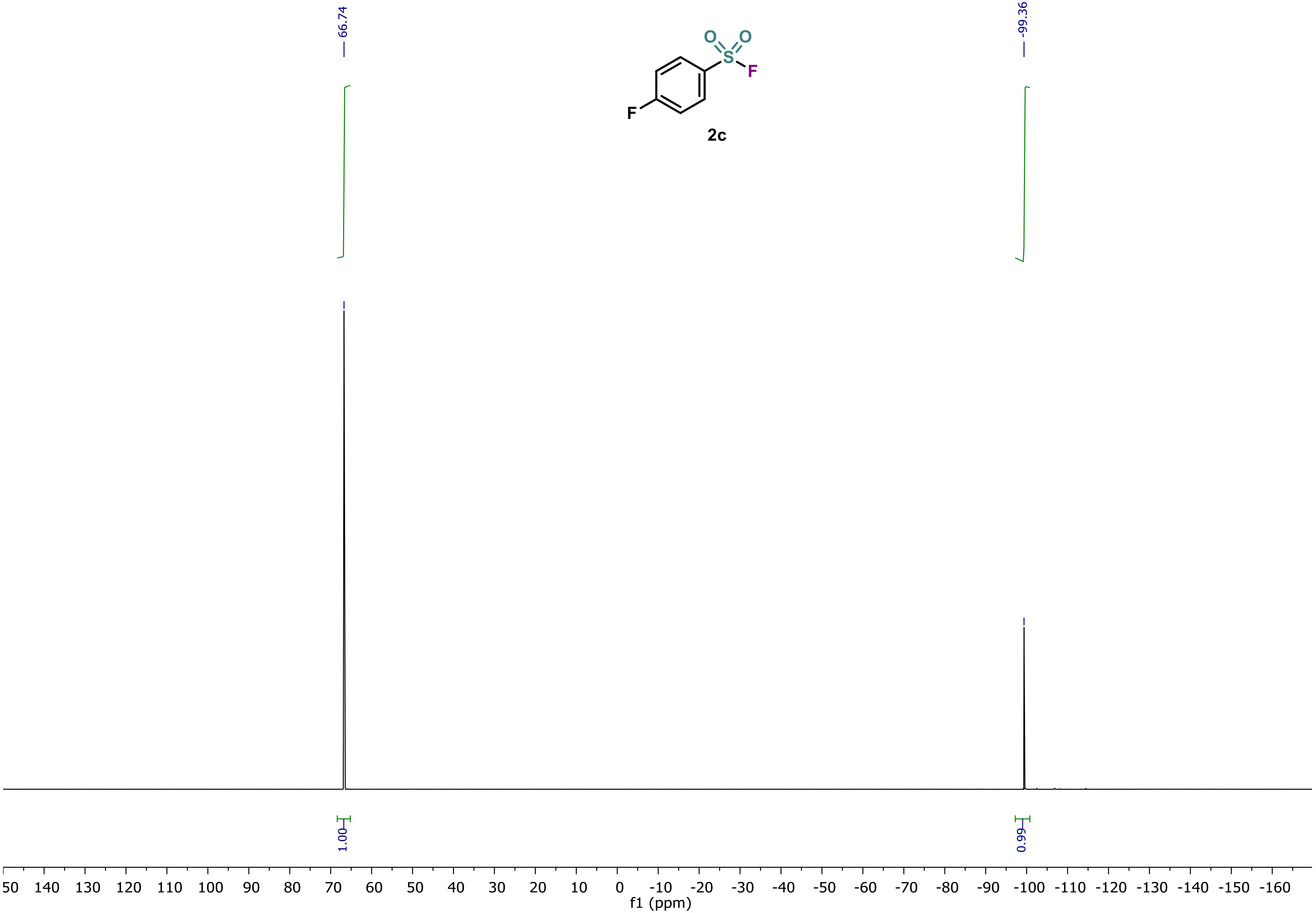

^{1}H NMR (600 MHz, $CDCl_3$)

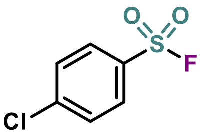

2d

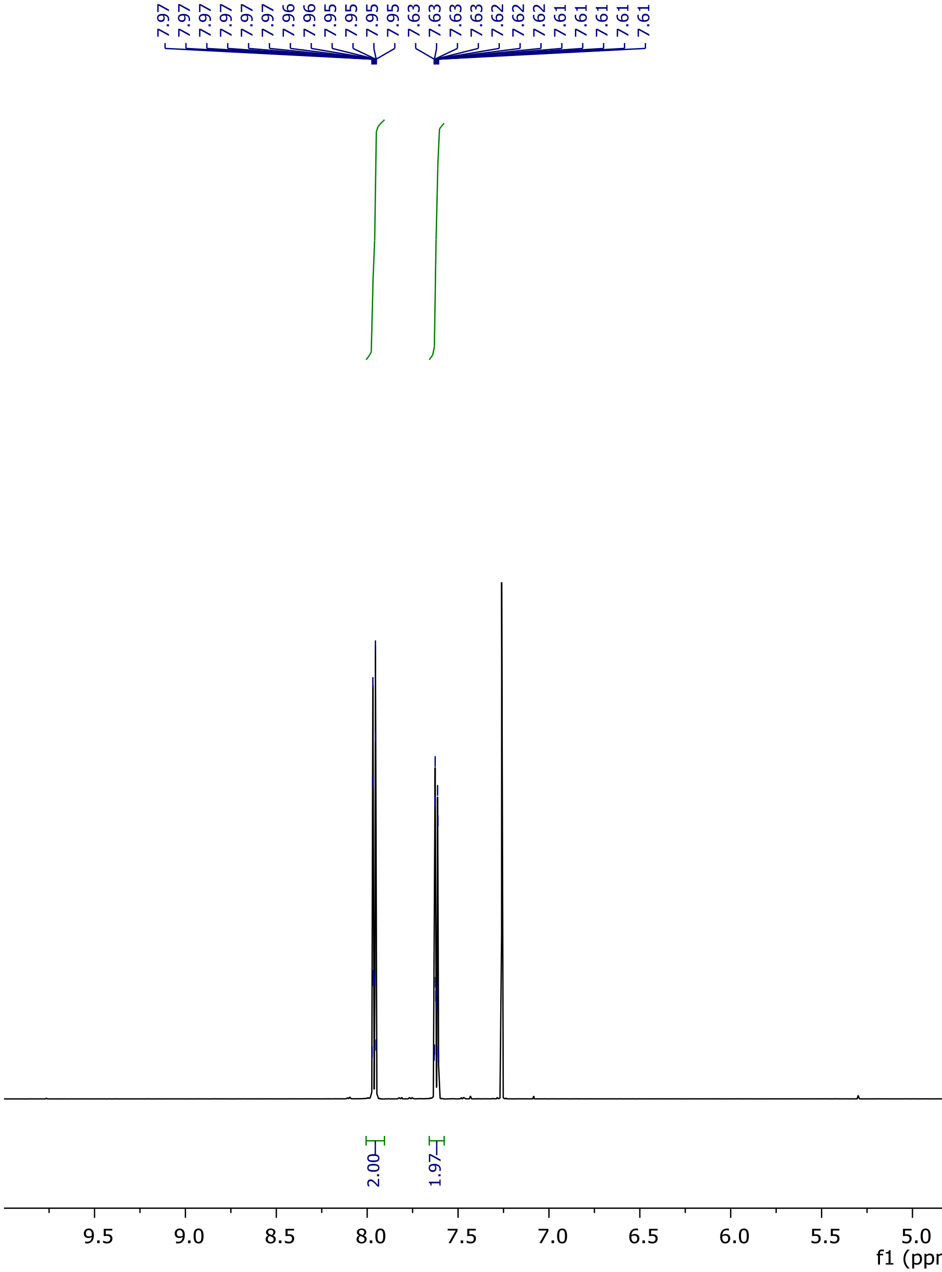

^{13}C NMR (151 MHz, $CDCl_3$)

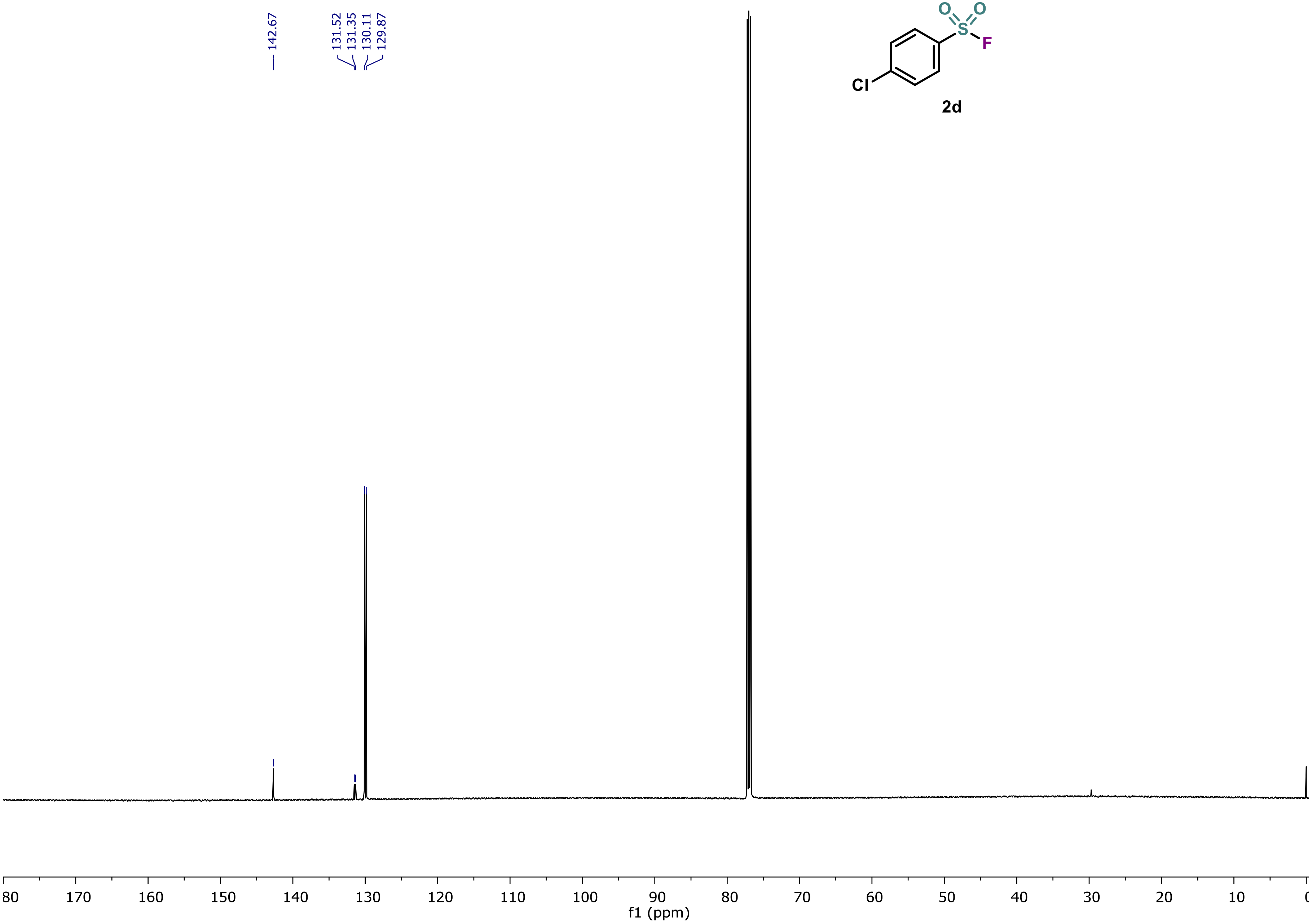

2d

^{19}F NMR (565 MHz, $CDCl_3$)

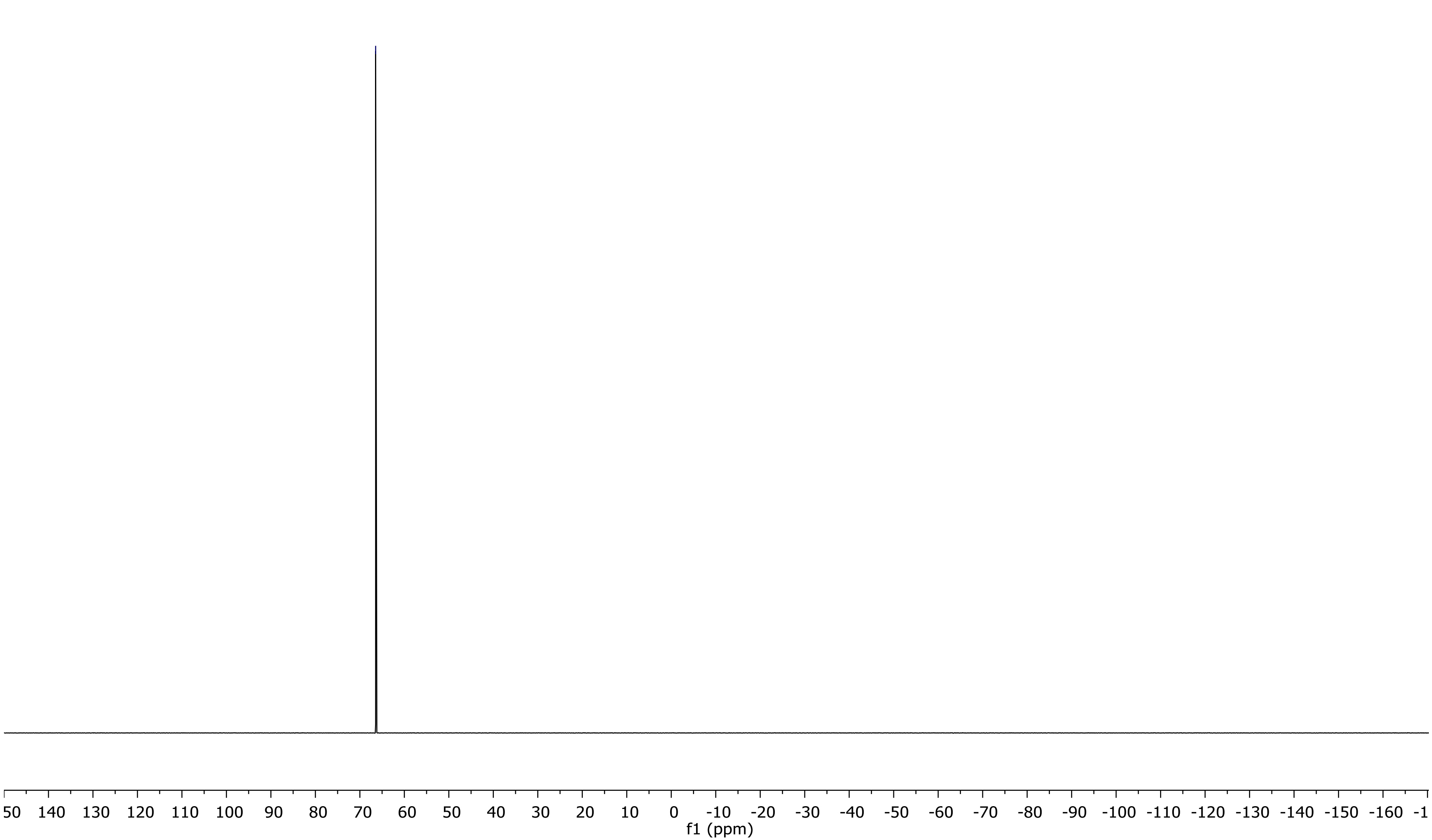

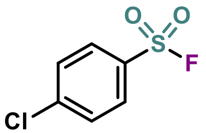

2d

^{1}H NMR (600 MHz, $CDCl_3$)

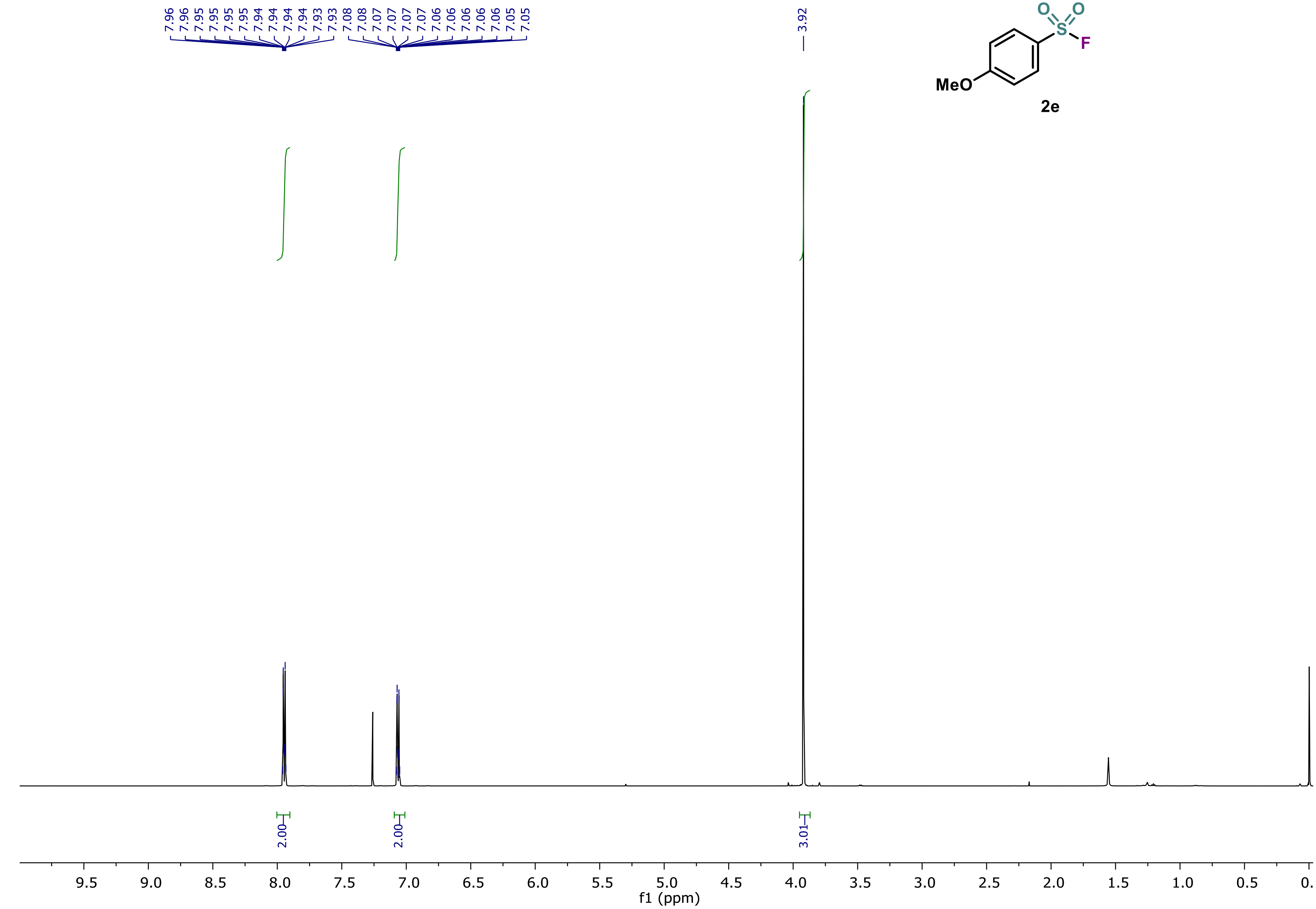

¹³C NMR (151 MHz, $CDCl_3$)

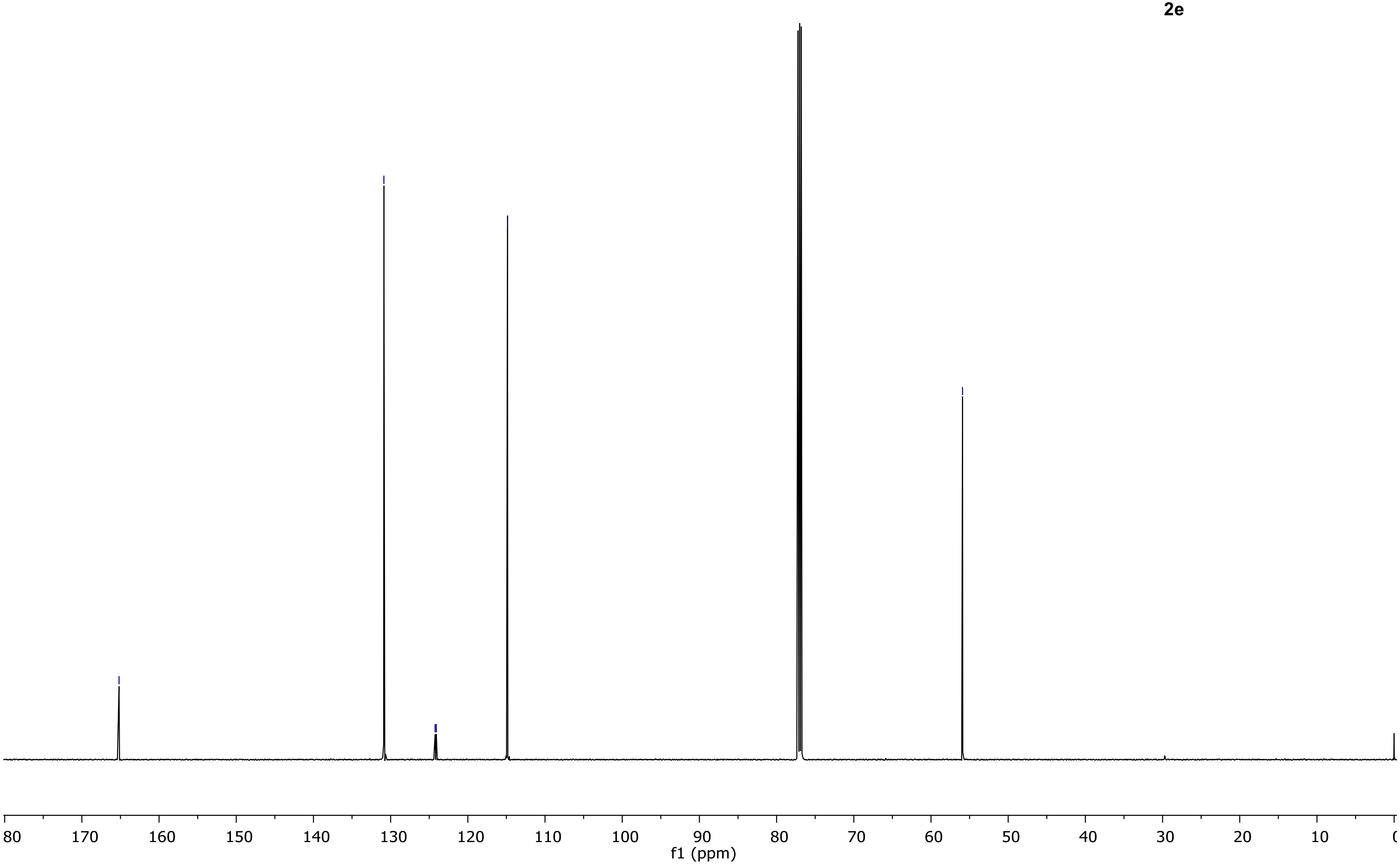

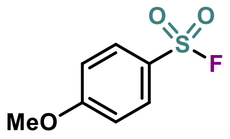

2e

^{19}F NMR (565 MHz, $CDCl_3$)

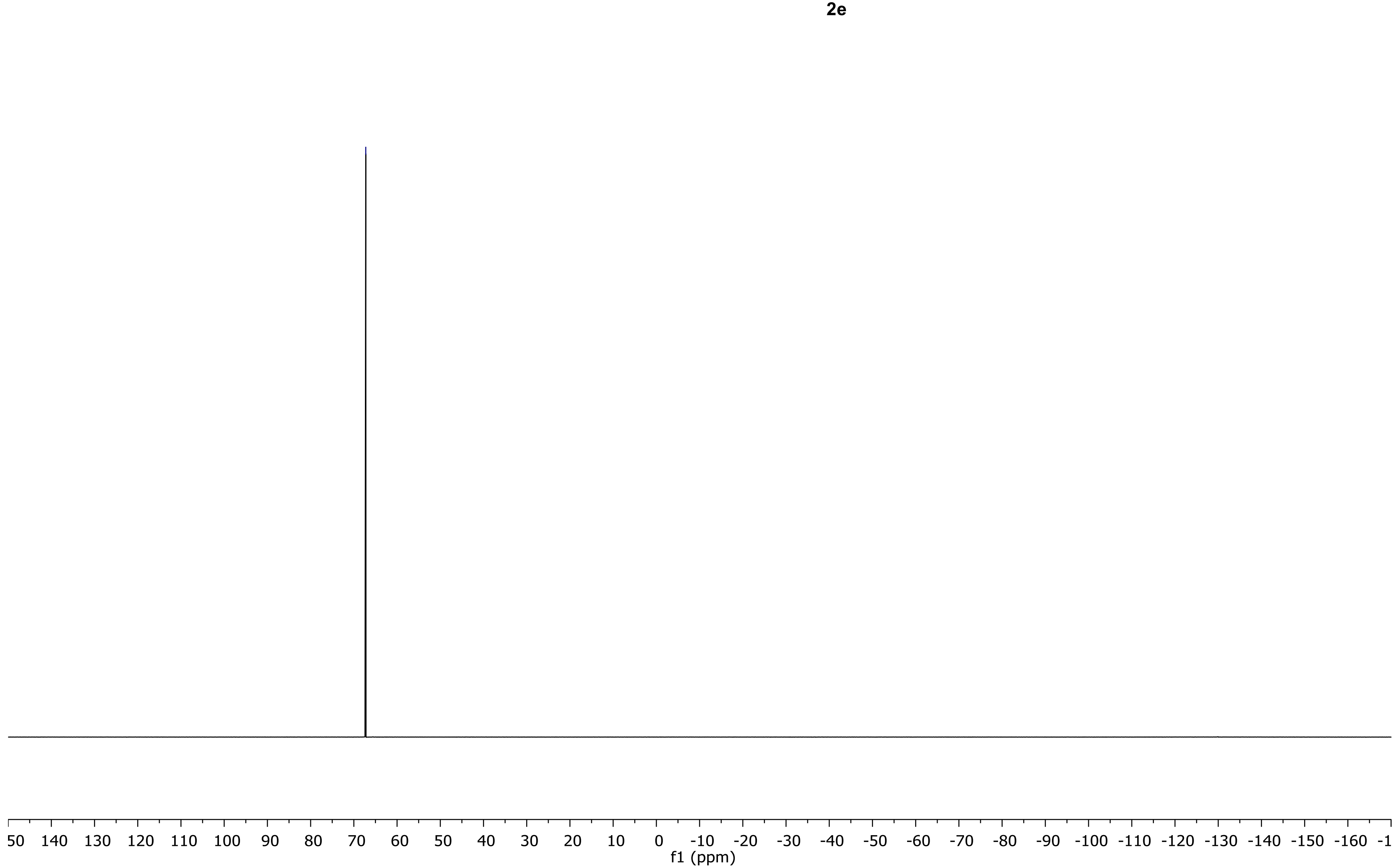

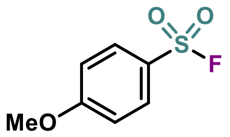

2e

^{1}H NMR (600 MHz, $CDCl_3$)

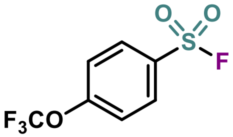

2f

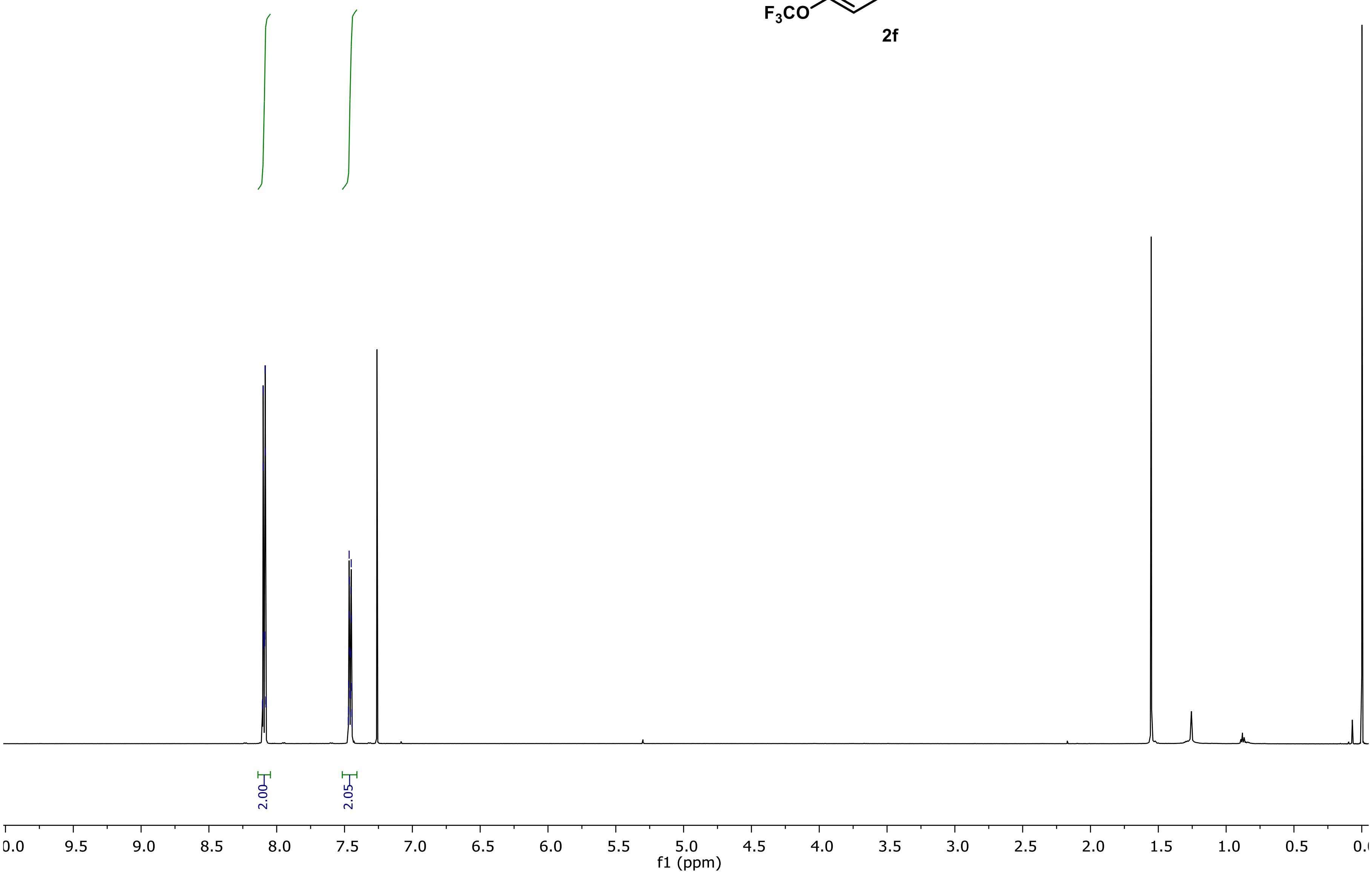

^{13}C NMR (151 MHz, $CDCl_3$)

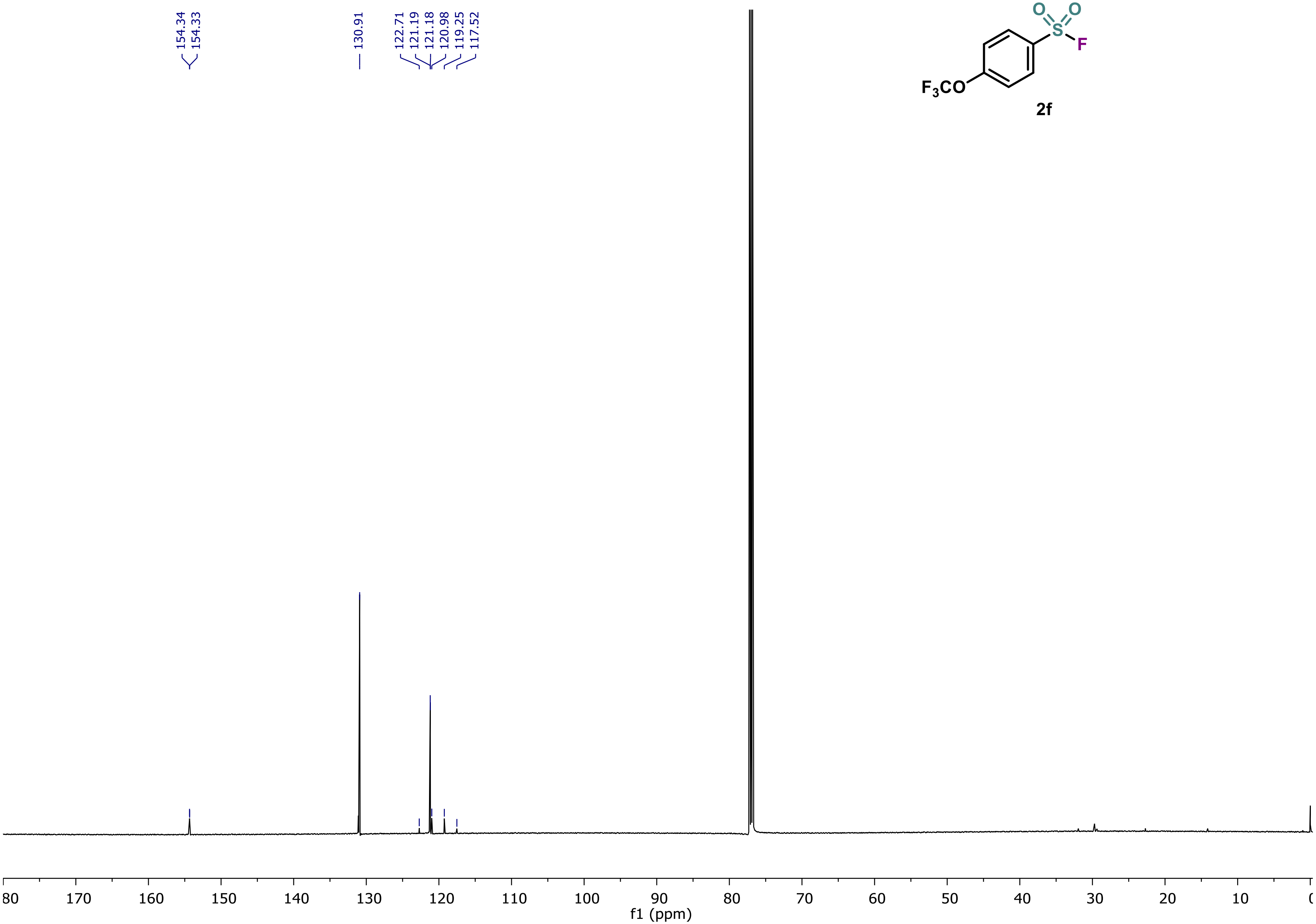

^{19}F NMR (565 MHz, $CDCl_3$)

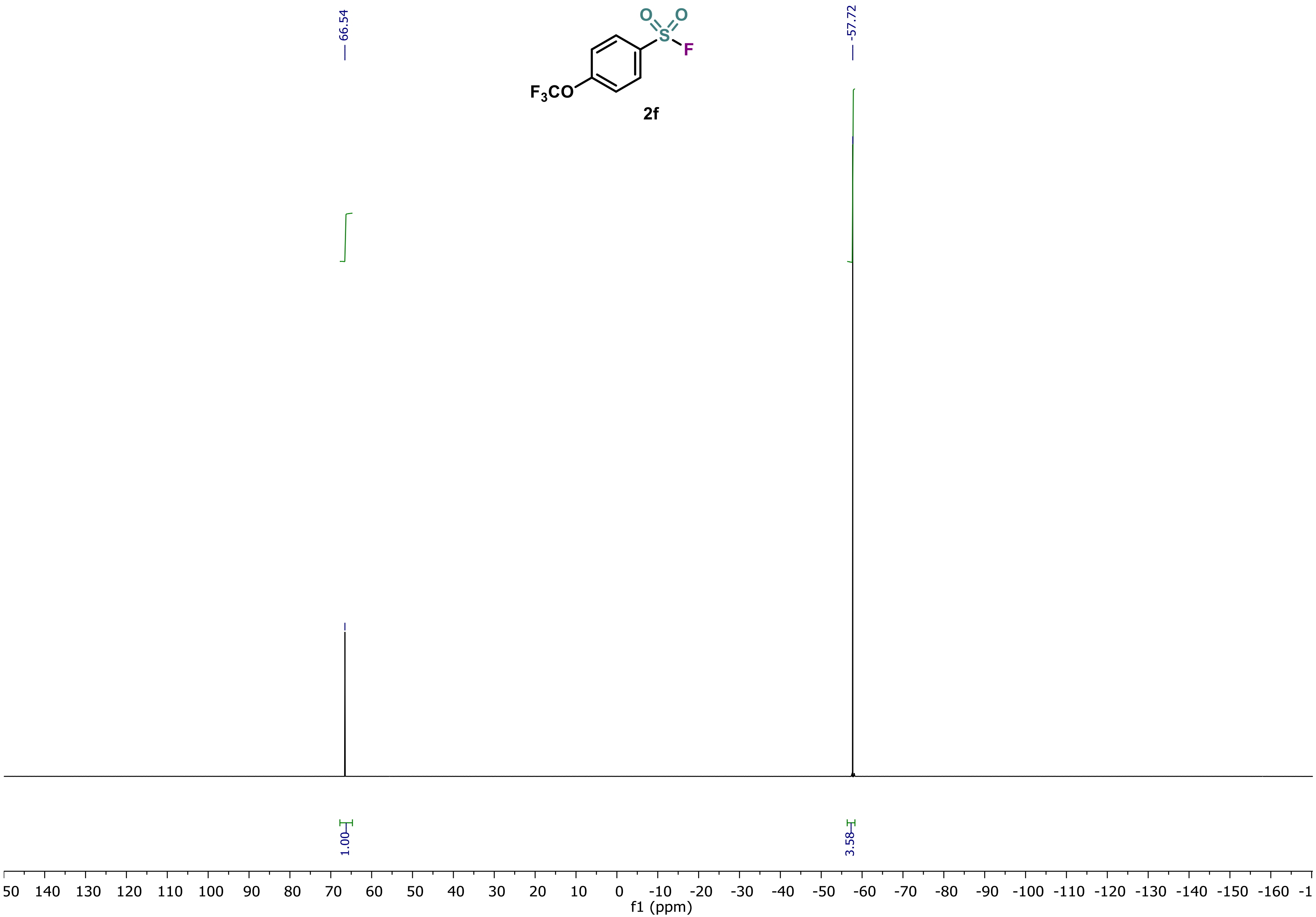

^{1}H NMR (600 MHz, CDCl$_3$)

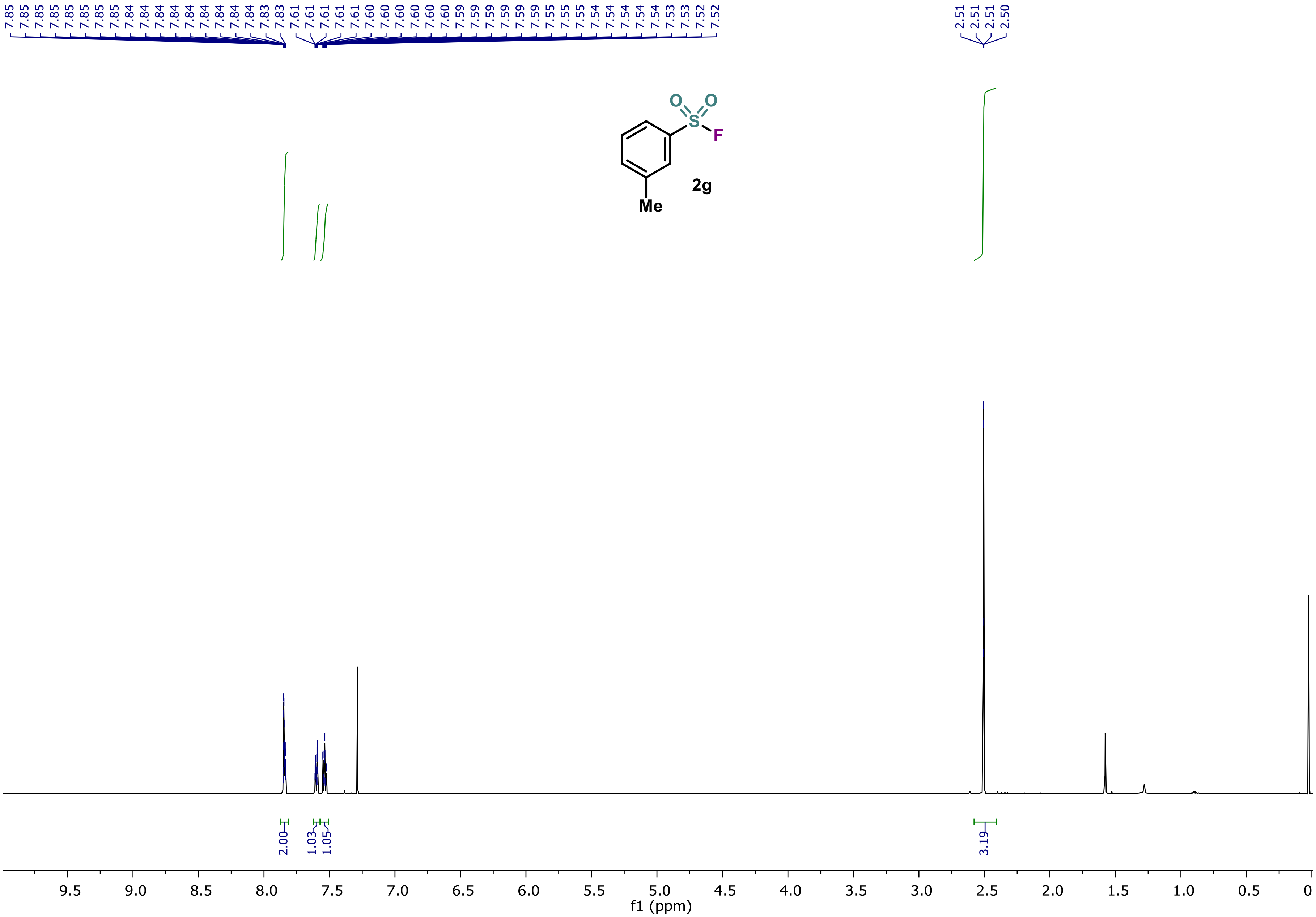

^{13}C NMR (151 MHz, $CDCl_3$)

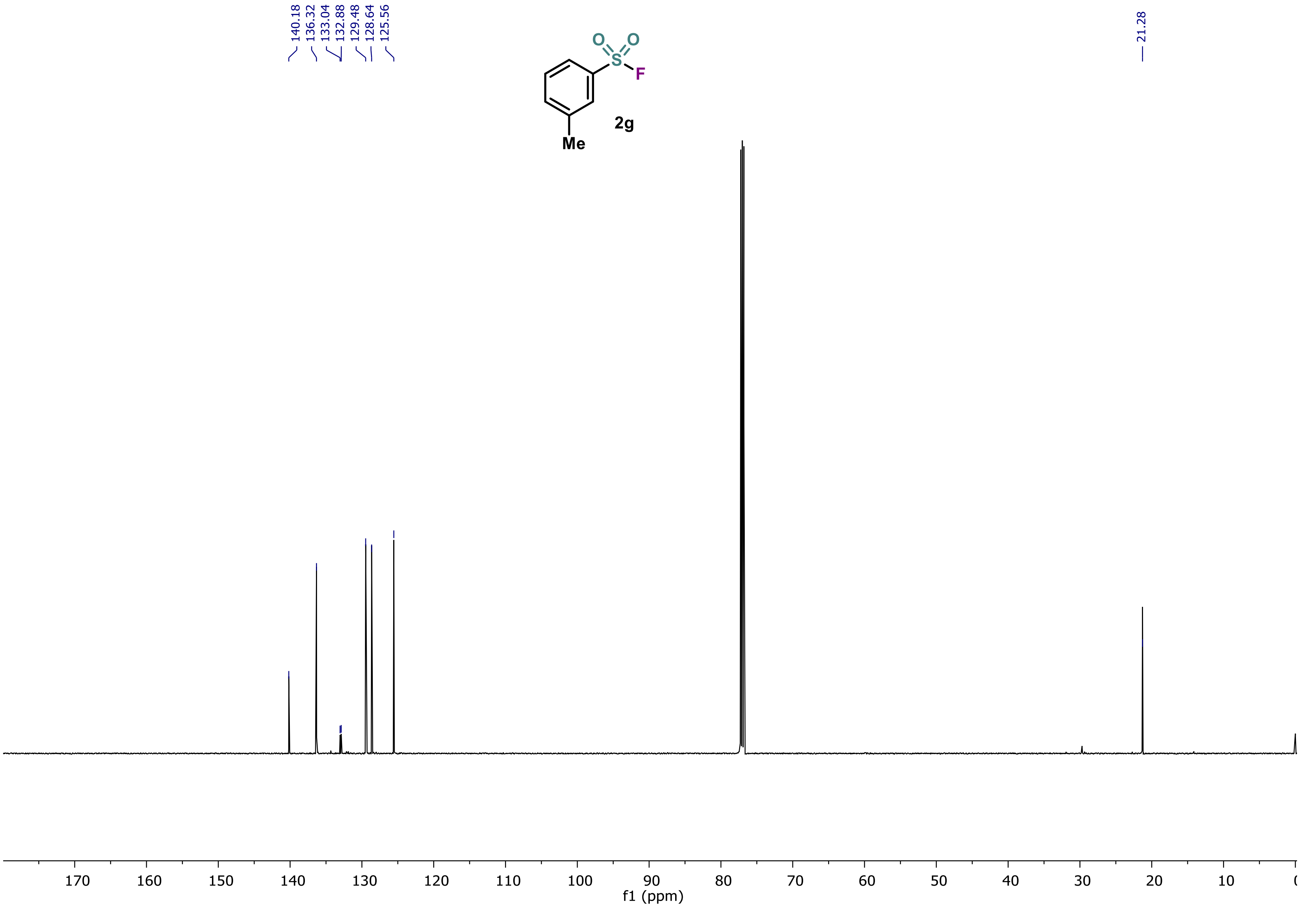

^{19}F NMR (565 MHz, $CDCl_3$)

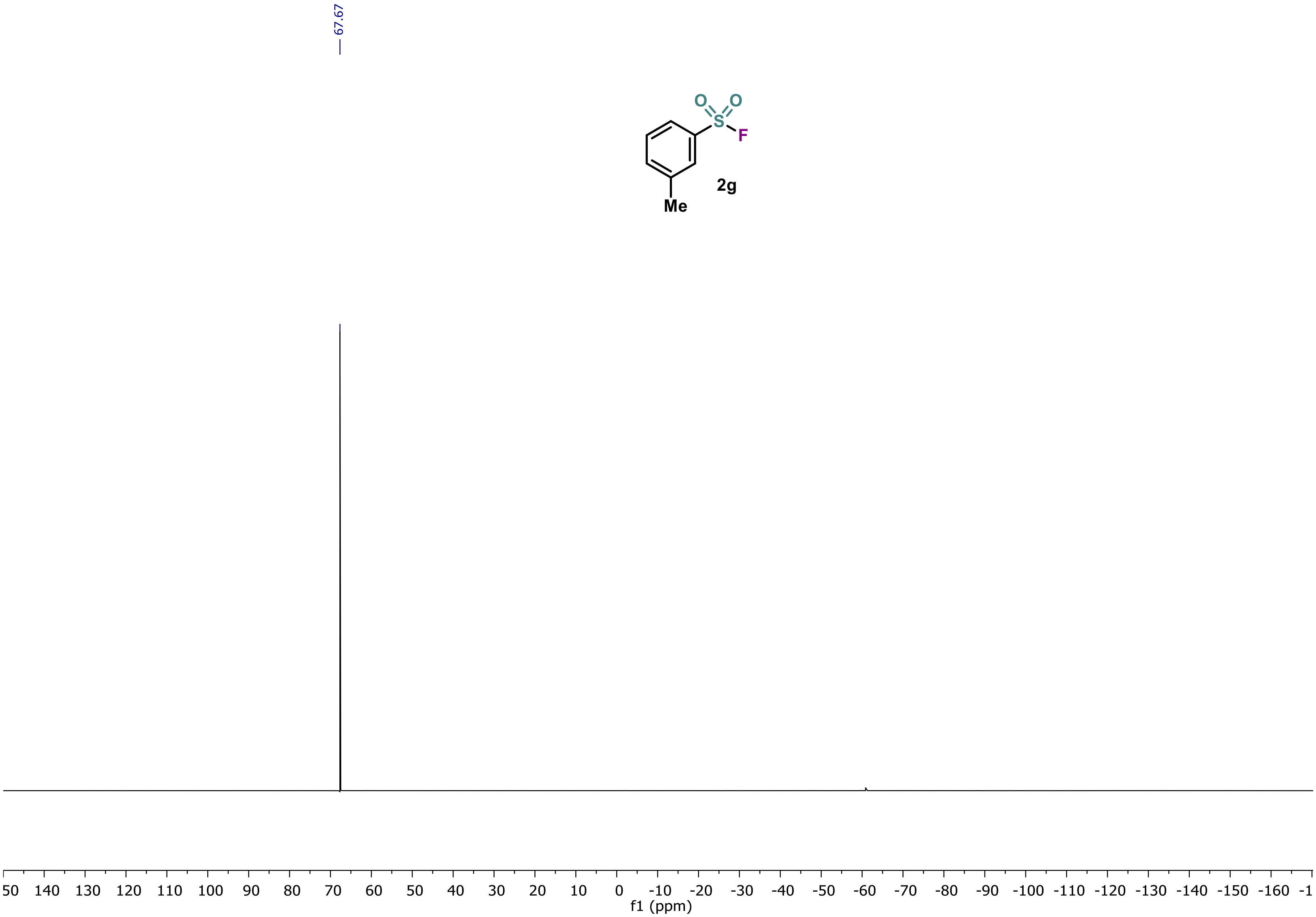

^{1}H NMR (600 MHz, $CDCl_3$)

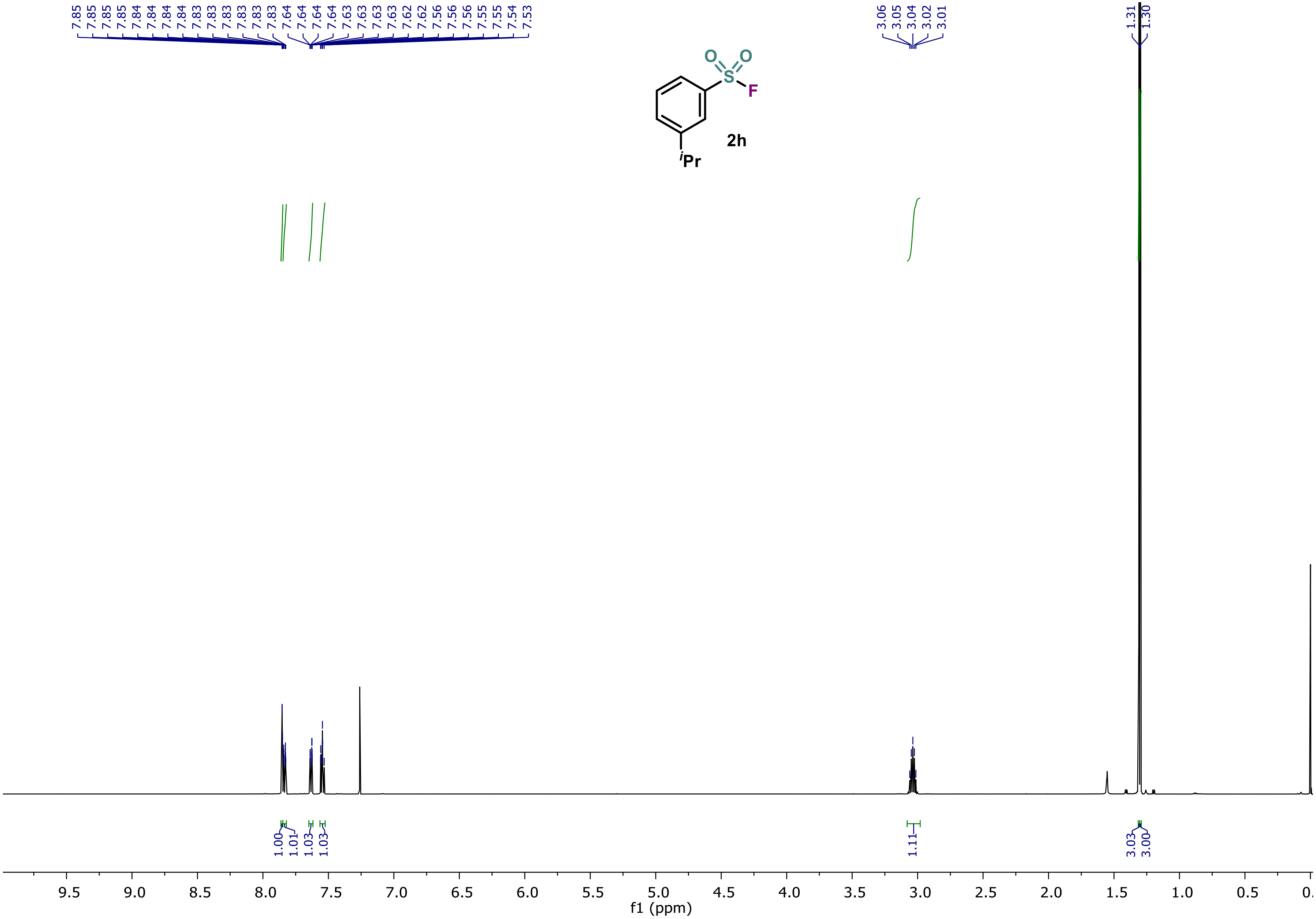

^{13}C NMR (151 MHz, $CDCl_3$)

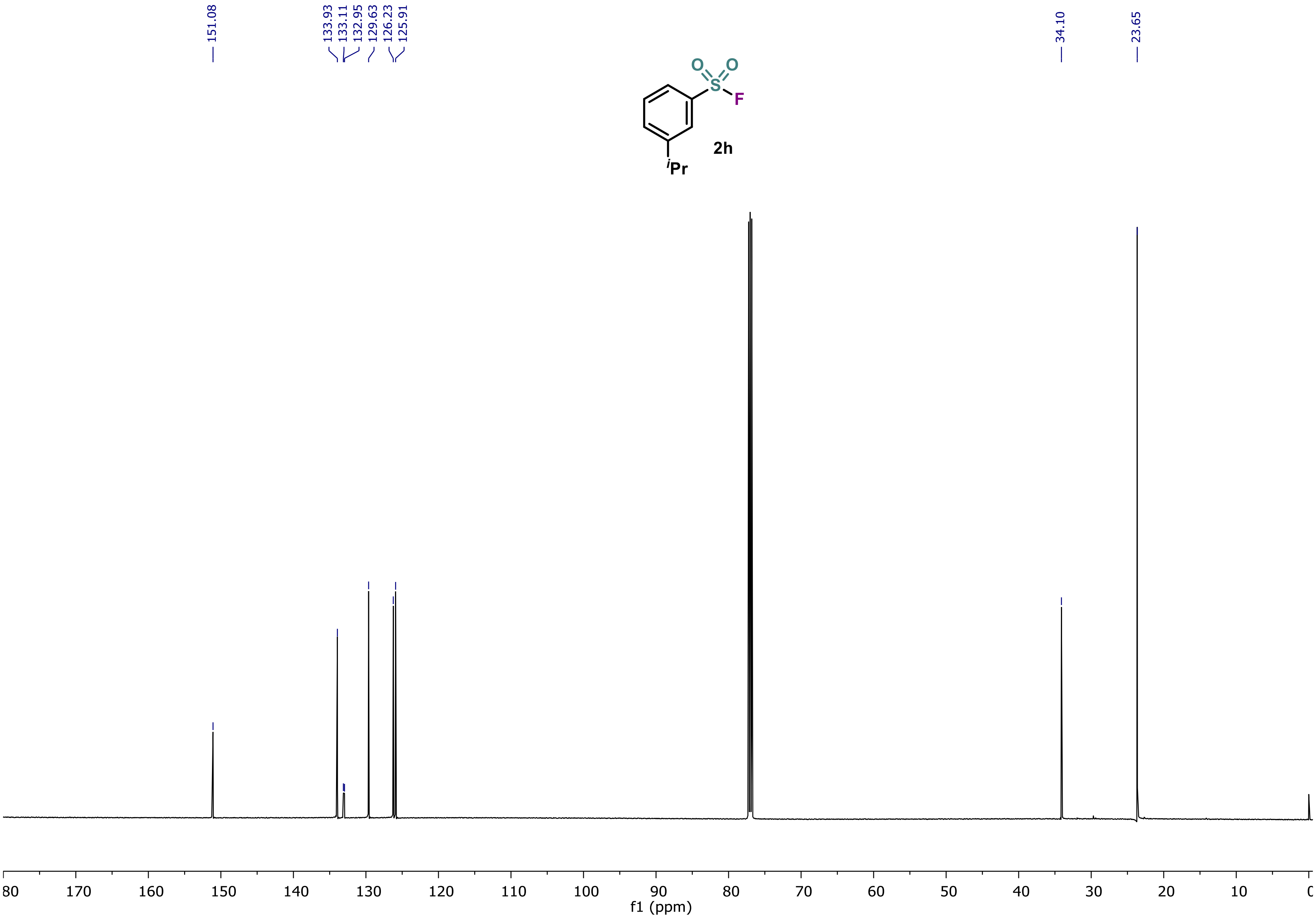

^{19}F NMR (565 MHz, $CDCl_3$)

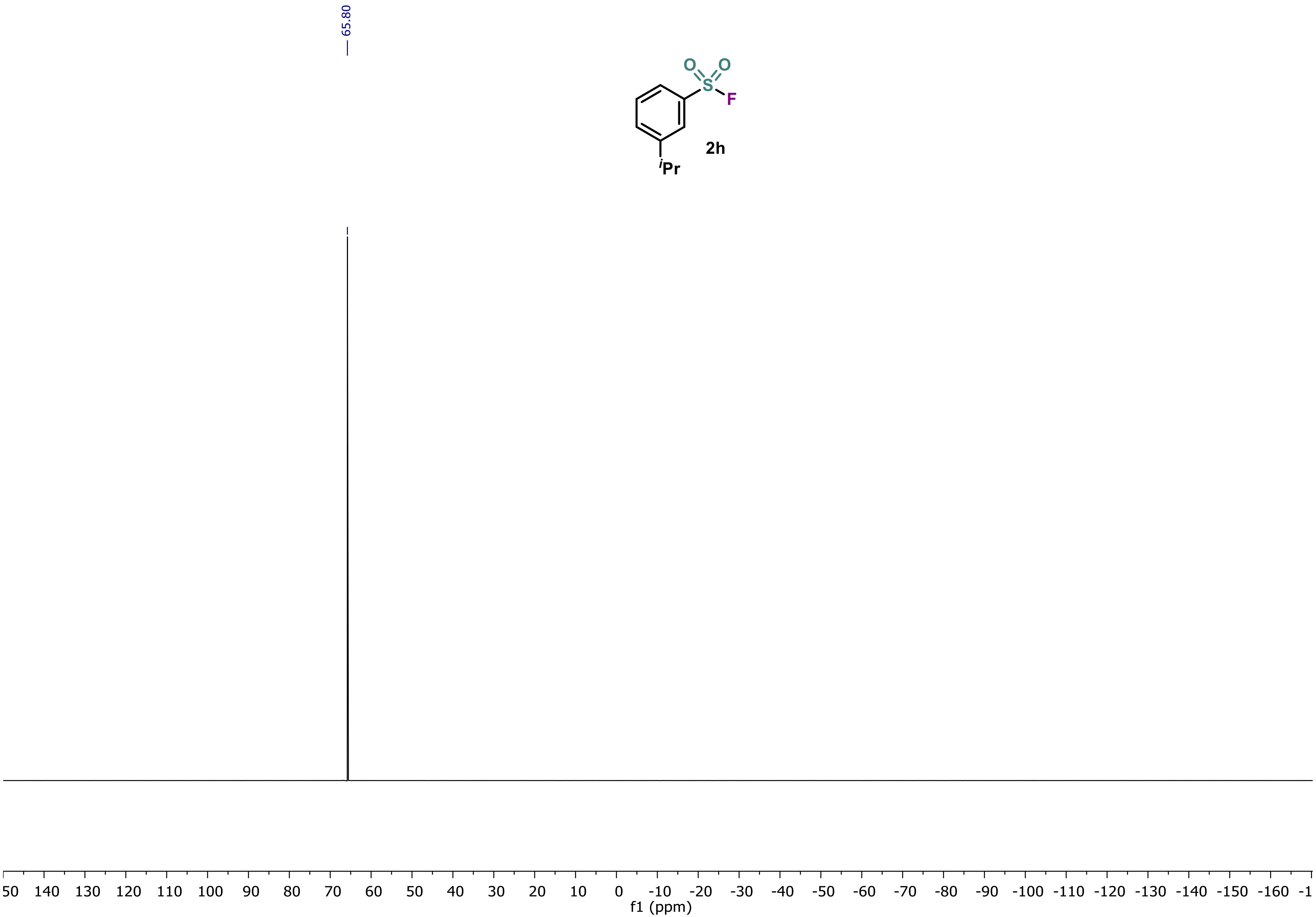

^{1}H NMR (600 MHz, CDCl$_3$)

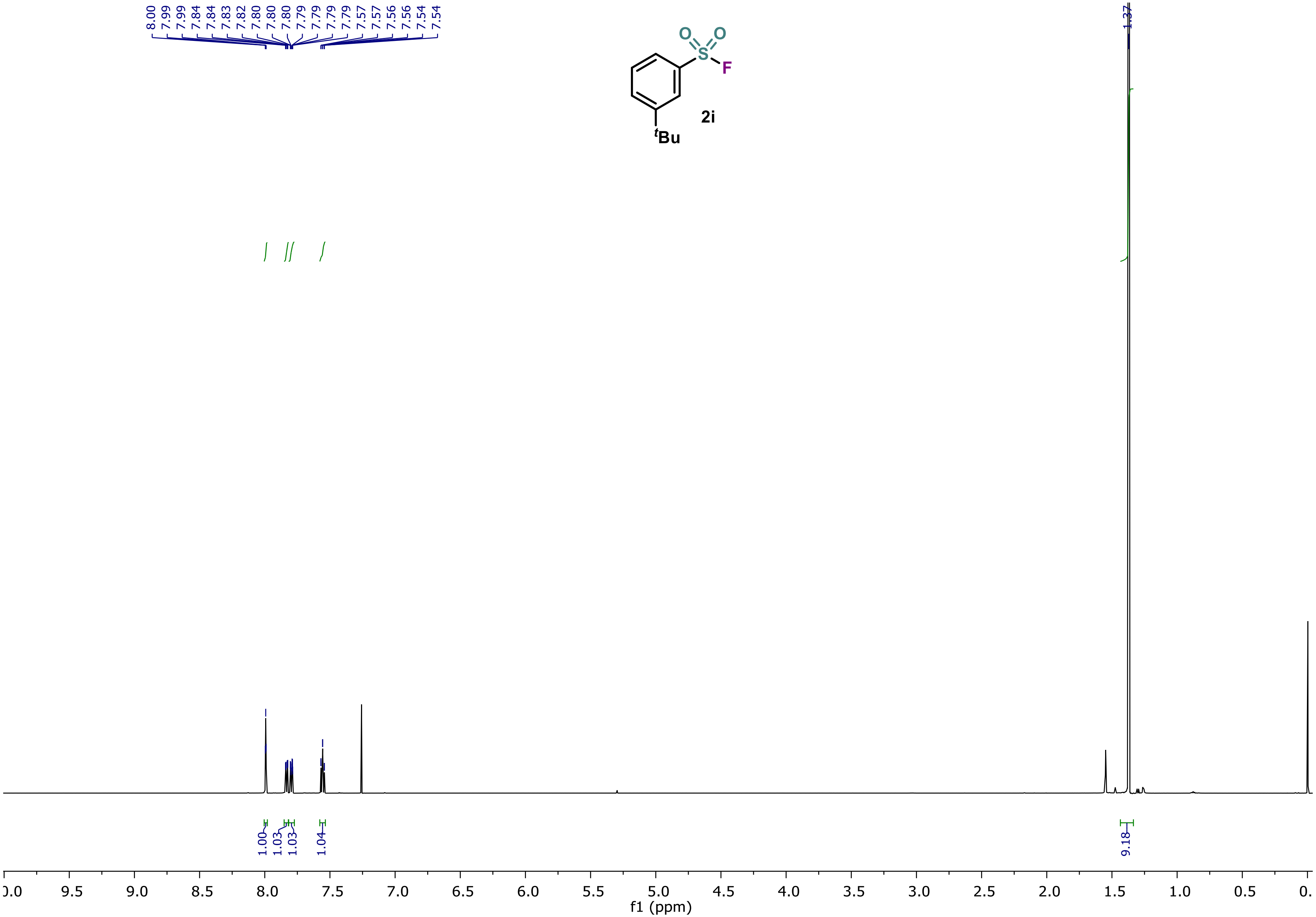

^{13}C NMR (151 MHz, $CDCl_3$)

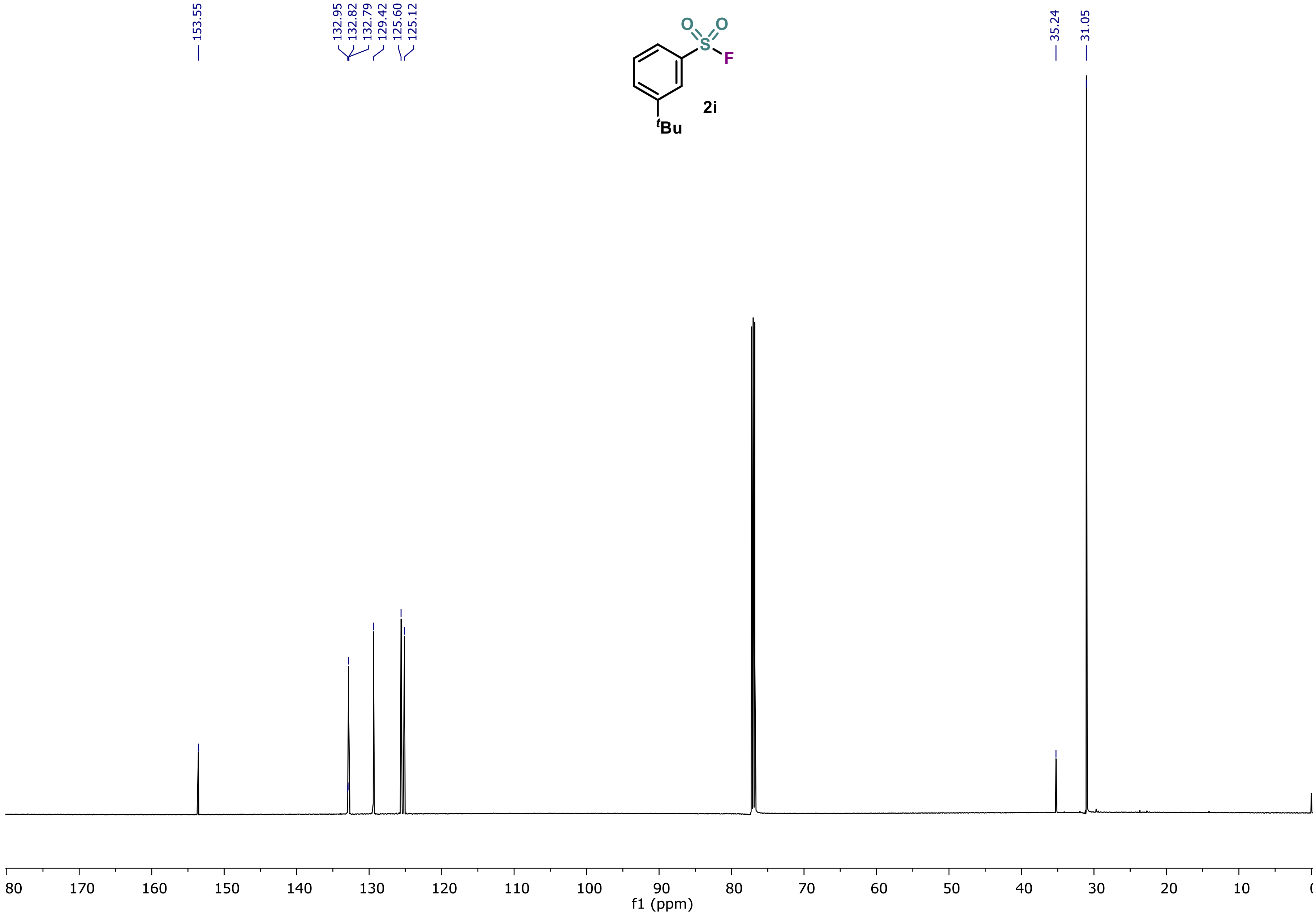

^{19}F NMR (565 MHz, $CDCl_3$)

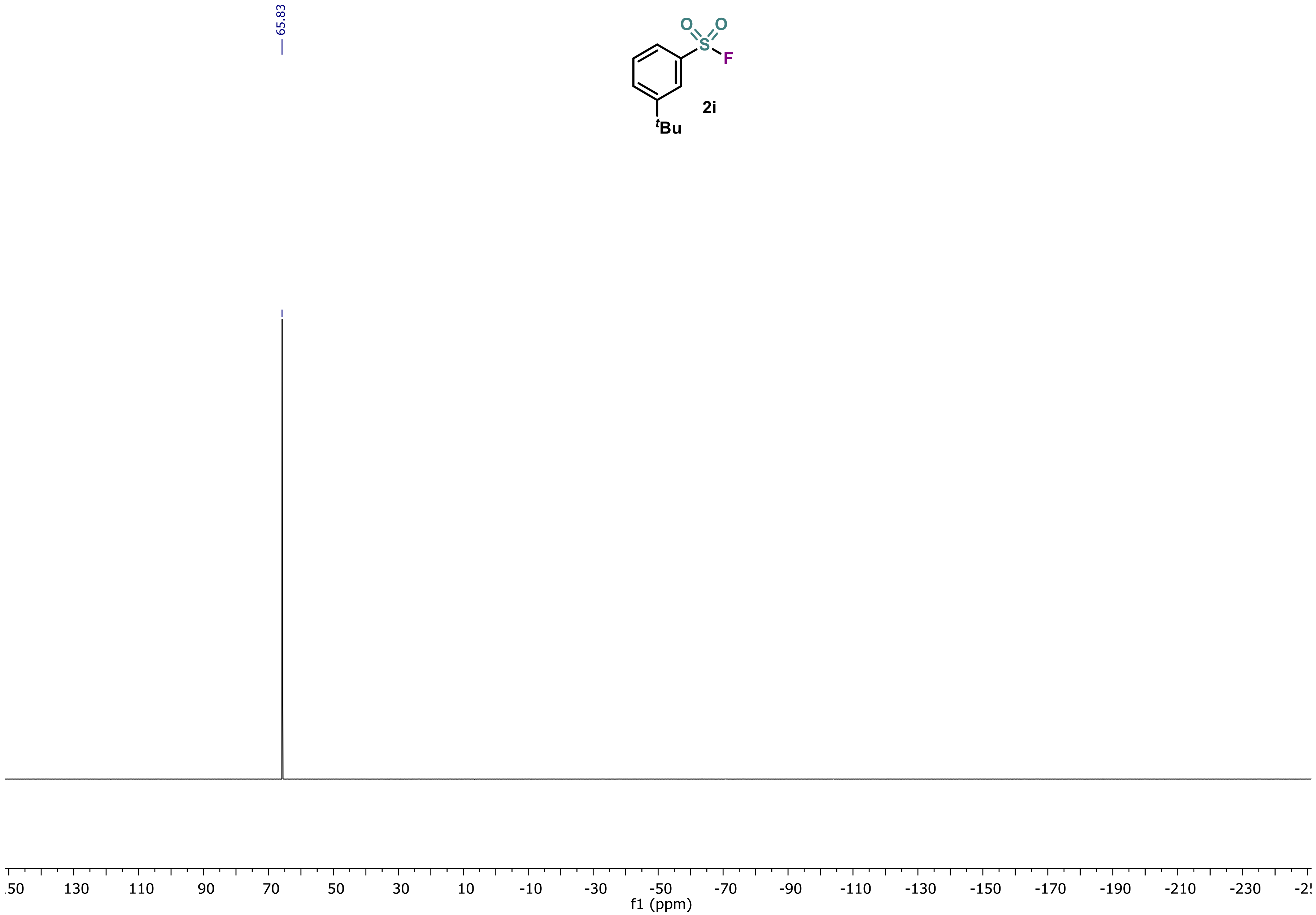

^{1}H NMR (600 MHz, CDCl$_3$)

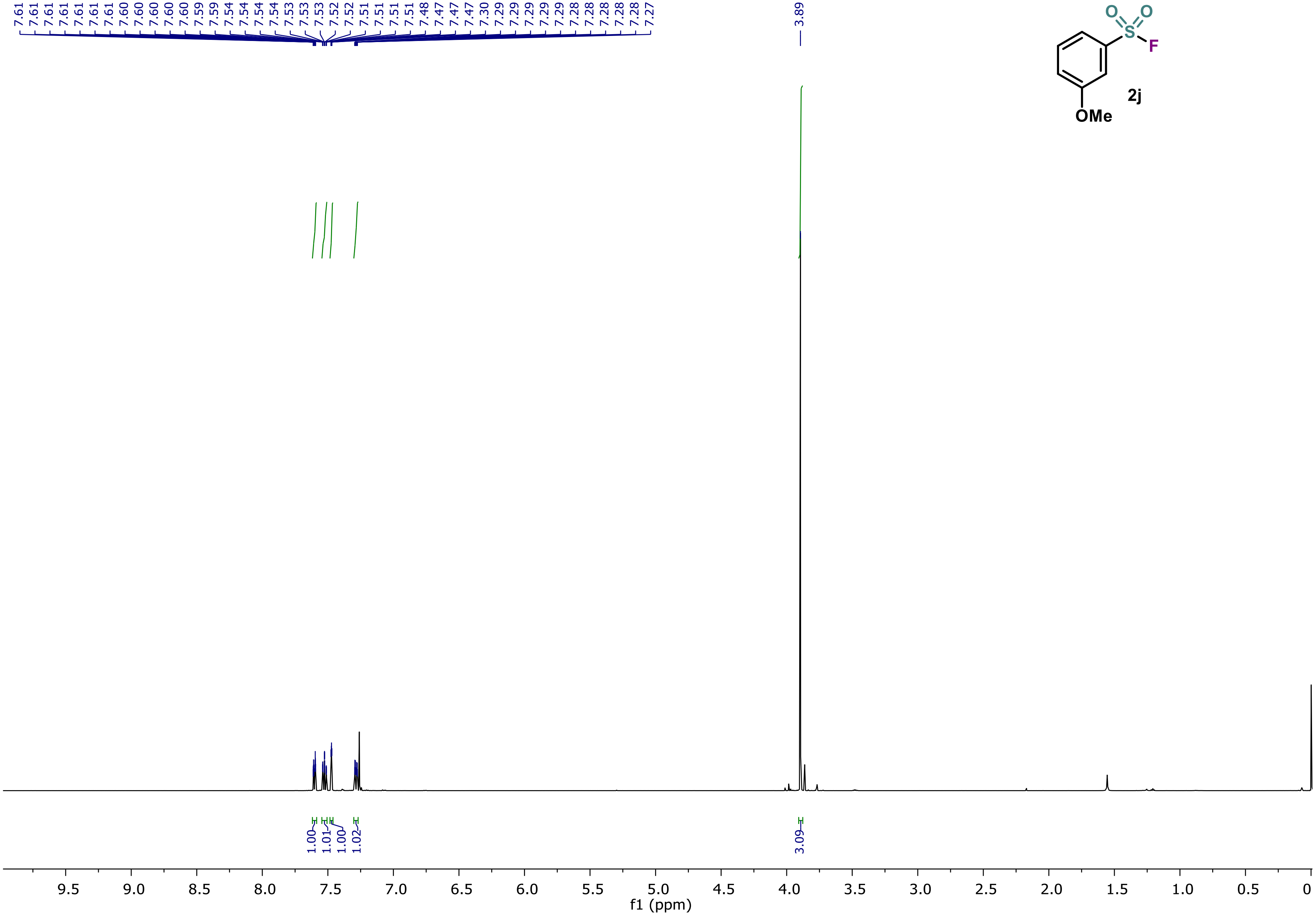

^{13}C NMR (151 MHz, $CDCl_3$)

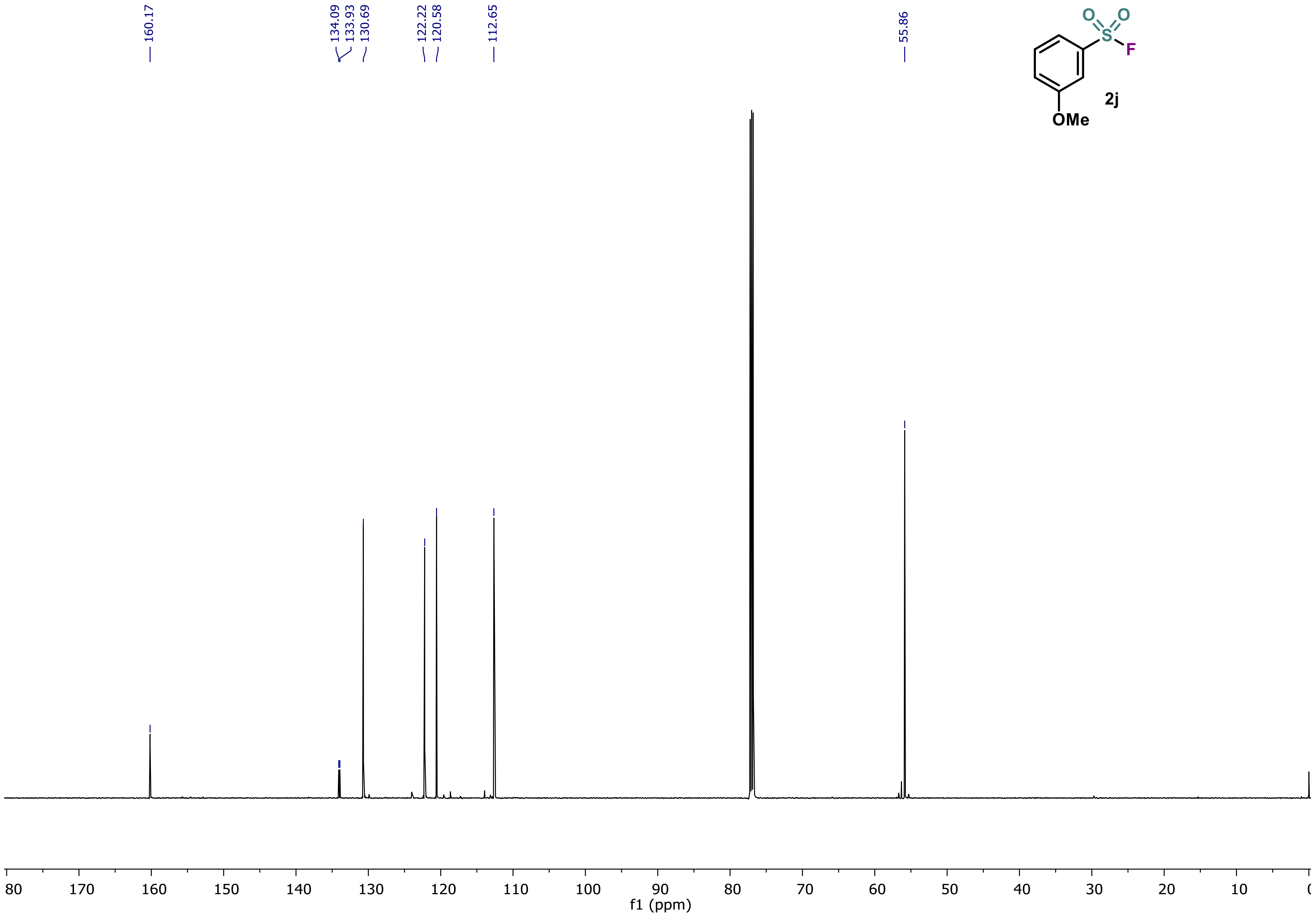

2j

^{19}F NMR (565 MHz, $CDCl_3$)

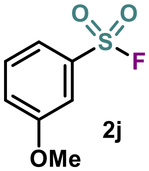

2j

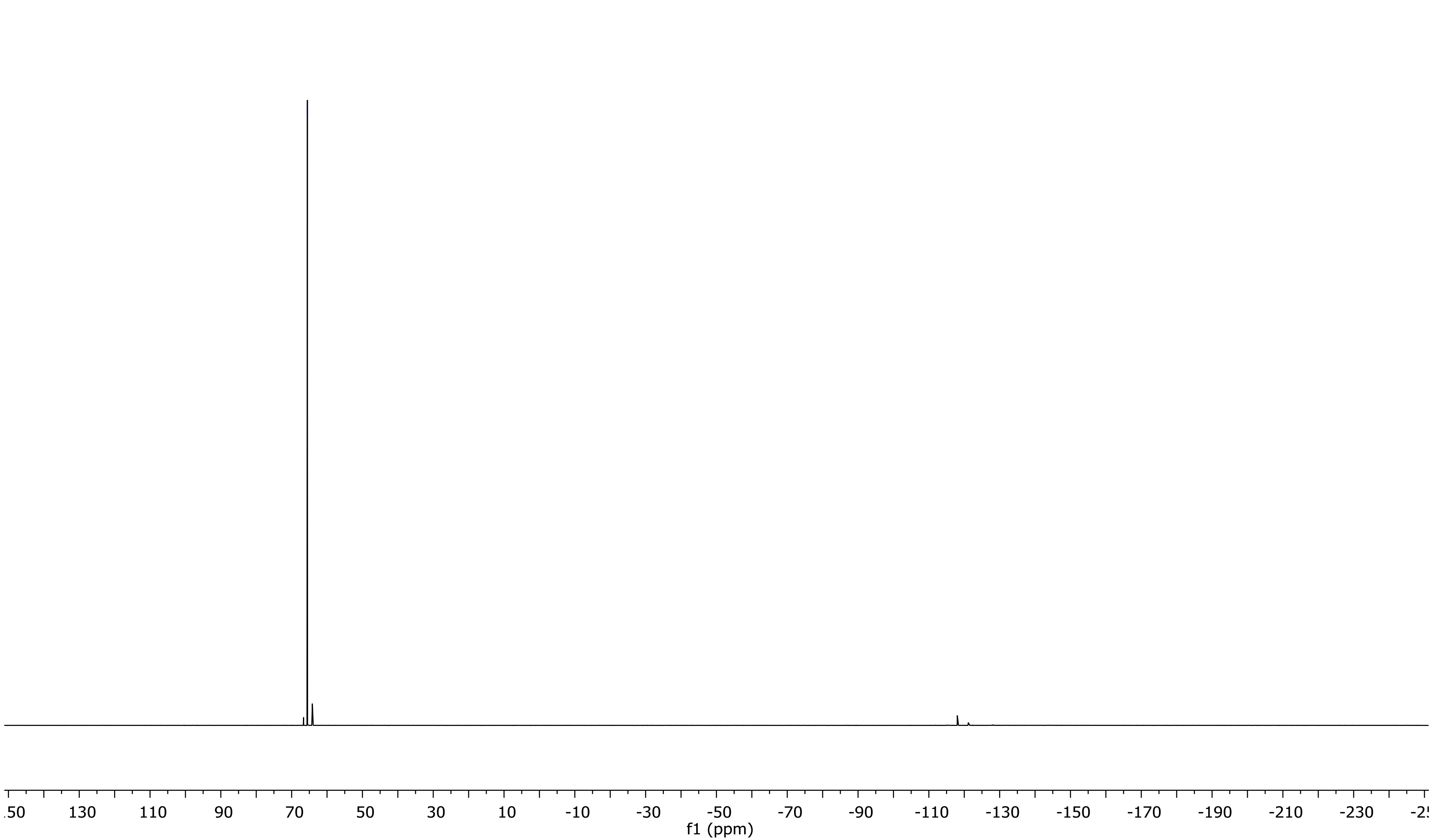

^1H NMR (600 MHz, $CDCl_3$)

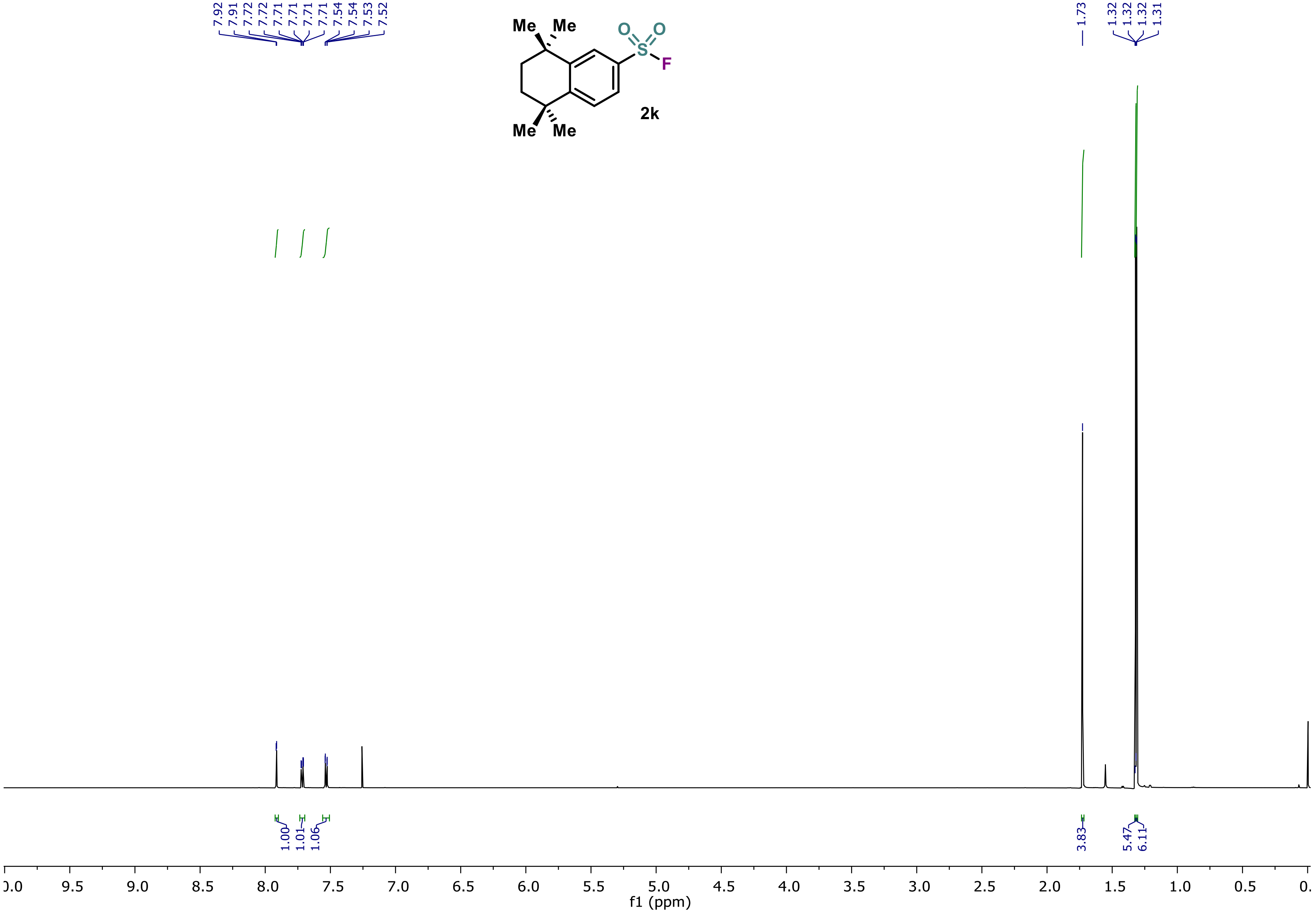

^{13}C NMR (151 MHz, $CDCl_3$)

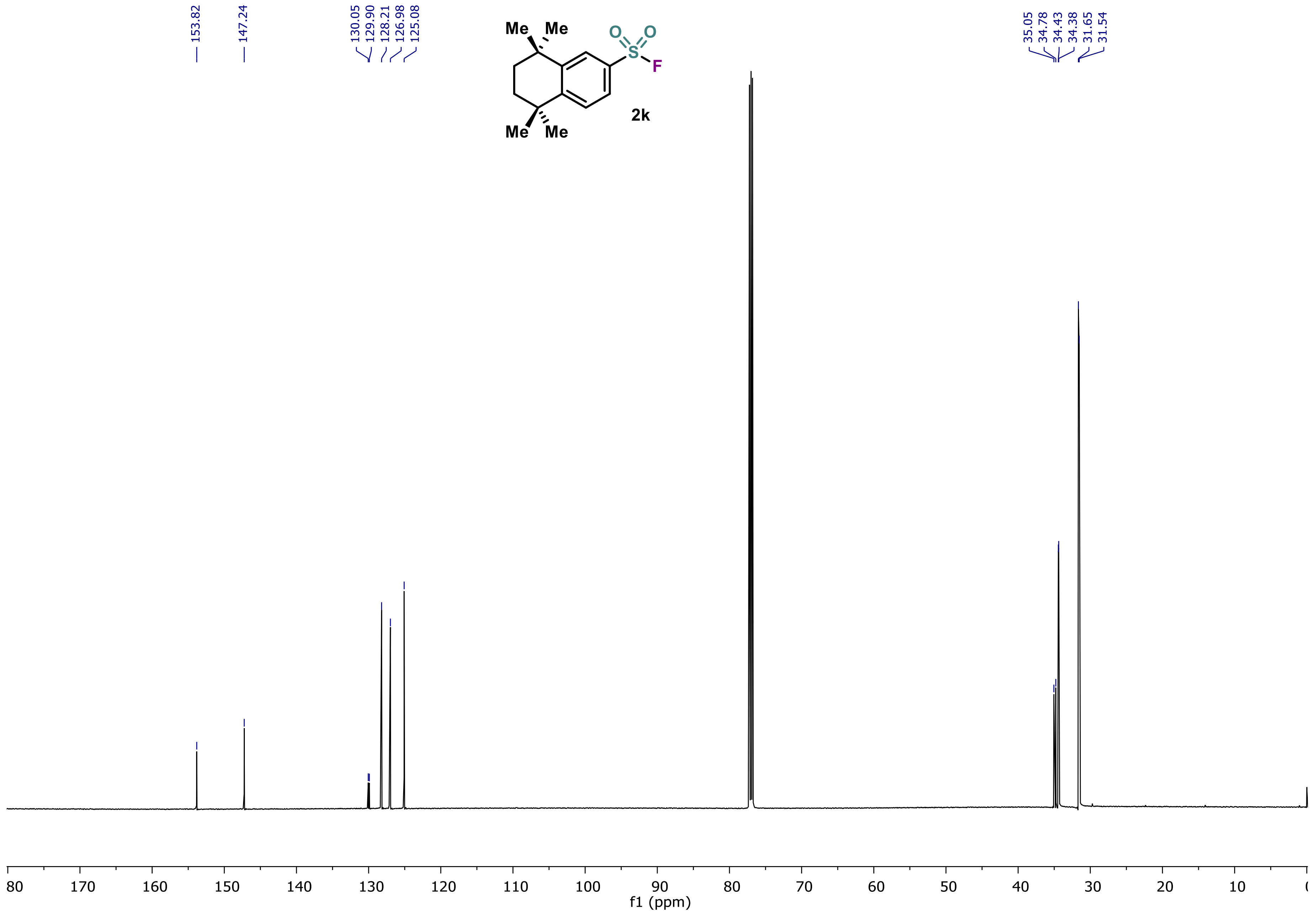

^{19}F NMR (565 MHz, CDCl$_3$)

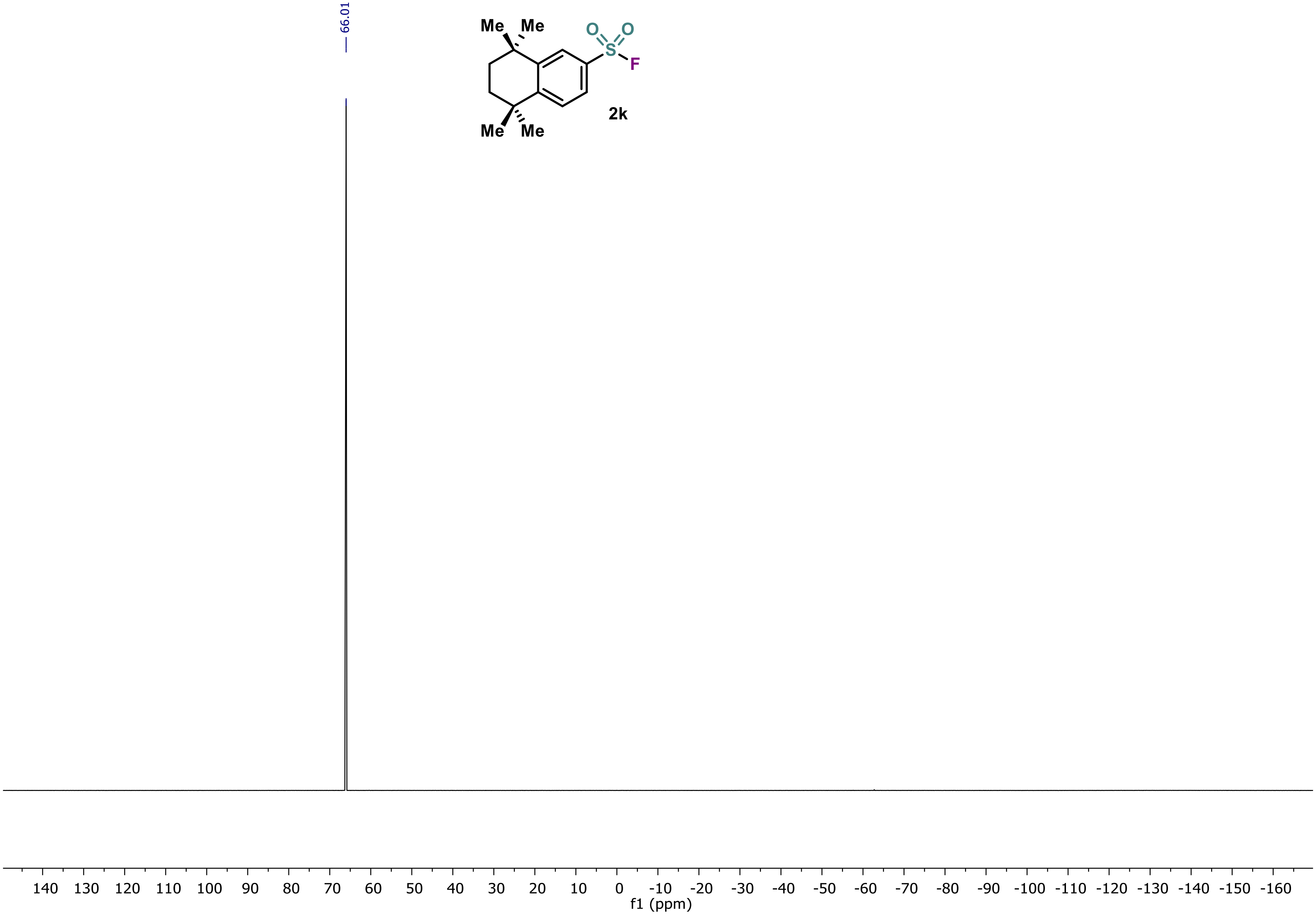

2k

^{1}H NMR (600 MHz, CDCl$_3$)

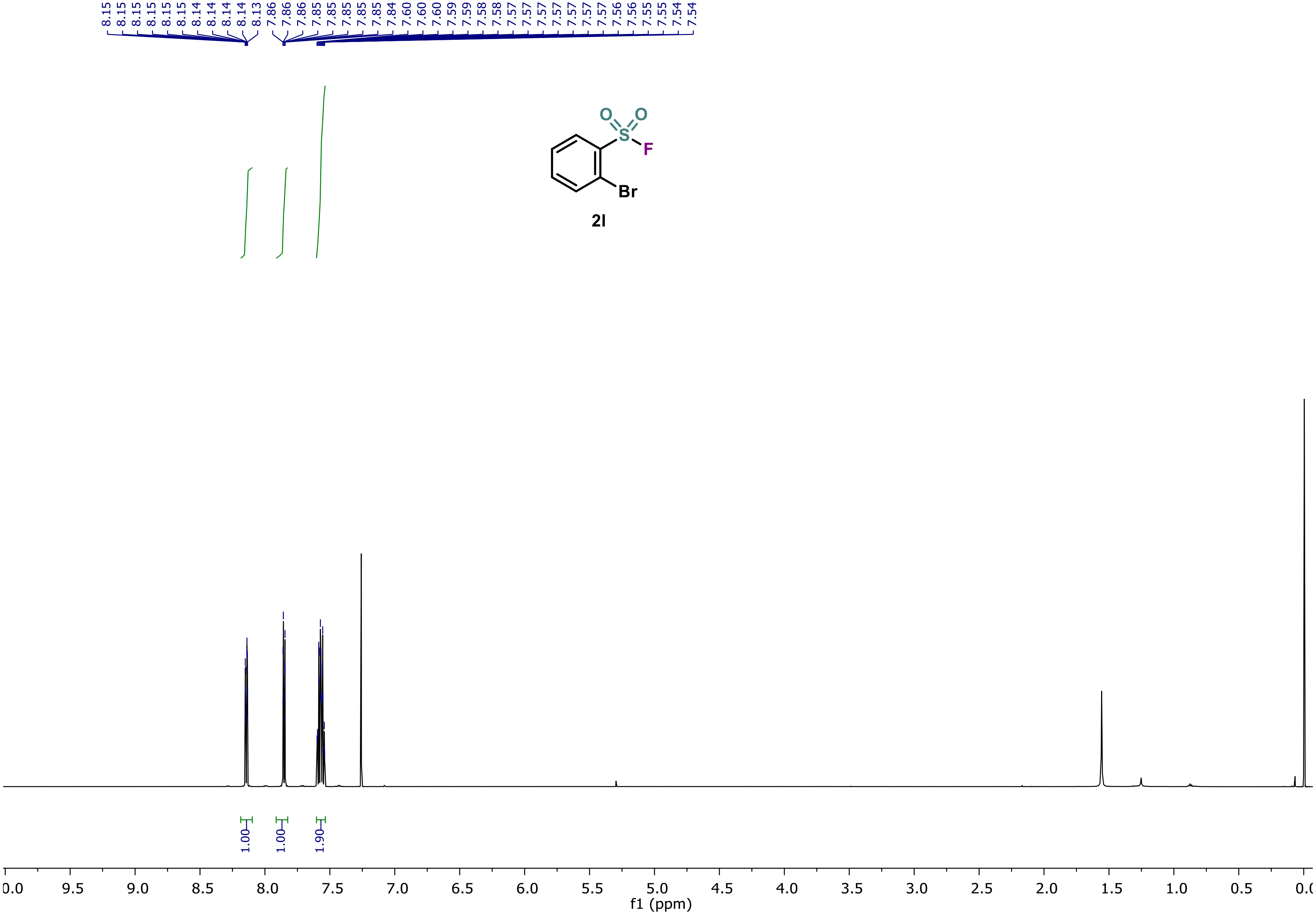

^{13}C NMR (151 MHz, CDCl$_3$)

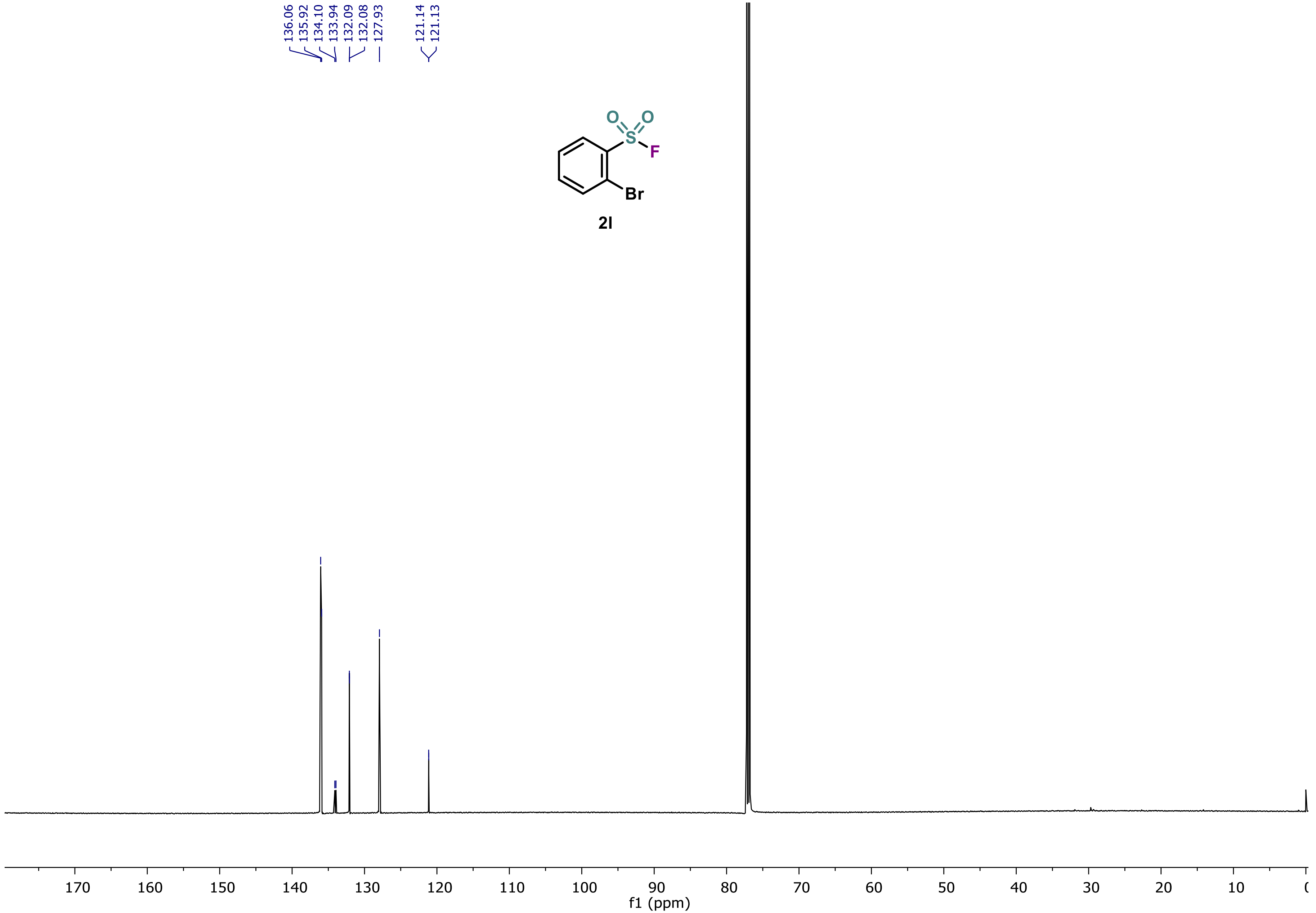

^{19}F NMR (565 MHz, $CDCl_3$)

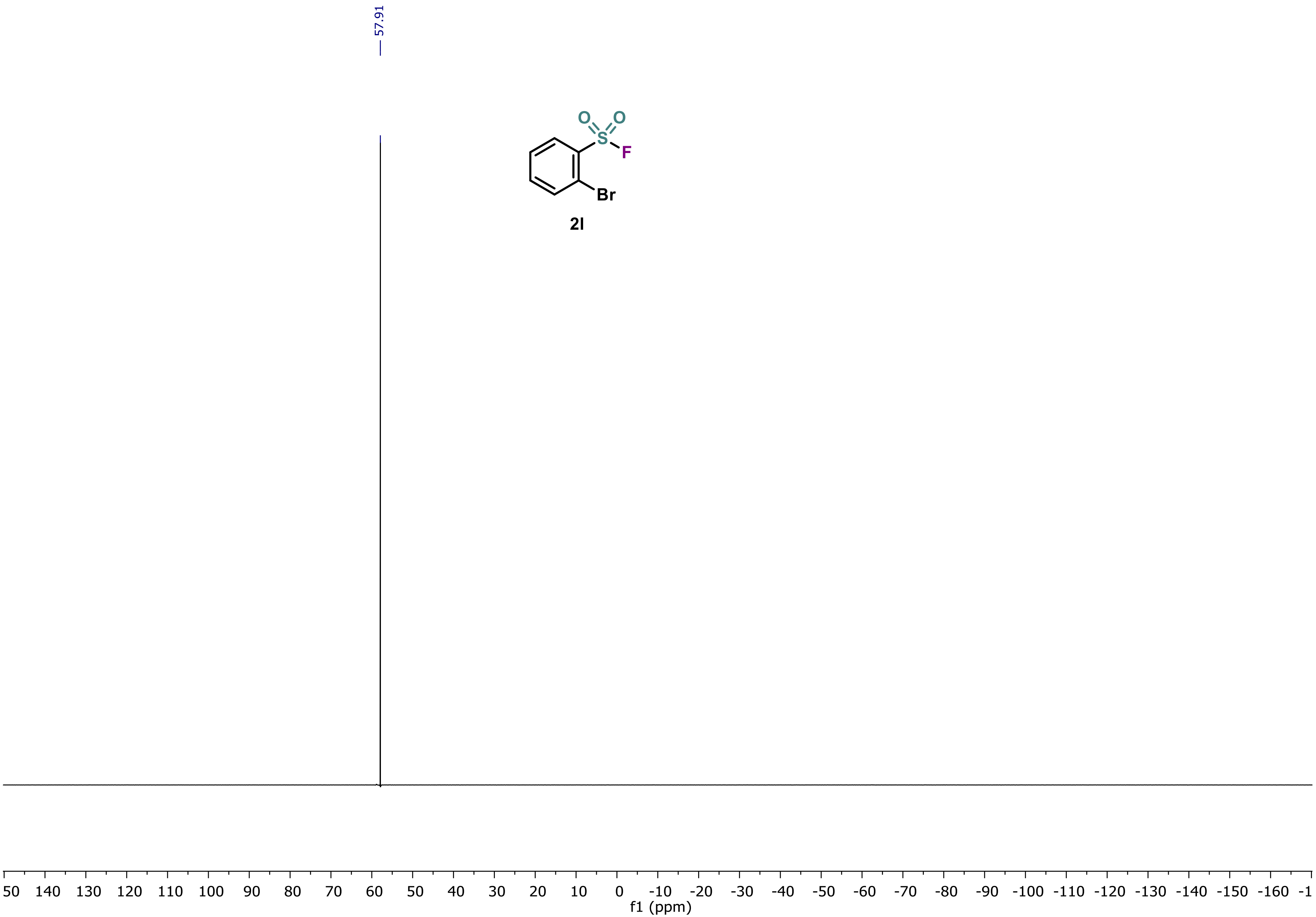

^{1}H NMR (600 MHz, $CDCl_3$)

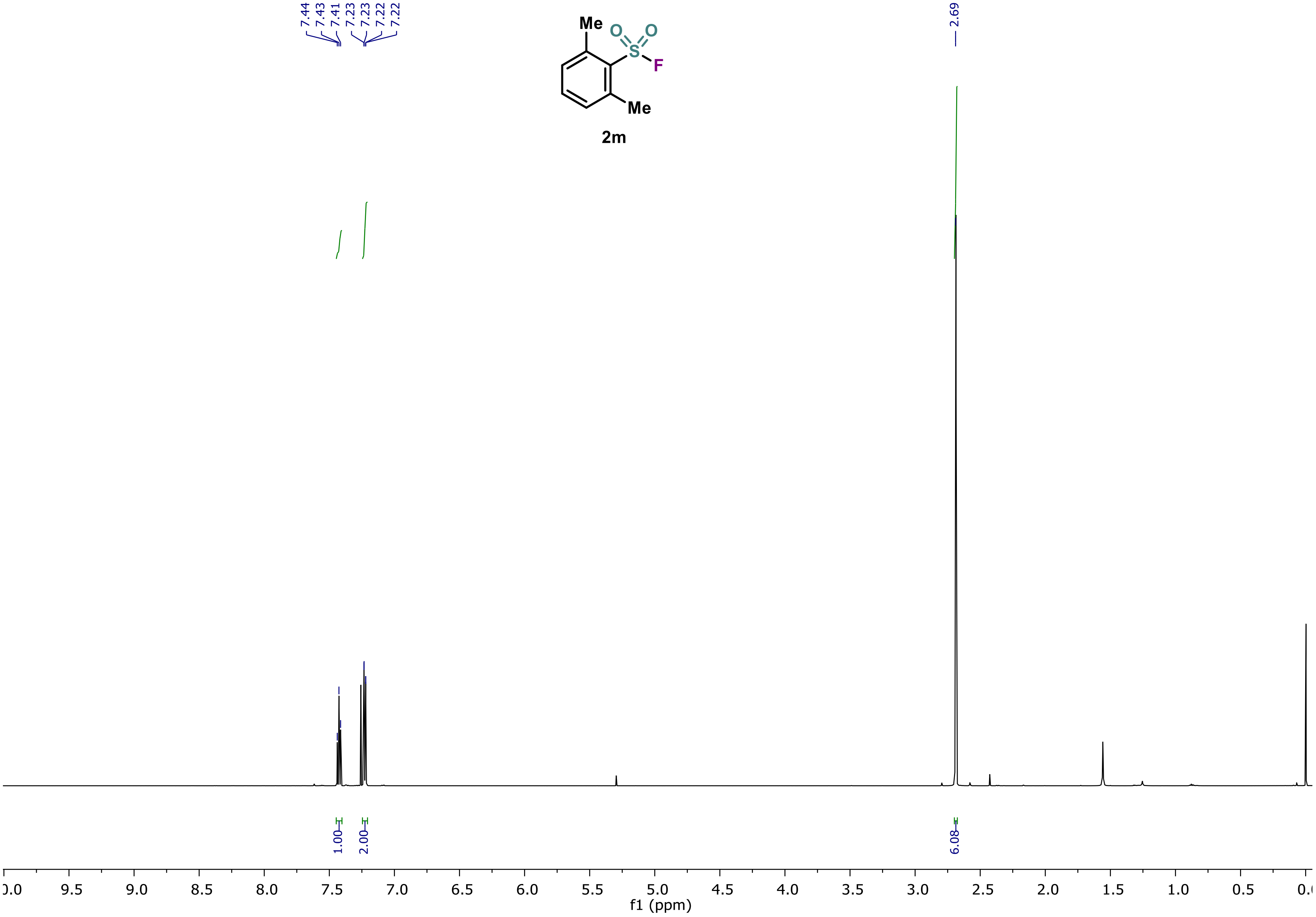

^{13}C NMR (151 MHz, $CDCl_3$)

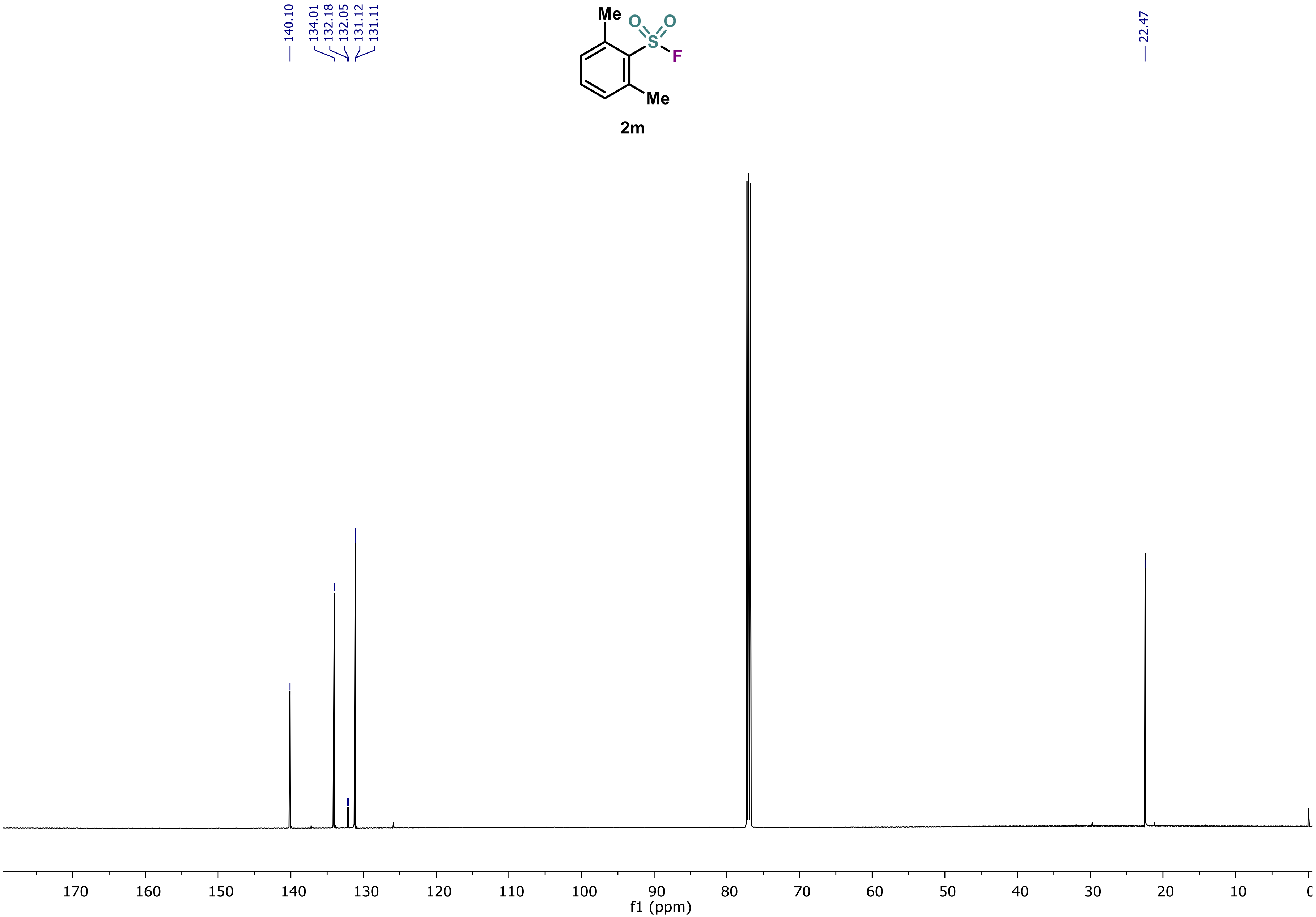

2m

^{19}F NMR (565 MHz, $CDCl_3$)

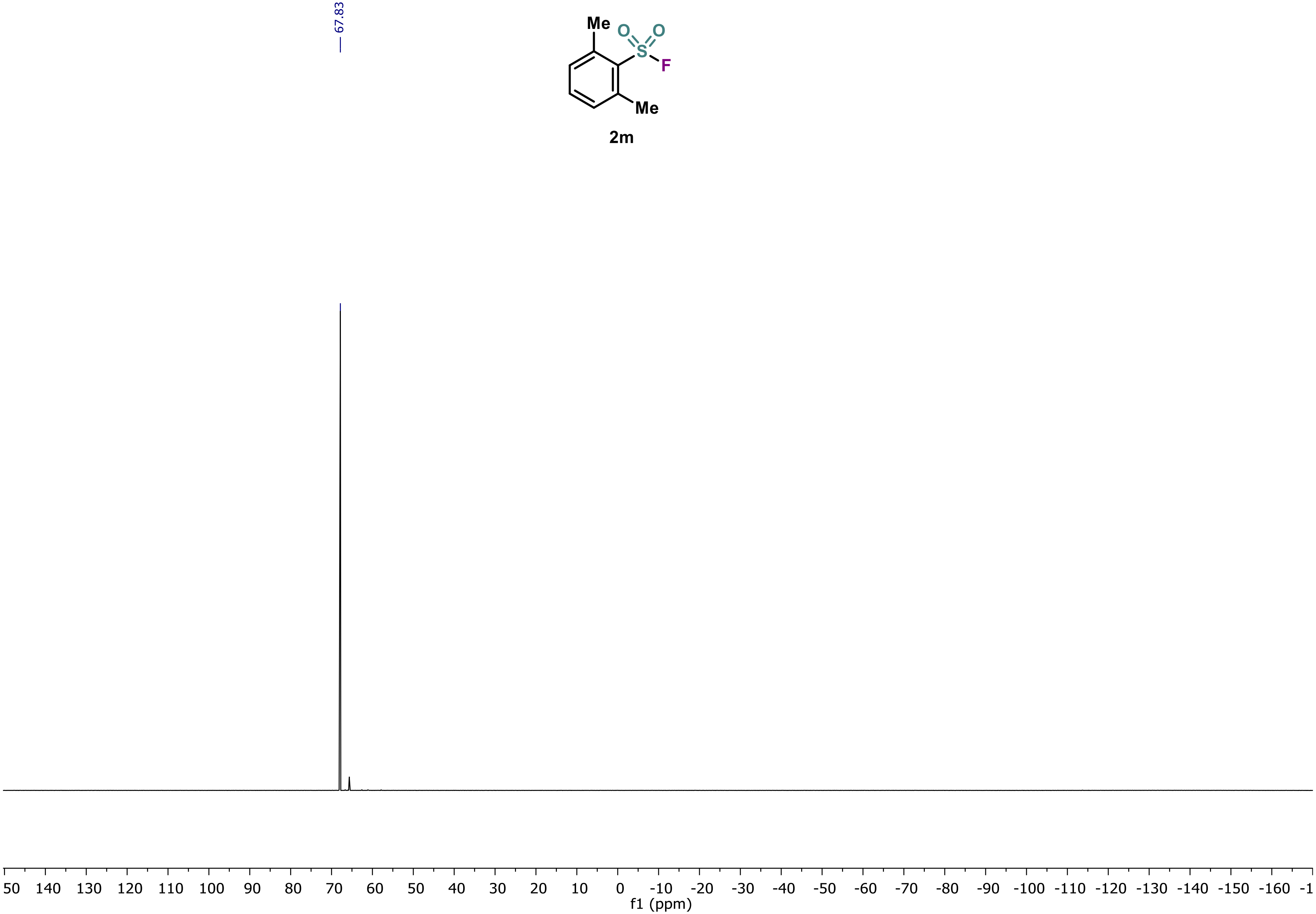

^{1}H NMR (600 MHz, $CDCl_3$)

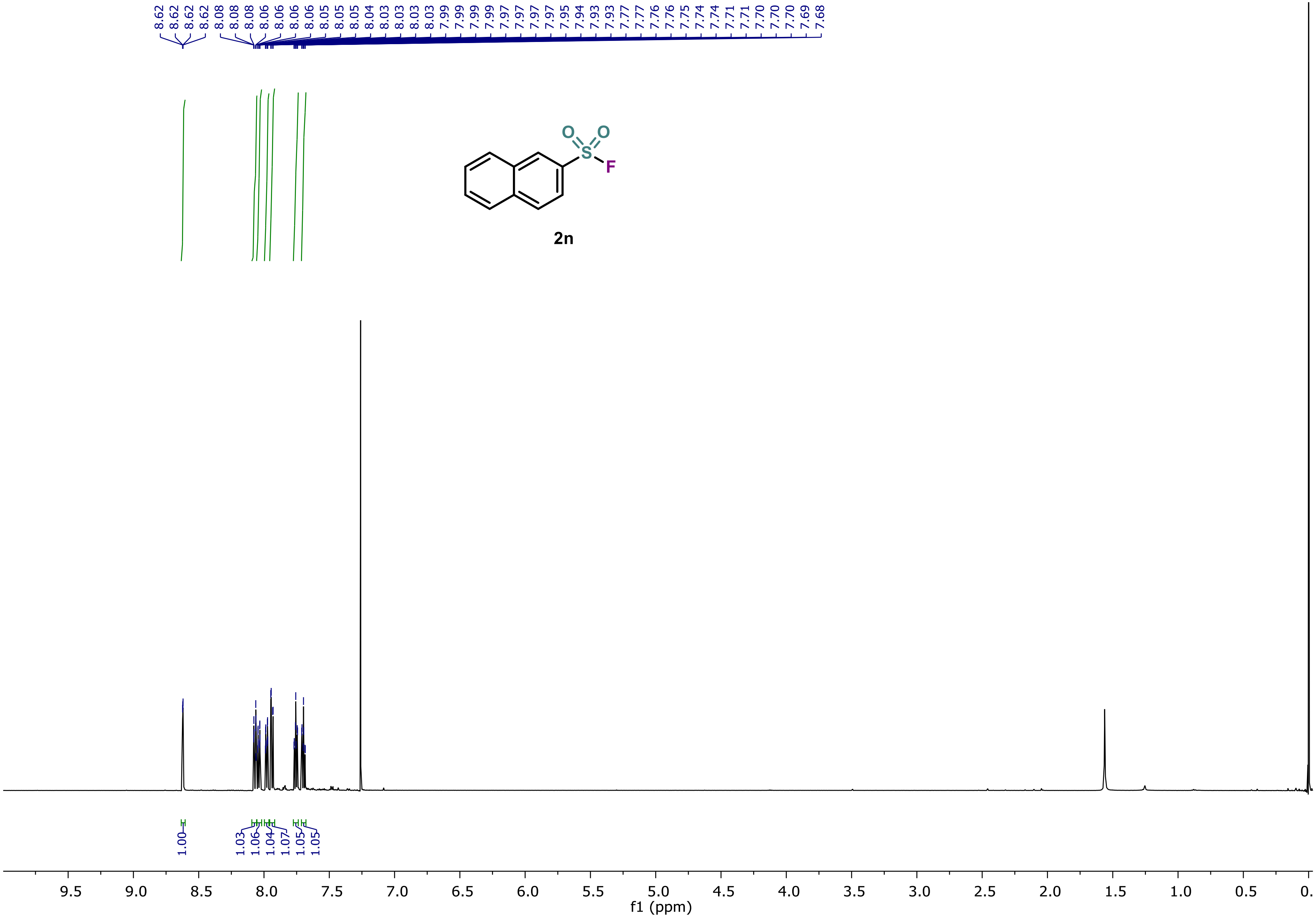

^{13}C NMR (151 MHz, $CDCl_3$)

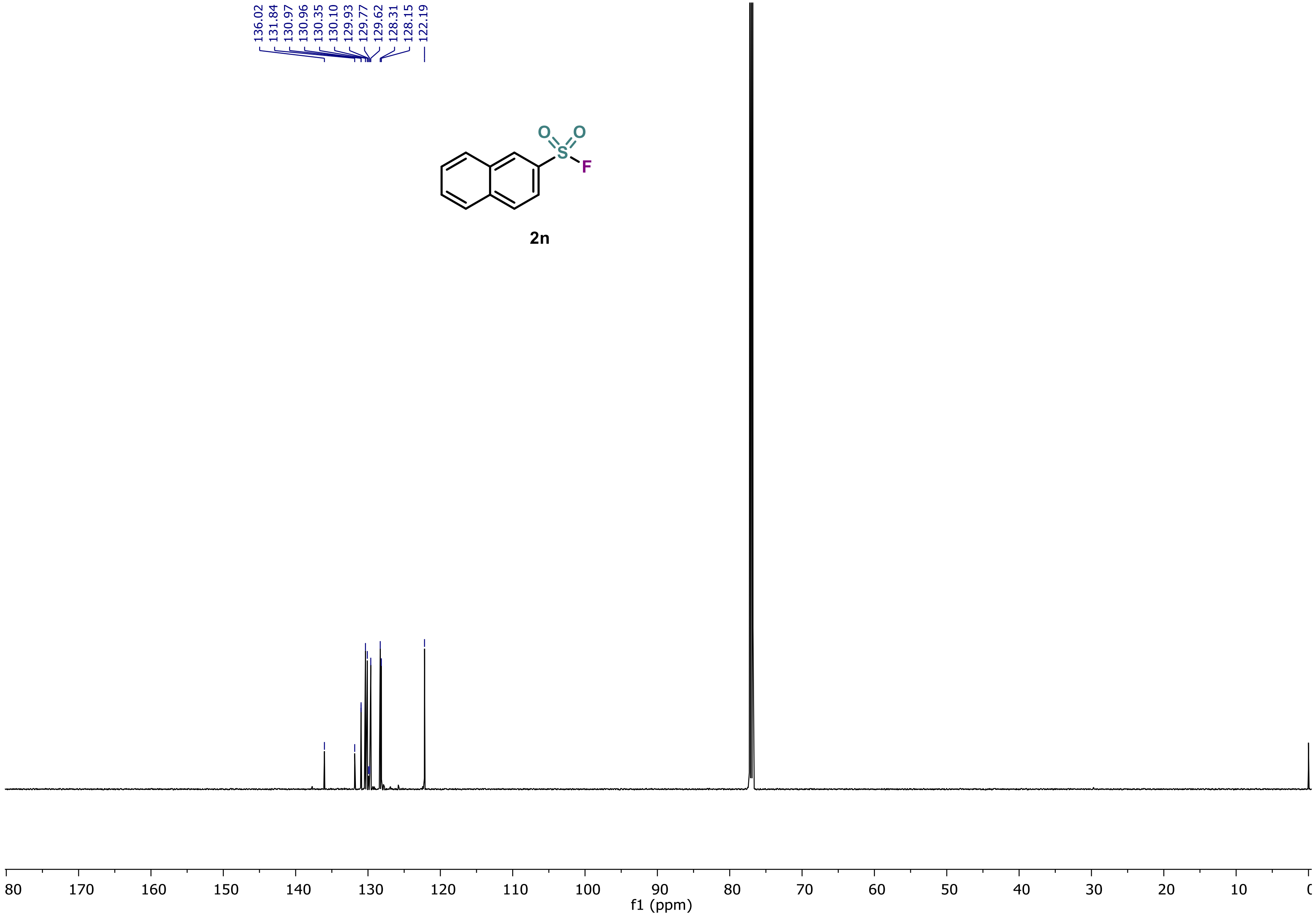

^{19}F NMR (565 MHz, $CDCl_3$)

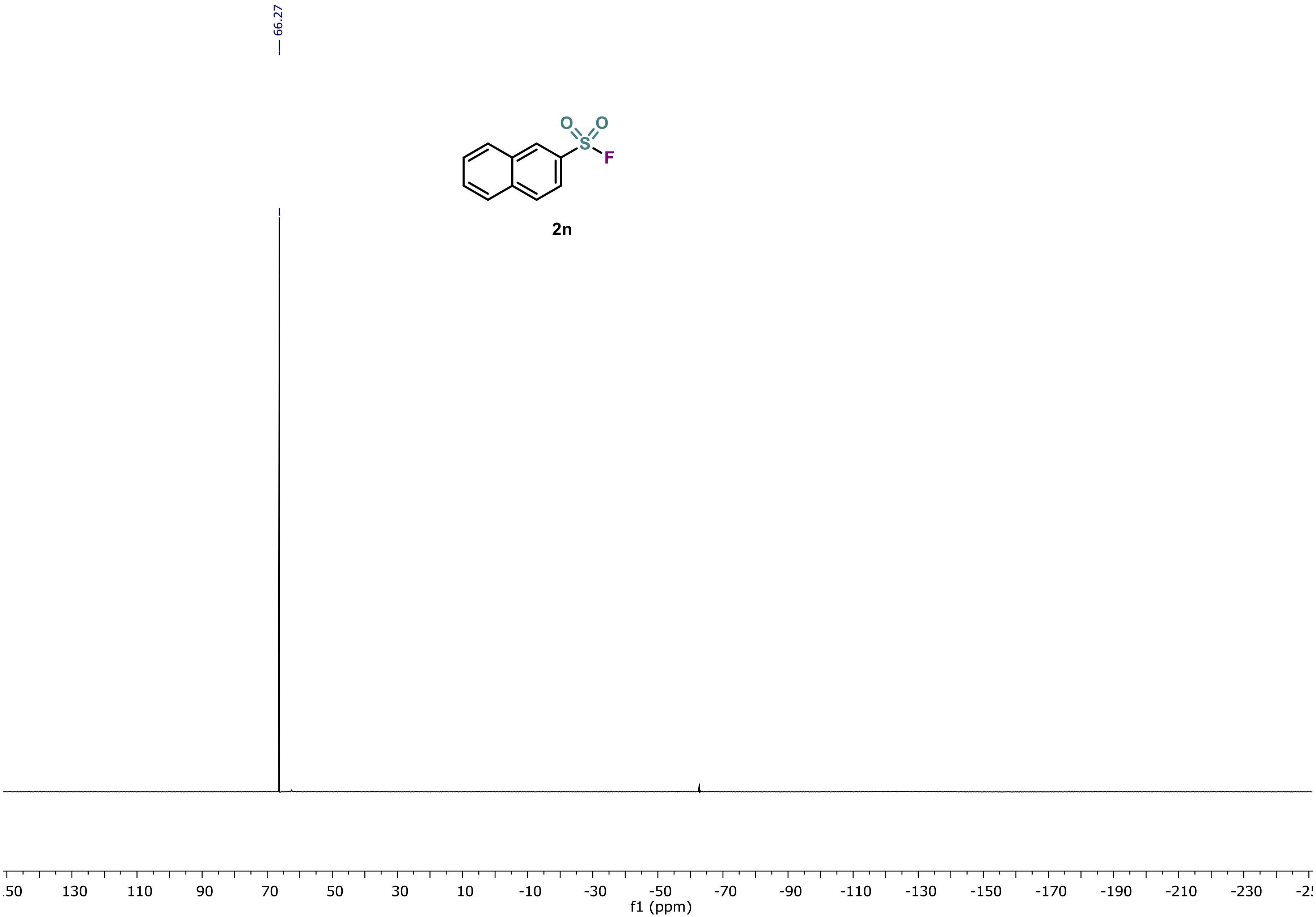

^{1}H NMR (600 MHz, $CDCl_3$)

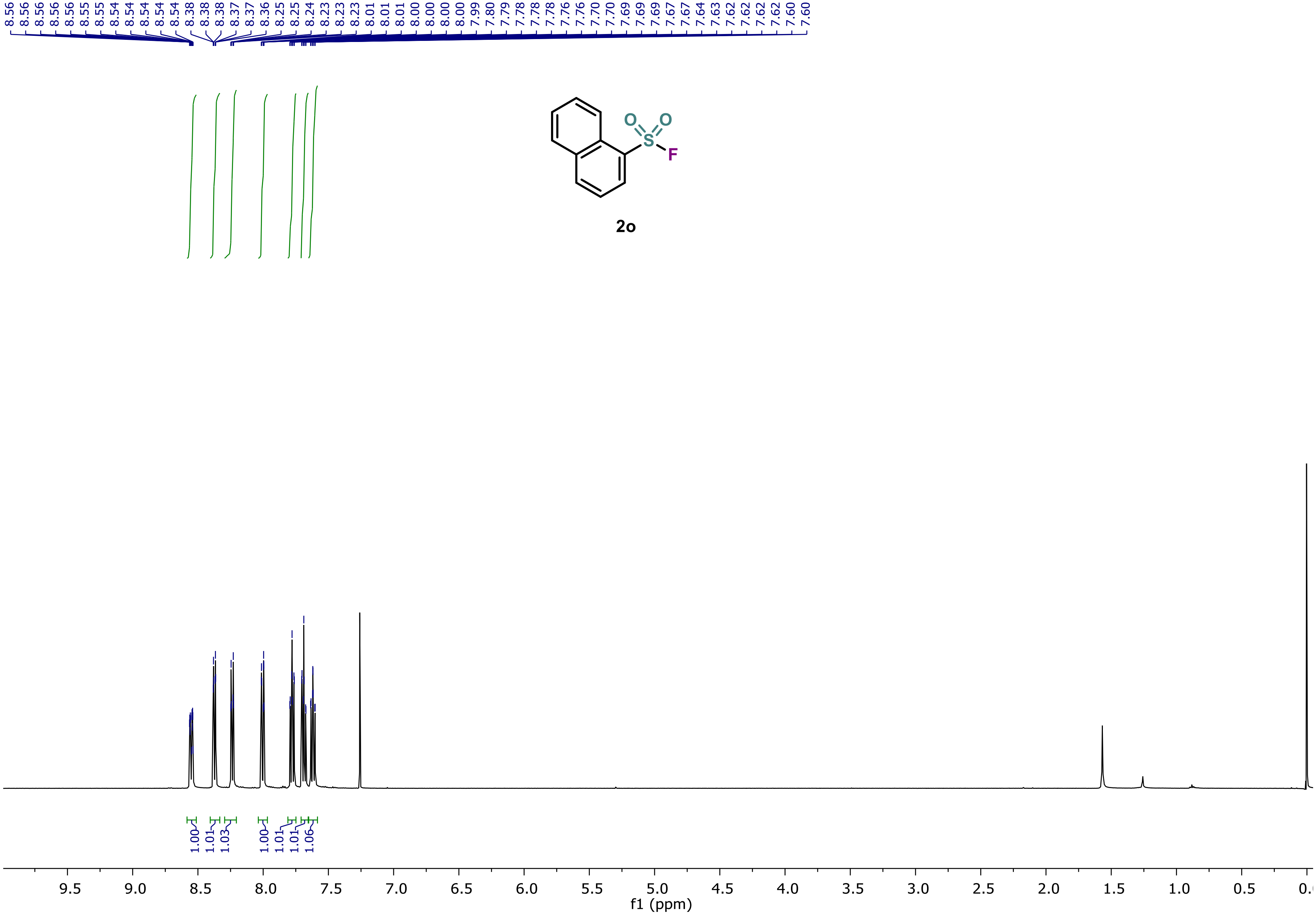

^{13}C NMR (151 MHz, $CDCl_3$)

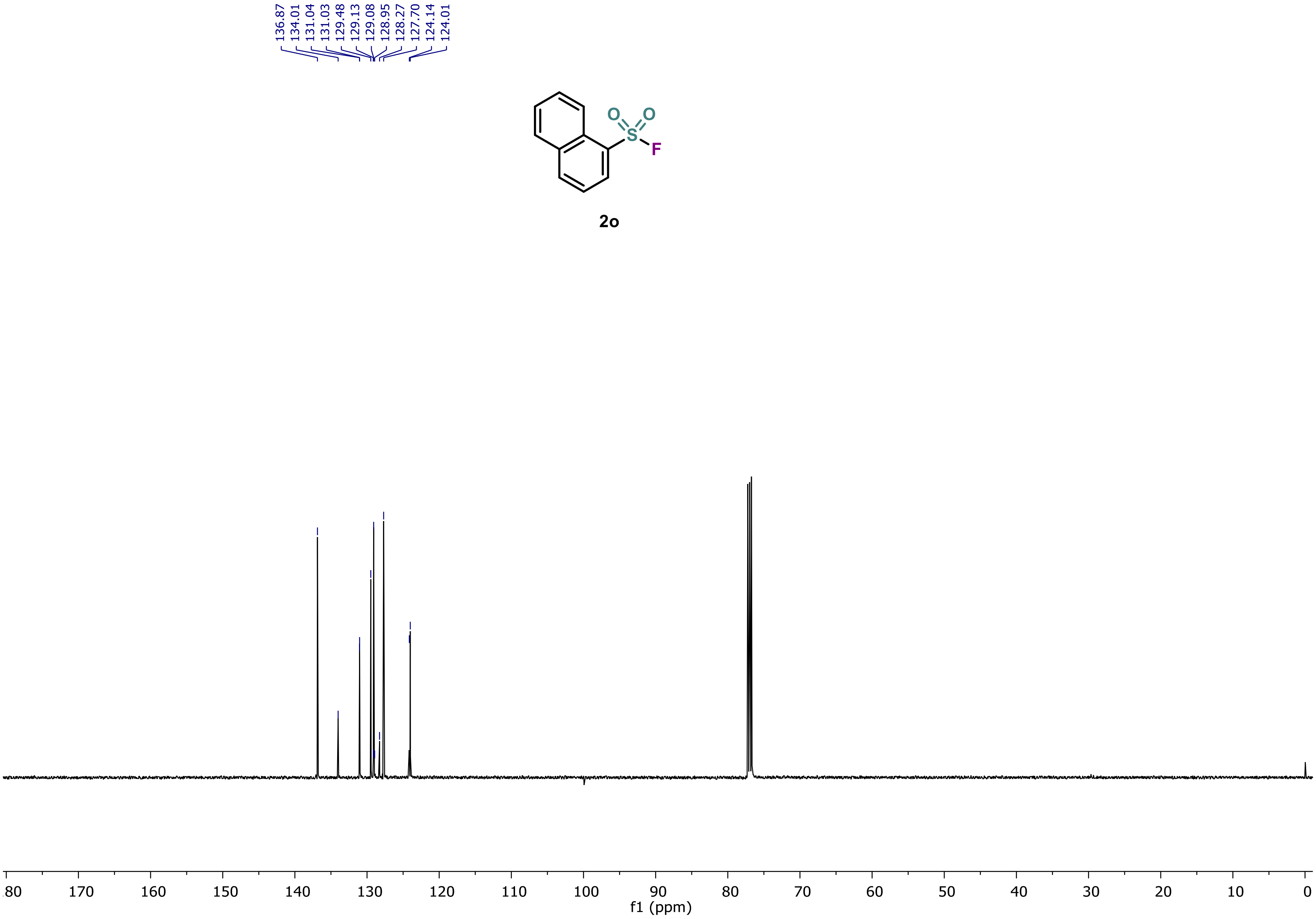

^{19}F NMR (565 MHz, $CDCl_3$)

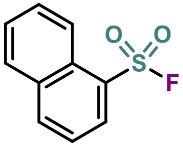

2o

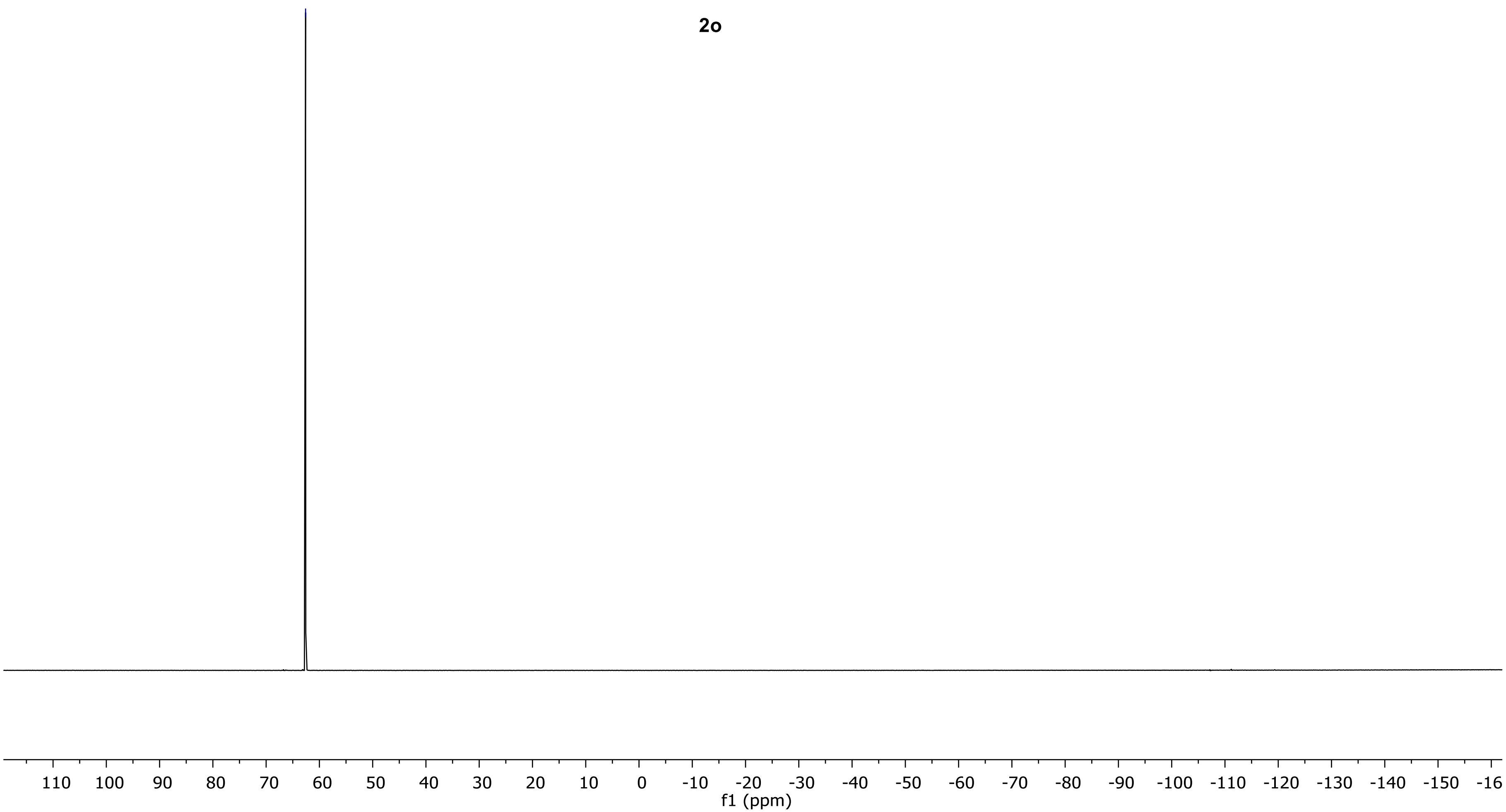

^1H NMR (600 MHz, $CDCl_3$)

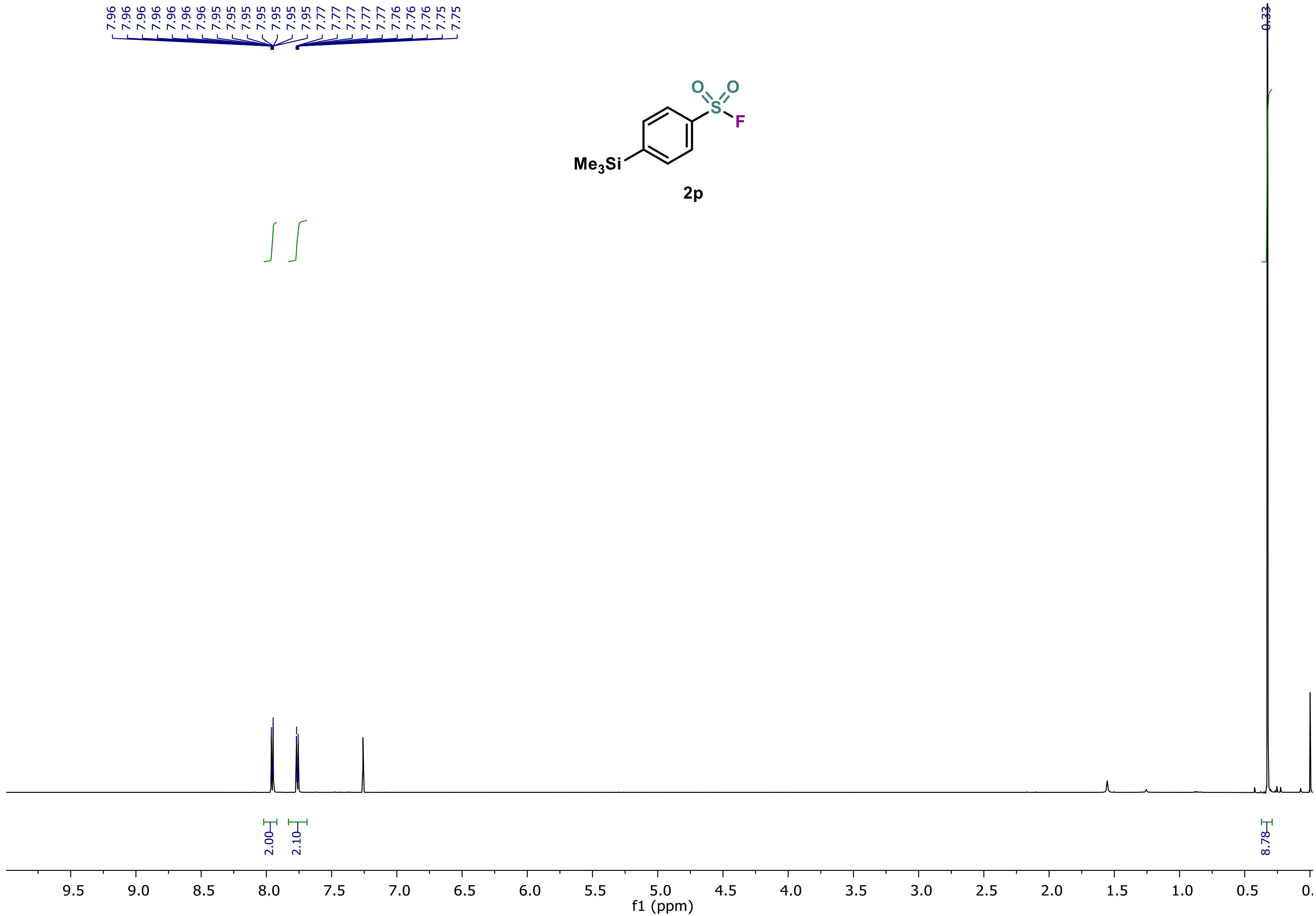

2p

^{13}C NMR (151 MHz, CDCl$_3$)

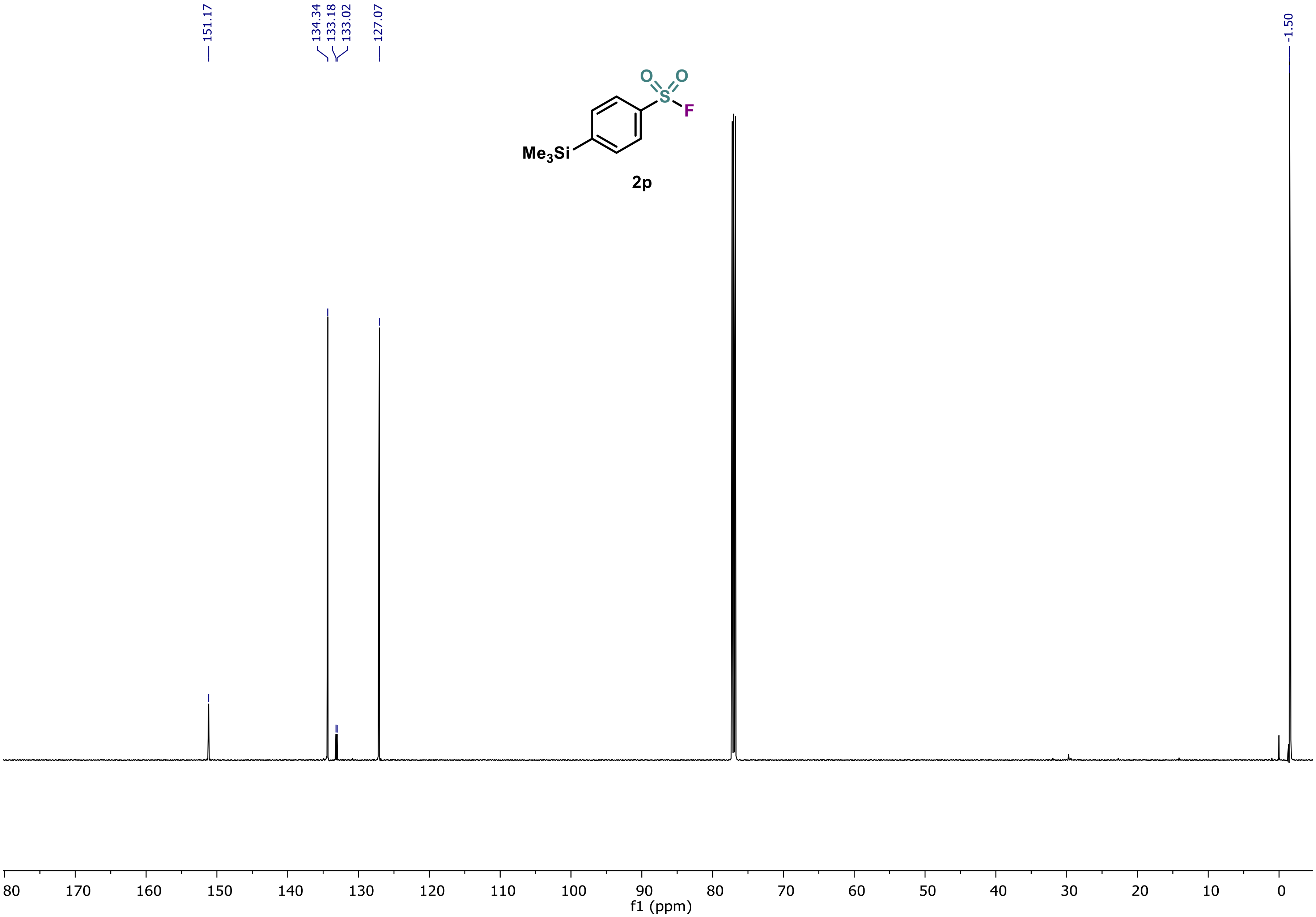

^{19}F NMR (565 MHz, $CDCl_3$)

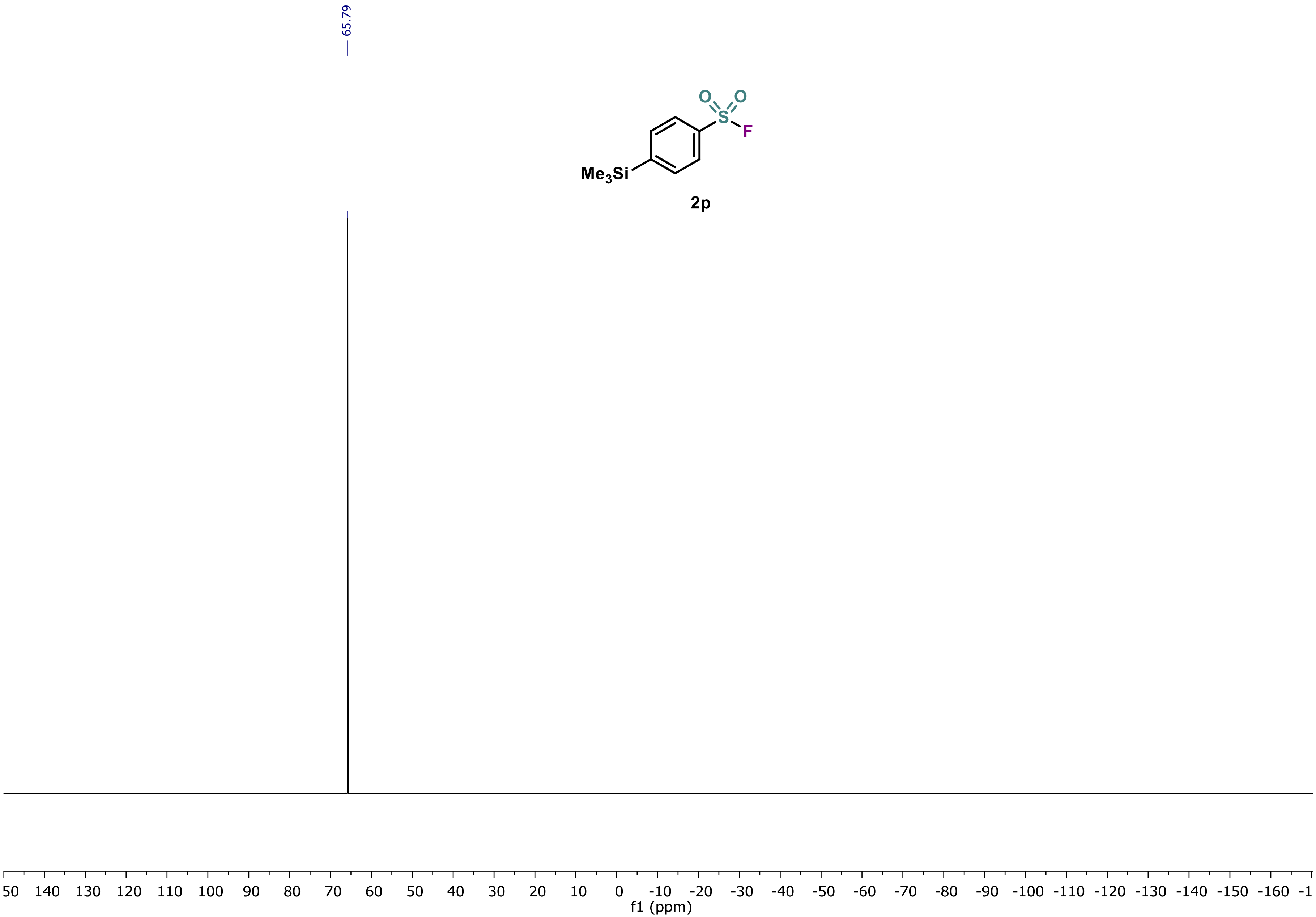

^{1}H NMR (600 MHz, $CDCl_3$)

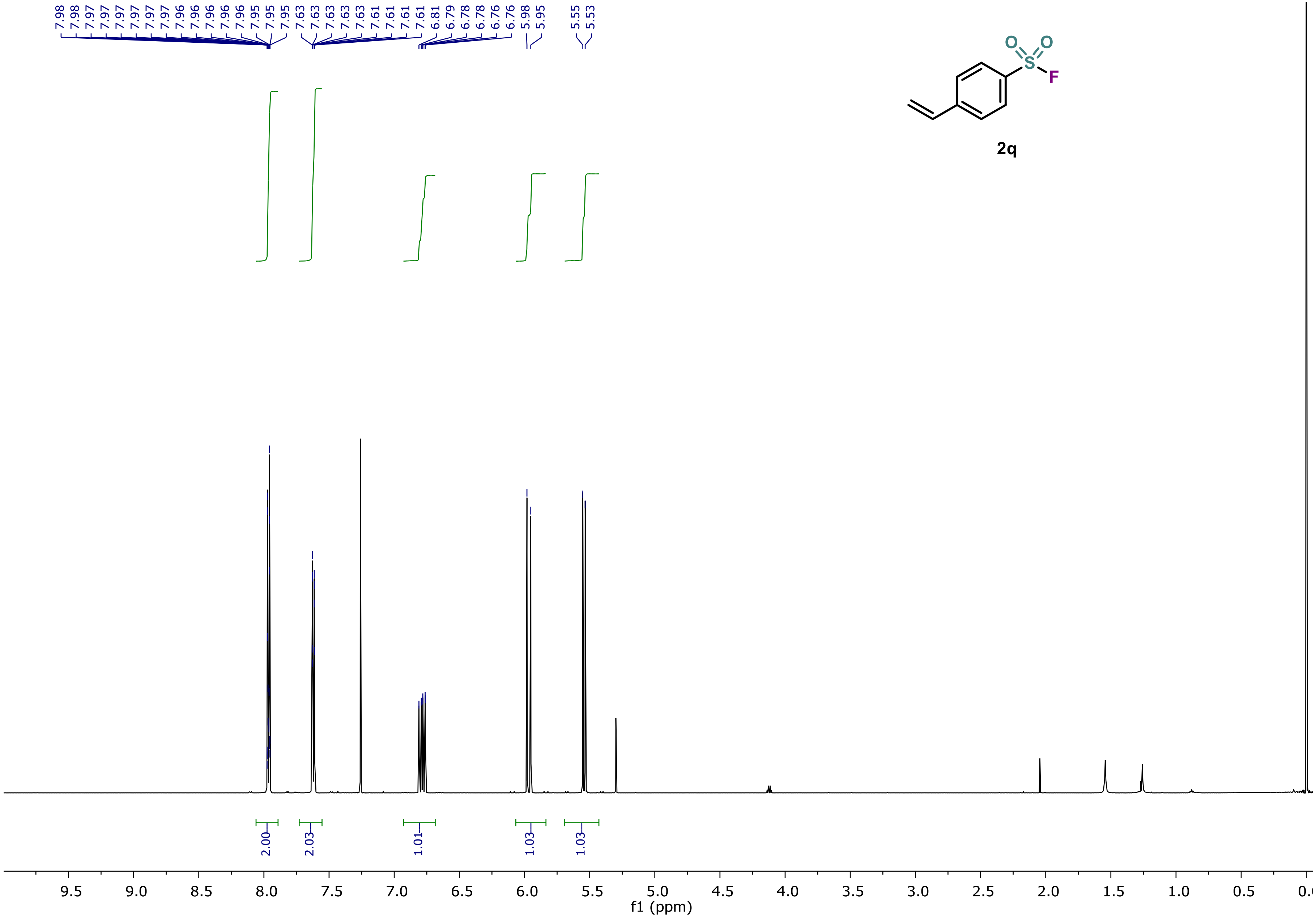

^{13}C NMR (151 MHz, CDCl$_3$)

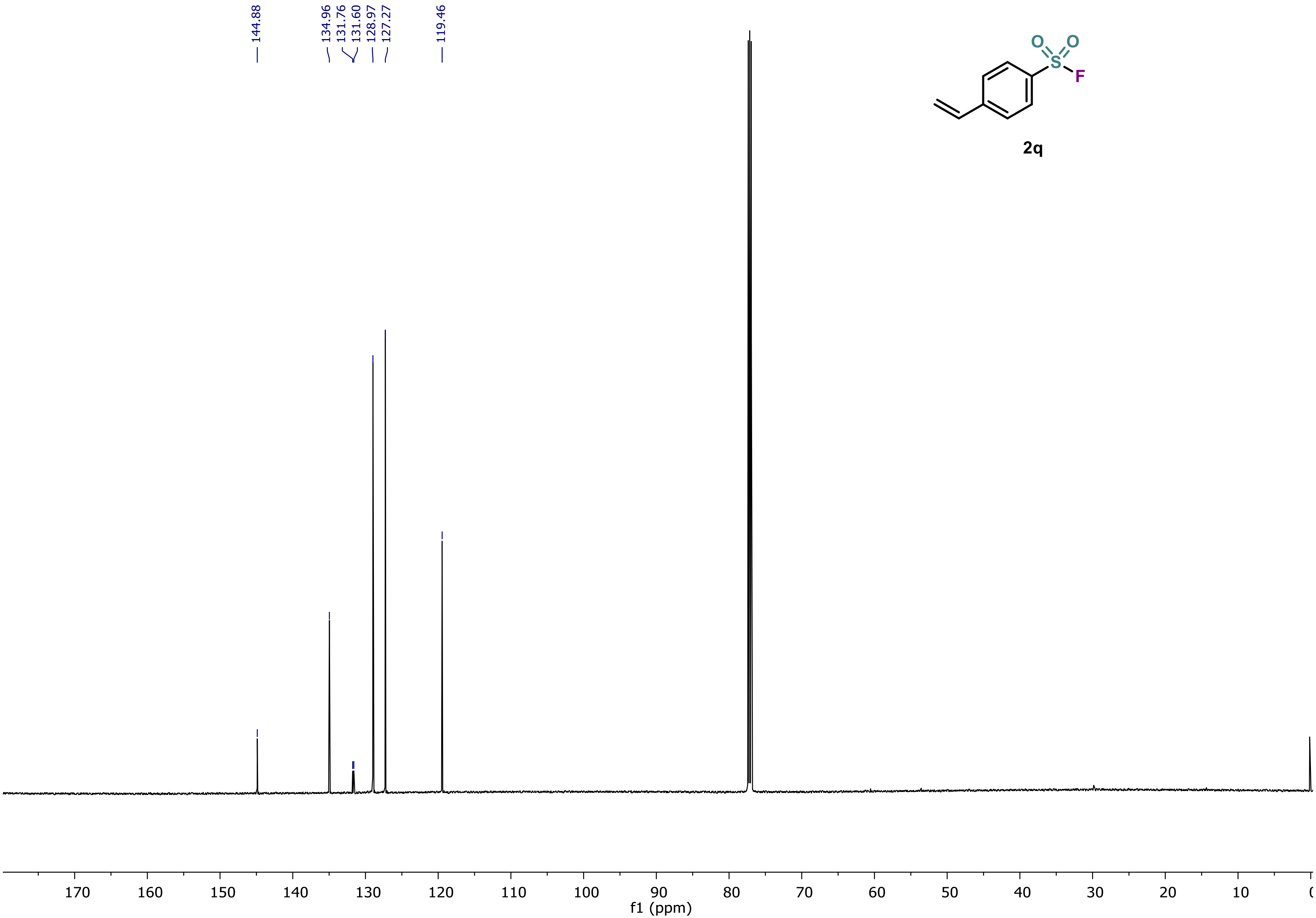

2q

^{19}F NMR (565 MHz, $CDCl_3$)

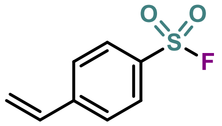

2q

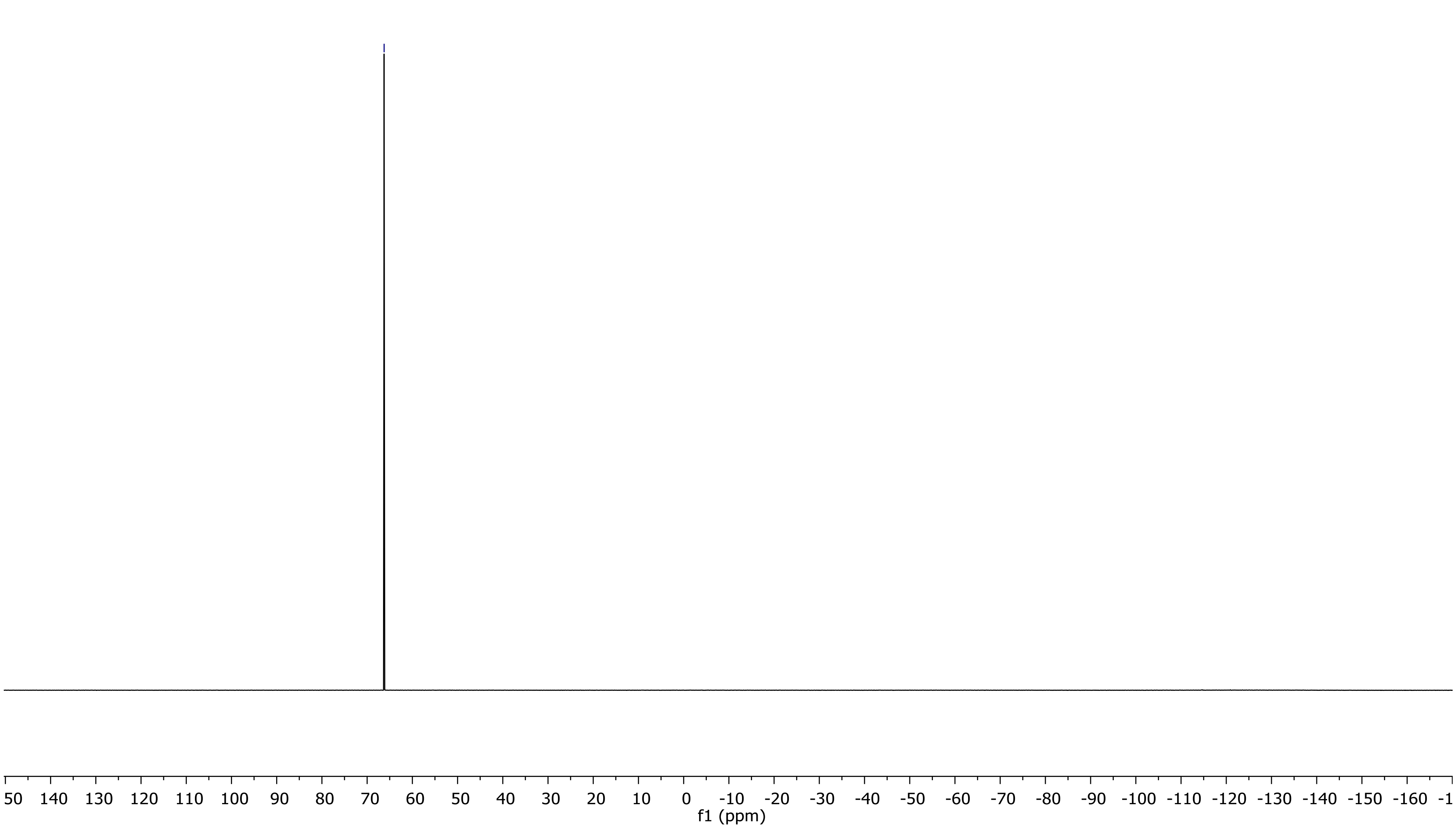

^{1}H NMR (600 MHz, $CDCl_3$)

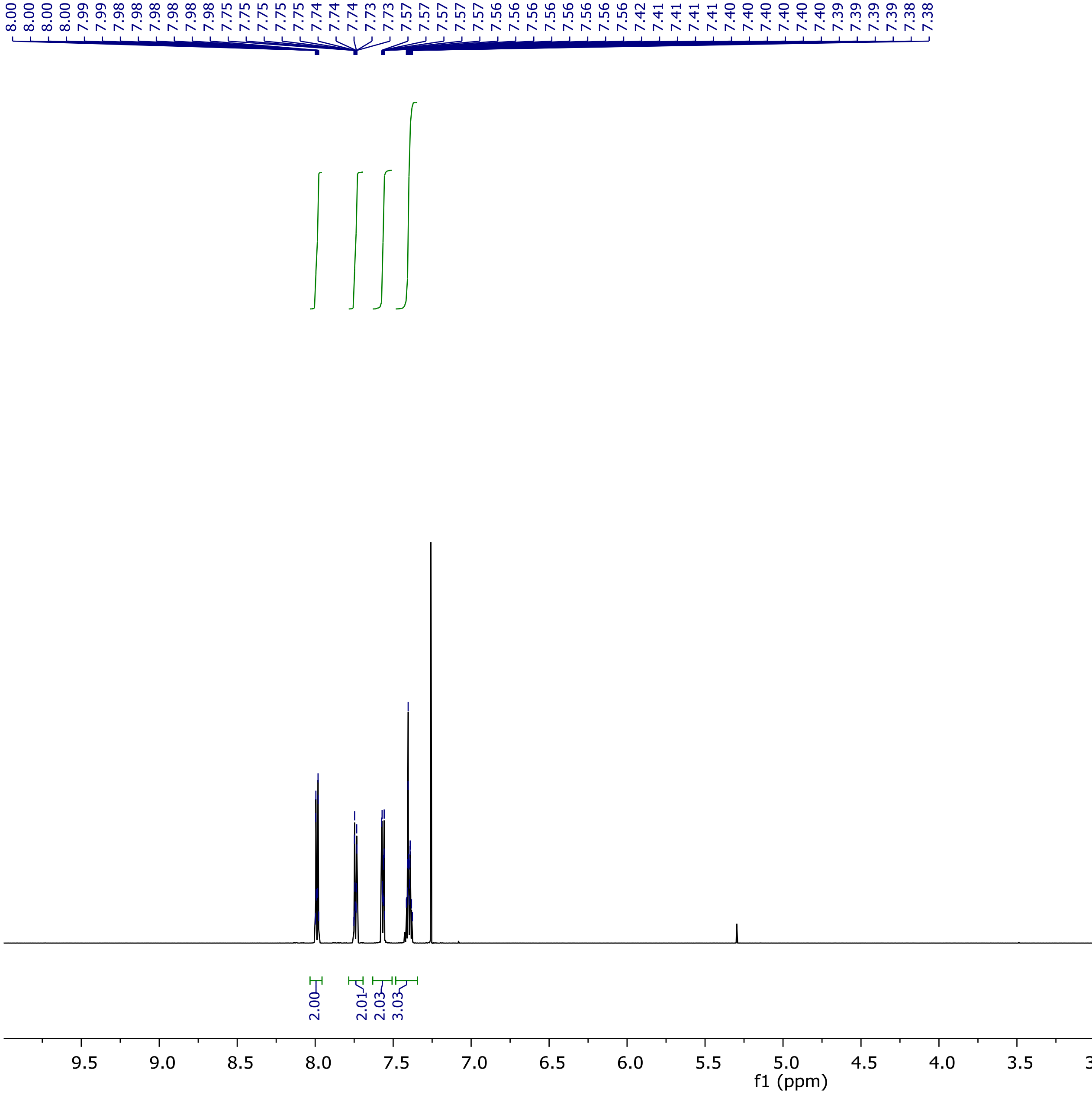

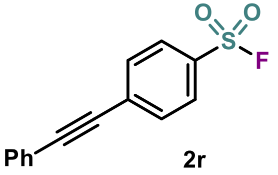

2r

^{13}C NMR (151 MHz, $CDCl_3$)

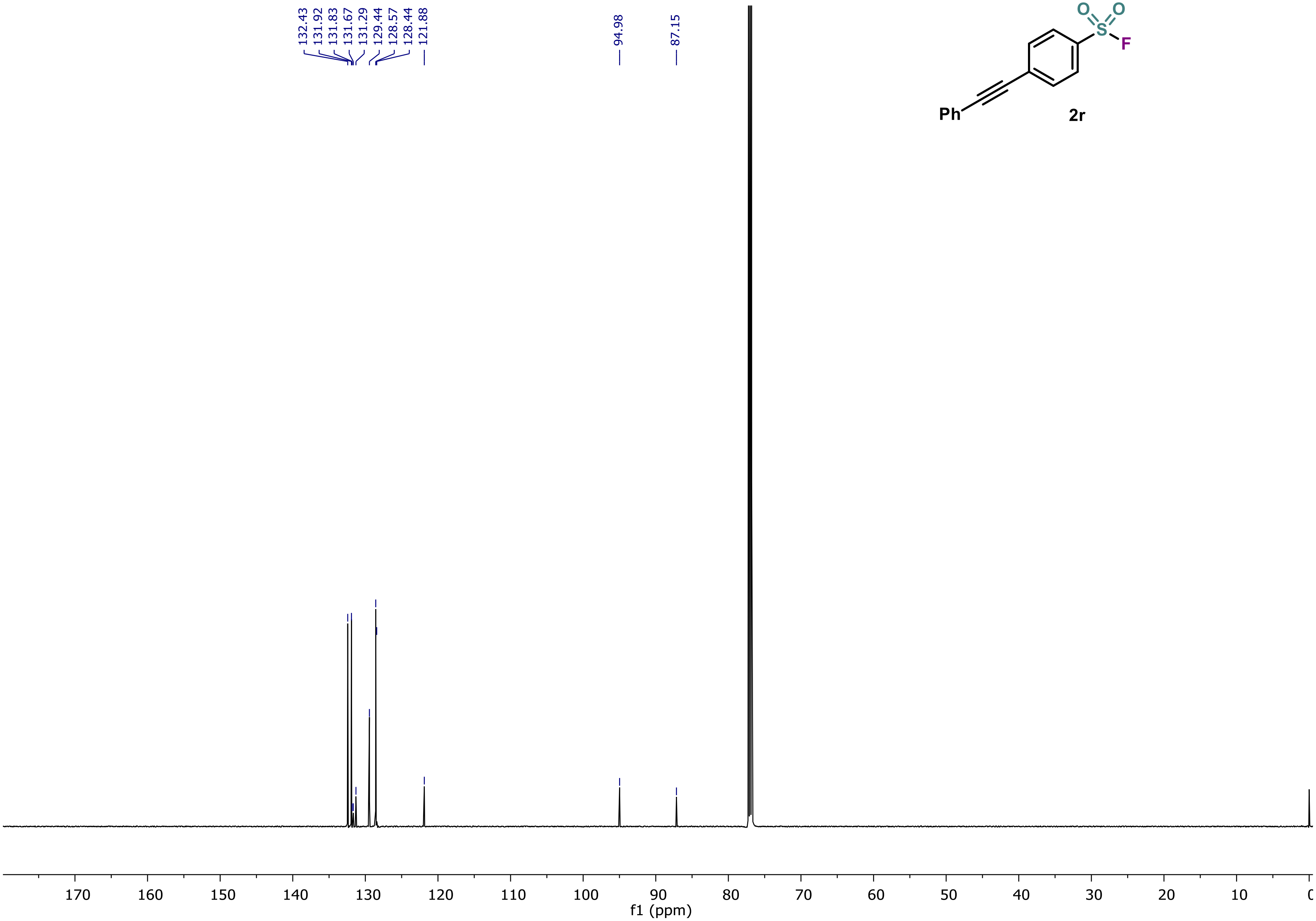

2r

^{19}F NMR (565 MHz, $CDCl_3$)

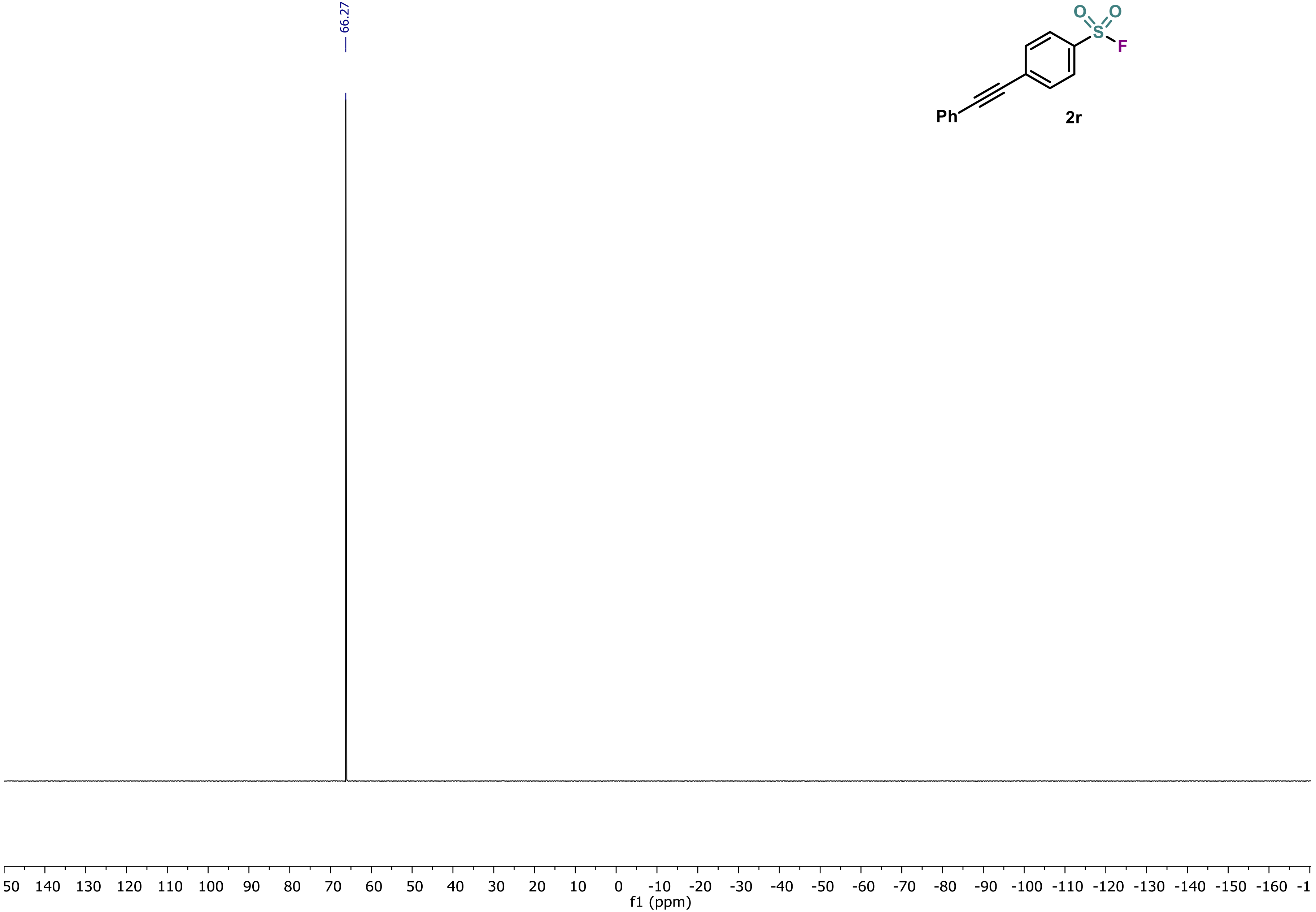

2r

^{1}H NMR (600 MHz, $CDCl_3$)

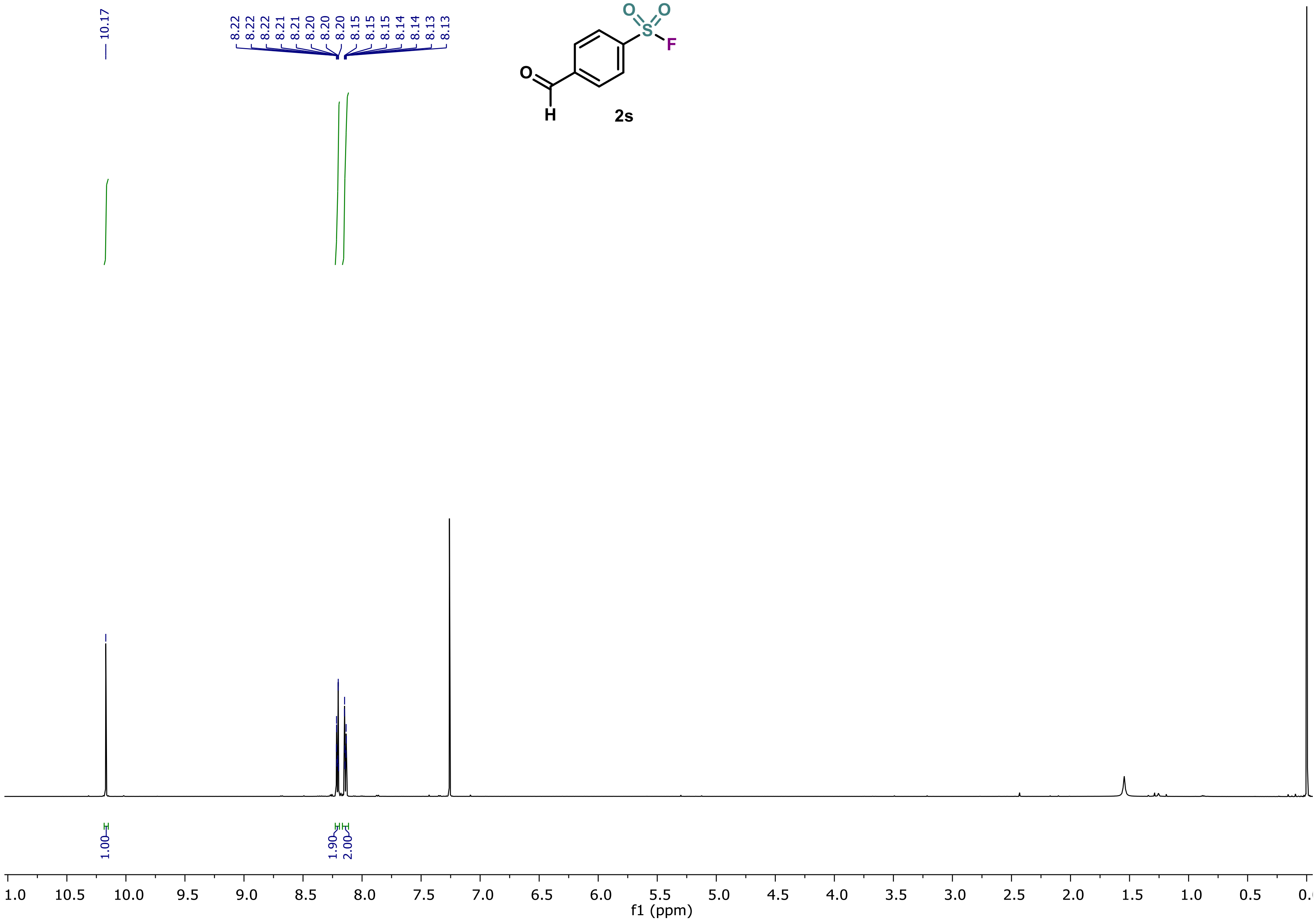

2s

^{13}C NMR (151 MHz, $CDCl_3$)

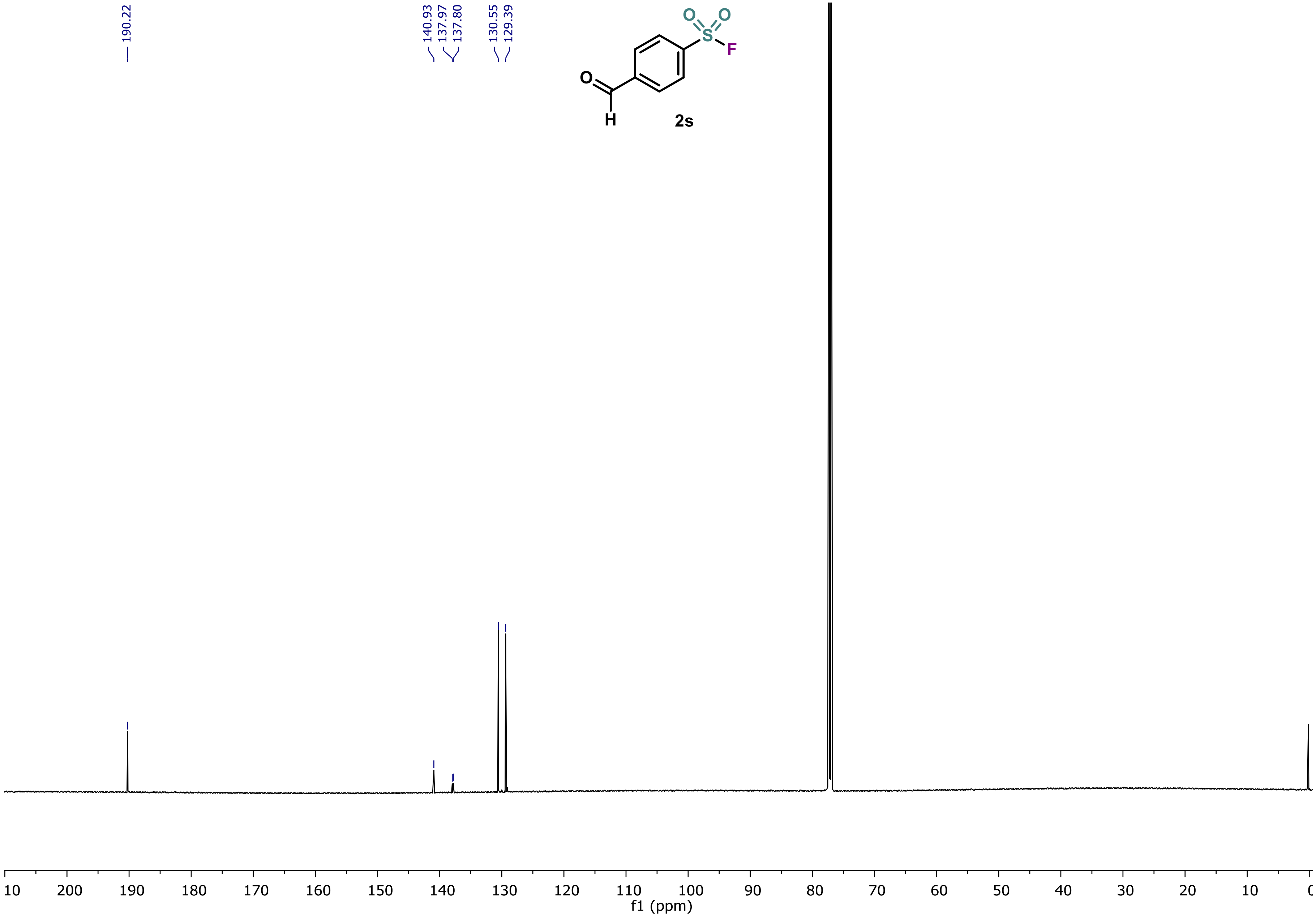

^{19}F NMR (565 MHz, $CDCl_3$)

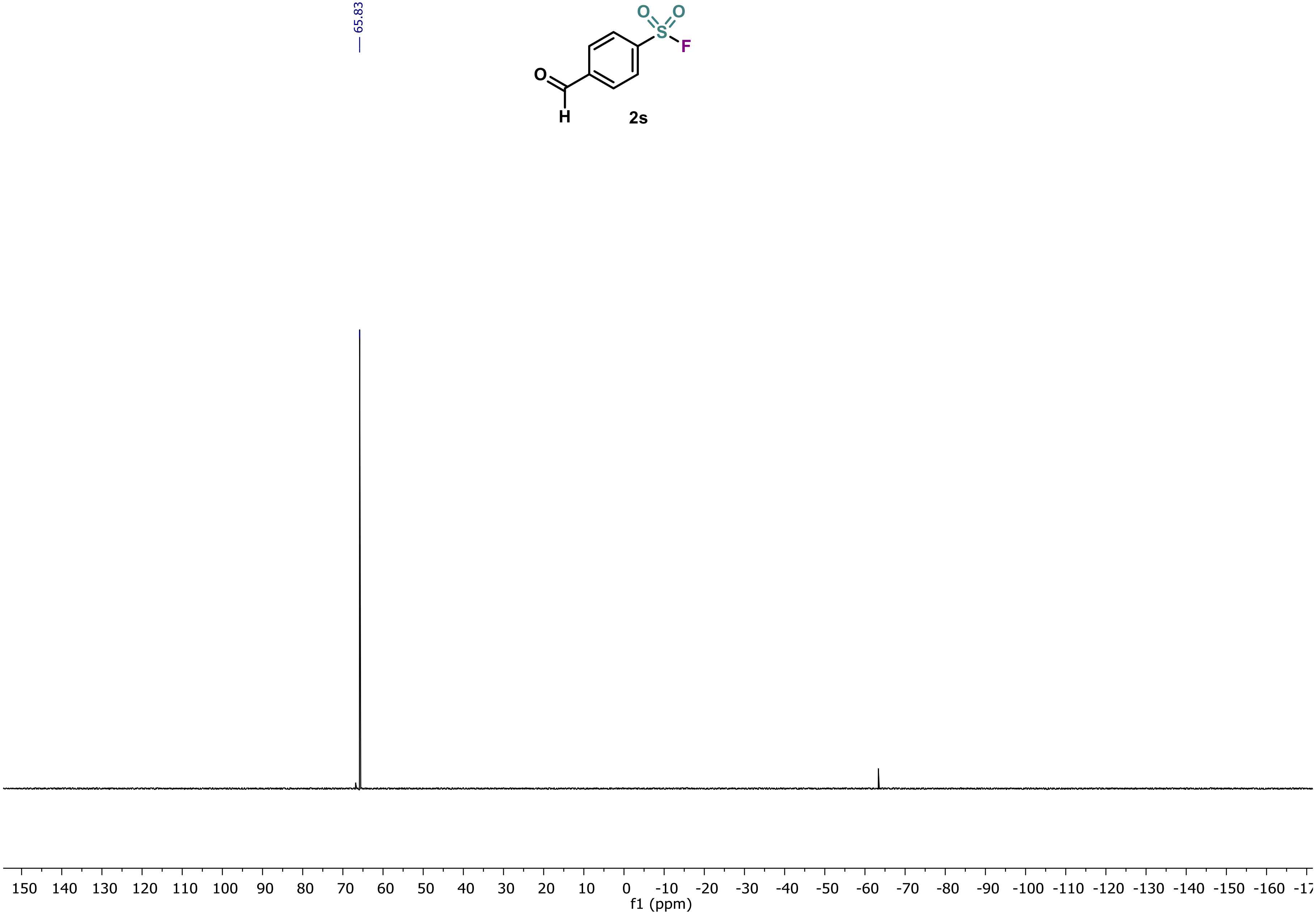

^{1}H NMR (600 MHz, $CDCl_3$)

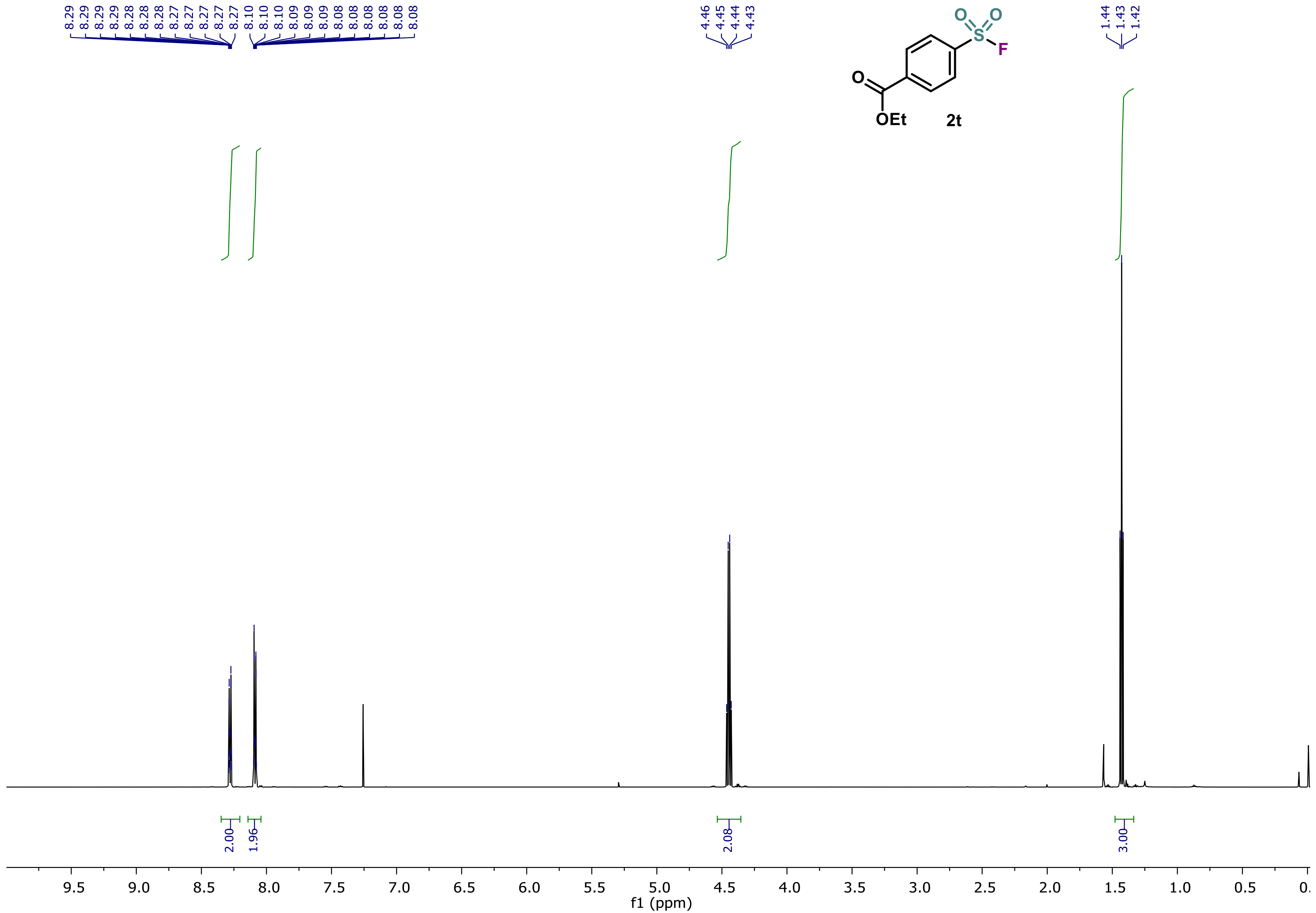

^{13}C NMR (151 MHz, $CDCl_3$)

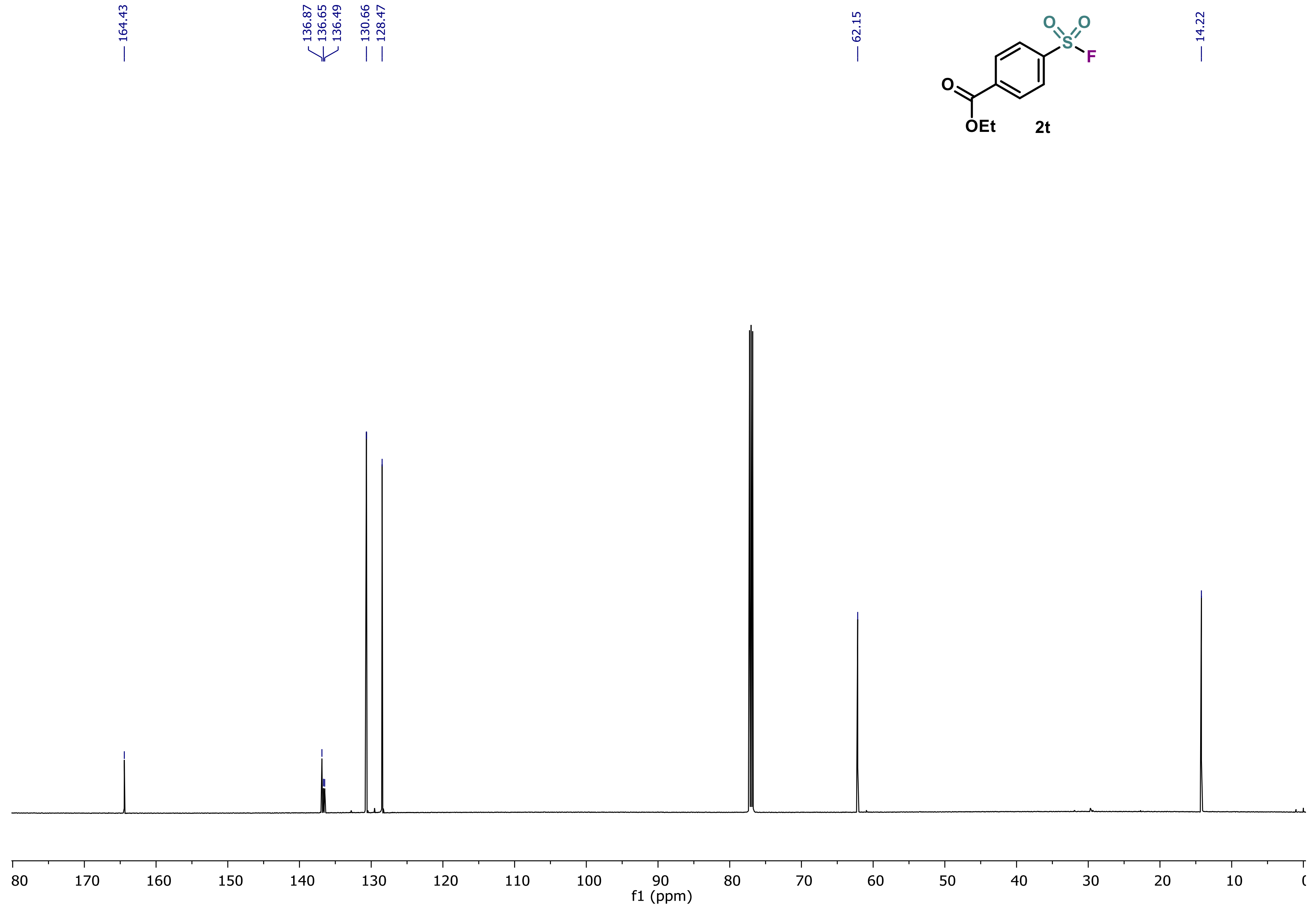

^{19}F NMR (565 MHz, $CDCl_3$)

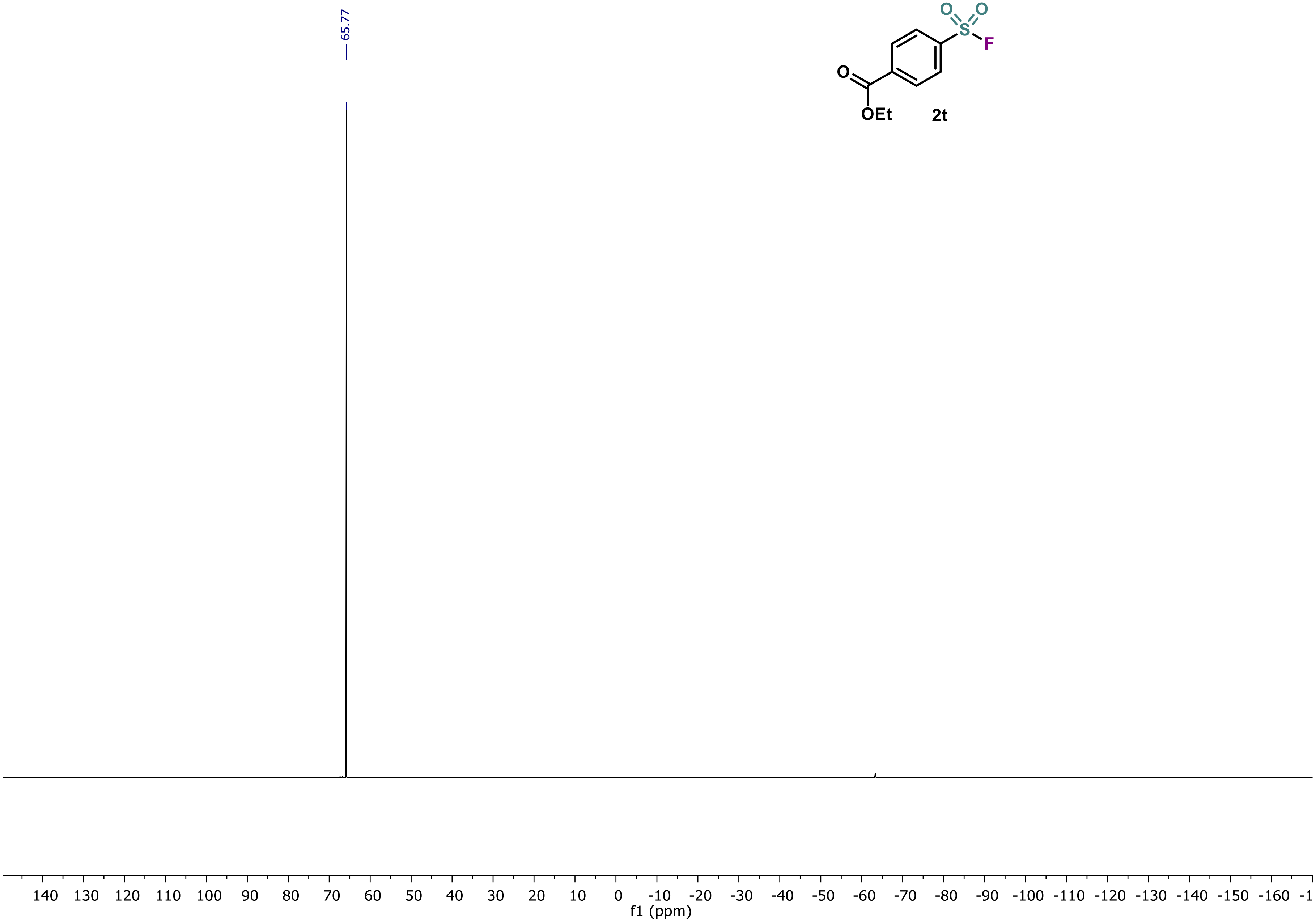

^{1}H NMR (600 MHz, $CDCl_3$)

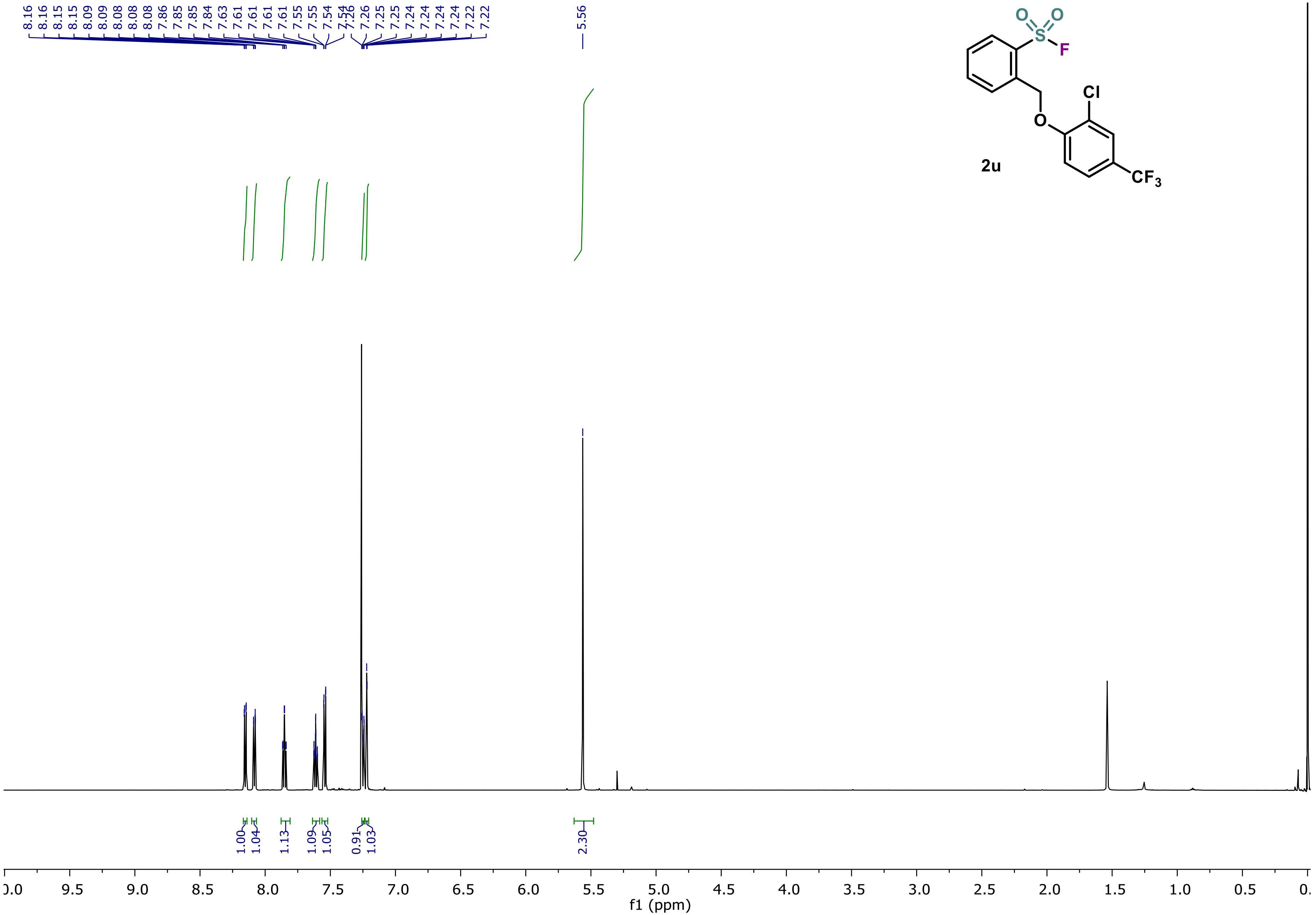

^{13}C NMR (151 MHz, CDCl$_3$)

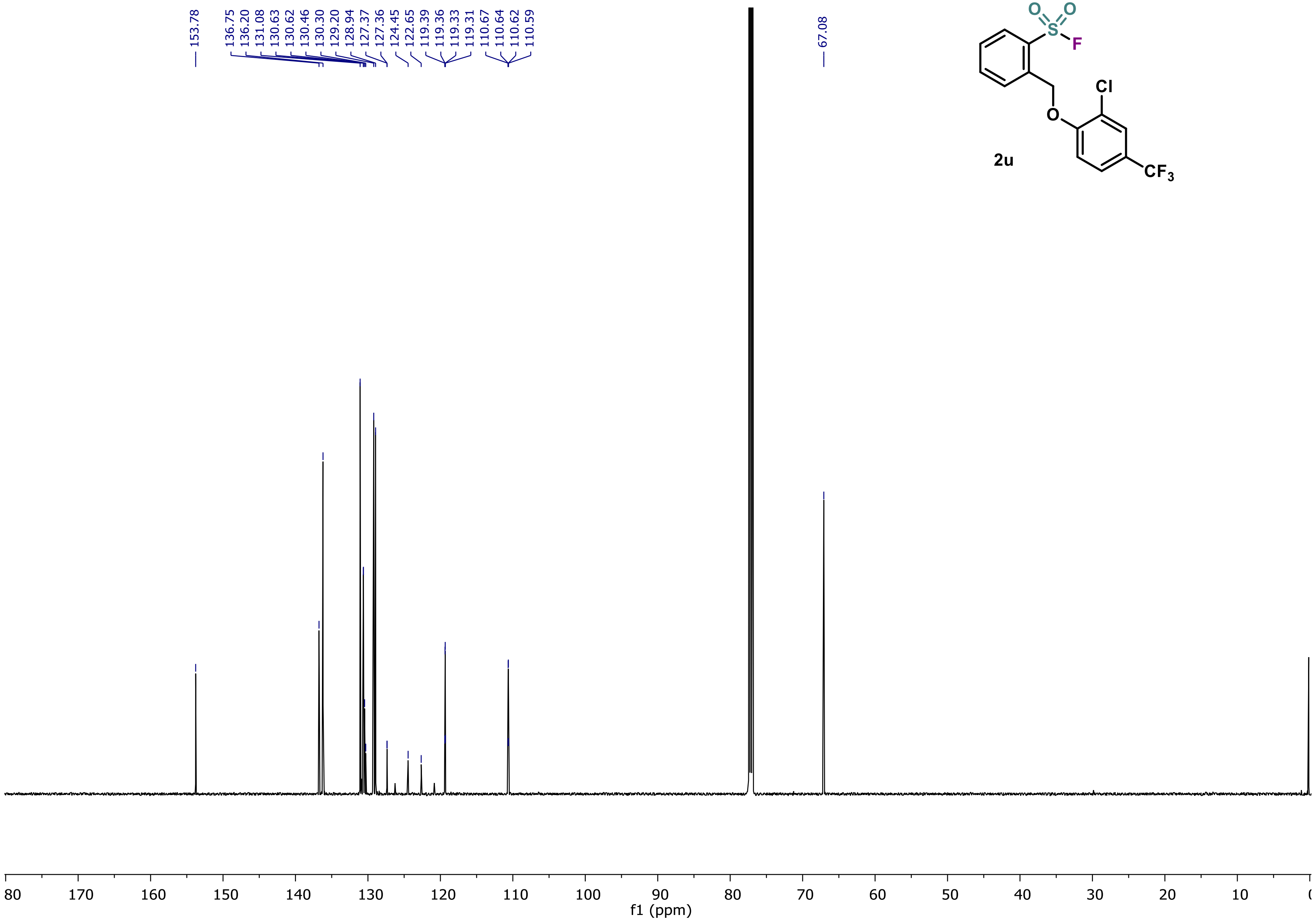

^{19}F NMR (565 MHz, $CDCl_3$)

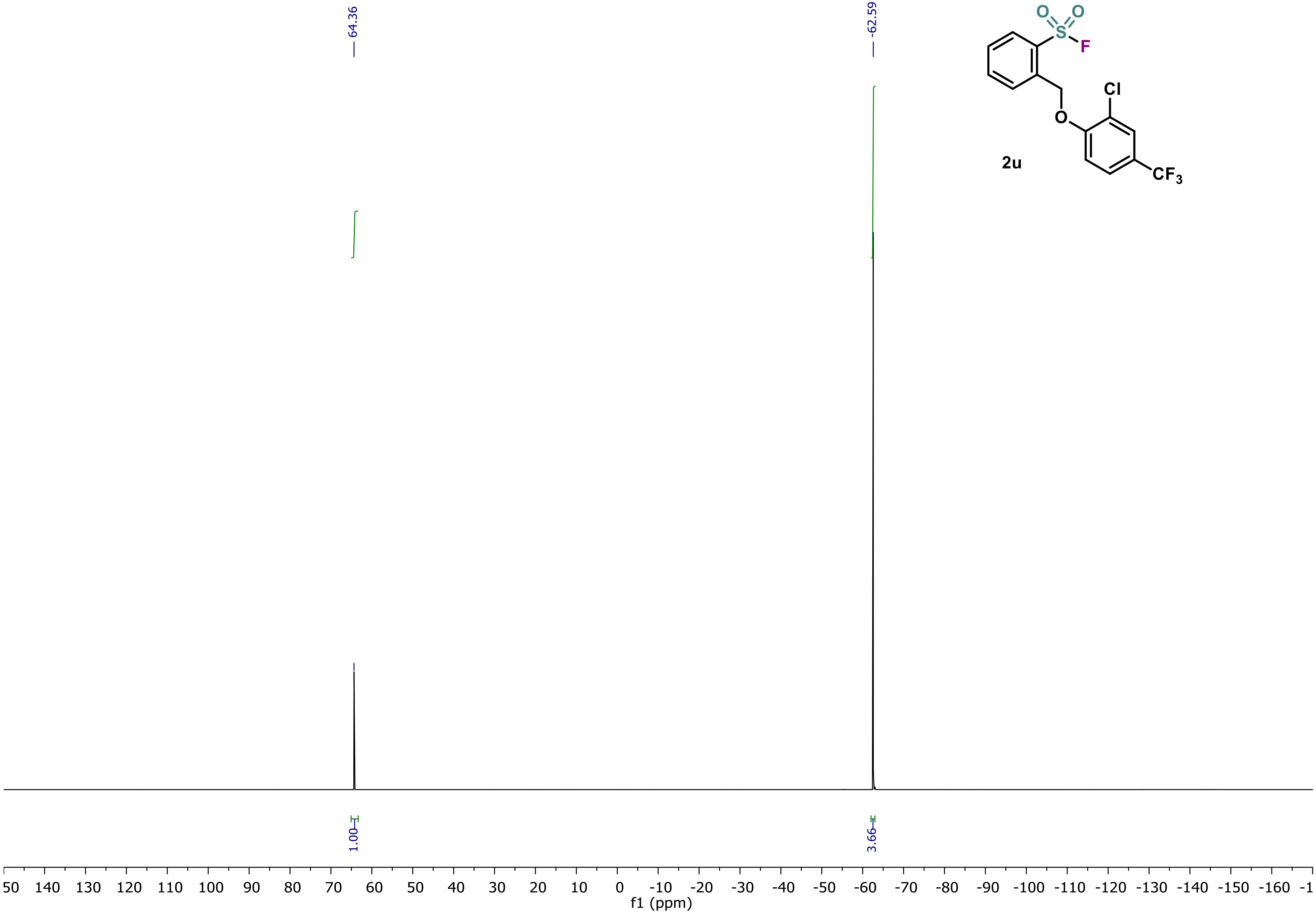

^{1}H NMR (600 MHz, $CDCl_3$)

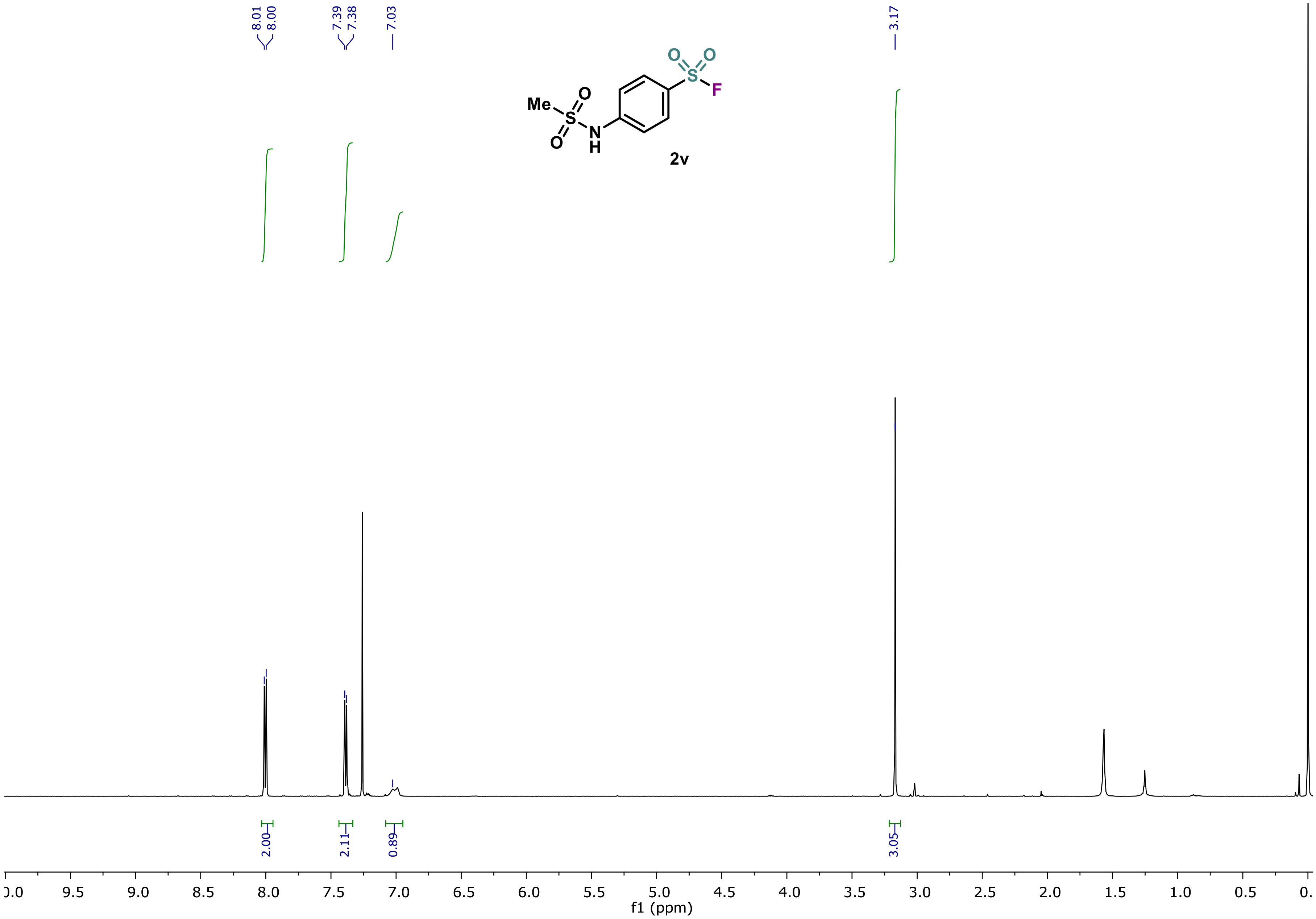

^{13}C NMR (151 MHz, $CDCl_3$)

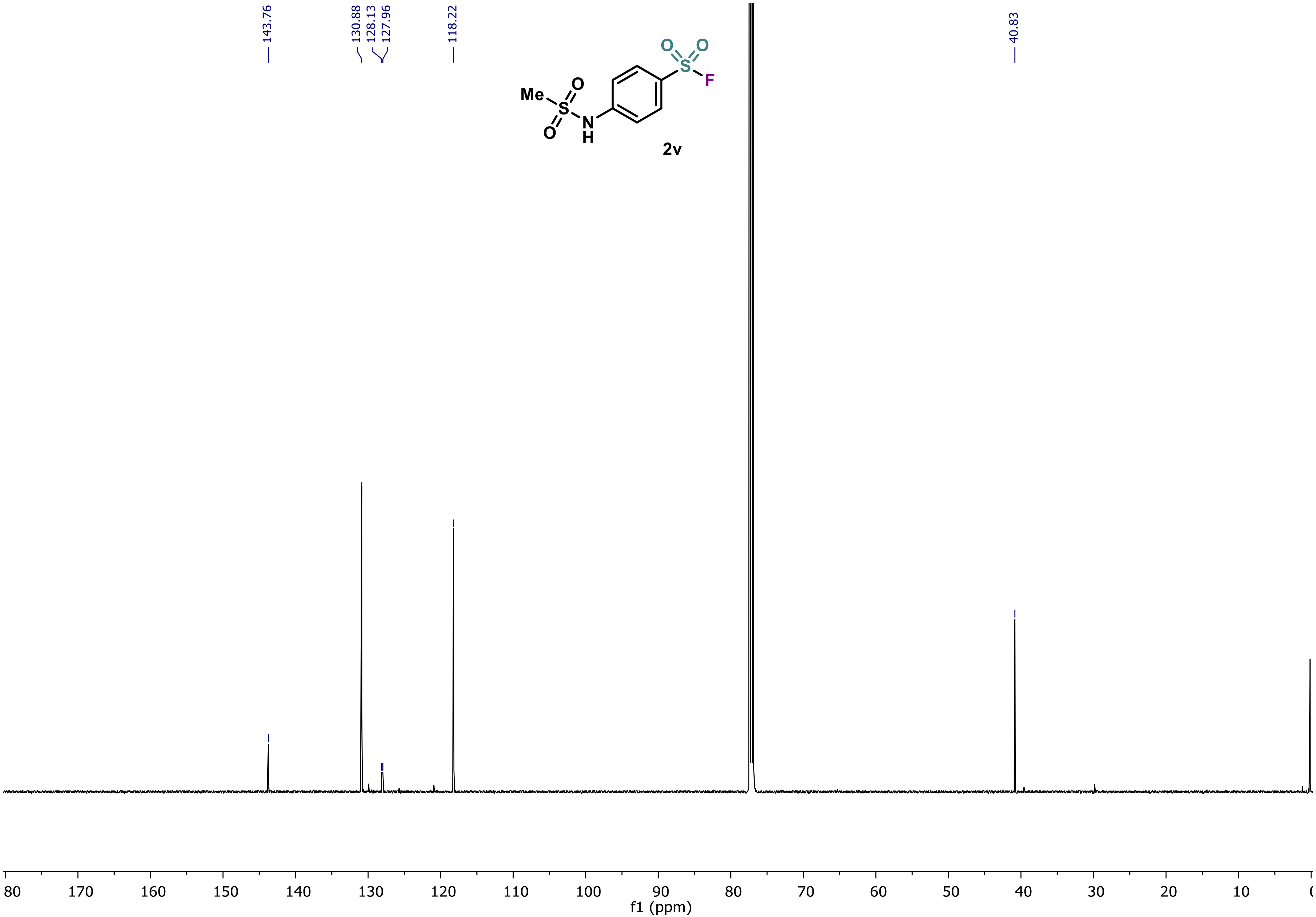

2v

^{19}F NMR (565 MHz, $CDCl_3$)

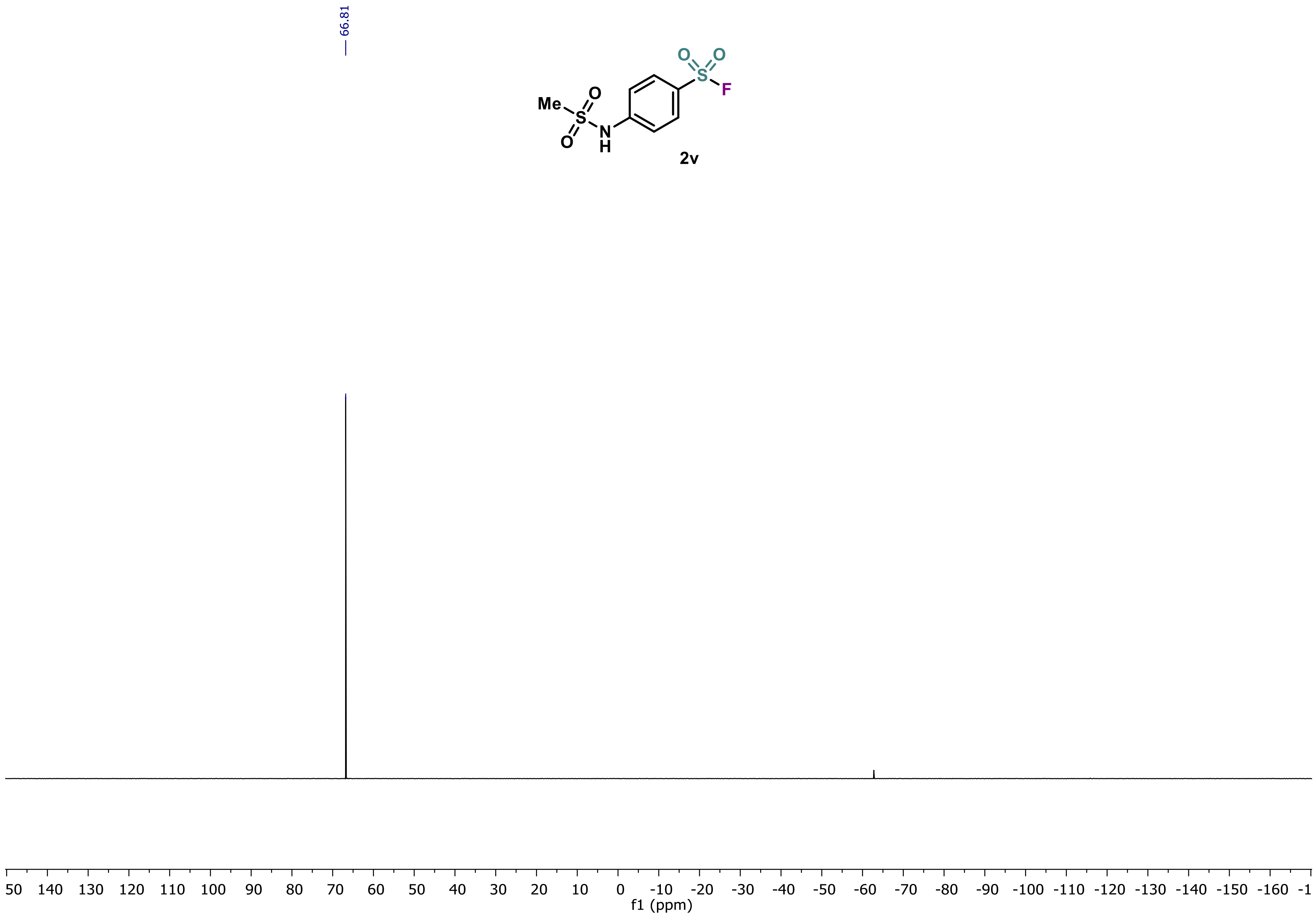

¹H NMR (600 MHz, $CDCl_3$)

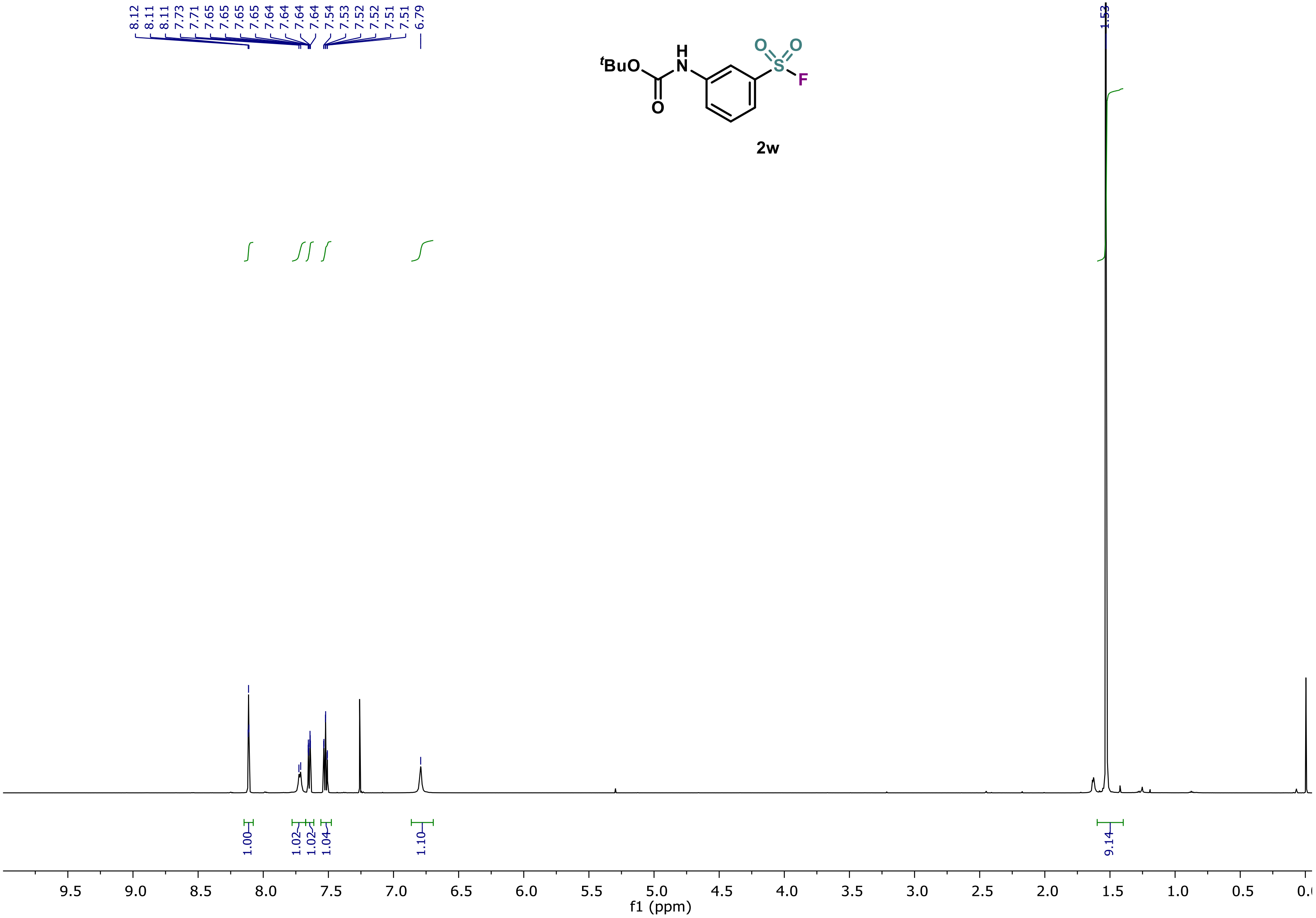

^{13}C NMR (151 MHz, CDCl$_3$)

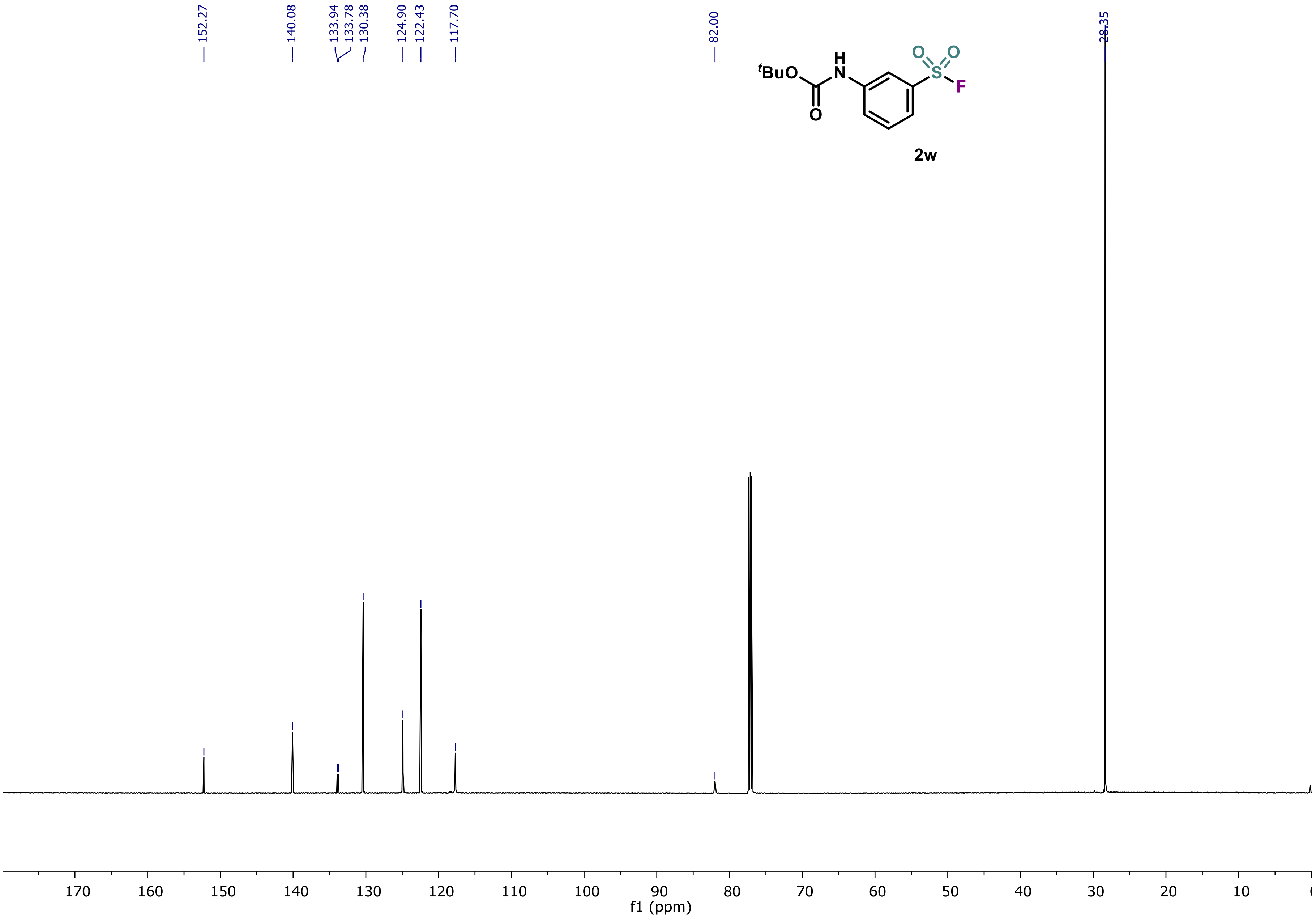

^{19}F NMR (565 MHz, $CDCl_3$)

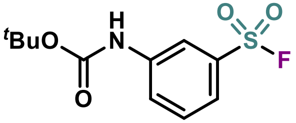

2w

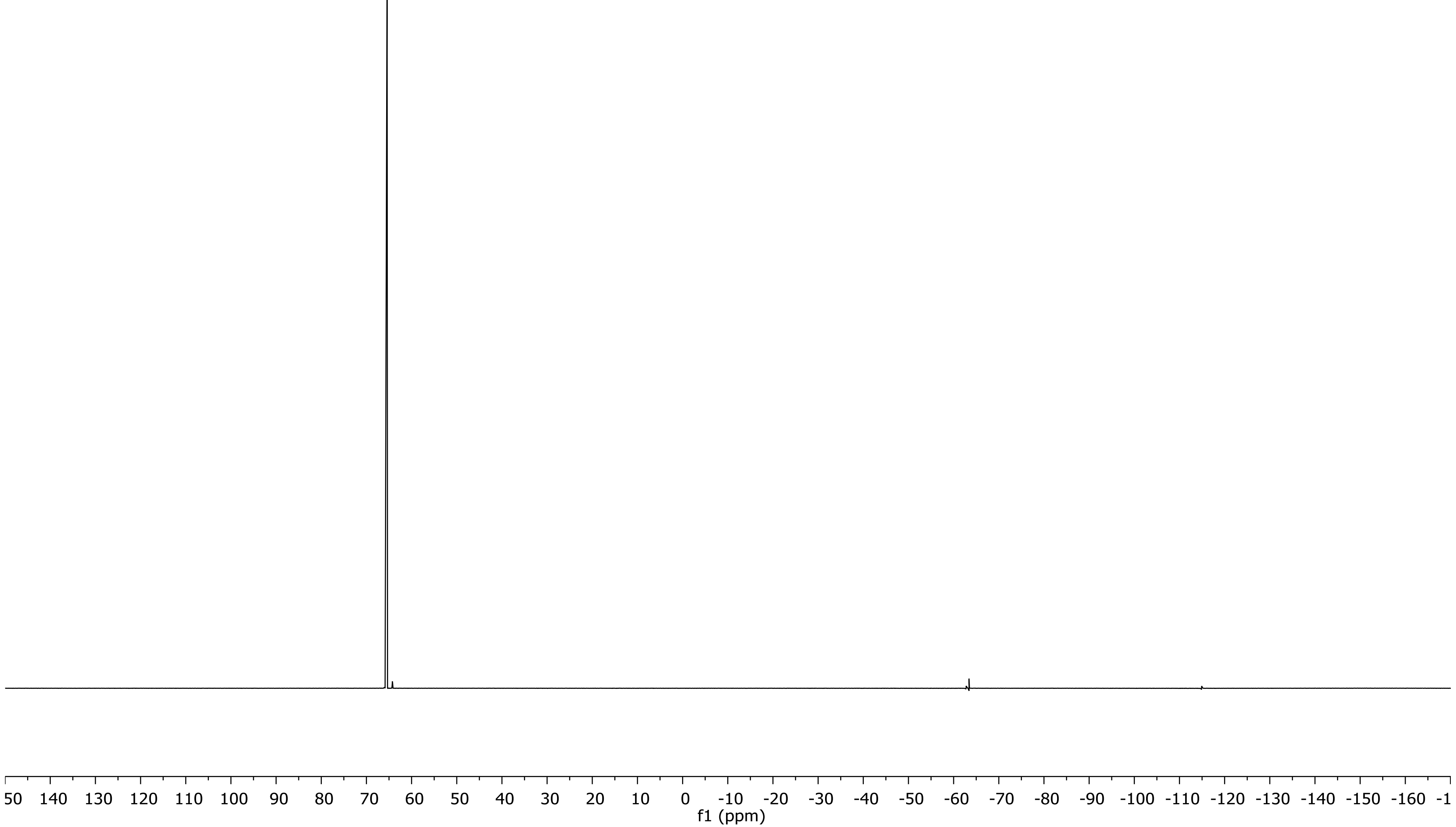

^{1}H NMR (600 MHz, $CDCl_3$)

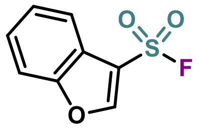

2x

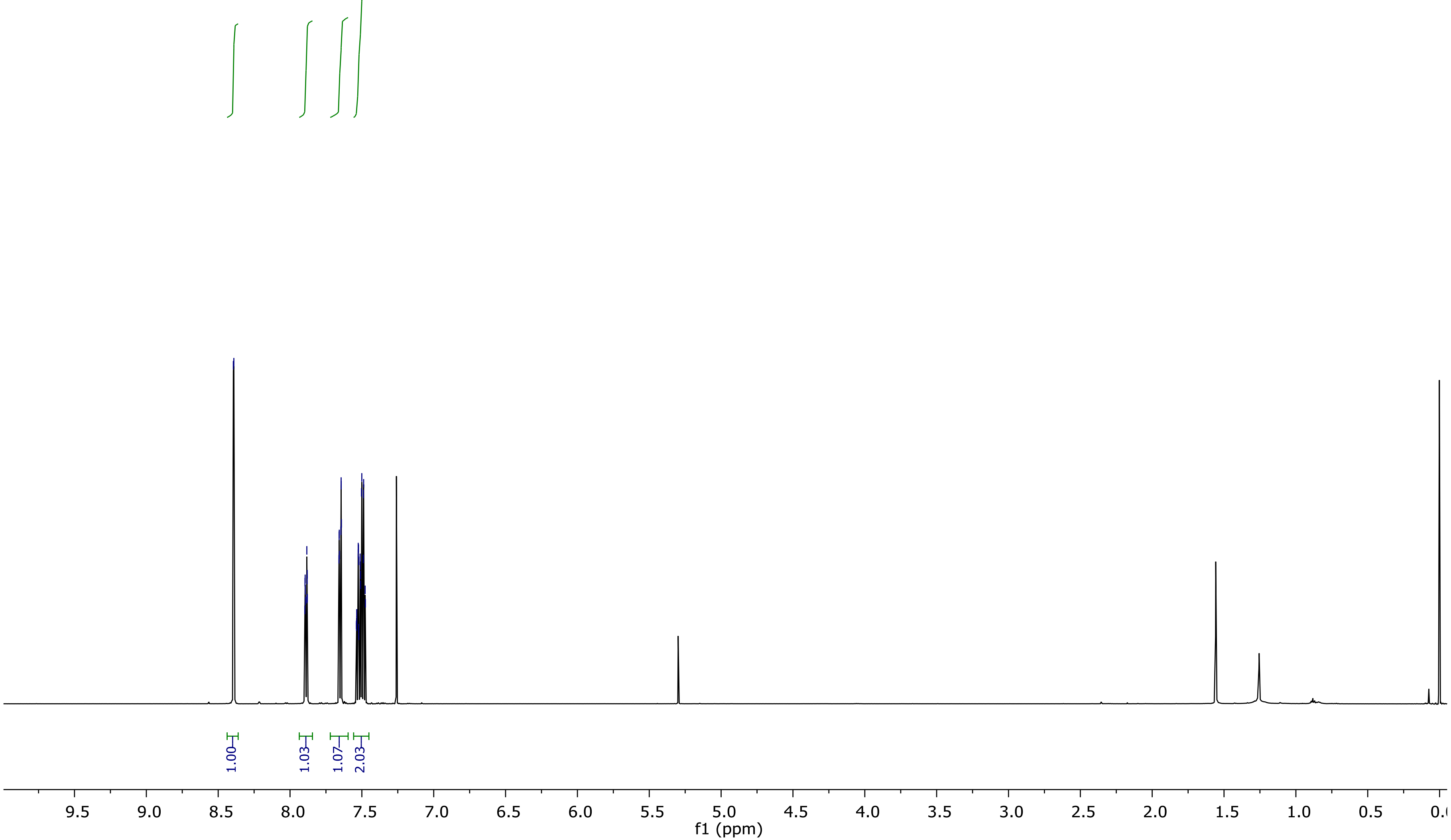

^{13}C NMR (151 MHz, $CDCl_3$)

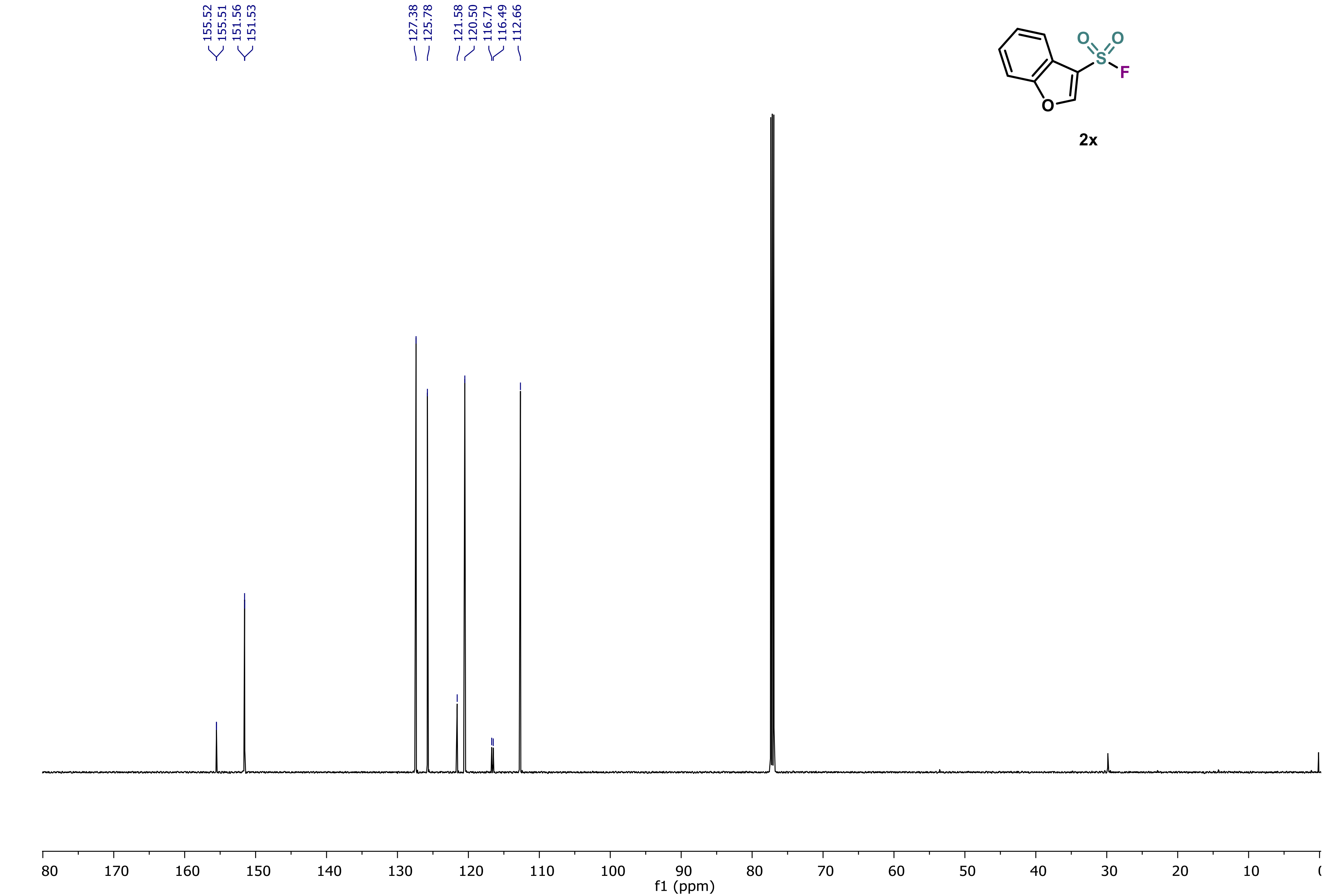

^{19}F NMR (565 MHz, $CDCl_3$)

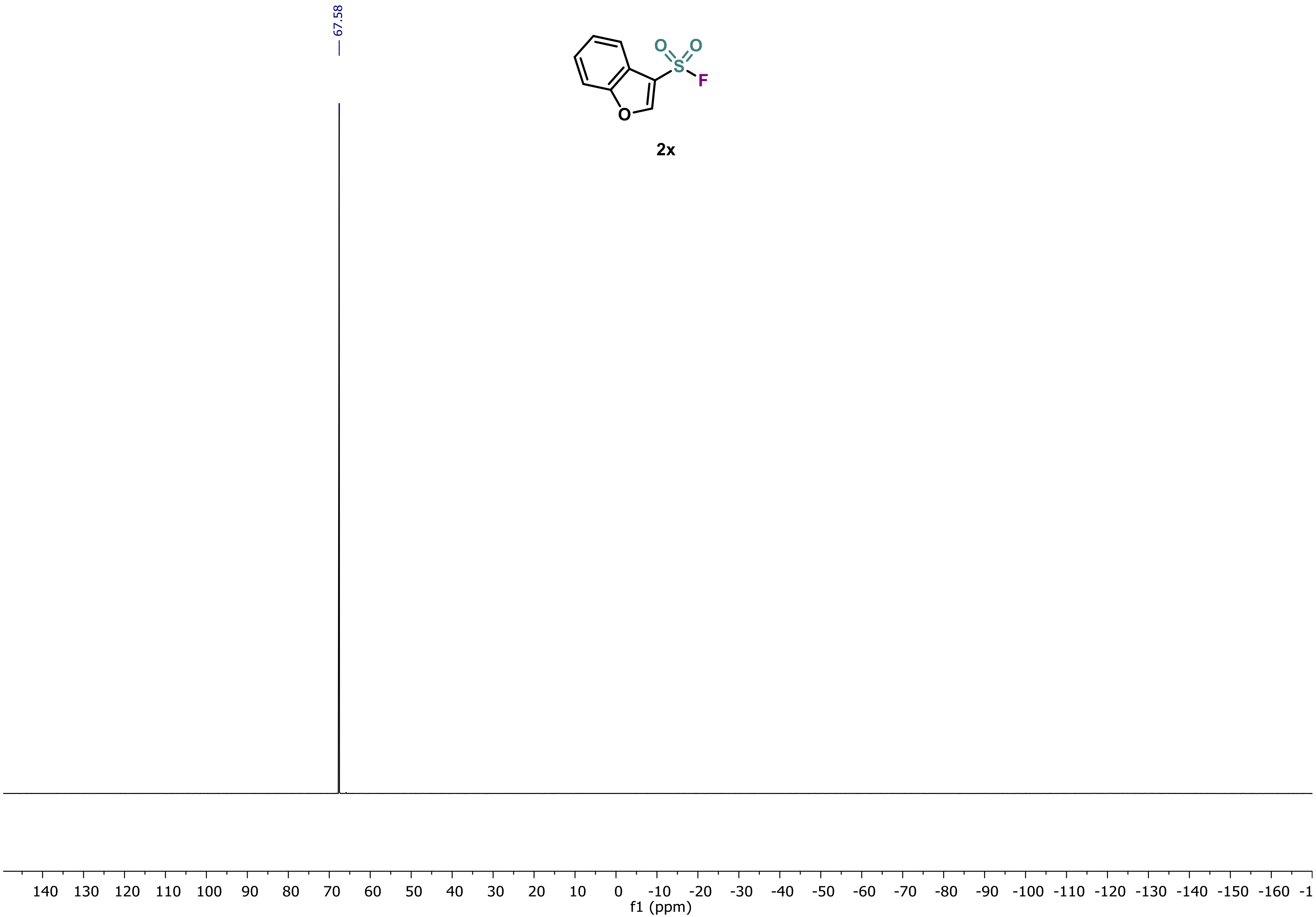

2x

^{1}H NMR (600 MHz, $CDCl_3$)

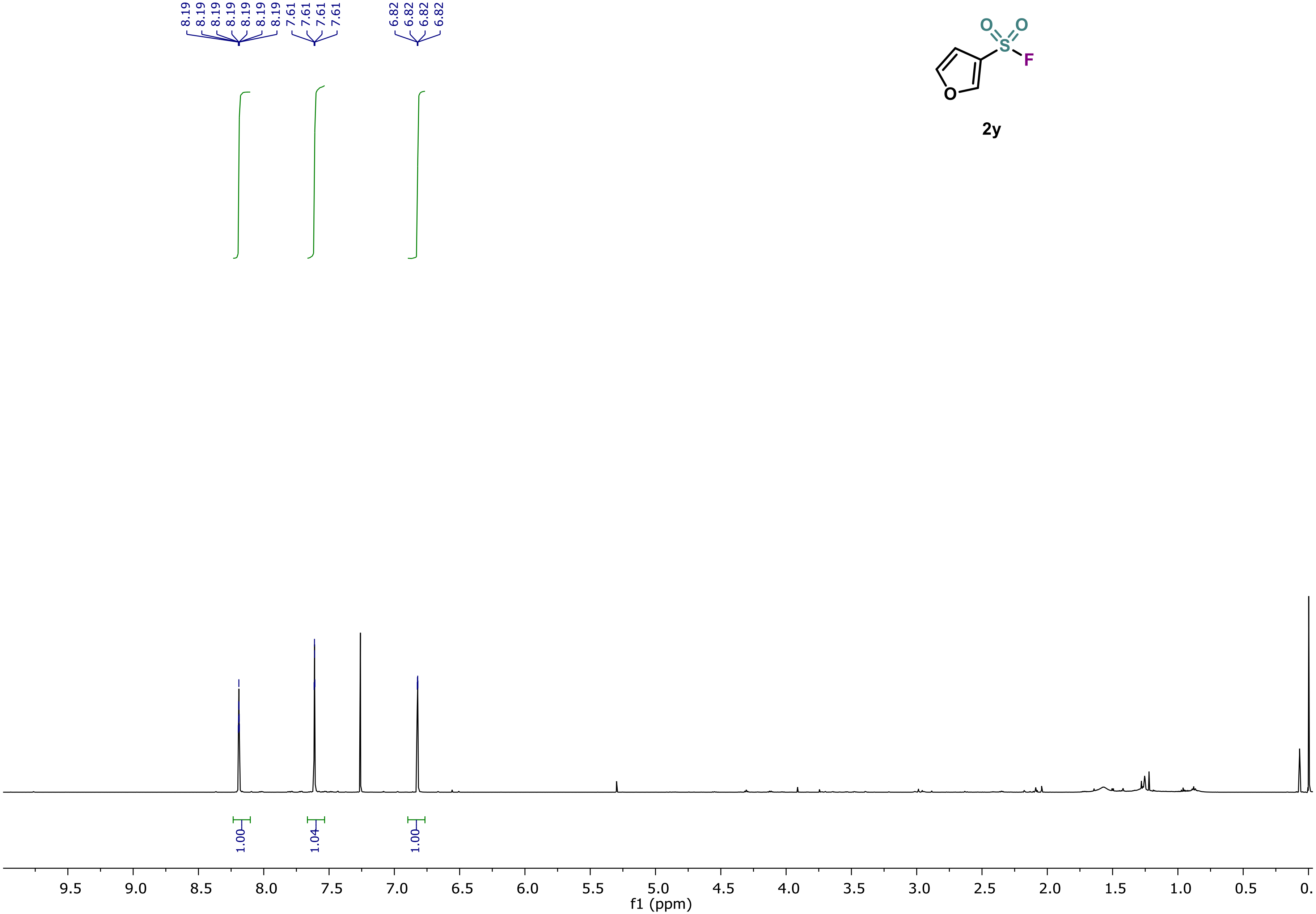

2y

^{13}C NMR (151 MHz, $CDCl_3$)

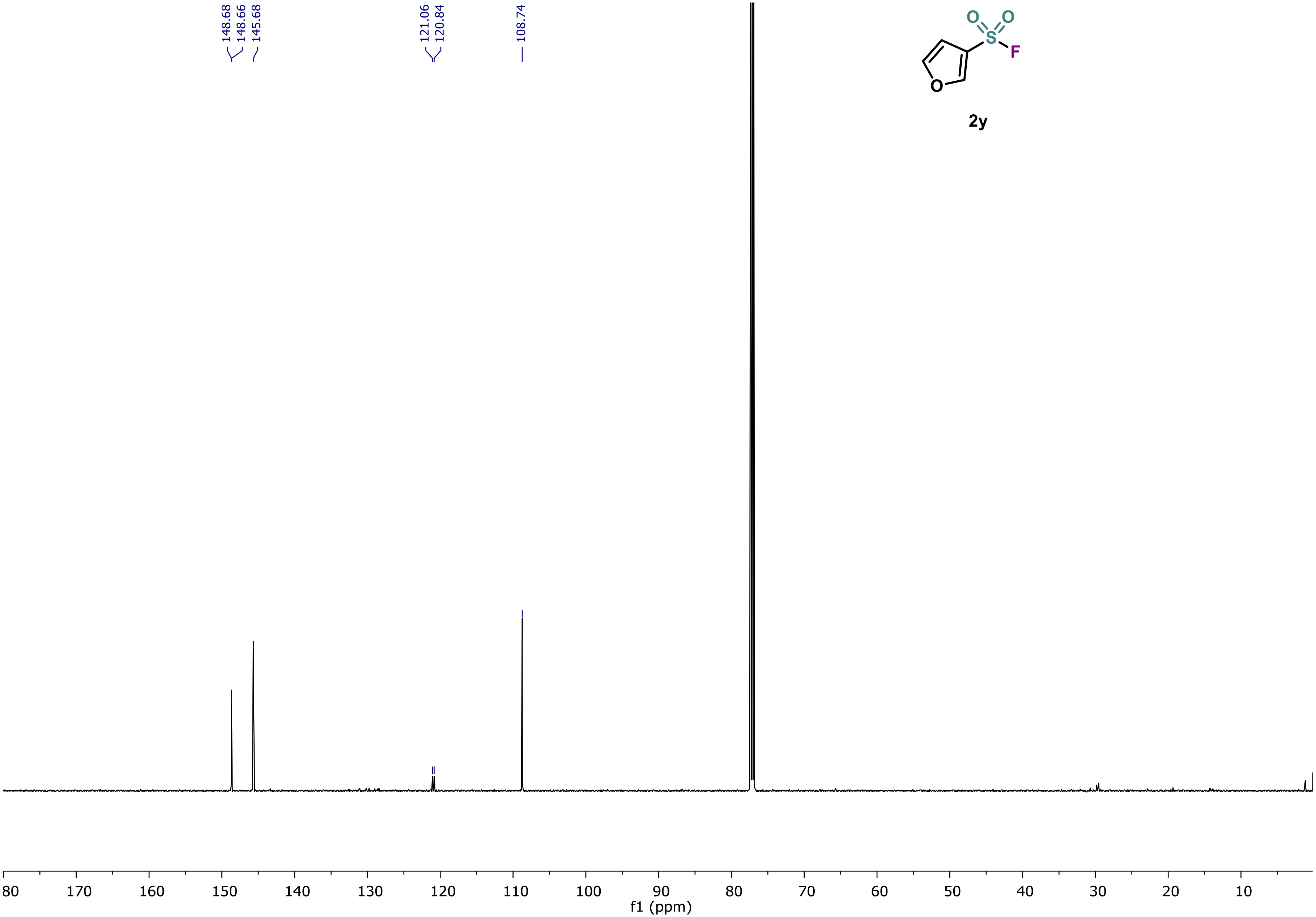

2y

^{19}F NMR (565 MHz, $CDCl_3$)

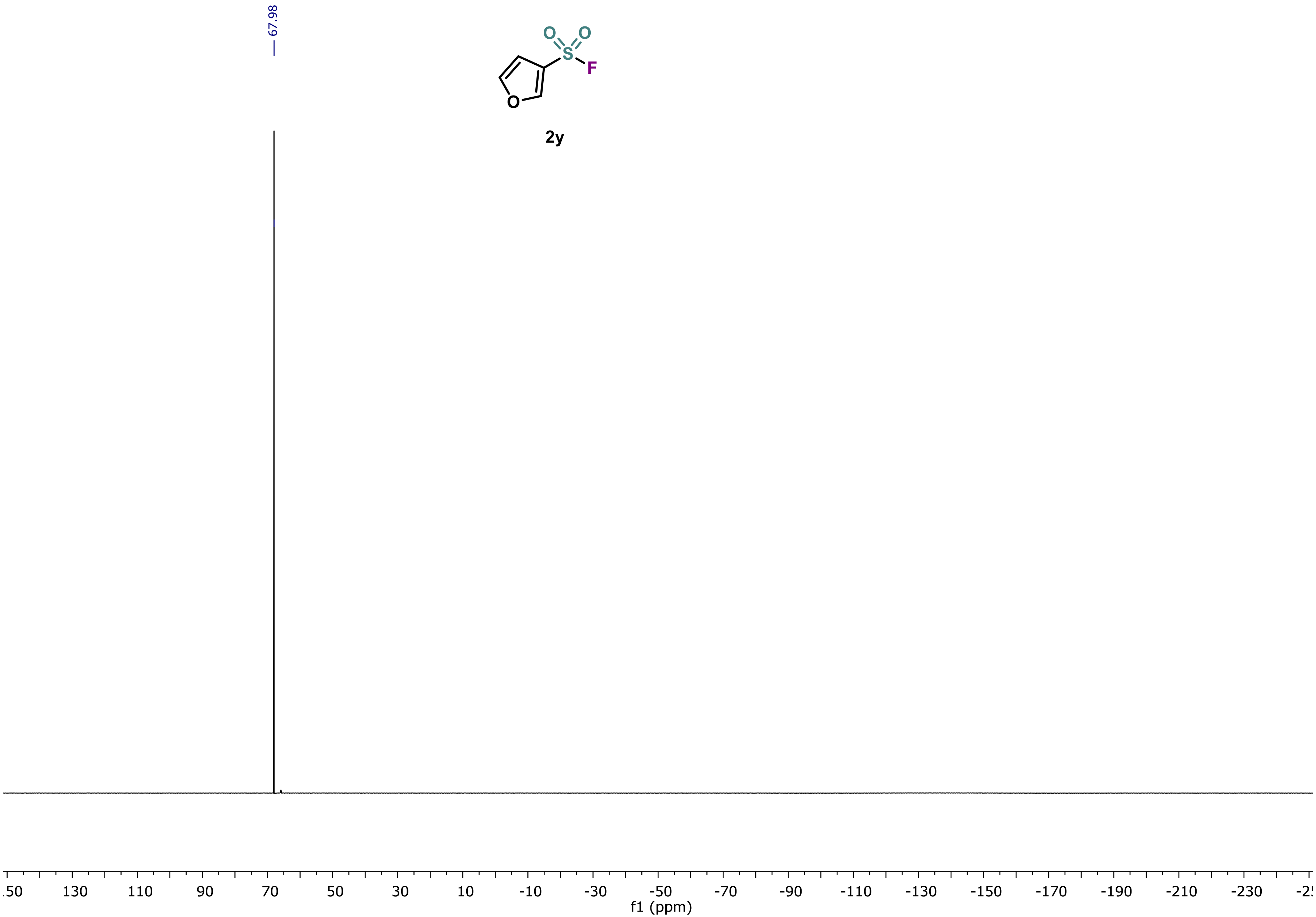

^{1}H NMR (600 MHz, $CDCl_3$)

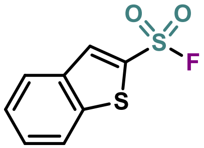

2z

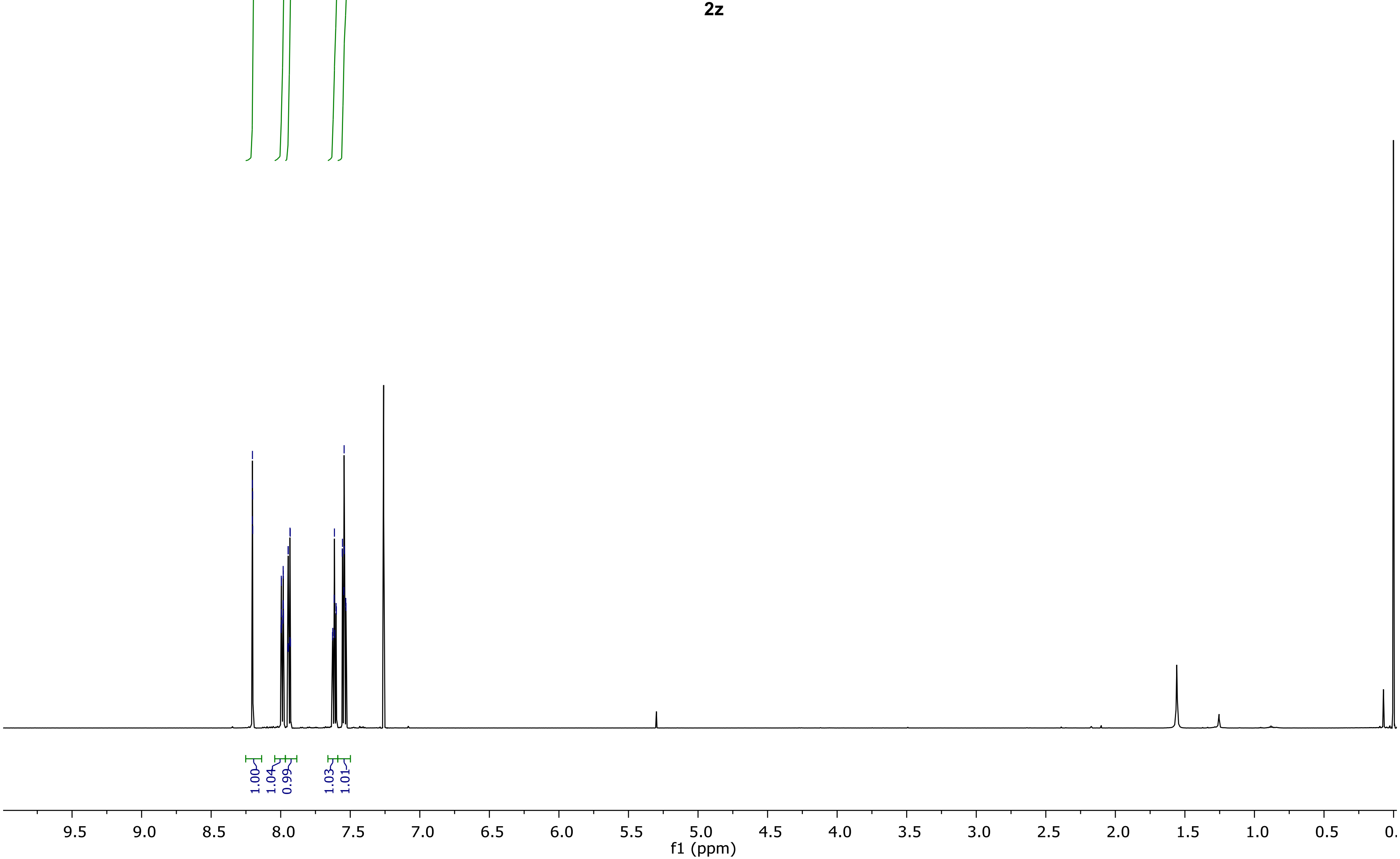

^{13}C NMR (151 MHz, $CDCl_3$)

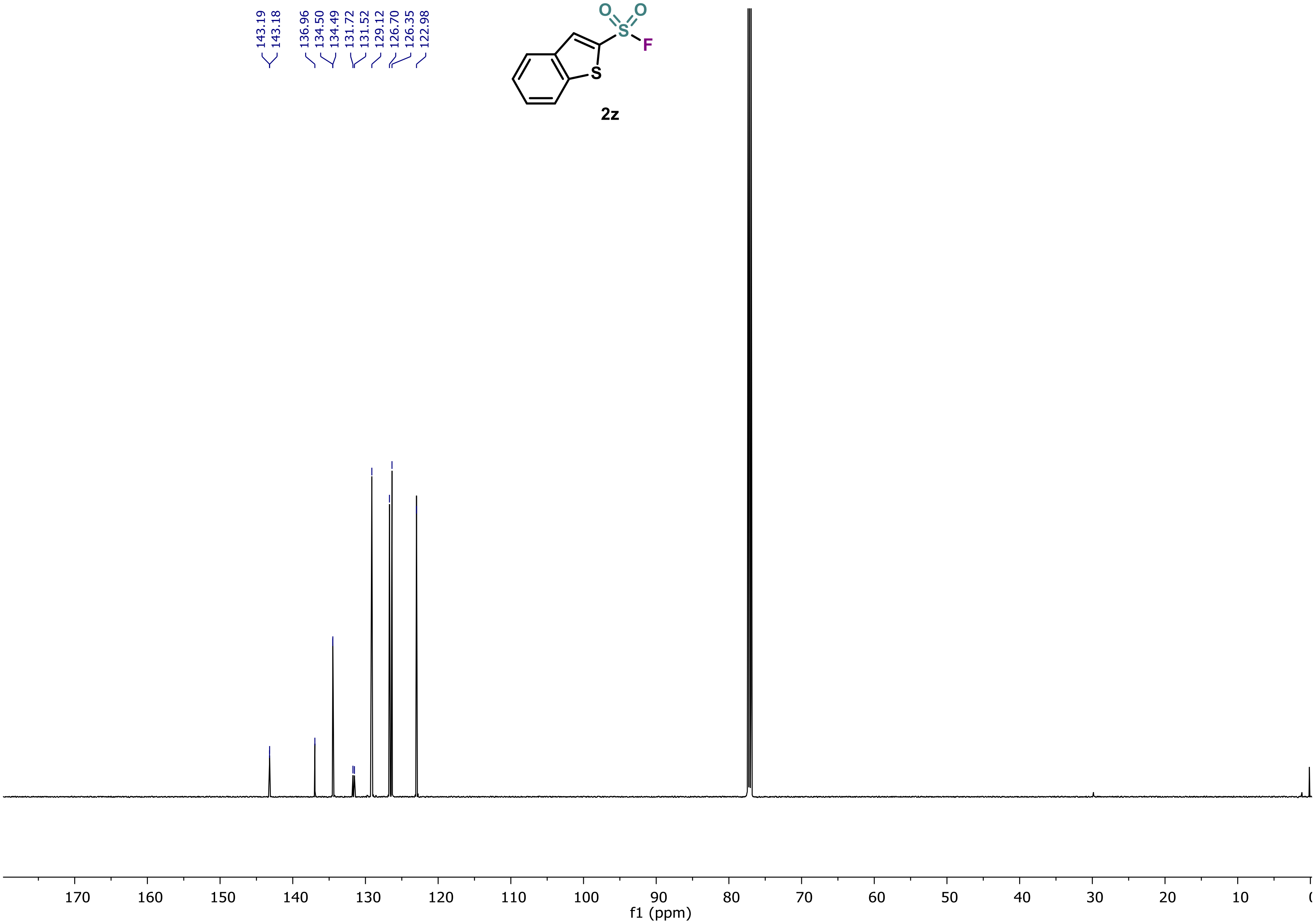

^{19}F NMR (565 MHz, $CDCl_3$)

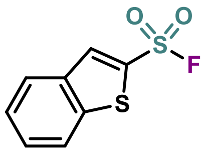

2z

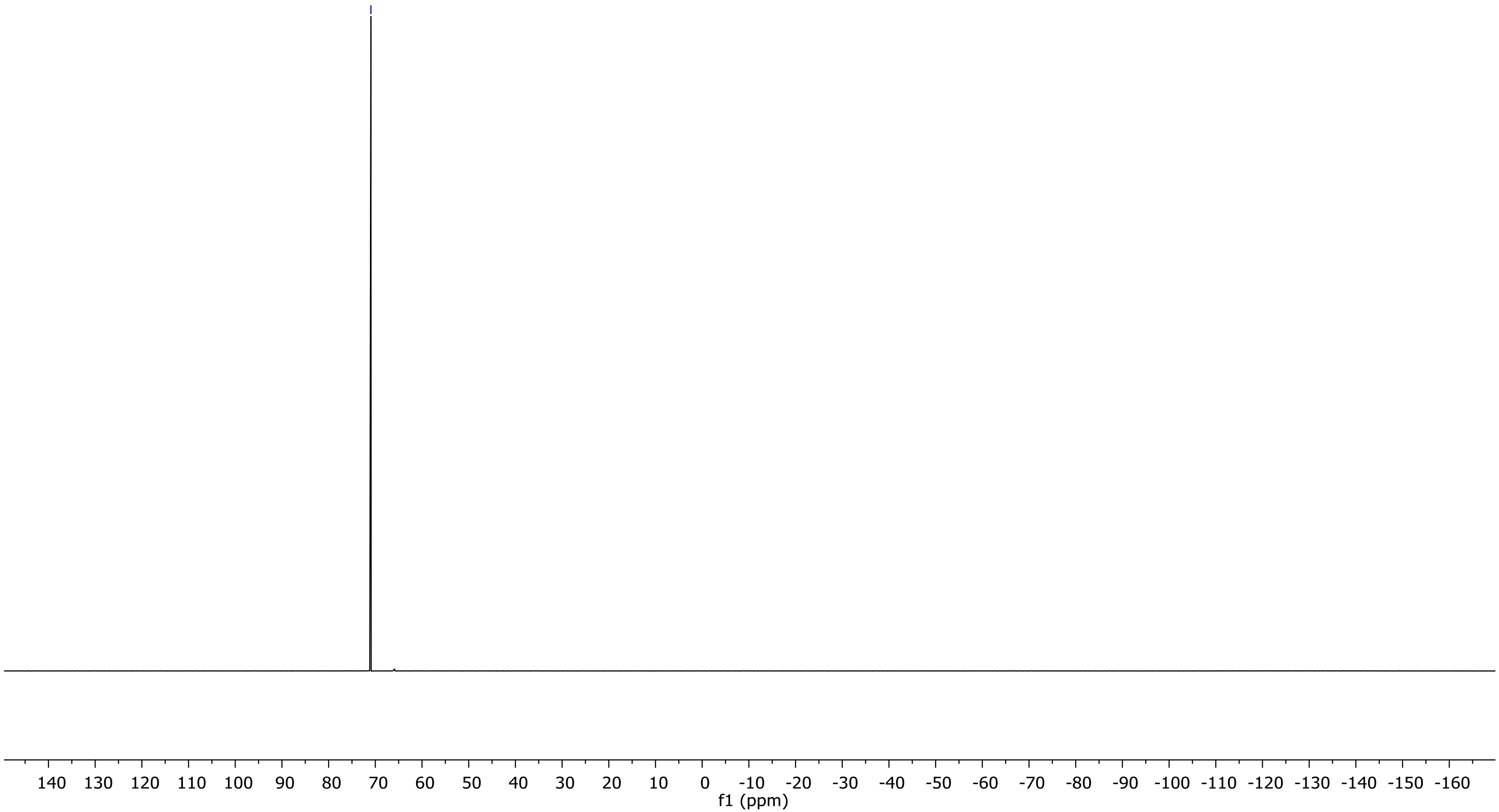

^{1}H NMR (600 MHz, $CDCl_3$)

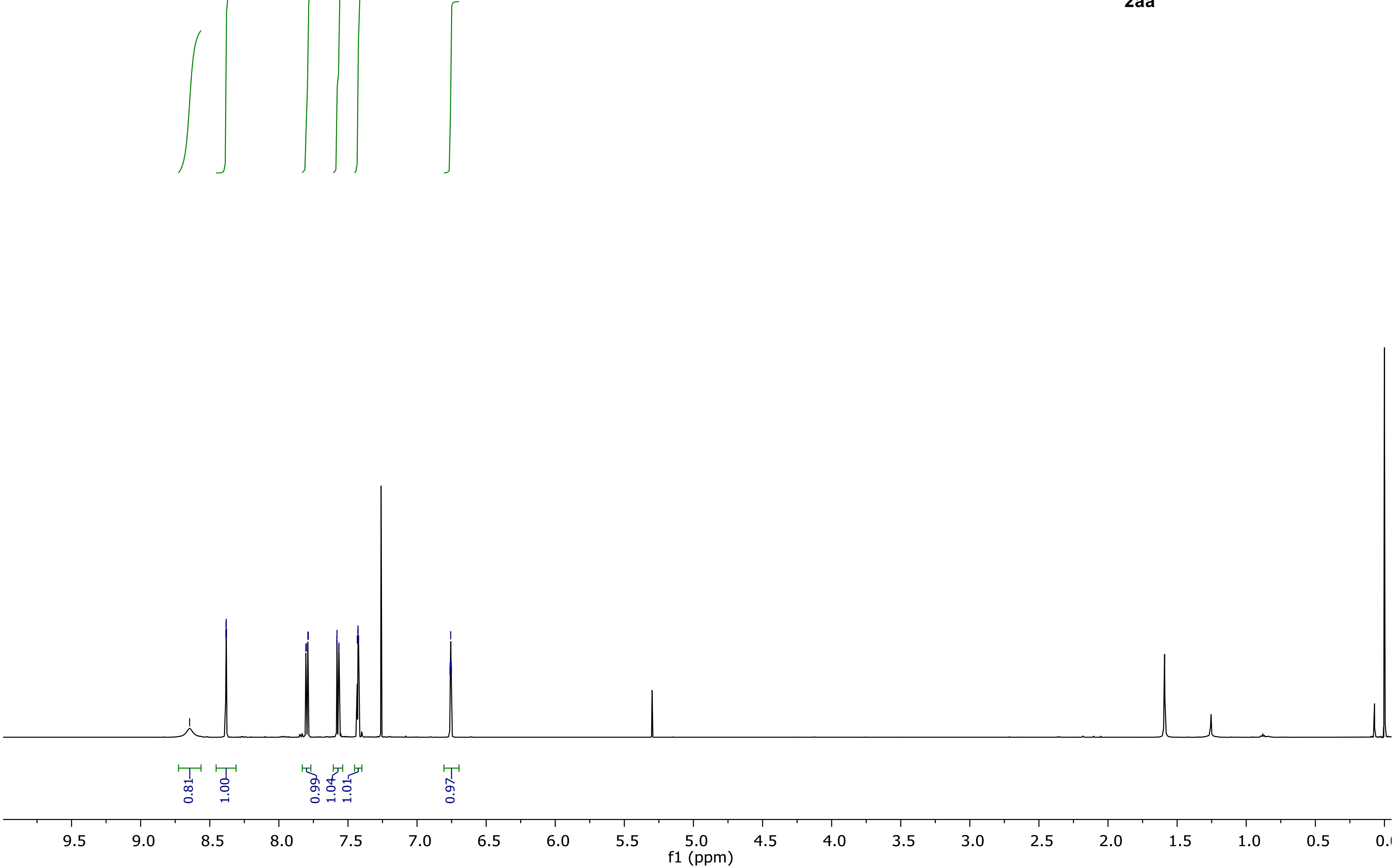

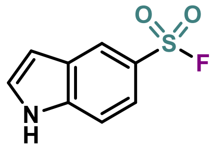

2aa

^{13}C NMR (151 MHz, $CDCl_3$)

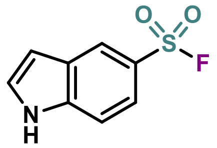

2aa

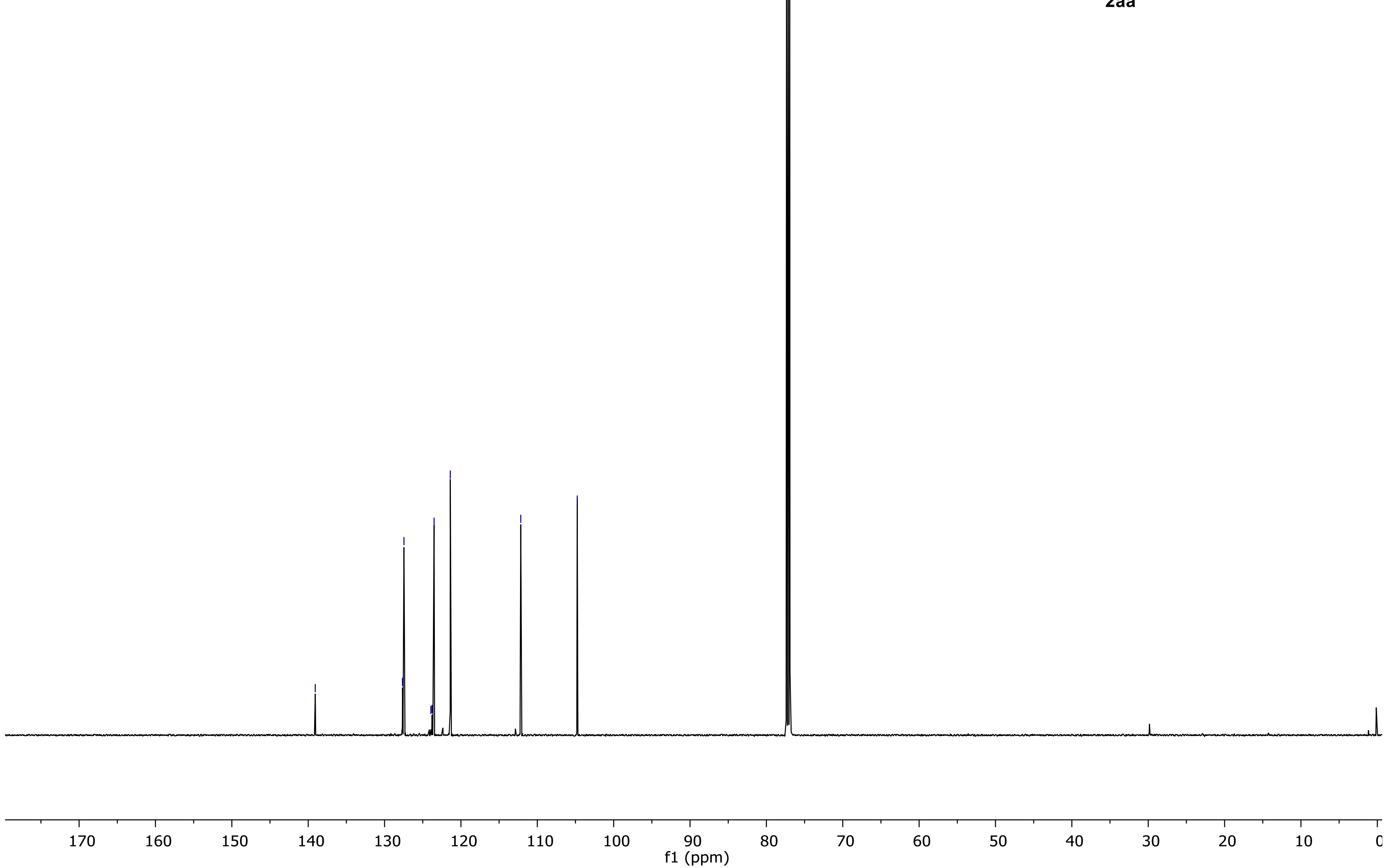

^{19}F NMR (565 MHz, CDCl$_3$)

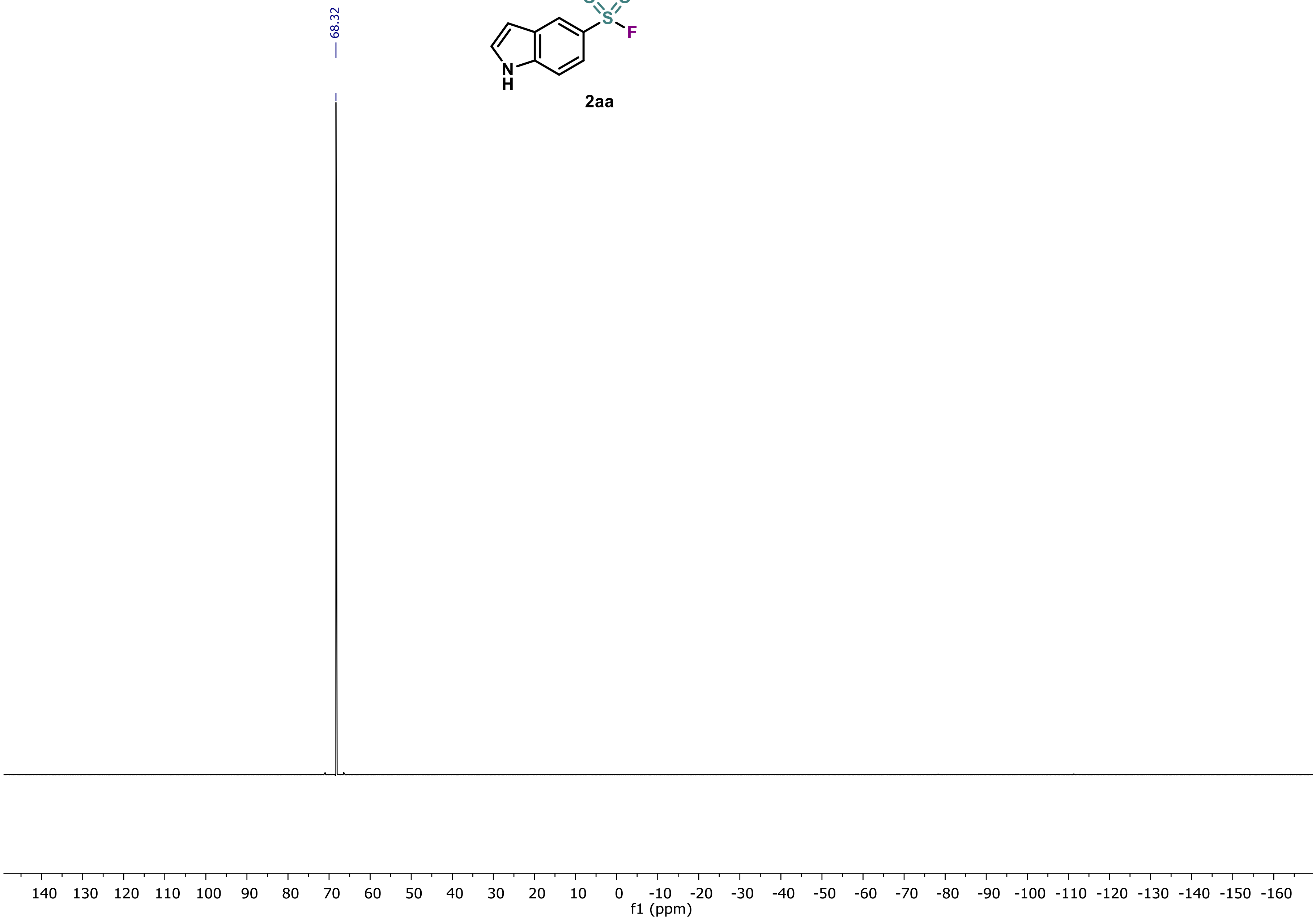

2aa

¹H NMR (600 MHz, CDCl$_3$)

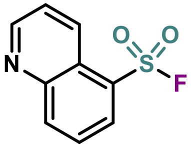

2ab

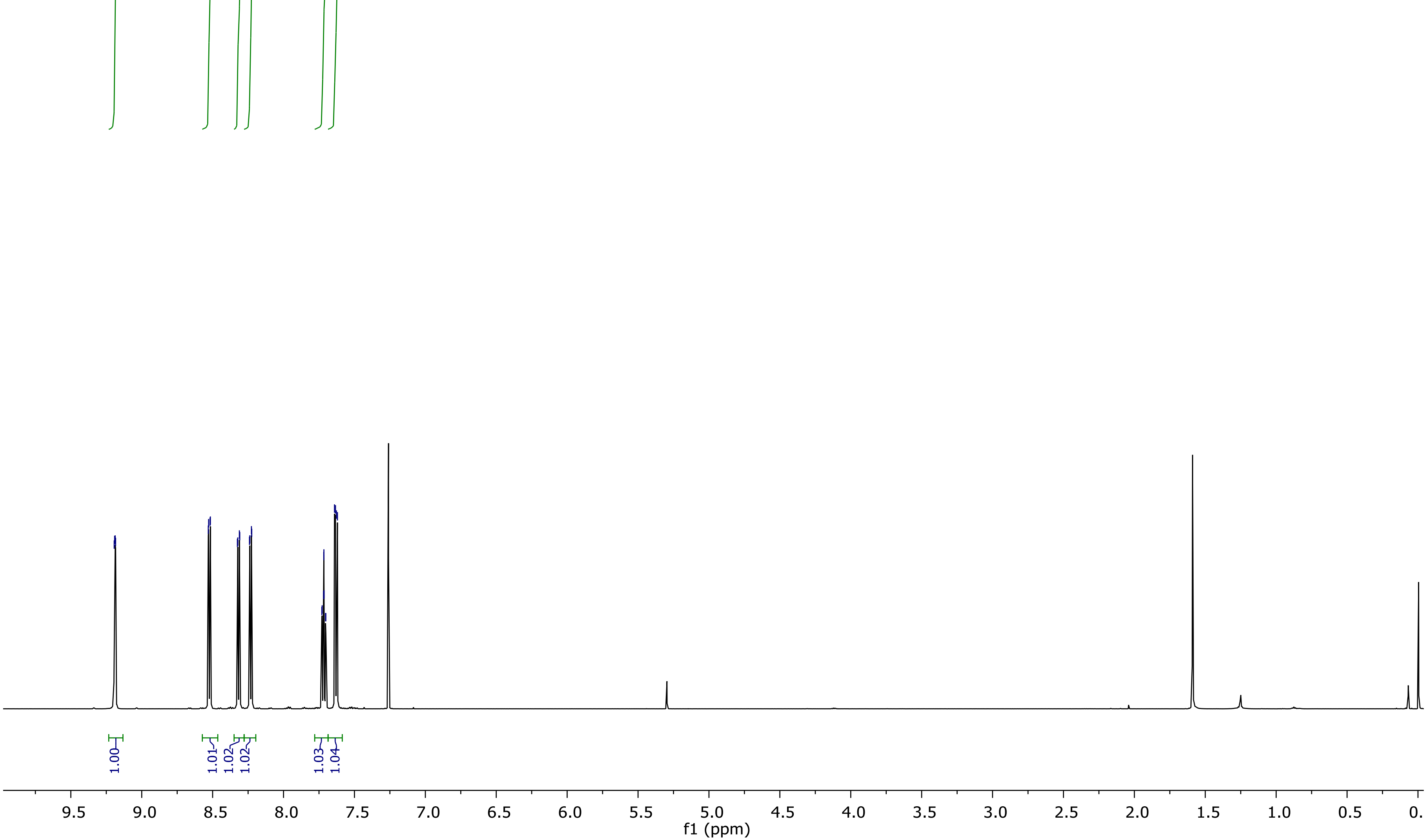

^{13}C NMR (151 MHz, $CDCl_3$)

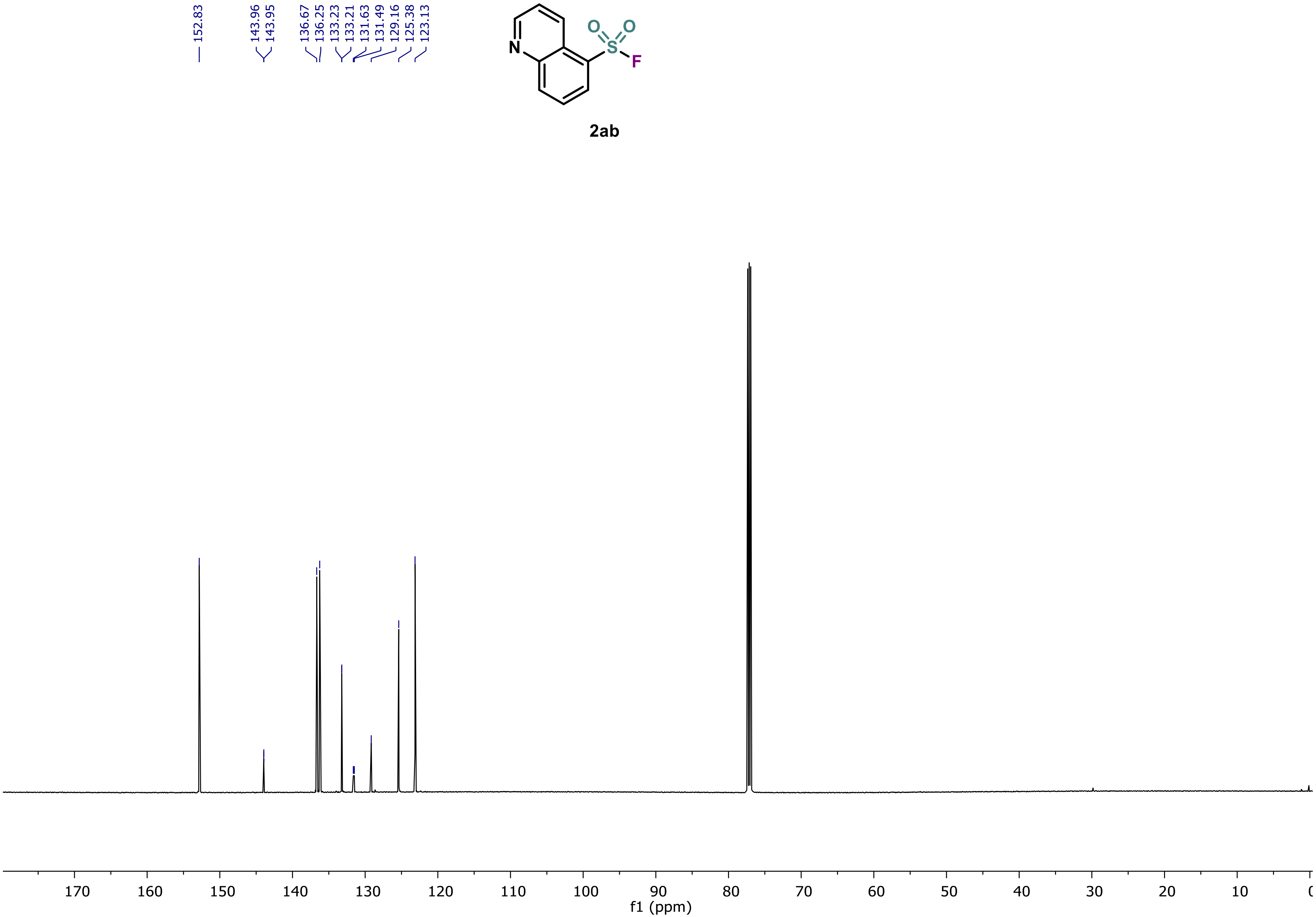

^{19}F NMR (565 MHz, $CDCl_3$)

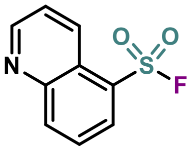

2ab

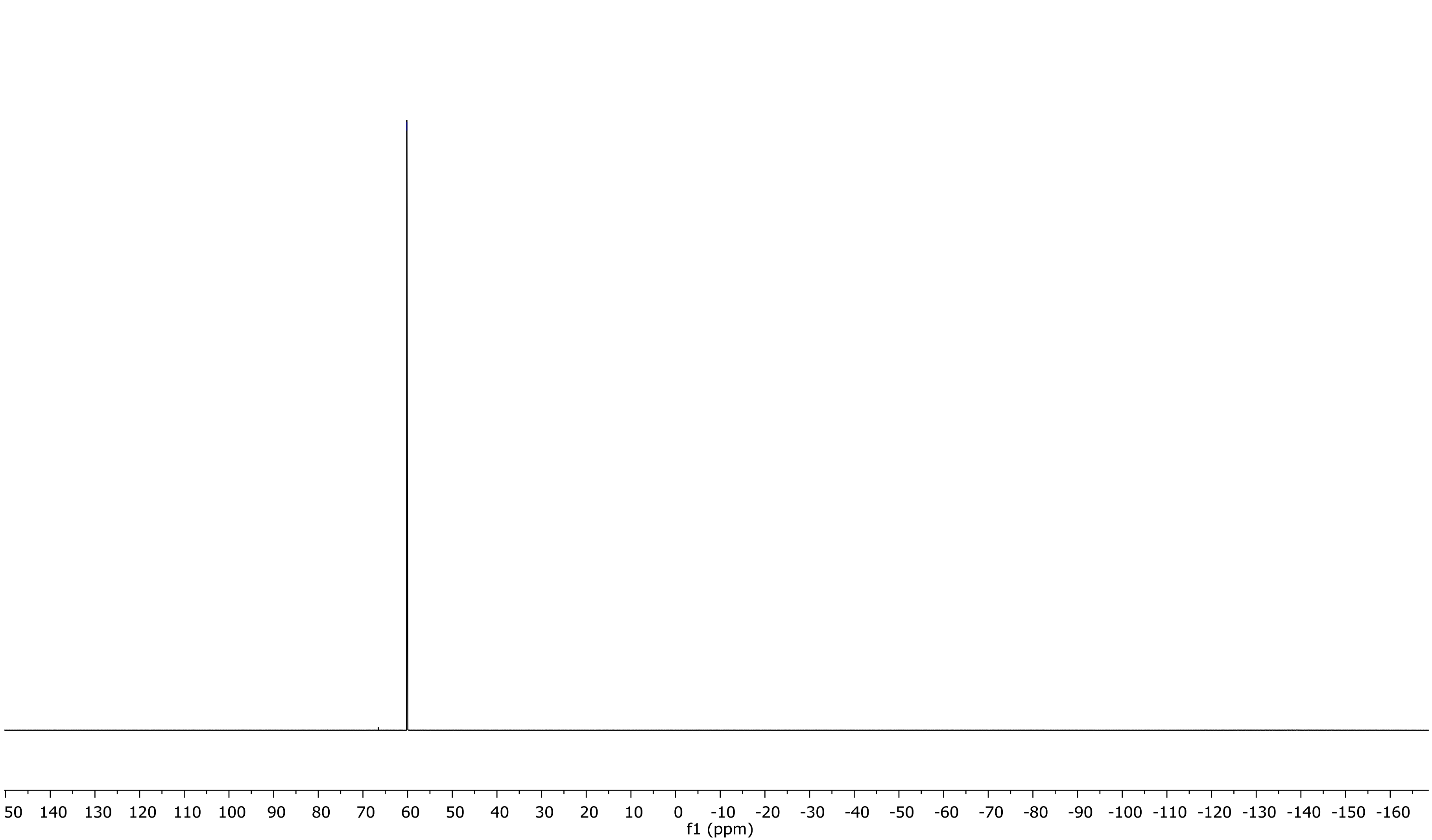

^{1}H NMR (600 MHz, $CDCl_3$)

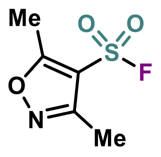

2ac

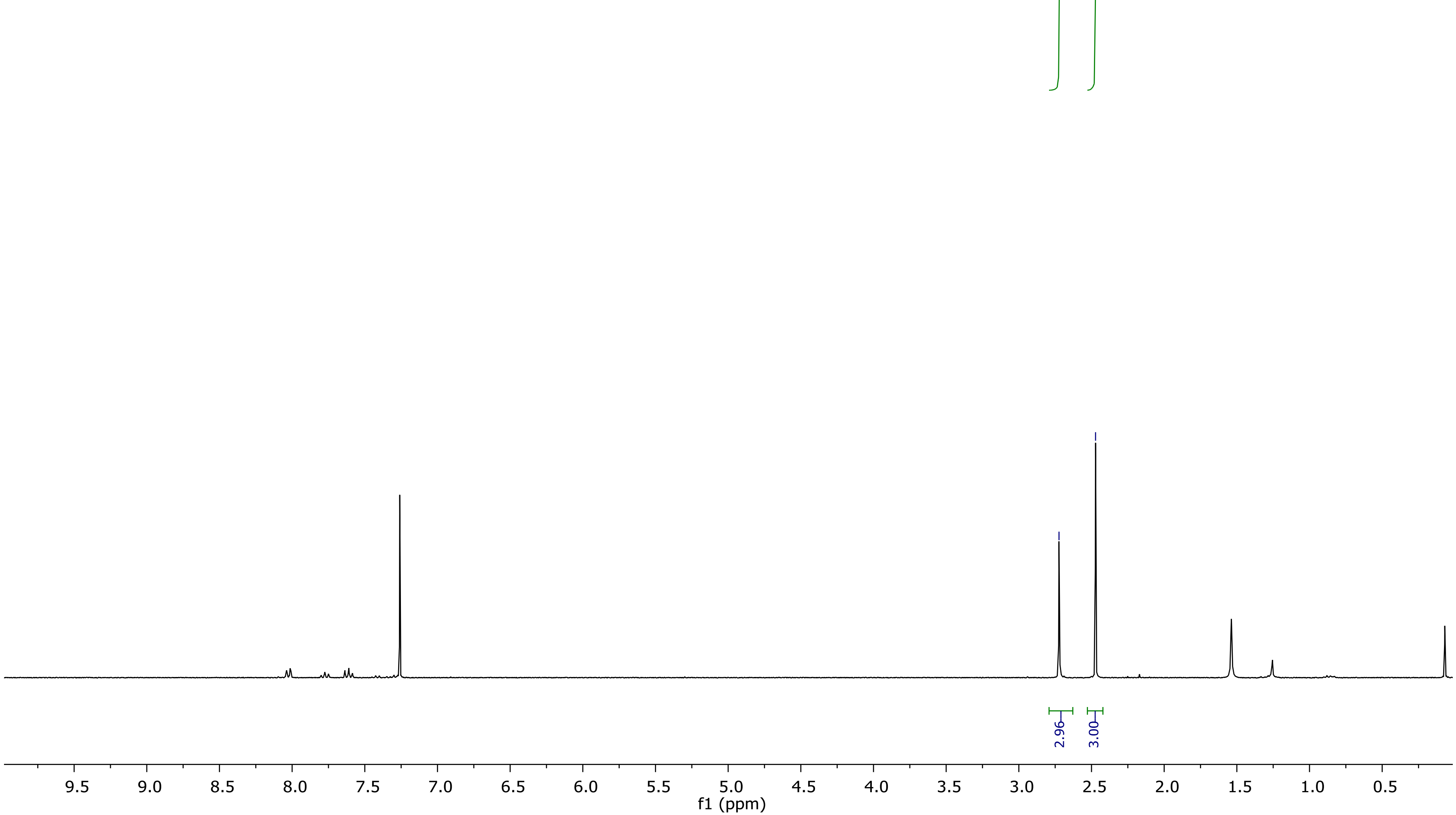

^{13}C NMR (151 MHz, $CDCl_3$)

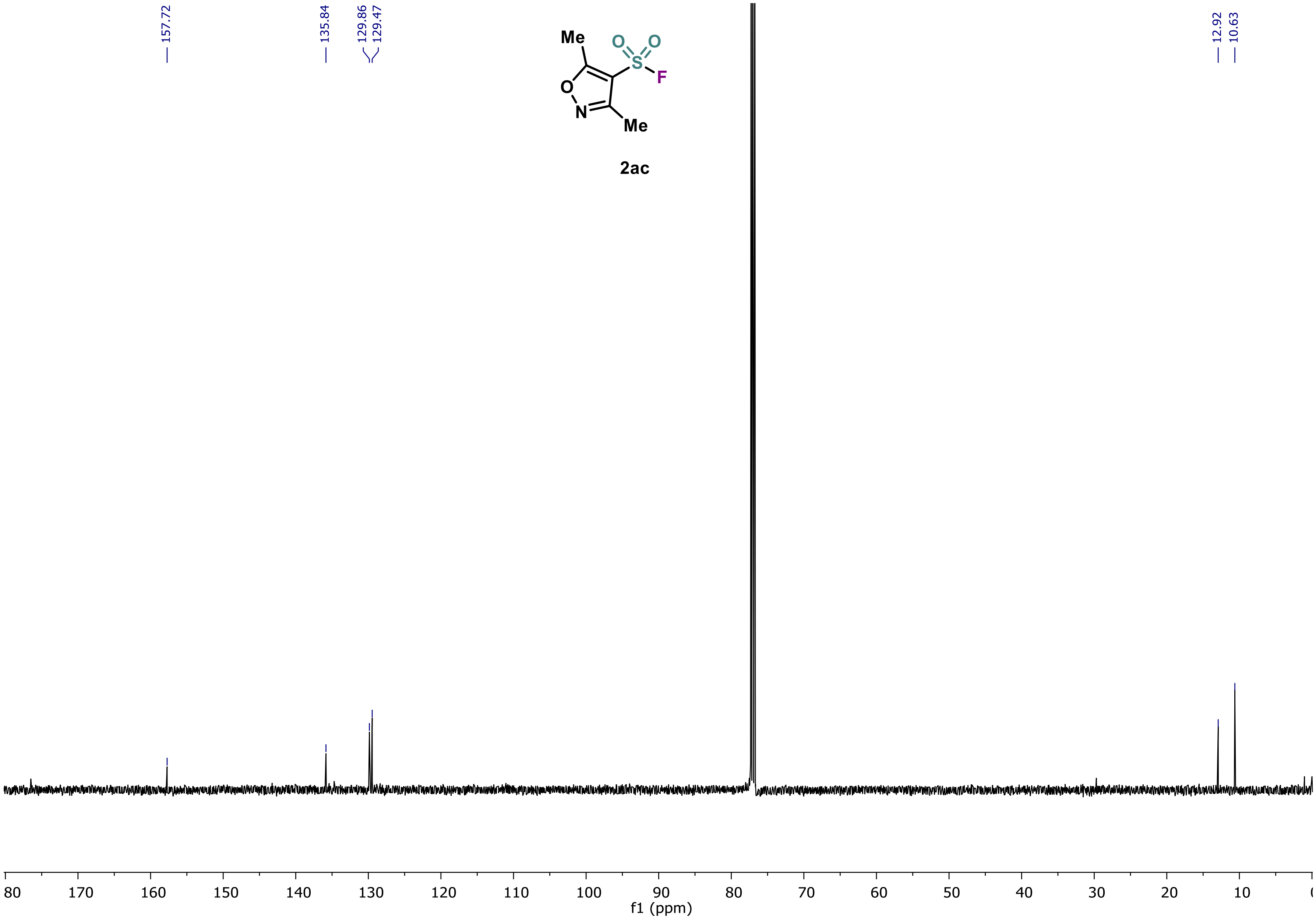

^{19}F NMR (565 MHz, $CDCl_3$)

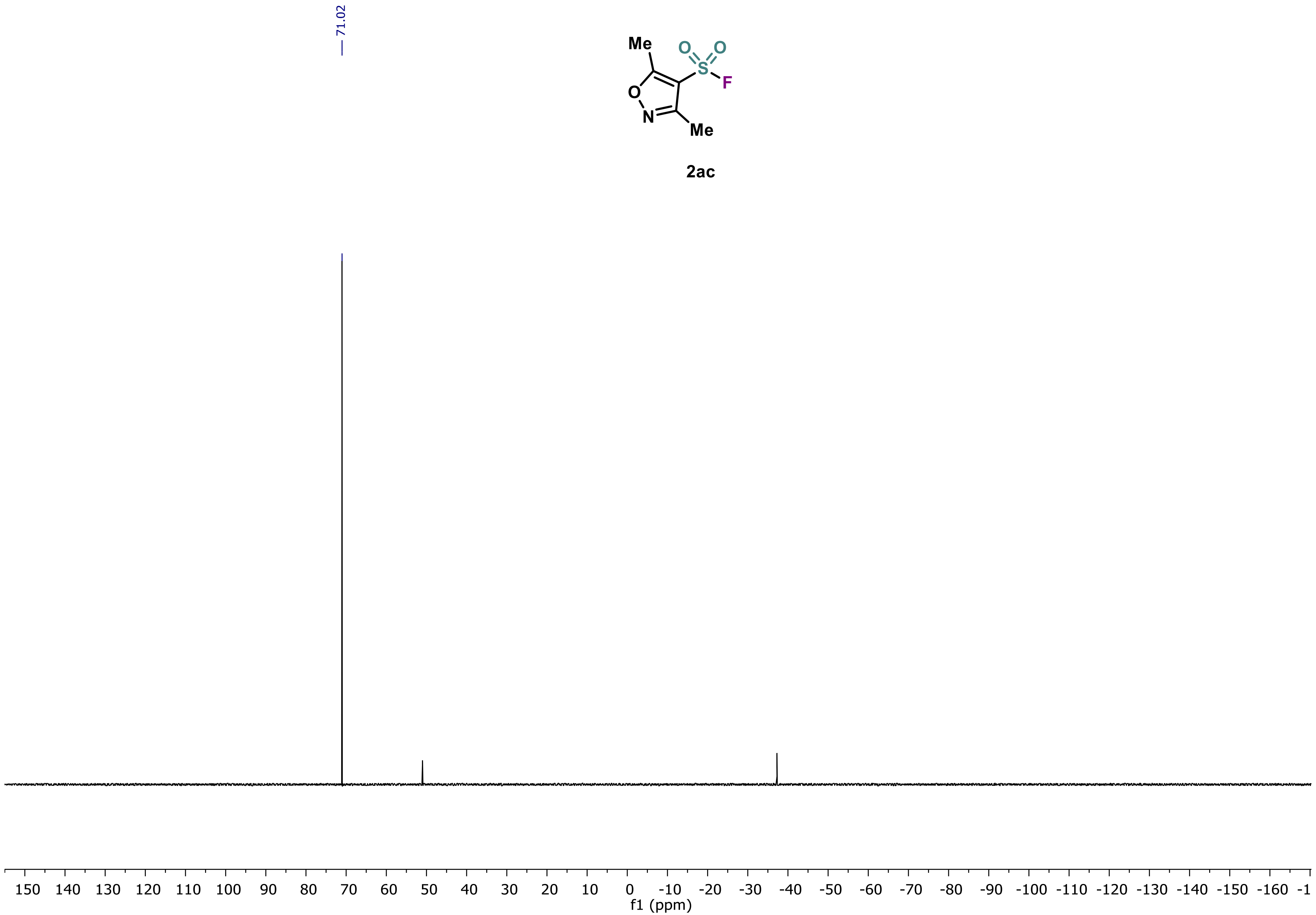

Supplement: Supplementary file 1 — ja1c11463_si_001.pdf [file ja1c11463_si_001.pdf]
